# Supplementary material for: Virologic suppression among HIV-positive pregnant and lactating women receiving antiretroviral therapy in Africa: A systematic review and meta-analysis
Source: PLoS One. 2026 Apr 13;21(4):e0346045. doi: 10.1371/journal.pone.0346045 (PMC13075719; doi:10.1371/journal.pone.0346045)
Supplement: S2 File — (DOCX) [file pone.0346045.s002.docx]

**PubMed**

((virological suppression) [All Fields] OR ((virologic suppression) [All Fields] OR (viral suppression)) [All Fields] OR (viral load non-suppression) [All Fields] OR (detectable viral load)) [All Fields] OR (undetectable viral load)) [All Fields] OR (plasma viremia)) [All Fields] OR (HIV viremia)) AND (pregnant)) OR (lactating)) OR (postpartum) AND (HIV-positive women)) [All Fields] AND (("antiretroviral therapy") [All Fields] OR (ART) AND (Africa))))) [All Fields]

**Hinari**

((virological) OR ((virologic) OR ("viral load")) OR (viral)) AND ((suppression) OR (non-suppression) OR (plasma viremia)) OR (viral suppression)) OR (HIV viremia)) OR (pregnant)) OR (lactating)) OR (postpartum) OR (HIV-positive women)) AND (("antiretroviral therapy") OR (ART) AND (Africa)))))))

**Science Direct**

((virologic suppression) OR ((virological suppression) OR (viral load suppression) OR HIV viral suppression AND “HIV-positive pregnant OR “postpartum women” OR “breastfeeding mothers” AND (Africa))

**Google Scholar**

((virologic suppression) OR ((virological suppression) OR (viral load suppression) OR HIV viral load OR “plasma viremia” OR HIV viral suppression OR “HIV viremia” AND “HIV-positive women” AND pregnant OR “lactating mothers” OR “postpartum women” OR “breastfeeding mothers” AND ("antiretroviral therapy") OR (ART) AND (Africa))
