# Supplementary material for: Virologic suppression among HIV-positive pregnant and lactating women receiving antiretroviral therapy in Africa: A systematic review and meta-analysis
Source: PLoS One. 2026 Apr 13;21(4):e0346045. doi: 10.1371/journal.pone.0346045 (PMC13075719; doi:10.1371/journal.pone.0346045)
Supplement: S4 Table — (DOCX) [file pone.0346045.s004.docx]

| Authors | Study design | Country | Study population | Sample size | VS (%) | JBI score |
| --- | --- | --- | --- | --- | --- | --- |
| Adeniyi et al., 2021 | CS | South Africa | Pregnant and postpartum | 1463 | 82 | 9 |
| Aduloju et al., 2020 | CS | Nigeria | Pregnant women | 170 | 70 | 7 |
| Alamneh et al., 2023 | CS | Ethiopia | Pregnant women | 992 | 90.9 | 8 |
| Anderson et al., 2024 | CH | South Africa | Pregnant women | 50764 | 87.4 | 8 |
| Anderson et al., 2024 | CH | South Africa | Postpartum women | 50764 | 90.9 | 9 |
| Anderson et al., 2025 | CH | South Africa | Intrapartum women | 50764 | 83.8 | 9 |
| Boisson-Walsh et al et al., 2024 | CS | DR Congo | Pregnant and postpartum | 2295 | 66.5 | 8 |
| Brittain et al., 2018 | CS | South Africa | Pregnant women | 482 | 88 | 9 |
| Chagomerana et al., 2018 | CH | Malawi | Postpartum women | 252 | 84.1 | 9 |
| Chagomerana et al., 2018 | CH | Malawi | Pregnant women | 864 | 92.4 | 9 |
| Chagomerana et al., 2021 | CH | Malawi | Pregnant women | 819 | 86.5 | 9 |
| Chagomerana et al., 2022 | CH | Malawi | Postpartum women | 102 | 88.9 | 9 |
| [Chetty et al., 2017](https://onlinelibrary.wiley.com/authored-by/Chetty/Terusha) | CH | South Africa | Pregnant and postpartum | 3941 | 83.2 | 8 |
| [Chetty et al., 2018](https://onlinelibrary.wiley.com/authored-by/Chetty/Terusha) | CH | South Africa | Pregnant women | 150 | 77.3 | 8 |
| [Chetty et al., 2019](https://onlinelibrary.wiley.com/authored-by/Chetty/Terusha) | CH | South Africa | Postpartum women | 150 | 80.7 | 8 |
| Demissie et al., 2020 | CS | Ethiopia | Pregnant women | 319 | 89.7 | 9 |
| Duri et al., 2020 | CH | Zimbabwe | Pregnant women | 608 | 78.1 | 8 |
| Gabagaya et al., 2021 | CH | Uganda | Pregnant women | 532 | 29.1 | 9 |
| Hailu et al., 2025 | CS | Ethiopia | Pregnant women | 7764 | 96.8 | 9 |
| Kafack et al., 2022 | CH | Cameroon | Pregnant women | 135 | 89.7 | 8 |
| Koss et al., 2017 | CH | Uganda | Pregnant women | 150 | 80 | 9 |
| Landes et al., 2019 | CS | Malawi | Postpartum women | 1124 | 87.9 | 8 |
| Landes et al., 2021 | CH | Malawi | Postpartum women | 773 | 87.4 | 8 |
| Langwenya et al., 2018 | CH | South Africa | Pregnant and postpartum | 428 | 77 | 7 |
| M. Flynn et al., 2021 | CH | SSA | Postpartum women | 1220 | 75 | 8 |
| M. Gill et al., 2016 | CH | Rwanda | Pregnant women | 603 | 84.6 | 8 |
| M. Humphrey et al., 2022 | CH | Kenya | Pregnant women | 167 | 88 | 8 |
| M. Humphrey et al., 2023 | CH | Kenya | Pregnant women | 689 | 93 | 8 |
| Mazuguni et al., 2021 | CS | Tanzania | Pregnant women | 148 | 77 | 9 |
| Moyo et al., 2020 | CS | South Africa | Pregnant women | 2769 | 77.6 | 8 |
| Moyo et al., 2020 | CH | South Africa | Pregnant women | 40660 | 79.2 | 8 |
| Moyo et al., 2020 | CH | South Africa | Intrapartum women | 40660 | 85.7 | 8 |
| Munyaneza et al., 2025 |  | Rwanda | Both women | 530 | 91.9 | 8 |
| Musanhu et al., 2022 |  | Zimbabwe | Pregnant women | 121 | 87.7 | 8 |
| Myer et al., 2015 | CS | South Africa | Pregnant women | 574 | 87 | 9 |
| Myer et al., 2017 | CH | South Africa | Pregnant women | 523 | 78 | 8 |
| Myer et al., 2017 | CS | South Africa | Pregnant women | 620 | 91 | 8 |
| N. Atanga et al., 2018 | CH | Cameroon | Pregnant women | 268 | 92.7 | 9 |
| Napyo et al., 2020 | CS | Uganda | Pregnant women | 420 | 91.9 | 9 |
| Ndlangamandla et al., 2023 | CS | Estiwani | Pregnant women | 195 | 81.8 | 7 |
| Ngandu et al., 2022 | CS | South Africa | Pregnant and postpartum | 667 | 85.3 | 9 |
| Ntombela et al., 2022 | CS | South Africa | Pregnant women | 546 | 90.8 | 7 |
| Phillips et al., 2017 | CS | South Africa | Pregnant and postpartum | 452 | 92 | 9 |
| Sandbulte et al., 2020 | CH | Kenya | Pregnant women | 115 | 71 | 8 |
| Schrubbe et al., 2021 | CS | SSA | Pregnant and postpartum | 1685 | 63.8 | 8 |
| Schrubbe et al., 2022 | CS | SSA | Pregnant women | 560 | 59.9 | 8 |
| Schrubbe et al., 2023 | CS | SSA | Postpartum women | 1129 | 66 | 8 |
| Tsondai et al., 2016 | CH | South Africa | Pregnant women | 520 | 88 | 7 |
| Weldesenbet et al., 2020 | CS | South Africa | Pregnant women | 10052 | 73 | 9 |
| Woldesemayat et al., 2024 | CS | Ethiopia | Pregnant women | 496 | 97.8 | 9 |
| Woldesenbet et al., 2022 | CS | South Africa | Pregnant women | 8087 | 69.4 | 9 |
| Woldesenbet et al., 2022 | CS | South Africa | Pregnant women | 9733 | 73.3 | 9 |
| Yotebieng et al., 2019 | CS | DR Congo | Pregnant women | 1623 | 62 | 9 |
| Yotebieng et al., 2020 | CS | DR Congo | Intrapartum women | 1623 | 57 | 9 |
| Yotebieng et al., 2020 | CS | DR Congo | Postpartum women | 1623 | 65 | 9 |

Key: CS: Cross-sectional studies, CH: cohort studies, DR: Democratic Republic of Congo, SSA: Sub-Saharan Africa
