# Supplementary material for: Virologic suppression among HIV-positive pregnant and lactating women receiving antiretroviral therapy in Africa: A systematic review and meta-analysis
Source: PLoS One. 2026 Apr 13;21(4):e0346045. doi: 10.1371/journal.pone.0346045 (PMC13075719; doi:10.1371/journal.pone.0346045)
Supplement: S5 Table — (DOCX) [file pone.0346045.s005.docx]

| Authors | Factors associated with Virologic suppression (VL ≤1,000) | Authors | Factors associated with virologic failure (VL >1000) |
| --- | --- | --- | --- |
| Adeniyi et al., 2021 | - Maternal age 15-24 years (AOR = 0.68; 0.48, 0.94) - Starting ART in 3^rd^ trimester (AOR = 0.53 (0.30, 0.96) - Unemployed (AOR = 0.54; 0.32, 0.90) - Duration on ART; 27–40 week (AOR = 0.53; 0.30, 0.96) | Woldesenbet et al., 2020 | - Booking for ANC during the third trimester (AOR = 1.8; 1.4, 2.3) - Fewer than 2 ANC visits (AOR = 2.0; 1.7, 2.4) - Delayed initiation of ART in the second trimester (AOR = 2.2; 1.8, 2.7) - Younger age of 15–24 years (AOR = 1.4; 1.2, 1.8) |
| Woldesenbet et al., 2022 | - Younger age (15-24 years) (AOR = 0.7; 0.6, 0.8) - Unmarried (AOR=1.2; 1.04, 1.41) - Initiation of ART during pregnancy (AOR = 0.5; 0.4, 0.6) - Secondary education (AOR=1.1; 0.9, 1.4) - Tertiary education (AOR=1.3; 1.0, 1.6) | Schrubbe et al., 2022 | - Adolescence (AOR = 4.85; 2.58, 9.14) - 20-24 years AOR = 2.5; 1.63, 3.83) - Non-disclosure of HIV status to partner (AOR = 1.48; 1.02, 2.14) - Lack of paid employment (AOR=0.67; 0.47, 0.94) |
| Schrubbe et al., 2023 | - Younger age (15–24 years) (AOR = 0.21; 0.11, 0.39) - Disclosing HIV status (AOR = 1.56; 1.02, 2.38) | Ngandu et al., 2022 | - Being on first line ART (AOR = 2.3; 1.1, 4.6) - Age 15-24 years (AOR = 2.6; 1.1, 6.4) - Married/cohabiting (AOR: 1.9; 1.0, 3.7) |
| Kabami et al., 2024 | - Anticipated stigma - Challenges with non-disclosure of HIV status - Pregnancy distress   Distance to the health facility | Alamneh et al., 2023 | - WHO Stage III (AOR = 3.4; 1.045, 11.048) - WHO Stage IV (AOR = 6.0; 1.317, 27.512) - Poor or fair ART adherence (AOR = 5.1; 1.667, 15.719) - Test reason: Suspected viral load (AOR = 6.7; 3.147,14.09) |
| Hailu et al., 2025 | - Age 19–29 years (AOR = 3.17; 1.17, 5.17) - Good ART adherence (AOR = 2.33; 1.1, 5.0) | Chohan et al., 2021 | - Depression symptoms - Adherence behavior skills - Disclosure of HIV status - Food insecurity |
| Koss et al., 2017 | - Disclosing of HIV status (AOR = 4.51; 1.02, 19.8) | Myer et al., 2017 | - Age (15-22 years) (IRR=3.67; 1.78, 7.56) - Age (23-25 years) (IRR = 2.66; 1.31, 5.41) |
| Moyo et al., 2021 | - Age 25-35 years (AOR = -0.08, -0.09, -0.06) - Age ≥35 years (AOR = -0.10; -0.12, -0.07) - Baseline VL during pregnancy: 50–<1000 (AOR = 1.19; 1.16, 1.20); ≥1000 (AOR=2.31 2.30, 2.32) - CD4 count at ANC visit ≥500 (AOR = -0.28; -0.34, -0.2) - Presence of comorbidities (AOR = 0.12; 0.09, 0.14) - Not being on ART (AOR = -0.18; -0.19, -0.16) | Woldesenbet et al., 2024 | - Family size (X^2^=7.20) - Poor and fair level of Adherence (X^2^=18.553) - Exposure to opportunistic infection (X^2^=25.29) - WHO clinical stages II & III (X^2^=25.29) - HIV status non-disclosure (X^2^=4.408) - CD4 count <350 cells/ul (X^2^ = 15.989) |
| Yotebieng et al., 2019 | - Urban residency (APR=1.24; 1.00,1.54) - Time of ART initiation: 12–24 months (APR = 1.19; 1.03,1.37), >24 months (APR=1.14; 1.02, 1.27) - 25–34 years (APR=1.11; 0.96,1.28) - Age ≥ 35 years (APR = 1.21; 1.05, 1.39) - Disclosing of HIV status (APR = 1.15; 1.07, 1.25) - Married/cohabitating (APR = 1.09; 1.00, 1.19) | Woldesenbet et al., 2022 | - Unintended pregnancy (RR = 1.3; 1.1, 1.4) - No or primary (RR = 1.5; 1.2, 1.9) - Secondary education (RR =1.2, 1.0, 1.5) - Co-habiting (RR = 1.3; 1.1, 1.6) - Non-cohabiting relationship (RR = 1.3; 1.1, 1.6) - Rural dweller (RR = 1.3; 1.2, 1.5) - Initiating ART at third trimester of pregnancy (RR = 1.3; 1.1, 1.4) |
| Gabagaya et al., 2021 | - Non-disclosure of HIV status (AOR = 0.64; 0.42, 0.98) | Landes et al., 2019 | - Poor adherence of ART >= 2 doses/ month AOR = 3.1(2.0to4.9) |
| Alamneh et al., 2023 | - WHO clinical stage 2 (AOR = 1.64; 1.36, 2.036) - Fair or poor adherence to ART: (AOR = 0.505, 0.13, 0.721) - Delayed ART initiation (AOR = 0.14; 0.002, 0.035) - Missing drug doses (AOR = 0.38; 0.04, 0.280) | Ndlangamandla et al., 2023 | - Aged 17 - 24 years old (AOR=4.7; 1.8, 11.8) - Not on ARV use during pregnancy (AOR=8.1; 3.0, 21.8) - Multiple sexual partners in the last 12 months (AOR=7.9; 1.6, 40.5) |
| Boisson-Walsh et al., 2024 | - Urban residency (AOR = 1.54; 1.10, 2.15) - External supervision <=6 months (AOR =1.30; 1.03, 2.31) | Concepcion et al., 2023 | - Depressive symptoms (AOR = 2.2: 1.2, 4.0) |
| Nsubuga‑Nyombi et al., 2021 | - Long distances to health facility - Missed appointments - Running out of pills - Sharing antiretroviral drugs - Partner violence - Multiple sexual partners - Non‑disclosure to partners - Inadequate counseling - Stigma | Atanga et al., 2018 | Poor ART adherence (AOR= 7.6; 1.8, 30.8) |
| Ntombela et al., 2022 | - Being married (PRR = 1.11; 1.05, 1.18) |  |  |
| Musanhu et al., 2022 | - Government facilities - Barriers of VL testing: - Staff shortages - Non-availability of consumables - Sub-optimal sample transportation |  |  |
| M. Gill et al., 2016 | - Secondary and above education (AOR = 2.27; 1.37, 3.7) - Good adherence to ART (AOR = 1.32; 0.72, 2.44) - Disclosing HIV status (AOR= 1.96; 1.21, 3.23) |  |  |
| Landes et al., 2019 | - Primary education (AOR=0.3; 0.2, 0.7) - Secondary education or above (AOR =0.3; 0.1, 0.6) |  |  |

*APR: adjusted prevalence ratio, RR = Relative risk
