# Supplementary material for: Virologic suppression among HIV-positive pregnant and lactating women receiving antiretroviral therapy in Africa: A systematic review and meta-analysis
Source: PLoS One. 2026 Apr 13;21(4):e0346045. doi: 10.1371/journal.pone.0346045 (PMC13075719; doi:10.1371/journal.pone.0346045)
Supplement: Attachment — Submitted filename: PLOS_ONE(Review_Paper).pdf [file pone.0346045.s006.pdf]

# Review Paper

# Virologic suppression among HIV-positive pregnant and lactating women receiving antiretroviral therapy in Africa: A systematic review and meta-analysis

## Introduction:

This manuscript presents a systematic review and meta-analysis assessing the pooled prevalence of virologic suppression (VS) among HIV-positive pregnant and lactating women receiving antiretroviral therapy (ART) in Africa. The authors included 55 studies encompassing 304,883 participants across various African regions. The study followed PRISMA guidelines and used robust statistical methods, including a random-effects model, subgroup analyses, and heterogeneity assessment.

The topic is highly relevant, particularly in the context of achieving the global UNAIDS 95–95–95 targets and improving maternal and child health outcomes in sub-Saharan Africa. The manuscript contributes meaningful evidence regarding ART adherence and determinants of viral suppression in this key population group. This manuscript has major strengths such as relevance, scope and data size, systematic approach, robust analysis, policy value.

## Minor Editorial Comments:

- ✓ In Abstract section , replace "remains low" with quantitative context ("approximately 81%, below global target of 95%"). Conduct meta-regression and sensitivity analysis by study design, ART regimen, and publication year.
- ✓ Provide more discussion on possible small-study or regional publication bias.
- ✓ Include qualitative evidence or secondary data review to contextualize findings.
- ✓ Ensure all forest plots (Figures 2–8) have consistent formatting and readable axis labels.
- ✓ For References, several citations lack full journal details or DOI (e.g., refs. 70, 77). Please verify all references conform to PLOS ONE style. Conduct sensitivity analysis by publication period (pre/post-2020.
- ✓ Minor edits needed for flow in Introduction and Discussion to grammar.

## Closing Statement:

This manuscript is a well-executed, timely, and policy-relevant meta-analysis that adds significant value to the understanding of virologic suppression among HIV-positive women in Africa.

With minor clarifications on methodology, expanded heterogeneity analysis, and slight editorial adjustments, the paper will meet PLOS ONE’s publication standards and provide actionable insights for maternal HIV prevention and control programs across the continent.
