# Supplementary material for: Virologic suppression among HIV-positive pregnant and lactating women receiving antiretroviral therapy in Africa: A systematic review and meta-analysis
Source: PLoS One. 2026 Apr 13;21(4):e0346045. doi: 10.1371/journal.pone.0346045 (PMC13075719; doi:10.1371/journal.pone.0346045)
Supplement: Attachment — Submitted filename: PONE-D-25-49195_reviewer JB.pdf [file pone.0346045.s008.pdf]

PLOS ONE

Virologic suppression among HIV-positive pregnant and lactating women receiving antiretroviral therapy in Africa: A systematic review and meta-analysis

--Manuscript Draft--

**Manuscript Number:**	PONE-D-25-49195
**Article Type:**	Research Article
**Full Title:**	Virologic suppression among HIV-positive pregnant and lactating women receiving antiretroviral therapy in Africa: A systematic review and meta-analysis
**Short Title:**	Virologic suppression among HIV-positive women in Africa: systematic review and meta-analysis
**Corresponding Author:**	Berihun Agegn Mengistie<br>University of Gondar College of Medicine and Health Sciences<br>Gondar, ETHIOPIA
**Keywords:**	Africa; Antiretroviral therapy; HIV; pregnant women; lactating women; virologic suppression
**Abstract:**	Background: The elimination of mother-to-child transmission of human immunodeficiency virus (HIV) is a key global public health priority. Achieving viral load suppression is essential for preventing vertical transmission of HIV and reducing associated maternal morbidity and mortality. While many African countries have adopted the global 95-95-95 targets, comprehensive data on virologic suppression among pregnant and lactating mothers across the continent remains limited. The objective of this systematic review and meta-analysis was to determine the pooled estimate of virologic suppression and to examine the factors associated with it among HIV-positive pregnant and lactating women on antiretroviral therapy in Africa.<br>Methods: This study followed the Preferred Reporting Items for Systematic Reviews and Meta-Analyses (PRISMA) guidelines. The study protocol was registered in the International Prospective Register of Systematic Reviews (PROSPERO; CRD420251077063). We carried out a thorough systematic review by examining PubMed, ScienceDirect, Hinari, and Google Scholar for relevant studies. Data from the studies were retrieved using an Excel sheet and analyzed with STATA version 17. The Joanna Briggs Institute appraisal tool was used to evaluate the methodological quality of studies. A random-effects model (Der Simonian–Laird method) was applied to calculate the pooled prevalence of virologic suppression in Africa. A funnel plot and the Egger's test were used to investigate publication bias. Statistical heterogeneity was assessed using the I^2 statistic and Cochrane's Q test.<br>Results: A total of 55 eligible studies conducted in Africa, comprising 304,883 participants, were included in the quantitative meta-analysis. Accordingly, the overall prevalence of virologic suppression among HIV-positive pregnant and breastfeeding women in Africa was 80.92% (95% CI: 78.92%-82.91%). Virological suppression was significantly associated with women's age (15-24 years) (AOR = 0.49; 95% CI: 0.32-0.77), disclosure of HIV status to a partner (AOR = 1.66; 95% CI: 1.31-2.11), first-line antiretroviral therapy regimen (AOR = 6.53; 95% CI: 1.93-22.06), and good antiretroviral drug adherence (AOR = 3.61; 95% CI: 1.18-11.02).<br>Conclusion: The overall pooled estimate of virologic suppression among HIV-positive pregnant and breastfeeding women in Africa remains low compared with the global target for viral suppression. This emphasizes the necessity of targeted strategies for younger HIV-positive women, disclosing HIV status, initiating first-line antiretroviral regimens, and promoting antiretroviral treatment adherence. Upgrading health care systems to enable regular viral load monitoring, as well as addressing socio-demographic and antiretroviral therapy-related variables, are vital steps towards attaining reduced viral load and improved treatment outcomes.
**Order of Authors:**	Berihun Agegn Mengistie
	Getie Mihret Aragaw
	Gebrye Gizaw Mulatu
	Kindu Yinges Wondie

Powered by Editorial Manager® and ProduXion Manager® from Aries Systems Corporation

	Alemneh Tadesse Kassie
	Alemken Eyayu Abuhay
	Wondimnew Mersha Biset
	Moges Tesfa Tsega
	Abay Eyayu Asrie
	Tazeb Alemu Anteneh
	Habtu Kifle Negash
	Eshet Gebrie
	Nuhamin Tesfa Tsega
**Additional Information:**	
**Question**	**Response**
**Financial Disclosure**<br><br>Enter a financial disclosure statement that describes the sources of funding for the work included in this submission. Review the submission guidelines for detailed requirements. View published research articles from *PLOS ONE* for specific examples.<br><br>This statement is required for submission and **will appear in the published article** if the submission is accepted. Please make sure it is accurate.<br><br>**Funded studies**<br>Enter a statement with the following details:<br>• Initials of the authors who received each award<br>• Grant numbers awarded to each author<br>• The full name of each funder<br>• URL of each funder website<br>• Did the sponsors or funders play any role in the study design, data collection and analysis, decision to publish, or preparation of the manuscript?<br><br>Did you receive funding for this work?	The author(s) received no specific funding for this work.
**Competing Interests**<br><br>Use the instructions below to enter a competing interest statement for this submission. On behalf of all authors, disclose any competing interests that	The authors have declared that no competing interests exist.

could be perceived to bias this work—acknowledging all financial support and any other relevant financial or non-financial competing interests.<br><br>This statement is required for submission and **will appear in the published article** if the submission is accepted. Please make sure it is accurate and that any funding sources listed in your Funding Information later in the submission form are also declared in your Financial Disclosure statement.<br><br>View published research articles from *PLOS ONE* for specific examples.<br><br>**NO authors have competing interests**<br><br>Enter: *The authors have declared that no competing interests exist.*<br><br>**Authors with competing interests**<br><br>Enter competing interest details beginning with this statement:<br><br>*I have read the journal's policy and the authors of this manuscript have the following competing interests: [insert competing interests here]*<br><br>* typeset	
**Ethics Statement**<br><br>Enter an ethics statement for this submission. This statement is required if the study involved:<br><br>• Human participants<br>• Human specimens or tissue<br>• Vertebrate animals or cephalopods<br>• Vertebrate embryos or tissues<br>• Field research<br><br>Write "N/A" if the submission does not require an ethics statement.	N/A

Powered by Editorial Manager® and ProduXion Manager® from Aries Systems Corporation

General guidance is provided below. Consult the submission guidelines for detailed instructions. **Make sure that all information entered here is included in the Methods section of the manuscript.**<br><br>**Format for specific study types**<br><br>**Human Subject Research (involving human participants and/or tissue)**<br>• Give the name of the institutional review board or ethics committee that approved the study<br>• Include the approval number and/or a statement indicating approval of this research<br>• Indicate the form of consent obtained (written/oral) or the reason that consent was not obtained (e.g. the data were analyzed anonymously)<br><br>**Animal Research (involving vertebrate animals, embryos or tissues)**<br>• Provide the name of the Institutional Animal Care and Use Committee (IACUC) or other relevant ethics board that reviewed the study protocol, and indicate whether they approved this research or granted a formal waiver of ethical approval<br>• Include an approval number if one was obtained<br>• If the study involved *non-human primates*, add *additional details* about animal welfare and steps taken to ameliorate suffering<br>• If anesthesia, euthanasia, or any kind of animal sacrifice is part of the study, include briefly which substances and/or methods were applied<br><br>**Field Research**<br><br>Include the following details if this study involves the collection of plant, animal, or other materials from a natural setting:<br>• Field permit number<br>• Name of the institution or relevant body that granted permission	
**Data Availability**<br><br>Authors are required to make all data	Yes - all data are fully available without restriction

Powered by Editorial Manager® and ProduXion Manager® from Aries Systems Corporation

underlying the findings described fully available, without restriction, and from the time of publication. PLOS allows rare exceptions to address legal and ethical concerns. See the PLOS Data Policy and FAQ for detailed information.<br><br>A Data Availability Statement describing where the data can be found is required at submission. Your answers to this question constitute the Data Availability Statement and **will be published in the article**, if accepted.<br><br>**Important:** Stating 'data available on request from the author' is not sufficient. If your data are only available upon request, select 'No' for the first question and explain your exceptional situation in the text box.<br><br>Do the authors confirm that all data underlying the findings described in their manuscript are fully available without restriction?	
**Describe where the data may be found in full sentences. If you are copying our sample text, replace any instances of XXX with the appropriate details.**<br><br>• If the data are **held or will be held in a public repository**, include URLs, accession numbers or DOIs. If this information will only be available after acceptance, indicate this by ticking the box below. For example: *All XXX files are available from the XXX database (accession number(s) XXX, XXX.*).<br>• If the data are all contained **within the manuscript and/or Supporting Information files**, enter the following: *All relevant data are within the manuscript and its Supporting Information files.*<br>• If neither of these applies but you are able to provide **details of access elsewhere**, with or without limitations, please do so. For example:<br><br>*Data cannot be shared publicly because of [XXX]. Data are available from the*	All relevant data are within the manuscript and its Supporting Information files.

Powered by Editorial Manager® and ProduXion Manager® from Aries Systems Corporation

XXX Institutional Data Access / Ethics Committee (contact via XXX) for researchers who meet the criteria for access to confidential data.<br><br>*The data underlying the results presented in the study are available from (include the name of the third party and contact information or URL).*<br>• This text is appropriate if the data are owned by a third party and authors do not have permission to share the data.<br><br>* typeset	
Additional data availability information:	

Powered by Editorial Manager® and ProduXion Manager® from Aries Systems Corporation

Manuscript Click here to access/download;Manuscript;Manuscript.docx

Virologic suppression among HIV-positive pregnant and lactating women receiving antiretroviral therapy in Africa: A systematic review and meta-analysis

Berihun Agegn Mengistie (BAM)[1*], Getie Mihret Aragaw (GMA)[1], Gebrye Gizaw Mulatu (GGM)[2], Kindu Yinges Wondie (KYW)[3], Alemneh Tadesse Kassie (ATK)[3], Alemken Eyayu Abuhay (AEA)[4], Wondimnew Mersha Biset (WMB)[5], Moges Tesfa Tsega (MTT)[6], Abay Eyayu Asrie (AEA)[7], Tazeb Alemu Anteneh (TAA)[3], Habtu Kifle Negash (HKN)[8], Eshet Gebrie (EG)[9], Nuhamin Tesfa Tsega (NTT)[10]

[1]Department of General Midwifery, School of Midwifery, College of Medicine and Health Sciences, University of Gondar, Gondar, Ethiopia, [2]Department of Health Informatics, College of Medicine and Health Sciences, University of Gondar, Gondar, Ethiopia, [3]Department of Clinical Midwifery, School of Midwifery, College on Medicine and Health Sciences, University of Gondar, Gondar, Ethiopia, [4]University of Gondar Comprehensive Specialized Hospital, Gondar, Ethiopia, [5]Department of Anesthesiology, School of Medicine, Saint Paul's Hospital Millennium Medical College, Addis Ababa, Ethiopia, [6]Department of Internal Medicine, School of Medicine, College of Medicine and Health Sciences, University of Gondar, Gondar, Ethiopia, [7]Department of Obstetrics and Gynecology, College of Medicine and Health Sciences, University of Gondar, Gondar, Ethiopia, [8]Department of Human Anatomy, School of Medicine, College of Medicine and Health Sciences, University of Gondar, Gondar, Ethiopia, [9]Department of Clinical Chemistry, School of Biomedical and Laboratory Sciences, College of Medicine and Health Sciences, University of Gondar, P.O. Box 196, Gondar, Ethiopia, [10]Department of Women's and Family Health, School of Midwifery, College on Medicine and Health Sciences, University of Gondar, Gondar, Ethiopia

Authors' addresses

BAM: berihunagegn21@gmail.com

GMA: getie895@gmail.com

GGM: gebrye.gizaw@gmail.com

KYW: Kinduyinges2010@gmail.com

ATK: alexotadesse.79@gmail.com

AEA: alexeyayu6@gmail.com

WMB: Wondemer2096@gmail.com

MTT: mogestesfa46@gmail.com

AEA: eyayuabay3@gmail.com

TAA: tazebalemu05@gmail.com

HKN: habtuk8@gmail.com

EG: eshetgebrie@gmail.com

NTT: nuha26tesfa@gmail.com

BAM*: berihunagegn21@gmail.com (Corresponding author)

Abstract

Background: The elimination of mother-to-child transmission of human immunodeficiency virus (HIV) is a key global public health priority. Achieving viral load suppression is essential for preventing vertical transmission of HIV and reducing associated maternal morbidity and mortality. While many African countries have adopted the global 95-95-95 targets, comprehensive data on virologic suppression among pregnant and lactating mothers across the continent remains limited. The objective of this systematic review and meta-analysis was to determine the pooled estimate of virologic suppression and to examine the factors associated with it among HIV-positive pregnant and lactating women on antiretroviral therapy in Africa.

Methods: This study followed the Preferred Reporting Items for Systematic Reviews and Meta-Analyses (PRISMA) guidelines. The study protocol was registered in the International Prospective Register of Systematic Reviews (PROSPERO; CRD420251077063). We carried out a thorough systematic review by examining PubMed, ScienceDirect, Hinari, and Google Scholar for relevant studies. Data from the studies were retrieved using an Excel sheet and analyzed with STATA version 17. The Joanna Briggs Institute appraisal tool was used to evaluate the methodological quality of studies. A random-effects model (Der Simonian–Laird method) was applied to calculate the pooled prevalence of virologic suppression in Africa. A funnel plot and the Egger's test were used to investigate publication bias. Statistical heterogeneity was assessed using the I^2 statistic and Cochrane's Q test.

Results: A total of 55 eligible studies conducted in Africa, comprising 304,883 participants, were included in the quantitative meta-analysis. Accordingly, the overall prevalence of virologic suppression among HIV-positive pregnant and breastfeeding women in Africa was 80.92% (95%

CI: 78.92%-82.91%). Virological suppression was significantly associated with women's age (15-24 years) (AOR = 0.49; 95% CI: 0.32-0.77), disclosure of HIV status to a partner (AOR = 1.66; 95% CI: 1.31-2.11), first-line antiretroviral therapy regimen (AOR = 6.53; 95% CI: 1.93-22.06), and good antiretroviral drug adherence (AOR = 3.61; 95% CI: 1.18-11.02).

Conclusion: The overall pooled estimate of virologic suppression among HIV-positive pregnant and breastfeeding women in Africa remains low compared with the global target for viral suppression. This emphasizes the necessity of targeted strategies for younger HIV-positive women, disclosing HIV status, initiating first-line antiretroviral regimens, and promoting antiretroviral treatment adherence. Upgrading health care systems to enable regular viral load monitoring, as well as addressing socio-demographic and antiretroviral therapy-related variables, are vital steps towards attaining reduced viral load and improved treatment outcomes.

Keywords: Africa; Antiretroviral therapy; HIV; pregnant women; lactating women; virologic suppression

Introduction

Human immunodeficiency virus (HIV) is one of the world's most critical public health challenges, affecting millions of people. In 2024, 39.9 million people worldwide were infected with HIV, 1.3 million new HIV cases were reported, and 630,000 people died from AIDS-related illnesses [1, 2]. Sub-Saharan African countries were responsible for 65% of the worldwide HIV burden. Women and girls of all ages contributed to 44% of all new HIV infections globally in 2023 [1]. According to the Joint United Nations Programme on HIV/AIDS (UNAIDS) 2024 report, nearly 120,000 children aged 0-14 were infected with HIV for the first time [1].

The primary route of HIV infection in children is mother-to-child transmission (MTCT), also referred to as vertical transmission [3]. It accounts for more than 90% of all newly acquired pediatric HIV infections worldwide [4]. Without intervention, the overall MTCT incidence ranges from 15% to 45% [5]. However, appropriate interventions during pregnancy, labor, and breastfeeding can lower the risk of perinatal HIV transmission to less than 2% in non-breastfeeding and 5% in breastfeeding populations, respectively [6, 7].

Maternal plasma HIV viral load (VL) is the strongest determinant of HIV transmission from mother to child (MTCT) [8-10]. HIV viral load assays fall into three main categories: undetectable (≤50 copies/ml), low-level viremia (50-1000 copies/ml), and high VL (>1000 copies/ml) [8, 11]. Pregnant and lactating women who do not achieve virologic suppression (VS) are at a higher risk of HIV-related complications, death, and vertical transmission to their infants [8, 12].

Maintaining viral load suppression (≤1000 copies/ml) in HIV-positive pregnant and postpartum women is a global public health goal for improving maternal health and producing HIV-free children [10, 13]. Viral load suppression (VLS) is an important indicator of antiretroviral therapy's (ART) effectiveness. PMTCT programs are implemented by a number of interventions, such as

lifetime ART for pregnant and breastfeeding women, preventive antiretroviral (ARV) medications for HIV-exposed infants (HEIs), serial testing for HEIs, and prompt ART initiation for children who acquire HIV [3, 14].

Antiretroviral therapy (ART) as part of PMTCT is an important strategy to combat the global AIDS crisis and to lower vertical transmission rates [15]. The WHO guidelines indicated that ART should be initiated in all pregnant and breastfeeding HIV-positive women, regardless of their VL, clinical stage, or CD4 cell count [12]. The fixed-dose combination (FDC) of tenofovir disoproxil fumarate (TDF), lamivudine (3TC), and dolutegravir (DTG) is the ideal first-line regimen for the treatment of HIV infection in adults and adolescents, including pregnant and breastfeeding women [16]. Timely initiation and consistent adherence to ART are key predictors for preventing mother-to-child transmission of HIV, mitigating a range of clinical, virological, and immunological risks [12, 17].

Since 2016, viral load measurement has been the major approach for monitoring HIV patients' clinical response to ART every 3–6 months [18]. Factors associated with virologic non-suppression include suboptimal adherence, medication intolerance, drug–diet interactions, low CD4 cell counts, prior treatment history, and antiretroviral drug resistance [19].

To tackle the continuing HIV crisis, WHO, the Global Fund, and UNAIDS have established global HIV plans that support the achievement of SDG target 3.3, aiming to end the HIV epidemic by 2030 [2]. The UNAIDS program is now working toward a 95-95-95 global target, aiming at eliminating the global epidemic by 2030 [20, 21]. These universal aims include ensuring that 95% of individuals living with HIV are aware of their HIV status, 95% of those diagnosed obtain continuous ART medication, and at least 95% of patients beginning ART achieve viral suppression [21]. However, in many of the SSA countries, viral load suppression is still behind the UNAIDS

95-95-95 targets [22, 23]. Broadly, previous study findings on the proportion of viral load suppression among pregnant and breastfeeding women living with HIV in Africa revealed inconsistent and equivocal results that ranged from 29.1% to 97.8% [24, 25].

Women in most African countries frequently experience major challenges to accessing healthcare due to socioeconomic barriers, caregiver responsibilities, gender-based discrimination, and low economic freedom [26-28]. This could have been associated with a greater viral load in pregnant and nursing women. However, there is a lack of comprehensive study on virologic suppression among pregnant and breastfeeding women at the national and regional levels in Africa.

Thus, the goal of this systematic review and meta-analysis is to assess the pooled prevalence of virologic suppression and identify associated factors among HIV-positive pregnant and breastfeeding women receiving ART in Africa. This comprehensive data will assist health professionals, policymakers, and guideline developers in implementing evidence-based strategies to improve viral load suppression in these populations, hence reducing HIV perinatal transmission.

Research questions

1. What is the pooled estimate of virologic suppression among HIV-positive pregnant and lactating women receiving antiretroviral therapy in Africa?
2. What are the determinant factors of virologic suppression among HIV-positive pregnant and lactating women receiving antiretroviral therapy in Africa?

Methods and materials

Study protocol and search strategy

This systematic review adhered to the Preferred Reporting Items for Systematic Reviews and Meta-Analysis (PRISMA) standards [29] **(S1 file)**. The study protocol for the review has been registered on PROSPERO (ID CRD420251077063). A rigorous and comprehensive review of Google Scholar, PubMed, Hinari, and ScienceDirect for original research on virologic suppression and associated factors conducted in Africa. The inclusion and exclusion criteria were established based on the condition, context, and study population (Co Co Pop) approach. Previous studies that met the eligibility criteria were retrieved.

A search strategy was developed for the databases by combining keywords with Boolean operators. Ultimately, we employed the following search term combination: “viral load suppression”, “virologic suppression”, “viral suppression”, “HIV viral load non-suppression”, “viral load”, “detectable viral load”, “undetectable viral load”, “virological suppression”, “HIV viral load”, “plasma viremia”, “HIV viral suppression”, “HIV viremia”, “HIV-positive women”, pregnant, “lactating mothers”, “postpartum women”, “breastfeeding mothers”, "antiretroviral therapy", ART and Africa. To obtain additional articles from the citation list of publications found in the databases that were accessible, snowballing techniques were also employed **(S2 file)**

Eligibility criteria

Inclusion criteria

Condition: The condition of interest was virologic suppression among HIV-positive pregnant and lactating women receiving antiretroviral therapy in Africa.

Context: All primary studies that reported the prevalence and/or associated factors of virologic suppression in the Africa context.

Population: The study population for this review is pregnant and lactating mothers living with HIV on ART.

Study design: All primary observational studies, including cross-sectional, case-control, and cohort studies that reported the prevalence and/or associated factors virologic suppression among HIV-positive pregnant and lactating women receiving ART.

Publication year: Both published and unpublished articles between January 1, 2016, and April 30, 2025, were included.

Exclusion criteria

Articles that failed to report the outcome of interest, narrative reviews, qualitative reviews, expert comments, case reports, editorials, letters, and methodological studies were excluded from the review.

Measurement of outcome variables

This study aimed to determine the pooled prevalence of virologic suppression among African pregnant and lactating women receiving antiretroviral therapy. This study's second objective was to determine the variables that are associated with virologic suppression in Africa. These variables were assessed using adjusted odds ratios from previous studies. The World Health Organization (WHO) defined virological suppression as when viral load copies become ≤1000 copies/mL of blood after six months of ART initiation [30]. However, virologic failure (VF) is defined as a VL persistently over 1000 copies/mL in two consecutive measurements, 3 months apart, after 6 months on ART and adherence support [30].

Quality assessment for included studies

The quality of the included primary studies was evaluated independently by two authors (BAM and NTT). Each study was assessed using the Critical Appraisal Checklist developed by the Joanna Briggs Institute (JBI). Twenty-eight of the included papers were evaluated using the 9-item cross-sectional checklist, while 27 used the 11-item cohort checklist [31, 32]. Studies with a JBI checklist score of five or higher were deemed to be of excellent quality or to have minimal likelihood of bias. Any disputes between the two authors were settled by open conversation and consultation with GMA, the third author **(Table 1).**

Data extraction and management

Data extraction from the included publications was performed independently by two authors (BAM and NTT) using a standardized abstraction form developed in Excel. In accordance with the inclusion and exclusion criteria, all identified studies were imported into the reference manager EndNote 20. After screening titles and abstracts, full texts were reviewed for eligibility. Disagreements were resolved through discussion with a third reviewer (GMA). To avoid duplication, studies with overlapping or identical data were excluded. For eligible studies, the following information was extracted: identification details (first author's last name and year of publication), proportion of virologic suppression, factors associated with virologic suppression, adjusted odds ratios with 95% confidence intervals, study area, study design, study population, sample size, and risk of bias assessment method **(S3 file, Table 1)**.

Data synthesis and statistical analysis

All statistical data analyses were carried out after the data were collected using a Microsoft Excel spreadsheet and exported to the statistical program STATA version 17. Texts, tables, and forest plots were used to display the extracted data. A binomial distribution was used to determine each study's standard error of prevalence of the outcome variable. The Higgins I-squared (I^2) test was

used to check for heterogeneity in the pooled prevalence of the studies. The heterogeneity among the included studies was classified as low, moderate, or high based on I^2 values of <25%, 50–75%, and >75%, respectively [33].

A random-effects model (Der Simonian and Laird's method) was used to determine the pooled prevalence of virologic suppression in Africa. A subgroup analysis was conducted across the country and study population to identify potential sources of study heterogeneity. In addition, we performed a leave-one-out sensitivity analysis to look at how particular studies influence the pooled estimate. The pooled estimates from across the continent were then displayed in forest plots and tables, along with their corresponding 95% confidence intervals. Visually, publication bias has been assessed using a funnel plot (23). Statistically, potential publication bias was evaluated using Egger's and Begg's tests, with a p-value < 0.05 suggesting evidence of publication bias [34]. Finally, the pooled adjusted odds ratios (AORs) with 95% confidence intervals (CI) were presented in forest plots.

Results

Study selection and characteristics of the included studies

All searching databases, including Google Scholar, yielded a total of 1,639 research articles; 406 duplicate records were deleted, and the remaining 1,233 papers were further screened. However, the majority of papers (n = 1,108) were removed after reading their titles and abstracts. The remaining 125 full-text articles were then reviewed for eligibility criteria, and 70 studies were excluded for a variety of reasons, including insufficient data, a difference in the VLS cut-point, variation in the study context, being unrelated to the outcome of interest, systematic reviews, and qualitative reviews. Then, the remaining 55 eligible studies with a total of 304,883 HIV-positive pregnant and breastfeeding women were included in the final quantitative meta-analysis [35-77].

In terms of study distribution across Africa, 31 and 13 studies were undertaken in Southern Africa and Eastern Africa, respectively **(Fig. 1).**

Prevalence of virological suppression in Africa

In this meta-analysis, the pooled prevalence of virological suppression among pregnant and lactating women receiving ART in Africa was 80.92% (95% CI: 78.92%, 82.91%). A statistical test indicated the presence of significant heterogeneity among the included studies (I^2 = 99.57%, p-value = 0.000). As a result, a random-effects model, subgroup analyses, and sensitivity analyses were performed **(Fig. 2)**.

Heterogeneity and subgroup analysis

Subgroup analysis was performed based on the country, the regions of Africa, and the study population. Accordingly, Ethiopia had the highest prevalence of VS, 94.11% (95% CI: 91.16, 97.07), while the Democratic Republic of Congo had the lowest prevalence, 62.66% (95% CI: 58.56, 66.75). Moreover, the lowest overall prevalence of VS in pregnant and lactating mothers living with HIV was found in the Central Africa region (62.66%; 95% CI: 58.56, 66.75), whereas the highest was in Western and East Africa, which found nearly 84% of viral suppression. In terms of the study population, 81% of pregnant and postpartum mothers had VS, which is nearly the same pooled prevalence. Even though subgroup analysis was performed based on the previously listed parameters, no noticeable reduction in heterogeneity was observed in the pooled estimate of virological suppression **(Table 2)**.

Publication bias

The presence of publication bias was graphically checked using a funnel plot, and it was confirmed using Egger's test. The symmetrical appearance of the funnel plot in this analysis indicates that

there is no publication bias among the studies **(Fig. 3)**. Egger's (p-value = 0.0512) and Begg's (p-value = 0.5135) tests were statistically non-significant, suggesting that there was no substantial publication bias in the included studies.

Sensitivity analysis

To examine the impact of a single study on the estimated effect size, a leave-one-out sensitivity analysis was conducted using the random-effects model. The results, however, demonstrate that the pooled estimate was not substantially impacted by a single study, and the point estimate of the excluded study is within the confidence interval of the overall estimate of virologic suppression. This demonstrated that the average estimate of virologic suppression among HIV-positive pregnant and breastfeeding mothers in Africa was robust **(Fig. 4)**.

Meta-analysis of factors associated with virological suppression

This systematic review and meta-analysis investigated 11 studies that identified factors associated with virologic suppression in Africa [36, 37, 41, 52, 54, 58-60, 66, 68, 77]. Thereby, virologic suppression among HIV-positive pregnant and lactating women was substantially correlated with younger age (15–24 years), being unmarried, first-line ART use, HIV status disclosure to a partner, and good adherence to ART.

In this review, women aged 15–24 years (AOR = 0.49; 95% CI: 0.32–0.77) were 51% less likely to attain virologic suppression compared to those aged 25 years and older **(Fig. 5)**. Furthermore, a pooled analysis of three studies found that women who disclosed their HIV status (AOR = 1.66; 95% CI: 1.31, 2.11) were 1.66 times more likely to achieve virologic suppression compared to their counterparts **(Fig. 6)**. In addition, the likelihood of achieving viral load suppression was 6.5 times higher for pregnant and lactating women on a first-line ART regimen (AOR = 6.53; 95% CI: 1.93, 22.06) than for those on second- or third-line regimens **(Fig. 7)**. Finally, the odds of virologic

suppression among pregnant and lactating women who had good ART adherence (AOR = 3.61, 95% CI: 1.18, 11.02) were 3.61 times more likely as compared to those who had poor adherence to ART **(Fig. 8)**.

Discussion

The main objectives of this systematic review and meta-analysis were to determine the proportion of virologic suppression among pregnant and lactating women receiving ART and identifying factors associated with it. Thus, the pooled prevalence of virologic suppression among pregnant and lactating women taking ART in Africa was 80.92% (95% CI: 78.92%, 82.91%). This conclusion was comparable with a study conducted in East Africa, which found 80.6% viral load suppression among patients living with HIV [78]. However, the finding of this review was lower than studies reported 84% in Ethiopia [79], 85% in Sub-Saharan Africa [80], and a global target of achieving viral suppression in at least 95% of patients initiating ART [21]. In contrast, the findings of the current study were higher than a study conducted in Ethiopia (71%) [81].

This could be due to the difference in the study population, study setting, and availability and adherence to ART. Thus, the study population for the previous studies was adults living with HIV, whereas the current study involves pregnant and lactating women receiving ART in Africa. The possible explanation could relate to physiological and immunological changes during perinatal periods [82]. Pregnancy alters the immune system and pharmacokinetics of ART drugs, which reduce the effectiveness of ART and increase maternal viral load [82, 83]. Furthermore, delayed initiation or inadequate compliance to ART, perceived shame, and socioeconomic or health system constraints, such as limited regular VL surveillance, ART stock-outs, and a shortage of maternity professionals could be the possible reason for non-virological suppression [36, 77, 84].

Suboptimal viral suppression or virologic failure jeopardizes not only the health of women but also raises the risk of HIV MTCT during pregnancy, childbirth, and breastfeeding, posing a challenge to the global effort towards eliminating pediatric HIV infection [36, 85, 86]. The finding of this SRM highlights the need for targeted interventions, including better ART adherence support, integrated maternal-HIV care, and robust monitoring to close the gap and achieve the global target, ultimately lowering vertical transmission and improving maternal and child health outcomes.

According to the findings of this study, Africa has not yet achieved the global target of attaining virological suppression in 95% of patients who received ART. To increase the possibility of successful virological outcomes, a variety of factors and problems must be addressed. In this systematic review and meta-analysis, women's age category of 15-24 years, being unmarried, being on a first-line ART regimen, disclosure of HIV status to a partner, and good adherence to ART were significantly associated with virological suppression in Africa.

In this study, the odds of virologic suppression among young women, particularly those 15-24 years old, were decreased by 51% when compared with those aged 25 years and above. The finding of this study was consistent with other studies [84, 87-89]. Younger people receiving treatment for HIV might have difficulty with compliance due to factors including lifestyle demands such as education or a job, societal expectations, worries regarding stigma and disclosure, mental health challenges, and lacking health literacy [84]. Understanding and addressing the unique challenges faced by young women helps healthcare workers and decision-makers create specific treatments that boost viral suppression rates and enhance health for this at-risk age group. Enhance compliance support, introduce youth-friendly programs, and cater to the special requirements of youths and young adults, while guaranteeing accessibility, privacy, and a favorable environment.

Moreover, pregnant and breastfeeding women who disclosed their HIV status were significantly associated with achieving virological suppression compared to those who did not. This finding is in agreement with other studies that revealed disclosure of HIV status increases the likelihood of viral load suppression [79, 90, 91]. The disclosure of one's HIV status to family, friends, and intimate relationships can have major health consequences. Several studies show that individuals who disclosed their serostatus had better social assistance, stronger family and relationship solidarity, lower symptoms of depression and anxiety, improved physical health, psychological assistance, and financial support, and were better able to take and adhere to ART [92-94].

This study also found that patients on first-line ART regimens had higher rates of virologic suppression compared to those on second- or third-line regimens, consistent with findings from other studies [91, 95-97]. This could be explained as first-line ART drugs (TDF-3TC-DTG) having higher efficacy (sustained VL suppression), fewer adverse effects, and lower resistance when compared to second- and third-line ART regimens [16, 98]. Therefore, all HIV-positive pregnant and lactating women should initiate triple ART as early as possible, regardless of their CD4 count or WHO clinical stage. This leads to optimal suppression of viral load to undetectable levels, thereby preventing the vertical transmission of HIV. Nevertheless, the virus can never be totally eliminated from the human body, so an individual should continue to take the treatments even after the signs and symptoms have subsided and adhere to safer sexual practices.

Finally, good adherence to antiretroviral therapy was significantly associated with virologic suppression among pregnant and breastfeeding women, aligning with findings from previous studies [91, 95, 96]. The plausible explanation is due to the fact that good adherence to ART sustains maximal viral load suppression to undetectable levels, lowers drug resistance, and boosts the immune system (CD4 cell counts), which leads to substantially lowering the risk of mother-to-

child transmission of HIV and improving the overall health of individuals [98, 99]. However, compliance with ART remains a major health issue worldwide, particularly in low- and middle-income nations. Virologic and clinical outcomes are highly dependent on strong compliance to ART, while poor adherence to antiretroviral drugs has been found to be a significant predictor of virologic failure, drug resistance, progression of disease, admissions to hospitals, high death rate, and medical care expenses [100].

In general, the findings of this review have various implications for clinical practice, policy, and future research works in these population groups. Since lifelong first-line ART regimens and enhanced adherence to ART have been strongly linked to greater virologic suppression, healthcare practitioners should increase adherence counseling, particularly during prenatal and postpartum contacts. The lower suppression rates in younger women demonstrate the necessity for youth-specific strategies like youth-friendly clinics, psychosocial assistance, and sexual and reproductive health education incorporated into HIV treatment programs. Optimizing virologic suppression in pregnant and breastfeeding women on ART in Africa requires a comprehensive strategy that includes upgrading clinical practices, ensuring responsive policy, and ongoing monitoring and evaluation to address ongoing gaps and disparities. Further qualitative and quantitative studies is required to investigate different sociocultural, behavioral, psychological, and structural factors that lead to virologic non-suppression among HIV-positive pregnant and breastfeeding women in the continent.

Strengths and limitations of the study

To our knowledge, this would be the first meta-analysis to assess the overall estimate of virologic suppression and associated factors among HIV-positive pregnant and nursing women in Africa. We searched multiple databases to include both published and unpublished studies and followed a

meticulous screening approach to compile all eligible studies. Thus, the findings of this review provide important baseline evidence for health providers, policymakers, academicians, and global partners to design and implement evidence-based interventions to achieve the WHO 95-95-95 global target to eliminate vertical transmission of HIV.

Despite the fact that enough primary studies were used to estimate the overall viral load suppression, many countries and some African regions were not evenly represented. The presence of significant heterogeneity among the included studies might affect the generalizability of the finding. This might be due to variations in the study area and the methodological quality of the studies, such as study design, sampling technique, response rate, sample size, and study setting, that could alter the pooled estimate's generalizability. Additionally, due to the concentration of studies in some countries, such as South Africa, it could restrict the conclusion of the finding to the whole continent. We employed a random-effects model for analysis in order to overcome this significant variability. Additionally, we performed subgroup analyses and sensitivity analyses to identify potential causes of substantial heterogeneity.

Conclusions

According to this systematic review and meta-analysis, the overall prevalence of virologic suppression among pregnant and lactating women taking ART in Africa (80.92%) is lower than USAID's global target of viral load suppression (95%). Being younger (15-24 years), being on a first-line ART regimen, disclosing HIV status to a partner, and having good adherence to ART were significantly associated with virologic suppression. This highlights the importance of targeted interventions for young HIV-positive women, disclosing HIV status, initiating first-line antiretroviral therapy regimens, and promoting antiretroviral therapy adherence; strengthening healthcare systems that offer regular viral load monitoring; and addressing socio-demographic and

ART-related factors as vital steps toward improving treatment outcomes and reducing perinatal transmission of HIV.

List of abbreviations

ART: Antiretroviral therapy, AOR: Adjusted odds ratio, CI: Confidence Interval, CD4: Cluster of differentiation 4, HIV: Human Immunodeficiency Virus, MTCT: Mother-to-Child Transmission of HIV, SRM: Systematic review and meta-analysis, VL: Viral load, VS: Virologic Suppression, UNAIDS: Joint United Nations Programme on HIV/AIDS, WHO: World health organization,

Declarations

Ethical approval

Not applicable

Consent for publication

Not applicable

Availability of data and materials

All relevant data are available within the manuscript or supplementary materials.

Competing interests

The authors have declared that no competing interests exist.

Funding

None funding

Author contributions

Conceptualization: BAM, NTT, GGM

Data curation: NTT, BAM, TAA, KYW, AEA, HKN

Formal analysis: NTT, BAM, KYW, HKN, ATK, WMB, GMA

Methodology: BAM, NTT, GMA, AEA, GGM, EG, MTT

Software: BAM, ATK, WMB, GMA, NTT

Validation: NTT, BAM, KYW, GMA, AEA, EG

Visualization: GMA, NTT, AEA, GGM

Writing original draft: TAA, KYW, ATK, MTT, WMB, HKN, GMA, AEA, NTT

Writing – review & editing: NTT, BAM, TAA, EG, KYW, ATK, GGM, WMB, GMA

Acknowledgements

The authors of this study would like to express our heartfelt gratitude to all of the authors of the studies included in this systematic review and meta-analysis.

References

1. UNAIDS. Fact sheet 2024 - Latest global and regional HIV statistics on the status of the AIDS epidemic. 2024.

2. (WHO) WHO. HIV and AIDS 2024 [

3. Tilaye BA, Hailemeskel HS, Baye FD, Hailu MK, Mekonnen GB, Arage G, et al. Incidence of mother-to-child transmission of HIV and predictors of positivity among HIV exposed infants in South Gondar public hospitals, Northwest Ethiopia: competing risk regression model. BMC Pediatr. 2024;24(1):597.

4. Chi BH, Mbori-Ngacha D, Essajee S, Mofenson LM, Tsiouris F, Mahy M, et al. Accelerating progress towards the elimination of mother-to-child transmission of HIV: a narrative review. J Int AIDS Soc. 2020;23(8):e25571.

5. WHO. Mother-to-child transmission of HIV. https://www.who.int/teams/global-hiv-hepatitis-and-stis-programmes/hiv/prevention/mother-to-child-transmission-of-hiv#:~:text=Globally%2C%20an%20estimated%201.3%20million,been%20shown%20to%20be%20feasible. 2024 [

6. Omonaiye O, Kusljic S, Nicholson P, Manias E. Medication adherence in pregnant women with human immunodeficiency virus receiving antiretroviral therapy in sub-Saharan Africa: a systematic review. BMC public health. 2018;18:1-20.

7. Organization WH. Progress report 2016: prevent HIV, test and treat all: WHO support for country impact. Progress report 2016: prevent HIV, test and treat all: WHO support for country impact2016.

8. Organization WH. The role of HIV viral suppression in improving individual health and reducing transmission: policy brief: World Health Organization; 2023.

9. Chilaka VN, Konje JC. HIV in pregnancy–An update. European Journal of Obstetrics & Gynecology and Reproductive Biology. 2021;256:484-91.

10. Musanhu CCC, Takarinda KC, Shea J, Chitsike I, Eley B. Viral load testing among pregnant women living with HIV in Mutare district of Manicaland province, Zimbabwe. AIDS Research and Therapy. 2022;19(1):52.

11. Organization WH. Updated Recommendations on HIV Prevention, Infant Diagnosis, Antiretroviral Initiation and Monitoring: World Health Organization; 2022.

12. WHO Guidelines Approved by the Guidelines Review Committee. Consolidated Guidelines on the Use of Antiretroviral Drugs for Treating and Preventing HIV Infection: Recommendations for a Public Health Approach. Geneva: World Health Organization

Copyright © World Health Organization 2016.; 2016.

13. Gilleece DY, Tariq DS, Bamford DA, Bhagani DS, Byrne DL, Clarke DE, et al. British HIV Association guidelines for the management of HIV in pregnancy and postpartum 2018. HIV Med. 2019;20 Suppl 3:s2-s85.

14. Adelekan B, Harry-Erin B, Okposo M, Aliyu A, Ndembi N, Dakum P, et al. Final HIV status outcome for HIV-exposed infants at 18 months of age in nine states and the Federal Capital Territory, Nigeria. PLoS One. 2022;17(2):e0263921.

15. Rasmussen DN, Vieira N, Hønge BL, da Silva Té D, Jespersen S, Bjerregaard-Andersen M, et al. HIV-1 and HIV-2 prevalence, risk factors and birth outcomes among pregnant women in Bissau, Guinea-Bissau: a retrospective cross-sectional hospital study. Sci Rep. 2020;10(1):12174.

16. Organization WH. Updated recommendations on first-line and second-line antiretroviral regimens and post-exposure prophylaxis and recommendations on early infant diagnosis of HIV.

Updated recommendations on first-line and second-line antiretroviral regimens and post-exposure prophylaxis and recommendations on early infant diagnosis of HIV2018. p. [1-82].
17. Hoffman RM, Black V, Technau K, van der Merwe KJ, Currier J, Coovadia A, et al. Effects of highly active antiretroviral therapy duration and regimen on risk for mother-to-child transmission of HIV in Johannesburg, South Africa. J Acquir Immune Defic Syndr. 2010;54(1):35-41.
18. Organization WH. Guidelines: updated recommendations on HIV prevention, infant diagnosis, antiretroviral initiation and monitoring. Guidelines: updated recommendations on HIV prevention, infant diagnosis, antiretroviral initiation and monitoring2021.
19. Bulage L, Ssewanyana I, Nankabirwa V, Nsubuga F, Kihembo C, Pande G, et al. Factors associated with virological non-suppression among HIV-positive patients on antiretroviral therapy in Uganda, August 2014–July 2015. BMC infectious diseases. 2017;17:1-11.
20. Awaidy SA, Ghazy RM, Mahomed O. Progress of the gulf cooperation council (gcc) countries towards achieving the 95-95-95 UNAIDS targets: a review. Journal of epidemiology and global health. 2023;13(3):397-406.
21. Mwende Wairimu G. The Global Burden of HIV and Progress toward the 2030 UNAIDS Targets: A Comprehensive Review.
22. Mosha IH, Nyondo GG, Munishi CG, Njiro BJ, Bwire GM. Prevalence and factors associated with viral non-suppression in people living with HIV receiving antiretroviral therapy in sub-Saharan Africa: A systematic review and meta-analysis. Rev Med Virol. 2024;34(3):e2540.
23. Agegnehu CD, Techane MA, Mersha AT, Atalell KA. Burden and Associated Factors of Virological Failure Among People Living with HIV in Sub-Saharan Africa: A Systematic Review and Meta-Analysis. AIDS Behav. 2022;26(10):3327-36.
24. Woldesemayat B, Yizengaw A, Worku A, Yilma A, Adane S, Bulti J, et al. HIV Viral Non-Suppression and Its Associated Factors Among PMTCT Mothers Receiving ARV Treatment in Ethiopia. medRxiv. 2024:2024.06. 13.24308915.
25. Gabagaya G, Rukundo G, Amone A, Wavamunno P, Namale-Matovu J, Lubega I, et al. Prevalence of undetectable and suppressed viral load in HIV-infected pregnant women initiating Option B+ in Uganda: an observational study nested within a randomized controlled trial. BMC infectious diseases. 2021;21:1-7.

26. Munkhondya TEM, Smyth RM, Lavender T. Facilitators and barriers to retention in care under universal antiretroviral therapy (Option B+) for the Prevention of Mother to Child Transmission of HIV (PMTCT): A narrative review. International Journal of Africa Nursing Sciences. 2021;15:100372.

27. Kadia BM, Dimala CA, Fongwen NT, Smith AD. Barriers to and enablers of uptake of antiretroviral therapy in integrated HIV and tuberculosis treatment programmes in sub-Saharan Africa: a systematic review and meta-analysis. AIDS research and therapy. 2021;18(1):85.

28. Omonaiye O, Kusljic S, Nicholson P, Manias E. Medication adherence in pregnant women with human immunodeficiency virus receiving antiretroviral therapy in sub-Saharan Africa: a systematic review. BMC public health. 2018;18(1):805.

29. Page MJ, McKenzie JE, Bossuyt PM, Boutron I, Hoffmann TC, Mulrow CD, et al. The PRISMA 2020 statement: an updated guideline for reporting systematic reviews. BMJ (Clinical research ed). 2021;372:n71.

30. Organization WH. Consolidated guidelines on HIV prevention, testing, treatment, service delivery and monitoring: recommendations for a public health approach: World Health Organization; 2021.

31. Munn Z, Moola S, Lisy K, Riitano D, Tufanaru C. Methodological guidance for systematic reviews of observational epidemiological studies reporting prevalence and cumulative incidence data. JBI Evidence Implementation. 2015;13(3):147-53.

32. Barker TH HS, Aromataris E, Stone JC, Leonardi-Bee J, Sears K, Habibi N, Klugar M, Tufanaru C, Moola S, Liu XL. The revised JBI critical appraisal tool for the assessment of risk of bias for cohort studies. . JBI Evidence Synthesis 2025.

33. Huedo-Medina TB, Sánchez-Meca J, Marín-Martínez F, Botella J. Assessing heterogeneity in meta-analysis: Q statistic or I^2 index? Psychological methods. 2006;11(2):193.

34. Begg CB, Mazumdar M. Operating characteristics of a rank correlation test for publication bias. Biometrics. 1994:1088-101.

35. Ntombela NP, Kharsany ABM, Soogun A, Yende-Zuma N, Baxter C, Kohler HP, et al. Viral suppression among pregnant adolescents and women living with HIV in rural KwaZulu-Natal, South Africa: a cross sectional study to assess progress towards UNAIDS indicators and Implications for HIV Epidemic Control. Reprod Health. 2022;19(1):116.

36. Ngandu NK, Lombard CJ, Mbira TE, Puren A, Waitt C, Prendergast AJ, et al. HIV viral load non-suppression and associated factors among pregnant and postpartum women in rural northeastern South Africa: a cross-sectional survey. BMJ open. 2022;12(3):e058347.

37. Adeniyi OV, Obi CL, Goon DT, Iweriebor B, Selanto-Chairman N, Carty C, et al. Factors Associated With Peripartum Virologic Suppression in Eastern Cape Province, South Africa: A Retrospective Cross-Sectional Analysis. Clin Infect Dis. 2021;73(10):1750-8.

38. Chetty T, Newell ML, Thorne C, Coutsoudis A. Viraemia before, during and after pregnancy in HIV-infected women on antiretroviral therapy in rural KwaZulu-Natal, South Africa, 2010-2015. Trop Med Int Health. 2018;23(1):79-91.

39. Phillips TK, Clouse K, Zerbe A, Orrell C, Abrams EJ, Myer L. Linkage to care, mobility and retention of HIV-positive postpartum women in antiretroviral therapy services in South Africa. J Int AIDS Soc. 2018;21 Suppl 4(Suppl Suppl 4):e25114.

40. Woldesenbet SA, Kufa T, Barron P, Chirombo BC, Cheyip M, Ayalew K, et al. Viral suppression and factors associated with failure to achieve viral suppression among pregnant women in South Africa. Aids. 2020;34(4):589-97.

41. Woldesenbet S, Cheyip M, Lombard C, Manda S, Ayalew K, Kufa T, et al. Progress towards the UNAIDS 95-95-95 targets among pregnant women in South Africa: Results from the 2017 and 2019 national Antenatal HIV Sentinel Surveys. PLoS One. 2022;17(7):e0271564.

42. Woldesenbet S, Kufa T, Manda S, Ayalew K, Lombard C, Cheyip M, et al. Association between viral suppression during the third trimester of pregnancy and unintended pregnancy among women on antiretroviral therapy: Results from the 2019 antenatal HIV Sentinel Survey, South Africa. PLoS One. 2022;17(3):e0265124.

43. Myer L, Dunning L, Lesosky M, Hsiao NY, Phillips T, Petro G, et al. Frequency of Viremic Episodes in HIV-Infected Women Initiating Antiretroviral Therapy During Pregnancy: A Cohort Study. Clin Infect Dis. 2017;64(4):422-7.

44. Myer L, Phillips TK, Hsiao NY, Zerbe A, Petro G, Bekker LG, et al. Plasma viraemia in HIV-positive pregnant women entering antenatal care in South Africa. J Int AIDS Soc. 2015;18(1):20045.

45. Brittain K, Remien RH, Mellins CA, Phillips TK, Zerbe A, Abrams EJ, et al. Determinants of suboptimal adherence and elevated HIV viral load in pregnant women already on antiretroviral

therapy when entering antenatal care in Cape Town, South Africa. AIDS Care. 2018;30(12):1517-23.

46. Moyo F, Haeri Mazanderani A, Murray T, Technau KG, Carmona S, Kufa T, et al. Characterizing Viral Load Burden Among HIV-Infected Women Around the Time of Delivery: Findings From Four Tertiary Obstetric Units in Gauteng, South Africa. J Acquir Immune Defic Syndr. 2020;83(4):390-6.

47. Myer L, Phillips TK, McIntyre JA, Hsiao NY, Petro G, Zerbe A, et al. HIV viraemia and mother-to-child transmission risk after antiretroviral therapy initiation in pregnancy in Cape Town, South Africa. HIV Med. 2017;18(2):80-8.

48. Langwenya N, Phillips TK, Brittain K, Zerbe A, Abrams EJ, Myer L. Same-day antiretroviral therapy (ART) initiation in pregnancy is not associated with viral suppression or engagement in care: A cohort study. J Int AIDS Soc. 2018;21(6):e25133.

49. Anderson K, Kalk E, Heekes A, Phelanyane F, Jacob N, Boulle A, et al. Factors associated with vertical transmission of HIV in the Western Cape, South Africa: a retrospective cohort analysis. J Int AIDS Soc. 2024;27(3):e26235.

50. Moyo F, Mazanderani AH, Murray T, Sherman GG, Kufa T. Achieving maternal viral load suppression for elimination of mother-to-child transmission of HIV in South Africa. Aids. 2021;35(2):307-16.

51. Tsondai PR. HIV viral load monitoring in HIV-infected pregnant women established on antiretroviral therapy in Cape Town, South Africa: University of Cape Town; 2016.

52. Landes M, van Lettow M, van Oosterhout JJ, Schouten E, Auld A, Kalua T, et al. Early post-partum viremia predicts long-term non-suppression of viral load in HIV-positive women on ART in Malawi: Implications for the elimination of infant transmission. PLoS One. 2021;16(3):e0248559.

53. Chagomerana MB, Miller WC, Tang JH, Hoffman IF, Harrington BJ, DiPrete B, et al. Prevalence of antiretroviral therapy treatment failure among HIV-infected pregnant women at first antenatal care: PMTCT Option B+ in Malawi. PLoS One. 2018;13(12):e0209052.

54. Landes M, van Lettow M, Nkhoma E, Tippett Barr B, Truwah Z, Shouten E, et al. Low detectable postpartum viral load is associated with HIV transmission in Malawi's prevention of mother-to-child transmission programme. J Int AIDS Soc. 2019;22(6):e25290.

55. Chagomerana MB, Miller WC, Tang JH, Hoffman IF, Mthiko BC, Phulusa J, et al. Optimizing prevention of HIV mother to child transmission: Duration of antiretroviral therapy and viral suppression at delivery among pregnant Malawian women. PLoS One. 2018;13(4):e0195033.

56. Napyo A, Tumwine JK, Mukunya D, Tumuhamye J, Arach AAO, Ndeezi G, et al. Detectable HIV-RNA Viral Load Among HIV-Infected Pregnant Women on Treatment in Northern Uganda. Int J MCH AIDS. 2020;9(2):232-41.

57. Munyaneza A, Kim HY, Shi Q, Brazier E, Ross J, Muhoza B, et al. Factors associated with viral load suppression in pregnant and postpartum women living with HIV in Rwanda: an open-observational cohort study. Front Public Health. 2025;13:1544165.

58. Gill MM, Hoffman HJ, Bobrow EA, Mugwaneza P, Ndatimana D, Ndayisaba GF, et al. Detectable Viral Load in Late Pregnancy among Women in the Rwanda Option B+ PMTCT Program: Enrollment Results from the Kabeho Study. PLoS One. 2016;11(12):e0168671.

59. Koss CA, Natureeba P, Kwarisiima D, Ogena M, Clark TD, Olwoch P, et al. Viral Suppression and Retention in Care up to 5 Years After Initiation of Lifelong ART During Pregnancy (Option B+) in Rural Uganda. J Acquir Immune Defic Syndr. 2017;74(3):279-84.

60. Gabagaya G, Rukundo G, Amone A, Wavamunno P, Namale-Matovu J, Lubega I, et al. Prevalence of undetectable and suppressed viral load in HIV-infected pregnant women initiating Option B+ in Uganda: an observational study nested within a randomized controlled trial. BMC Infect Dis. 2021;21(1):907.

61. Humphrey JM, Songok J, Ofner S, Musick B, Alera M, Kipchumba B, et al. Retention in care and viral suppression in the PMTCT continuum at a large referral facility in western Kenya. AIDS Behav. 2022;26(11):3494-505.

62. Sandbulte M, Brown M, Wexler C, Maloba M, Gautney B, Goggin K, et al. Maternal viral load monitoring: Coverage and clinical action at 4 Kenyan hospitals. PLoS One. 2020;15(5):e0232358.

63. Boisson-Walsh A, Ravelomanana NLR, Tabala M, Malongo F, Kawende B, Babakazo P, et al. Association of comprehensiveness of antiretroviral care and detectable HIV viral load suppression among pregnant and postpartum women in the Democratic Republic of the Congo: a cross-sectional study. Front Glob Womens Health. 2024;5:1308019.

64. Yotebieng M, Mpody C, Ravelomanana NL, Tabala M, Malongo F, Kawende B, et al. HIV viral suppression among pregnant and breastfeeding women in routine care in the Kinshasa

province: a baseline evaluation of participants in CQI-PMTCT study. J Int AIDS Soc. 2019;22(9):e25376.

65. Woldesemayat B, Yizengaw A, Worku A, Yilma A, Adane S, Bulti J, et al. HIV Viral Non-Suppression and Its Associated Factors Among PMTCT Mothers Receiving ARV Treatment in Ethiopia. medRxiv. 2024:2024.06.13.24308915.

66. Hailu G, Keraleme A, Zealiyas K, Tesema A, Nuramed N, Girmachew F, et al. Human Immunodeficiency Virus (HIV) viral load suppression status and associated factors among pregnant women receiving Highly Active Antiretroviral Therapy (HAART) in Ethiopia. Virol J. 2025;22(1):49.

67. Demissie DB, Bulto GA, Mekuria WT, Dufera FN, Gamshe EN. Evaluation of Antiretroviral Therapy Initiated Among Pregnant Women Under Option B+ by Viral Load and CD4 Count Outcomes in Selected Hospitals of West Shewa Zone, Oromia Region, Ethiopia. HIV AIDS (Auckl). 2020;12:127-34.

68. Endalamaw Alamneh D, Shiferaw MB, Getachew Demissie M, Emiru MA, Zemene Kassie T, Endaylalu Lakew K, et al. Virological Outcomes Among Pregnant Women Receiving Antiretroviral Treatment in the Amhara Region, North West Ethiopia. HIV AIDS (Auckl). 2023;15:209-16.

69. Aduloju O, Aduloju T, Ade-Ojo I, Akintayo A. Medication adherence in HIV-positive pregnant women on antiretroviral therapy attending antenatal clinics in Ado metropolis, south-west Nigeria: A multicentre study. South African Journal of Obstetrics and Gynaecology. 2020;26:65.

70. Musanhu CCC, Takarinda KC, Shea J, Chitsike I, Eley B. Viral load testing among pregnant women living with HIV in Mutare district of Manicaland province, Zimbabwe. AIDS Res Ther. 2022;19(1):52.

71. Duri K, Munjoma P, Mazhandu A, Marere T, Gomo E, Banhwa S, et al. Predictors and Timing to Viral Suppression in HIV-Infected Pregnant Women in the University of Zimbabwe Birth Cohort Study During the Era of Lifelong Antiretroviral Therapy (Option B+ Treatment Strategy). Frontiers in Virology. 2022;2.

72. Atanga PN, Ndetan HT, Fon PN, Meriki HD, Muffih TP, Achidi EA, et al. Using a composite adherence tool to assess ART response and risk factors of poor adherence in pregnant

and breastfeeding HIV-positive Cameroonian women at 6 and 12 months after initiating option B. BMC Pregnancy Childbirth. 2018;18(1):418.

73. Kafack EVF, Fokam J, Nana TN, Saniotis A, Halle-Ekane GE. Evaluation of plasma viral-load monitoring and the prevention of mother-to-child transmission of HIV-1 in three health facilities of the Littoral region of Cameroon. PLoS One. 2022;17(11):e0277271.

74. Tegueni K, Gbeasor-Komlanvi FA, Adama OIW, Sadio AJ, Amenyah-Ehlan AP, Dagnra CA, et al. Epidemiological and virological surveillance of the prevention of mother-to-child transmission of HIV among pregnant women in Togo. BMC Pregnancy Childbirth. 2024;24(1):278.

75. Flynn PM, Taha TE, Cababasay M, Butler K, Fowler MG, Mofenson LM, et al. Association of Maternal Viral Load and CD4 Count With Perinatal HIV-1 Transmission Risk During Breastfeeding in the PROMISE Postpartum Component. J Acquir Immune Defic Syndr. 2021;88(2):206-13.

76. Mazuguni N, Mazuguni F, Muro E. Detectable viral load among HIV -1 positive pregnant women on ART (Option B +) in northern Tanzania: Baseline results from the HIVDR study. East Africa Science. 2021;3:44-50.

77. Schrubbe LA, Stöckl H, Hatcher AM, Marston M, Kuchukhidze S, Calvert C. Prevalence and risk factors of unsuppressed viral load among pregnant and breastfeeding women in sub-Saharan Africa: analysis from population-based surveys. AIDS. 2023;37(4):659-69.

78. Namaganda MM, Mukasa Kafeero H, Nakatumba Nabende J, Kateete DP, Batte C, Wanyengera M, et al. Prevalence and predictors of virological failure among the people living with HIV on antiretroviral treatment in East Africa: evidence from a systematic review with meta-analysis and meta-regression of published studies from 2016 to 2023. HIV Research & Clinical Practice. 2025;26(1):2490774.

79. Aytenew TM, Asferie WN, Ejigu N, Birhane BM, Tiruneh YM, Kassaw A, et al. Virological failure and associated factors among patients receiving anti-retroviral therapy in Ethiopia: A systematic review and meta-analysis. BMJ open. 2024;14(11):e087569.

80. Taieb F, Madec Y, Cournil A, Delaporte E. Virological success after 12 and 24 months of antiretroviral therapy in sub-Saharan Africa: Comparing results of trials, cohorts and cross-sectional studies using a systematic review and meta-analysis. PloS one. 2017;12(4):e0174767.

81. Melak D, Bayou FD, Yasin H, Zerga AA, Wagaye B, Ayele FY, et al. Virological Suppression and its Predictors Among HIV/AIDS Patients on Antiretroviral Therapy in Ethiopia: Systematic Review and Meta-analysis. Open Forum Infectious Diseases. 2024;11(4).
82. Mor G, Cardenas I. The immune system in pregnancy: a unique complexity. American journal of reproductive immunology. 2010;63(6):425-33.
83. Hodel EM, Marzolini C, Waitt C, Rakhmanina N. Pharmacokinetics, placental and breast milk transfer of antiretroviral drugs in pregnant and lactating women living with HIV. Current pharmaceutical design. 2019;25(5):556-76.
84. Mosha IH, Nyondo GG, Munishi CG, Njiro BJ, Bwire GM. Prevalence and factors associated with viral non-suppression in people living with HIV receiving antiretroviral therapy in sub-Saharan Africa: A systematic review and meta-analysis. Reviews in medical virology. 2024;34(3):e2540.
85. Jiang W. Adverse pregnancy outcomes, non-retention, eligibility of differentiated care and virologic failure among women living with HIV in prevention of vertical transmission programs: University of Washington; 2023.
86. Ankunda C. Longitudinal Analysis of Viral Suppression Before, During, and After Pregnancy Among Women on Antiretroviral Therapy in Uganda: Six-year Real-World Evidence. 2025.
87. Nyongesa MK, Mwatasa MH, Kagonya VA, Mwambingu G, Ngetsa C, Newton C, et al. HIV virological non-suppression is highly prevalent among 18- to 24-year-old youths on antiretroviral therapy at the Kenyan coast. BMC Infect Dis. 2022;22(1):449.
88. USAIDS. Young people and HIV. accessed on: https://www.unaids.org/sites/default/files/media_asset/young-people-and-hiv_en.pdf 2021 [
89. Hlophe LD, Tamuzi JL, Shumba CS, Nyasulu PS. Barriers and facilitators to anti-retroviral therapy adherence among adolescents aged 10 to 19 years living with HIV in sub-Saharan Africa: A mixed-methods systematic review and meta-analysis. PLoS One. 2023;18(5):e0276411.
90. Duri K, Munjoma PT, Mazhandu AJ, Marere T, Gomo E, Banhwa S, et al., editors. Predictors and Timing to Viral Suppression in HIV-Infected Pregnant Women in the University of Zimbabwe Birth Cohort Study During the Era of Lifelong Antiretroviral Therapy (Option B+ Treatment Strategy). Frontiers in Virology; 2022.

91. Melak D, Bayou FD, Yasin H, Zerga AA, Wagaye B, Ayele FY, et al., editors. Virological suppression and its predictors among HIV/AIDS patients on antiretroviral therapy in Ethiopia: Systematic review and meta-analysis. Open forum infectious diseases; 2024: Oxford University Press US.

92. Dessie G, Wagnew F, Mulugeta H, Amare D, Jara D, Leshargie CT, et al. The effect of disclosure on adherence to antiretroviral therapy among adults living with HIV in Ethiopia: a systematic review and meta-analysis. BMC Infect Dis. 2019;19(1):528.

93. Endalamaw A, Assefa Y, Geremew D, Belete H, Dachew BA, Belachew A, et al. Disclosure of HIV seropositivity to sexual partner in Ethiopia: A systematic review. Womens Health (Lond). 2021;17:17455065211063021.

94. Mekonnen FA, Lakew AM, Muchie KF, Teshome DF. Sero-positive HIV result disclosure to sexual partner in Ethiopia: a systematic review and meta-analysis. BMC Public Health. 2019;19(1):1743.

95. Zhao S, Wang W, Li S, He J, Duan W, Fang Z, et al. The prevalence of low-level viraemia and its association with virological failure in people living with HIV: a systematic review and meta-analysis. Emerging Microbes & Infections. 2025;14(1):2447613.

96. Temere BC, Mewahegn AA, Zewudie BT, GebreEyesus FA, Abebe H, Nuru A, et al. Burden of virologic failure, and its determinants among HIV positive children in East Africa: a systematic review and meta-analysis. African Health Sciences. 2024;24(3):1-9.

97. Kibui NK. Factors Associated With Virological Non-suppression in Treatment-experienced HIV+ Patients in Kenya: a Populationwide Study: University of Nairobi; 2024.

98. Ministry of Health E. National Guideline for Prevention of Mother-to-child Transmission of HIV, Syphilis and Hepatitis B Virus. Access: https://www.prepwatch.org/wp-content/uploads/2022/07/National-Guideline-for-Prevention-of-Mother-to-child-Transmission-of-HIV-Syphilis-and-Hepatitis-B-Virus-2021.pdf 2021 [

99. Sibiude J, Le Chenadec J, Mandelbrot L, Hoctin A, Dollfus C, Faye A, et al. Update of perinatal human immunodeficiency virus type 1 transmission in France: zero transmission for 5482 mothers on continuous antiretroviral therapy from conception and with undetectable viral load at delivery. Clinical infectious diseases. 2023;76(3):e590-e8.

100. Nachega JB, Marconi VC, van Zyl GU, Gardner EM, Preiser W, Hong SY, et al. HIV treatment adherence, drug resistance, virologic failure: evolving concepts. Infect Disord Drug Targets. 2011;11(2):167-74.

Table 1: Descriptive summary of studies included in systematic review of virologic suppression among HIV-positive pregnant and lactating women receiving ART in Africa.

Authors	Study design	Country	Study population	Sample size	Virologic Suppression (%)	Risk of bias
Adeniyi et al., 2021	CS	South Africa	Pregnant and postpartum	1463	82	Low
Aduloju et al., 2020	CS	Nigeria	Pregnant women	170	70	Low
Alamneh et al., 2023	CS	Ethiopia	Pregnant women	992	90.9	Low
Anderson et al., 2024	CH	South Africa	Pregnant women	50764	87.4	Low
Anderson et al., 2024	CH	South Africa	Postpartum women	50764	90.9	Low
Anderson et al., 2025	CH	South Africa	Intrapartum women	50764	83.8	Low
Boisson-Walsh et al et al., 2024	CS	DR Congo	Pregnant and postpartum	2295	66.5	Low
Brittain et al., 2018	CS	South Africa	Pregnant women	482	88	Low
Chagomerana et al., 2018	CH	Malawi	Postpartum women	252	84.1	Low
Chagomerana et al., 2018	CH	Malawi	Pregnant women	864	92.4	Low
Chagomerana et al., 2021	CH	Malawi	Pregnant women	819	86.5	Low
Chagomerana et al., 2022	CH	Malawi	Postpartum women	102	88.9	Low
Chetty et al., 2017	CH	South Africa	Pregnant and postpartum	3941	83.2	Low
Chetty et al., 2018	CH	South Africa	Pregnant women	150	77.3	Low
Chetty et al., 2019	CH	South Africa	Postpartum women	150	80.7	Low
Demissieet al., 2020	CS	Ethiopia	Pregnant women	319	89.7	
Duri et al., 2020	CH	Zimbabwe	Pregnant women	608	78.1	Low
Gabagaya et al., 2021	CH	Uganda	Pregnant women	532	29.1	Low
Hailu et al., 2025	CS	Ethiopia	Pregnant women	7764	96.8	Low
Kafack et al., 2022	CH	Cameroon	Pregnant women	135	89.7	Low
Koss et al., 2017	CH	Uganda	Pregnant women	150	80	Low
Landes et al., 2019	CS	Malawi	Postpartum women	1124	87.9	Low
Landes et al., 2021	CH	Malawi	Postpartum women	773	87.4	Low
Langwenya et al., 2018	CH	South Africa	Pregnant and postpartum	428	77	Low
M. Flynn et al., 2021	CH	SSA	Postpartum women	1220	75	Low
M. Gill et al., 2016	CH	Rwanda	Pregnant women	603	84.6	Low
M. Humphrey et al., 2022	CH	Kenya	Pregnant women	167	88	Low
M. Humphrey et al., 2023	CH	Kenya	Pregnant women	689	93	Low
Mazuguni et al., 2021	CS	Tanzania	Pregnant women	148	77	Low
Moyo et al., 2020	CS	South Africa	Pregnant women	2769	77.6	Low
Moyo et al., 2020	CH	South Africa	Pregnant women	40660	79.2	Low
Moyo et al., 2020	CH	South Africa	Intrapartum women	40660	85.7	Low
Munyaneza et al., 2025		Rwanda	Both women	530	91.9	Low
Musanhu et al., 2022		Zimbabwe	Pregnant women	121	87.7	Low
Myer et al., 2015	CS	South Africa	Pregnant women	574	87	Low
Myer et al., 2017	CH	South Africa	Pregnant women	523	78	Low
Myer et al., 2017	CS	South Africa	Pregnant women	620	91	Low

N. Atanga et al., 2018	CH	Cameroon	Pregnant women	268	92.7	Low
Napyo et al., 2020	CS	Uganda	Pregnant women	420	91.9	Low
Ndlangamandla et al., 2023	CS	Estiwani	Pregnant women	195	81.8	Low
Ngandu et al., 2022	CS	South Africa	Pregnant and postpartum	667	85.3	Low
Ntombela et al., 2022	CS	South Africa	Pregnant women	546	90.8	Low
Phillips et al., 2017	CS	South Africa	Pregnant and postpartum	452	92	Low
Sandbulte et al., 2020	CH	Kenya	Pregnant women	115	71	Low
Schrubbe et al., 2021	CS	SSA	Pregnant and postpartum	1685	63.8	Low
Schrubbe et al., 2022	CS	SSA	Pregnant women	560	59.9	Low
Schrubbe et al., 2023	CS	SSA	Postpartum women	1129	66	Low
Tsondai et al., 2016	CH	South Africa	Pregnant women	520	88	Low
Weldesenbet et al., 2020	CS	South Africa	Pregnant women	10052	73	Low
Woldesemayat et al., 2024	CS	Ethiopia	Pregnant women	496	97.8	Low
Woldesenbet et al., 2022	CS	South Africa	Pregnant women	8087	69.4	Low
Woldesenbet et al., 2022	CS	South Africa	Pregnant women	9733	73.3	Low
Yotebieng et al., 2019	CS	DR Congo	Pregnant women	1623	62	Low
Yotebieng et al., 2020	CS	DR Congo	Intrapartum women	1623	57	Low
Yotebieng et al., 2020	CS	DR Congo	Postpartum women	1623	65	Low

Key: CS: Cross-sectional studies, CH: cohort studies, DR: Democratic Congo, SSA: Sub-Saharan Africa

Table 2: Sub-group analysis for virologic suppression among HIV-positive pregnant and lactating women receiving ART in Africa.

Parameters	Studies	Samples	VS (%) at 95% CI	I^2 (%)	P-value
Study design					
Cohort studies	27		82.74% (80.80, 84.69)	99.35%	0.000
Cross-sectional studies	28		79.34% (74.06, 84.63)	99.69%	0.000
**Country**					
Cameroon	2		91.89% (89.23, 94.55)	0.00	0.327
DR Congo	4		62.66% (58.56, 66.75)	92.49	0.000
Ethiopia	4		94.11% (91.16, 97.07)	94.97	0.000
Kenya	3		84.82% (74.86, 94.78)	92.80	0.000
Malawi	6		88.06% (85.62, 90.51)	80.81	0.000
SSA	3		66.28% (60.04, 72.51)	95.09	0.000
South Africa	21		82.8% (80.42, 85.21)	99.64	0.000
Uganda	3		66.99% (23.93, 110.05)	99.72	0.000
Rwanda	2		88.30% (81.15, 95.46)	93.31	0.000
Zimbabwe	2		82.58% (73.20, 91.97)	87.27	0.005
Others*	3		76.52% (69.68, 83.35)	71.31	0.031
**Regions of Africa**					
Central Africa	4		62.66% (58.56, 66.75)	92.49	0.000
East Africa	13		83.49% (77.69, 89.3)	99.09	0.000
Sub-Saharan Africa	4		66.28% (60.04, 72.51)	95.09	0.000
Southern Africa	31		83.74% (81.72, 85.76)	99.47	0.000
Western Africa	3		84.49% (72.77, 96.20)	94.24	0.000
**Study population**					
Pregnant women	38		81.15% (78.66, 83.63)	99.61	0.000
Postpartum women	9		80.61% (73.28, 87.94)	99.08	0.000
Both	8		80.21% (73.25, 87.18)	98.87	0.000

*Nigeria, Estiwani, Tanzania

Click here to access/download;Figure;List of Figures.docx

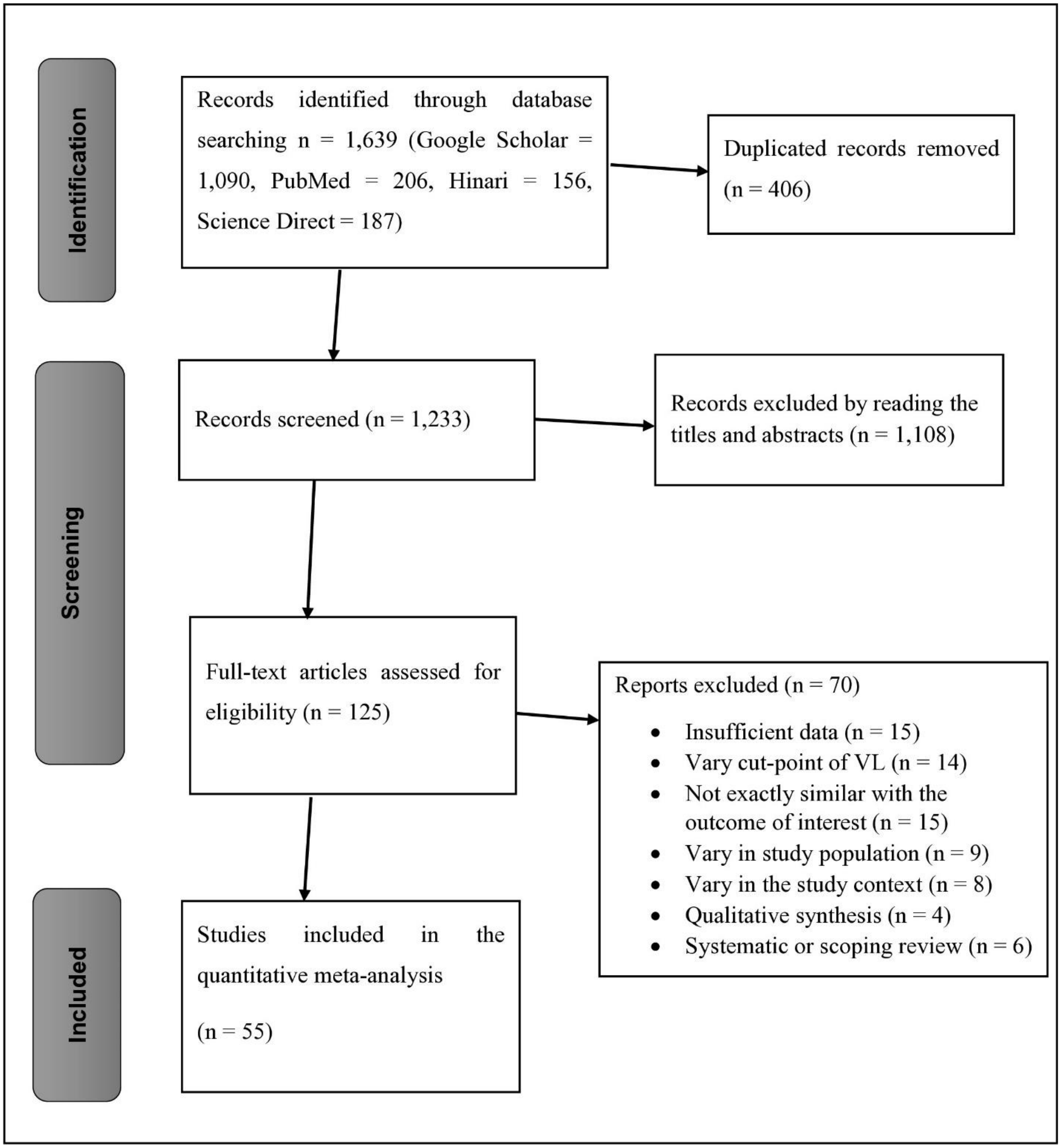

Figure 1: PRISMA flow chart showing the selection process of studies for the virologic suppression among HIV-positive pregnant and lactating women receiving ART in Africa.

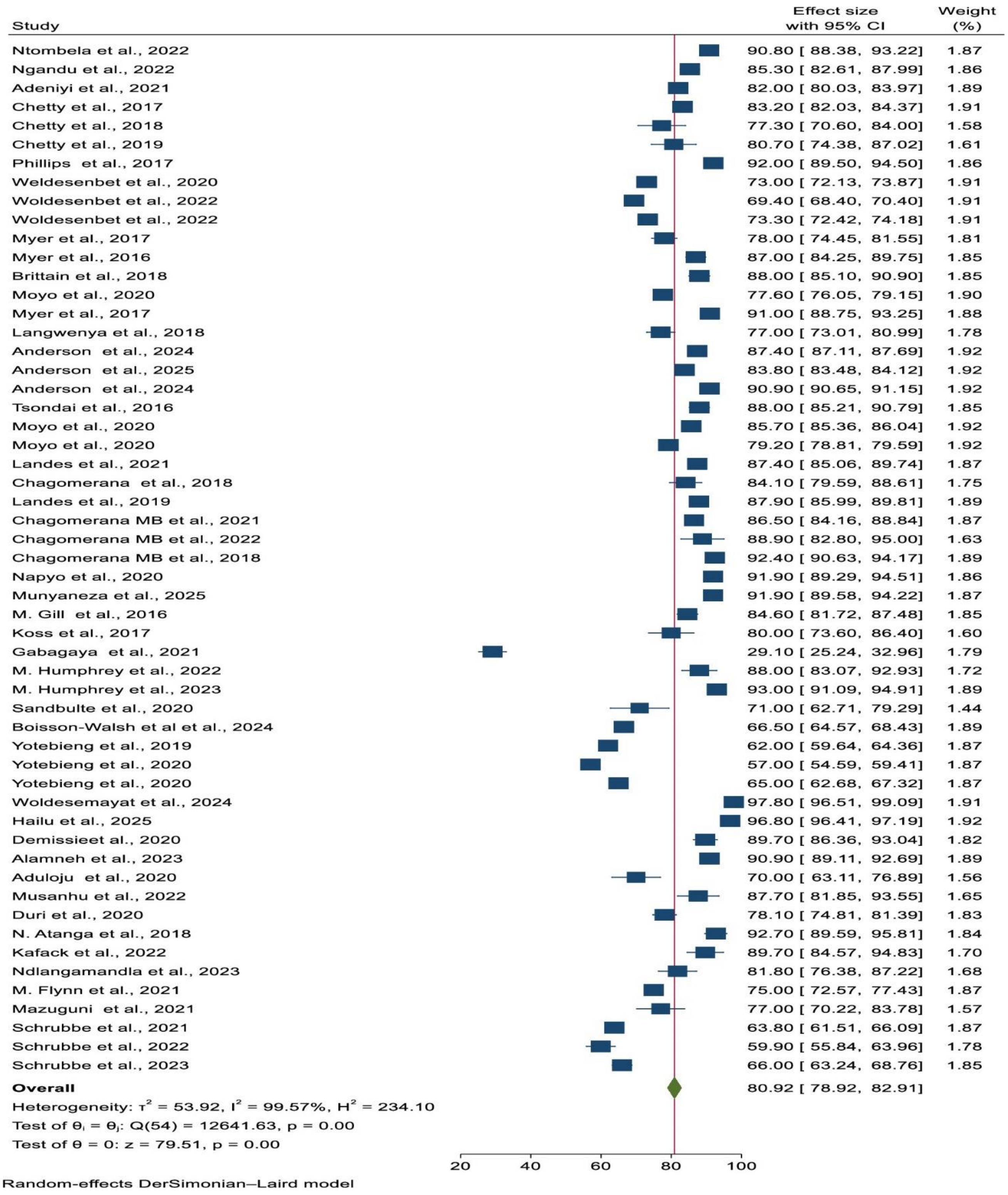

Figure 2: A forest plot that shows the pooled prevalence of virologic suppression among HIV-positive pregnant and lactating women receiving ART in Africa.

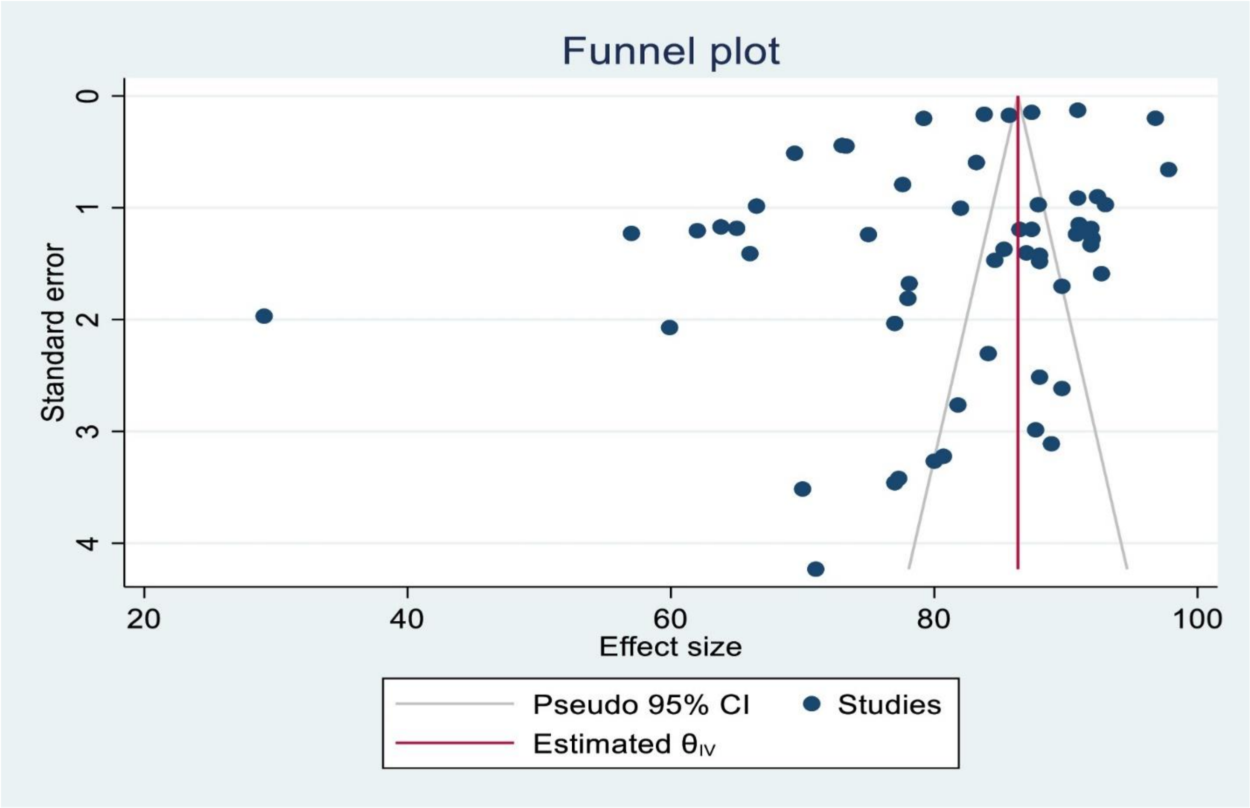

Figure 3: A funnel plot test that indicates the virologic suppression among HIV-positive pregnant and lactating women receiving ART in Africa.

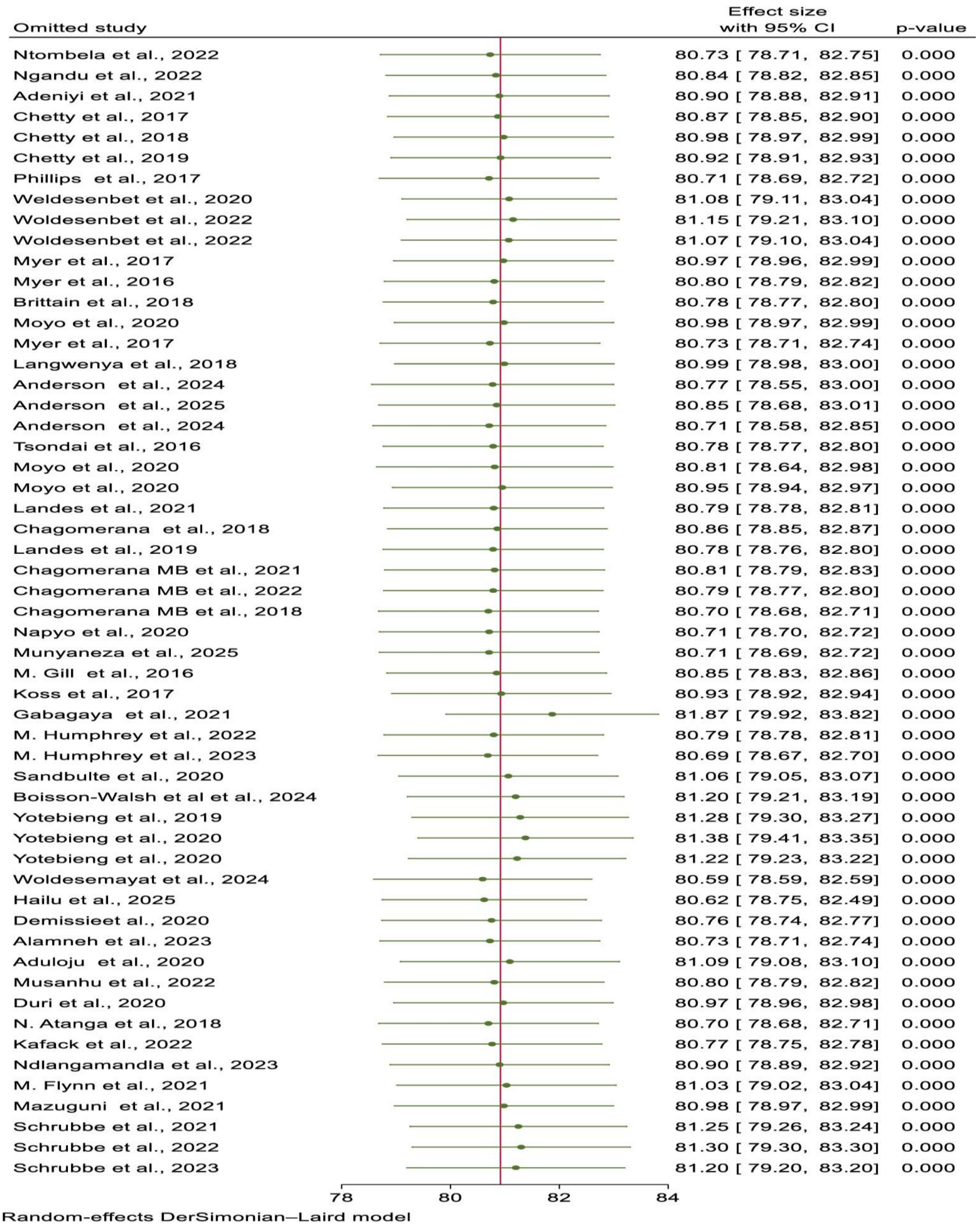

Figure 4: A leave-one-out sensitivity analysis of virologic suppression among HIV-positive pregnant and lactating women on ART in Africa.

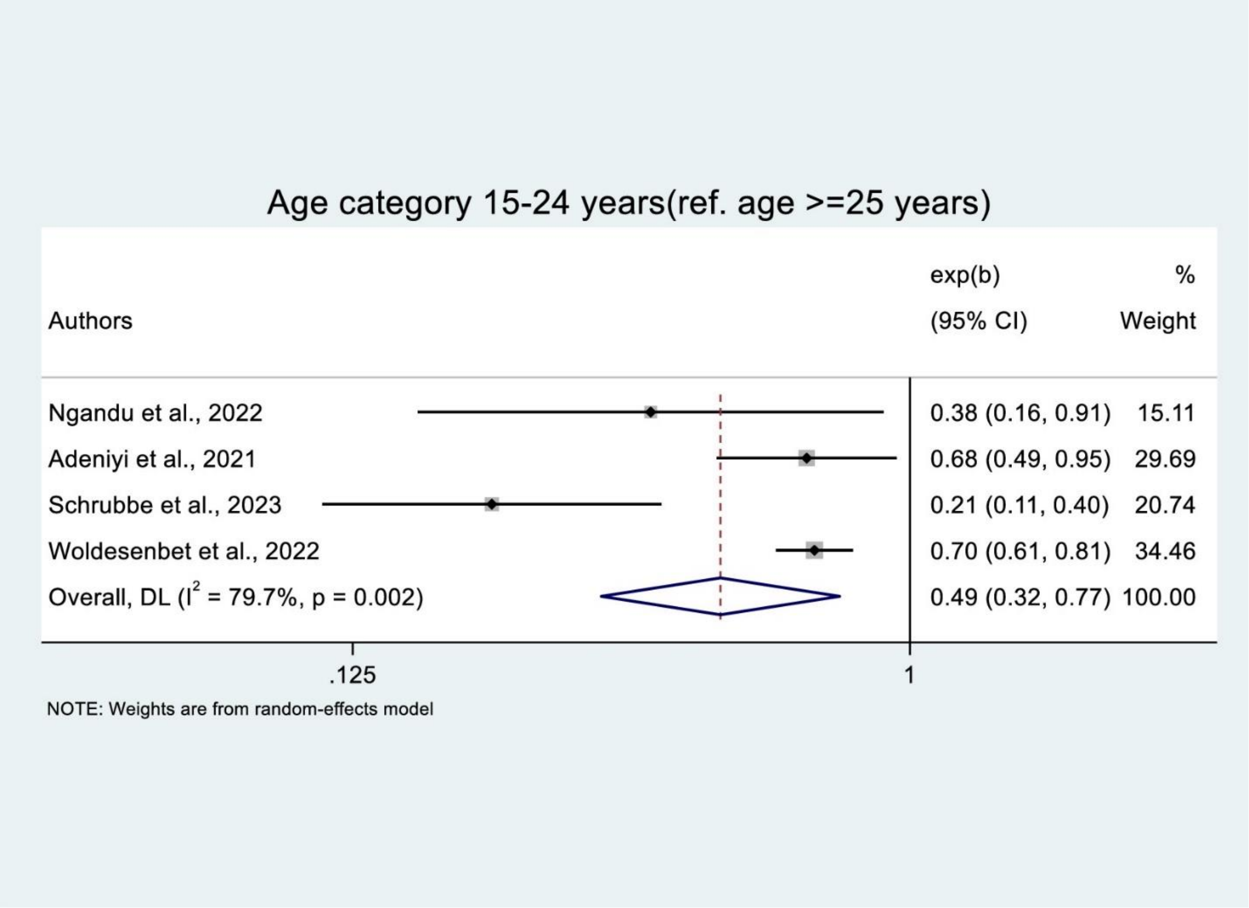

Figure 5: A Forest plot of the adjusted odds ratios with corresponding 95% CIs of studies on the association of young age (15-24 years) and virologic suppression among HIV-positive pregnant and lactating women on ART in Africa.

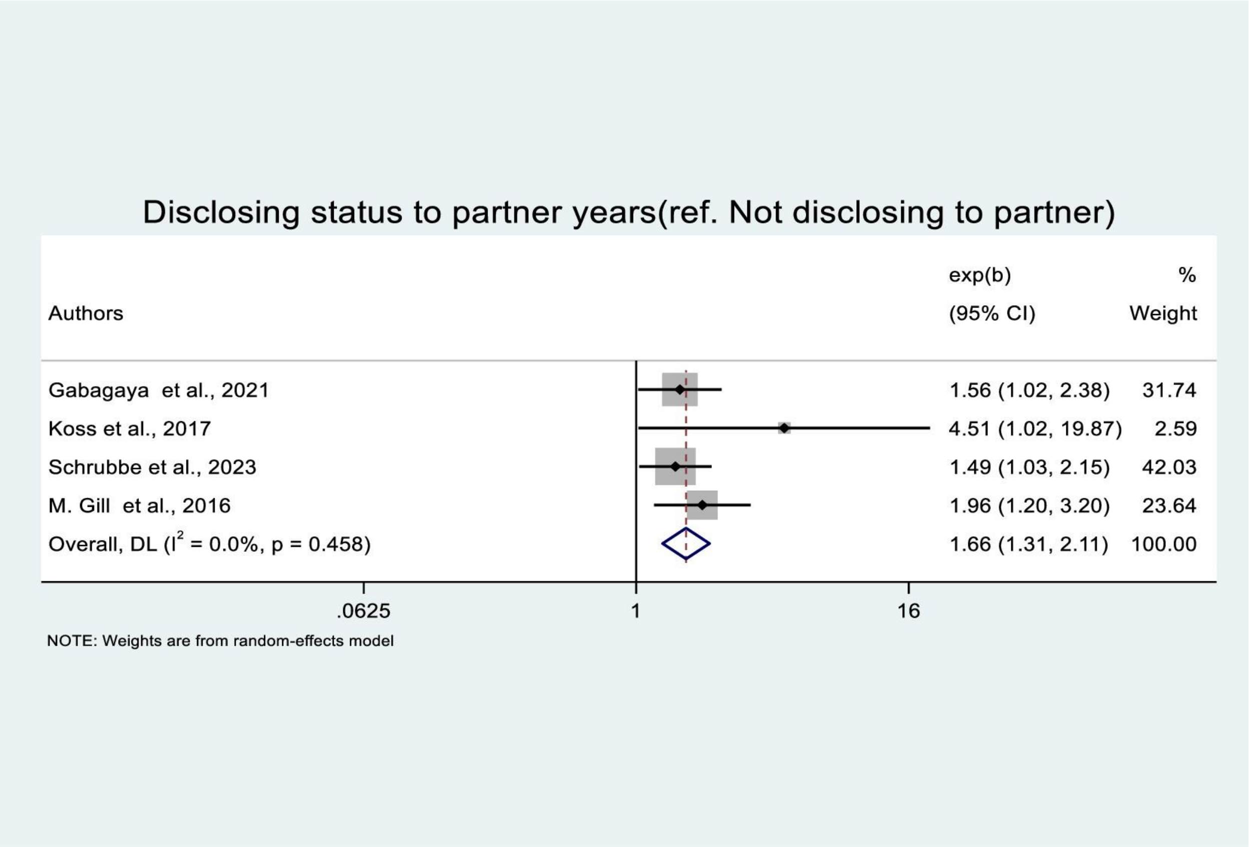

Figure 6: A Forest plot of the adjusted odds ratios with corresponding 95% CIs of studies on the association of disclosure of HIV status to partner and virologic suppression among HIV-positive pregnant and lactating women on ART in Africa.

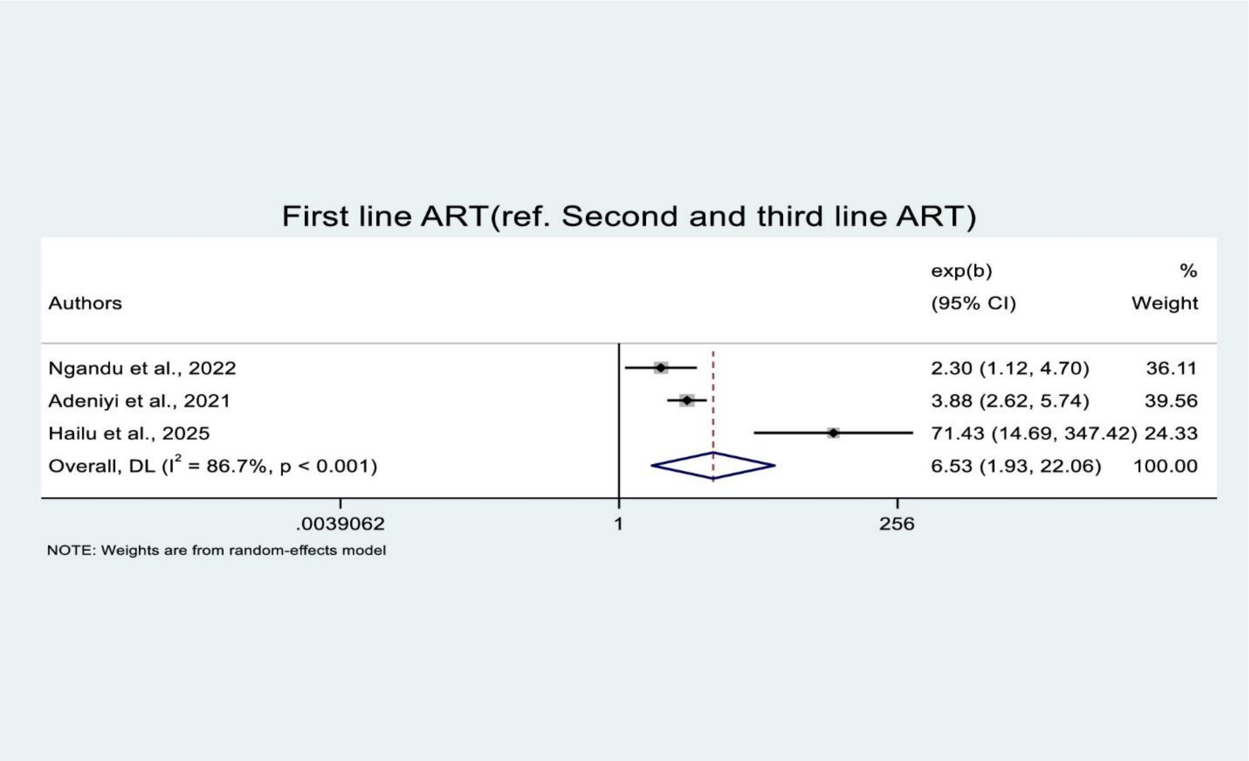

Figure 7: A Forest plot of the adjusted odds ratios with corresponding 95% CIs of studies on the association of first-line ART and virologic suppression among HIV-positive pregnant and lactating women on ART in Africa.

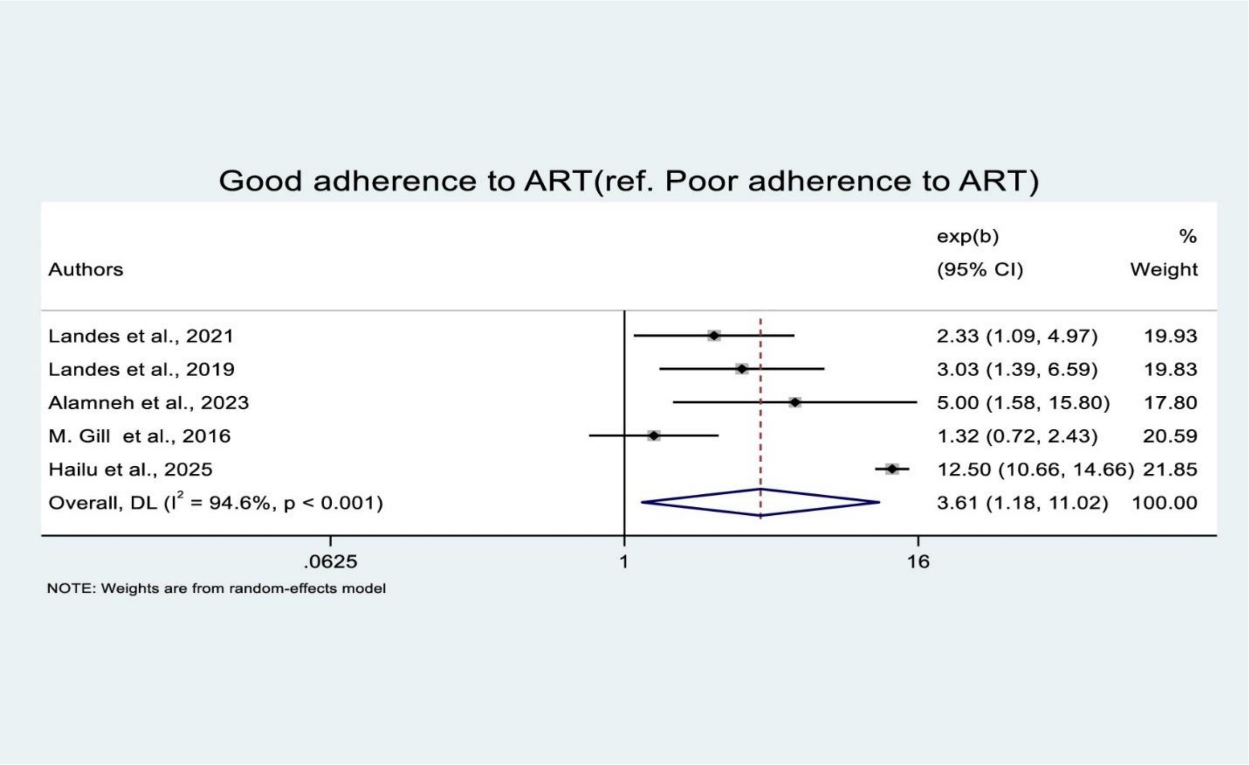

Figure 8: A Forest plot of the adjusted odds ratios with corresponding 95% CIs of studies on the association between good ART adherence and virologic suppression among HIV-positive pregnant and lactating women on ART in Africa.

S1 file

Click here to access/download
Supporting Information
S1 file PRISMA Checklist 2020.docx

Click here to access/download
Supporting Information
S2 file Search Strategies.docx

Click here to access/download
Supporting Information
S3 file Data_Extraction.xlsx
