# Supplementary material for: Virologic suppression among HIV-positive pregnant and lactating women receiving antiretroviral therapy in Africa: A systematic review and meta-analysis
Source: PLoS One. 2026 Apr 13;21(4):e0346045. doi: 10.1371/journal.pone.0346045 (PMC13075719; doi:10.1371/journal.pone.0346045)
Supplement: Attachment — Submitted filename: Review_meta-analysis.pdf [file pone.0346045.s010.pdf]

Manuscript Number: PONE-D-25-49195

Full Title: Virologic suppression among HIV-positive pregnant and lactating women receiving antiretroviral therapy in Africa: A systematic review and meta-analysis
Keywords: Africa; Antiretroviral therapy; HIV; pregnant women; lactating women; virologic Suppression

Comments and Recommendations

1. **Introduction**:
    a. Start with a wider lens on HIV in maternal health before narrowing to Africa.
    b. Define key terms early (e.g., VS, VLS, ART, PMTCT).
    c. Explicitly mention what the study aims to test or explore.

2. **Methods:**
    a. Specify exact search strings and Boolean logic used.
    b. Clarify how "Africa context" was defined and handled regionally.
    c. Mention tools/software used (e.g., RevMan, STATA) and how quality assessment was performed.
    d. Justify the choice of 2016–2025 as the publication window.
3. **Quality Assessment**
    a. Summarize how many studies met the quality threshold.
4. **Data Extraction**
    a. Explain handling of missing or inconsistent data.
5. **Statistical Analysis**
    a. Clarify how overlapping data was identified and excluded.
6. **Results**
    a. Include a summary table of study characteristics (e.g., country, design, sample size).
    b. Expand on regional distribution and differences in suppression rates.
    c. Explain why subgroup analysis didn't reduce heterogeneity.
7. **Discussion**
    a. Suggest future research directions (e.g., longitudinal, qualitative studies).

Conclusion

Overall, this is good research, this review takes a deep dive into how well HIV-positive pregnant and breastfeeding women in Africa are responding to antiretroviral therapy, specifically looking at how many are achieving virologic suppression. The study is methodologically solid, using validated tools and statistical models to analyze the data. It also identifies key factors that influence outcomes, like age, relationship status, ART regimen, HIV status disclosure, and adherence. Younger women and those with poor adherence were less likely to achieve suppression, pointing to the need for youth-friendly care and stronger support systems. While the results are promising, the high variability across studies and regions suggests more targeted interventions and further research are needed to close the gap and reduce mother-to-child transmission.
