# Supplementary material for: Virologic suppression among HIV-positive pregnant and lactating women receiving antiretroviral therapy in Africa: A systematic review and meta-analysis
Source: PLoS One. 2026 Apr 13;21(4):e0346045. doi: 10.1371/journal.pone.0346045 (PMC13075719; doi:10.1371/journal.pone.0346045)
Supplement: Attachment — Submitted filename: Review 2.pdf [file pone.0346045.s011.pdf]

Manuscript Title: Virologic suppression among HIV-positive pregnant and lactating women receiving antiretroviral therapy in Africa: A systematic review and meta-analysis

Reviewer: Duressa Shafi Ahmed

Date: October 22, 2025

1. General Assessment

This manuscript presents a systematic review and meta-analysis evaluating the prevalence of virologic suppression and its associated factors among HIV-positive pregnant and lactating women receiving ART in Africa. The study is timely, methodologically sound, and addresses a critical gap in maternal HIV care across the continent. The authors adhered to PRISMA guidelines, registered their protocol on PROSPERO, and employed appropriate statistical techniques including random-effects modeling, subgroup analysis, and sensitivity testing. The manuscript is generally well-written and organized, with clear objectives and relevant findings. However, several areas require clarification or refinement to meet PLOS ONE's standards for transparency, reproducibility, and scientific rigor.

2. Section-by-Section Evaluation

Methods and Materials

Strengths:

- PRISMA adherence and PROSPERO registration enhance transparency.
- Use of CoCoPop framework and JBI quality assessment tools is appropriate.
- Clear data extraction and statistical procedures.

Areas for Improvement:

1. **Search Strategy**: The Boolean search string is extensive but lacks structure. Present full search strategies (e.g., for PubMed) in a supplementary file.
2. **Database Justification**: Justify inclusion of Google Scholar due to its non-standard indexing.
3. **Quality Threshold**: Rephrase "excellent quality" for studies scoring ≥5 on JBI to "acceptable quality."

4. **Outcome Definitions**: Clarify whether all studies used the same VL threshold (≤1000 copies/mL).
5. **Statistical Rationale**: Justify use of DerSimonian and Laird’s method over alternatives like Hartung-Knapp.
6. **Publication Bias Tests**: Clarify whether Egger’s and Begg’s tests were applied to both prevalence and AORs.

Results

Strengths:

- Clear reporting of study selection and pooled prevalence.
- Appropriate use of random-effects model and sensitivity analysis.
- Subgroup analysis by region and population adds depth.

Areas for Improvement:

1. **PRISMA Flow**: Include a complete PRISMA diagram and a table of excluded studies with reasons.
2. **Heterogeneity**: $I^2 = 99.57\%$ is extremely high. Consider meta-regression or deeper exploration of sources.
3. **Subgroup Analysis**: Report statistical significance of subgroup differences and I^2 values per subgroup.
4. **Associated Factors**: Provide AORs and CIs for all discussed predictors. Clarify model type used for pooling.
5. **Figures**: Ensure all figures (e.g., funnel plot, forest plots) are clearly labeled and referenced.

Discussion, Strengths & Limitations, Conclusion

Strengths:

- Thoughtful interpretation of findings and comparison with regional/global benchmarks.
- Policy-relevant recommendations for youth-friendly services and integrated maternal HIV care.
- Honest reflection on limitations and geographic imbalance.

Areas for Improvement:

1. **Interpretation**: Discuss clinical significance of the 14% gap from the 95% target.
2. **Causal Language**: Soften causal claims to reflect observational nature (e.g., “associated with”).
3. **Policy Framing**: Reference WHO/UNAIDS frameworks when recommending interventions.
4. **Limitations**: Clarify geographic skew and discuss potential impact of publication bias despite non-significant tests.
5. **Conclusion**: Separate findings from recommendations for clarity. Specify which health system components need strengthening.

3. Minor Editorial Suggestions

- Correct grammar and phrasing (e.g., “All searching databases” → “All searched databases”).
- Ensure consistent terminology: “virologic suppression” vs. “viral load suppression.”
- Fix typos (e.g., “studies is required” → “studies are required”).

4. Recommendation

Minor to Moderate Revision

The manuscript is suitable for publication in PLOS ONE pending revisions to improve clarity, transparency, and interpretive precision.

5. Confidential Comments to the Editor

The manuscript addresses an important public health issue and is methodologically sound. I recommend acceptance after minor to moderate revisions. The authors should clarify their search strategy, refine their interpretation of heterogeneity, and improve the precision of their discussion on associated factors. Geographic imbalance and high heterogeneity should be acknowledged more explicitly.
