# Supplementary material for: Virologic suppression among HIV-positive pregnant and lactating women receiving antiretroviral therapy in Africa: A systematic review and meta-analysis
Source: PLoS One. 2026 Apr 13;21(4):e0346045. doi: 10.1371/journal.pone.0346045 (PMC13075719; doi:10.1371/journal.pone.0346045)
Supplement: Attachment — Submitted filename: Response to Reviewers.pdf [file pone.0346045.s016.pdf]

December 24, 2025

Virologic suppression among HIV-positive pregnant and lactating women receiving antiretroviral therapy in Africa: A systematic review and meta-analysis

Manuscript number: PONE-D-25-49195

Dear **Richard Makurumidze (MBChB, MPH, PhD, FCPHP, Academic Editor),** we are delighted to receive your comments for our submission to PLOS One. We are also grateful for the honored reviewers; their scientific comments, expertise, and guidance have been fundamental for enhancing the overall quality of our paper. We have diligently addressed the raised comments and suggestions given by the reviewers. All the amendments highlighted using tracked changes. The changes have been fully included in the revised manuscript. Finally, we kindly invite you to take a look at a point-by-point response below:

Point-by-point response to Reviewers' comments

Reviewer #1: The manuscript addresses a critical public health concern by evaluating virologic suppression among HIV-positive pregnant and lactating women in Africa. Its significance is evident given the implications for mother-to-child transmission and the broader context of achieving global HIV targets. The inclusion of 55 studies and over 300,000 participants strengthen the analysis through a large sample size, and the use of PRISMA guidelines, PROSPERO registration, and appropriate statistical tools such as random-effects models and Egger's tests demonstrate methodological rigor. The study presents relevant findings with public health utility, highlighting key factors such as age, adherence, disclosure of HIV status, and ART regimen as significantly associated with viral suppression.

Authors' response: Dear Reviewer, we would like to express our sincere gratitude for your valuable time and constructive feedback to our manuscript. We greatly appreciate your insightful comments and for sharing your expertise to improve our paper. In response to the reviewer's thoughtful comments and suggestions, we have carefully and thoroughly addressed each of the

points raised. As a result, we carried out a substantial revision throughout the manuscript and we have addressed all concerns raised by the reviewer as much as possible.

Reviewer's comment: Despite these strengths, several issues warrant attention. A major concern lies in the extremely high heterogeneity ($I^2 > 99\%$), which remains unexplained even after subgroup analyses. The use of meta-regression would have been appropriate to explore potential sources of heterogeneity more systematically. The overrepresentation of studies from South Africa introduces geographical bias, making it difficult to generalize findings across the diverse healthcare contexts of the African continent. Furthermore, although the authors state there was no publication bias, the Egger's test result ($p = 0.0512$) suggests borderline significance, which should not be dismissed as negligible without further investigation

Authors' response: Dear reviewer, thank you for these thoughtful comments. We acknowledge the high heterogeneity and agree that meta-regression would have been valuable. As a result, we have performed meta-regression analysis to examine potential moderators for the high heterogeneity. We also recognize the overrepresentation of studies in South African and have clearly noted its potential effect on generalizability of the finding as a limitation. Regarding assessing publication bias, we have re-analyzed using a random-effects model with Restricted maximum–likelihood (REML). Then, the statistical test (Egger's test) shows non-significant.

Reviewer's comment: The study also overlooks key confounding factors such as socioeconomic status, healthcare accessibility, or rural–urban disparities, which are known to influence ART outcomes. In addition, the reporting lacks clarity in some areas.

Authors' response: Thank you for the comment. We were constrained by the variables mentioned in the original papers because our review is based on secondary data from preexisting studies, making it tough to extract and identify all possible factor affecting virologic suppression in pregnant and lactating women. However, in response to the reviewer's comment, we strengthened our systematic synthesis by including additional variables when the available data permitted. The results and discussion sections have undergone significant revisions.

Reviewer's comment: The year range for included studies curiously extends to 2025, which undermines credibility and suggests a typographical or planning oversight. The quality

assessment using the Joanna Briggs Institute tool is not detailed, and no summary of individual study scores is provided, which limits transparency.

Authors' response: Thank you. Thank you for this helpful comment. The study period of 2016–2025 is correct, as our review includes all eligible publications up to 2025. We have clarified this as all articles between January 1, 2016 (benchmark for the beginning of the Sustainable Development Goals (SDG) era). We also appreciate the feedback on the quality assessment and have added the Joanna Briggs Institute criteria with a summary of each study scores in S4 file.

Reviewer's comment: Wide confidence intervals for certain effect estimates (e.g., ART adherence) further point to imprecision or potential small-study effects.

Authors' response: We appreciate your insightful comment. Exactly, the possible reason for slightly wide CI could be related with number of studies included. Along with the meta-analysis, we try to carry out a systematic synthesis to elaborate factors associated with VS.

Reviewer's comment: Narratively, the discussion is repetitive, often restating results without providing deeper contextual interpretation or drawing clear distinctions between policy implications and existing literature. The paper also misses the opportunity to provide targeted, practical recommendations for policymakers, especially considering its relevance to achieving UNAIDS 95-95-95 targets. Finally, factors such as ART duration, viral load assay variability, and breastfeeding practices, which could significantly influence virologic suppression, are insufficiently addressed. Overall, while the study presents valuable findings, it would benefit from a more nuanced analytical approach, clearer methodological transparency, and a stronger focus on contextual and actionable insights.

Authors' response: We appreciate the reviewer's insightful and helpful comments. As a result, we changed the Discussion to focus more on understanding and contextualizing within the body of current literature and less on repetition. We improve the link between our findings and advancement toward the UNAIDS 95–95–95 targets and more clearly differentiate implications for policy from previous studies. Additionally, we included more detailed and practical suggestions for decision-makers. We also went into more detail on important variables that affect virologic suppression, including as study socioeconomic status, viral load assay variability, and

the duration of ART. Overall, these revisions could enhance the analytical rigor, transparency, and practical relevance of the manuscript.

Reviewer #2: thank you for your interested topic

The study is scientifically relevant, methodologically solid, and has potential impact for HIV programmatic policy in Africa. I advise to Revise for minor grammar mistakes, consistent abbreviation use, and concise phrasing.

Authors' response: Dear Reviewer #2, thank you so much for your comments on grammatical errors, consistency and precision. We have considered the reviewer's comments throughout the manuscript to enhance its rigorous and clarity.

Reviewer #3: 1. Does the title accurately reflect the study's objectives and population?

Authors' response: We appreciate the reviewer's comment. We agree that the title adequately describes the study's emphasis and objectives. It precisely identifies the target population (HIV-positive pregnant and lactating women on ART), the study area (Africa), and the outcome of interest (virologic suppression), all of which are in line with the objectives of our systematic review and meta-analysis.

Reviewer #3: 2. The abstract mentions an 80.92% pooled prevalence should confidence intervals or heterogeneity measures (I2) also appear for completeness?

Authors' response: Thank you! We have stated the confidence intervals and heterogeneity measures (I^2) in the abstract section.

Reviewer #3: 3. Were the inclusion and exclusion criteria too broad (2016–2025) given the variability in ART regimens over time?

Authors' response: Thank you for your insightful comment. While our inclusion period spans years in which ART regimens evolved, most included studies were based on the Option B+ regimen. Only a few studies were in the current first-line regimen (TDF-3TC-DTG). To address this variability, we conducted a meta-analysis analysis by ART regimen, allowing us to account for differences in treatment protocols over time. As a result, ART regimen was found one of the moderators or covariates for virologic suppression. We have included all the changes in the revised manuscript.

Reviewer #3: 4. How were overlapping datasets or duplicate reports from large cohort programs handled?

Authors' response: Thank you for raising this important point. We thoroughly examined each study for overlapping datasets to prevent duplication. We only included the dataset that offered the most pertinent and non-overlapping data for our results when reports were from the same cohort program. We only considered publications that examined different time periods (pregnancy, intrapartum, or postpartum) to be independent if the populations did not overlap. Furthermore, other studies have reported the VS for certain study populations, such as breastfeeding mothers and pregnant women, at a particular point in time.

Reviewer #3: 5. Were data extraction and bias assessments blinded and independently verified by more than two reviewers?

Authors' response: Absolutely! Data extraction from the included publications was performed independently by two authors (BAM and NTT).

Reviewer #3: 6. How did the authors deal with variations in the definition of "virologic suppression" across studies (e.g., ≤50 vs ≤1000 copies/mL)?

Authors' response: I appreciate you making this crucial insight. By using the WHO definition, which defines virologic suppression as VL ≤1000 copies/mL, we were able to address differences in criteria. Furthermore, we extracted data independently and computed a pooled estimate of undetectable viral load for studies that reported VL ≤50 copies/ml. This approach helps us to harmonize the cutoff points for viral load thresholds.

Reviewer #3: 7. Given the high heterogeneity (I2 = 99.57%), was the use of a pooled random-effects model justified without meta-regression?

Authors' response: Thank you for highlighting this concern. We used a random-effects model because substantial methodological and contextual differences across studies were anticipated. To further explore the observed high heterogeneity, we conducted subgroup analyses based on key study characteristics (ART regimen, study design, and geographic region). Thank you for raising this important point. In addition, we conducted a meta-regression using sample size, study period, country, ART regimen, and African region as covariates to explore sources of

heterogeneity. ART regimen, and regions of Africa were found significant covariate for virologic suppression. All changes have been included in the revised manuscript.

Reviewer #3: 8. Could differences in study design (cross-sectional vs. cohort) have contributed to heterogeneity, and were these tested?

Authors' response: Thank you for this insightful comment. We conducted a subgroup analysis to see if the study design was a factor in the reported heterogeneity. For both cohort and cross-sectional studies, the pooled prevalence was 82.74% and 79.34%, respectively. The I-square remains significantly high in both groups (99.35% and 99.69%, respectively), despite the fact that the prevalence estimates are comparatively equal across the two study designs. This suggests that the statistical heterogeneity in this meta-analysis cannot be explained by the differences between cross-sectional and cohort designs.

Reviewer's comment: 9. how were missing data handled in the included studies?

Authors' response: Thank you for your insightful comment. Our analysis and findings relied on the reported data of the original studies. The majority of included studies state how missing data were handled or excluded the study subject with missing viral load values from their analyses. Due to the lack of individual participant-level data, we did not do extra imputation for missing outcome data.

Reviewer's comment: Could differences in viral-load testing availability or assay methods influence reported suppression rates?

Authors' response: Thank you very much for this scientific comment. We appreciate the reviewer's insightful comment regarding the influence of viral load (VL) testing availability and assay methods. We agree that these are critical determinants for regular assessment of VL. For instance, limited access to routine testing often results in 'targeted' testing for virologic failure.

Reviewer #4: Thank you for the opportunity to review this manuscript.

The topic, "virologic suppression among HIV-positive pregnant and lactating women in Africa" is important and policy-relevant. With a few targeted methodological clarifications and stronger transparency, this can become a solid, reproducible synthesis.

Authors' response: Thank you very much for your time and effort to review our manuscript. We express our gratitude to Reviewer #4 for giving extensive evaluation of our work and for acknowledging the significance and policy relevance of virologic suppression among pregnant and breastfeeding women in Africa. We value the constructive feedback that was provided. In response to your recommendations, we have improved the transparency of our reporting and included the necessary methodological explanations that ensure that the manuscript fulfills the highest requirements for a reproducible synthesis. We have diligently revised the main manuscript and our detailed responses to your specific points are provided below.

Reviewer #4: Major comments

1) Outcome definition & measurement window

Please harmonize the outcome definition and clarify, at study level, the VL threshold used (e.g., ≤1000 vs other), specimen type (plasma vs DBS), and timing (pregnancy trimester / intrapartum / postpartum window). Provide either:

• A succinct subgroup/meta-regression using these moderators; or

• A sensitivity analysis restricted to ≤1000 copies/ml.

This avoids definitional mixing and improves interpretability.

Authors' response: Thank you for your valuable comments.

We thank the reviewer for this important suggestion to improve the precision and transparency of our findings. We have addressed these points as follows:

To ensure consistency, our primary meta-analysis was restricted to studies using the standard threshold of ≤1000 copies/ml. We have performed a sensitivity analysis exclusively including studies that strictly utilized this threshold, which confirmed the robustness of our pooled prevalence estimates.

We recognize that virologic suppression (VS) could fluctuate across the continuum maternity care. Accordingly, we have conducted a subgroup analysis categorized by the timing of measurement: pregnancy (by trimester where available), intrapartum, and postpartum periods.

However, the subgroup analysis did not show significant variation in the VS. Additionally, based on the included studies, viral load threshold was determined commonly utilized blood plasma.

2) Modeling proportions under extreme heterogeneity

Given the very high between-study heterogeneity ($I^2 \approx 99.6\%$), please complement Der Simonian–Laird with HKSJ or REML, and add a logit/GLMM approach suitable for proportions (with appropriate continuity correction where needed).

In the main text, report τ^2 and a 95% prediction interval (PI) alongside the pooled mean.

Authors' response: Thank you for this valuable comment. We compared pooled estimates of viral load suppression using three random-effects meta-analysis estimators: Der Simonian–Laird (DL), Sidik–Jonkman (SJ), and restricted maximum likelihood (REML). All analyses included 55 studies and demonstrated extremely high between-study heterogeneity. Across all three methods, the pooled estimates were remarkably consistent, with point estimates centered around 81% VS. The Der Simonian–Laird method yielded a pooled estimate of 80.92% (95% CI: 78.92–82.91), while both REML and Sidik–Jonkman produced nearly identical pooled estimates of 80.86%, with slightly wider CI (REML: 77.63, 84.09; Sidik–Jonkman: 77.63, 84.08). Given the extreme heterogeneity observed ($I^2 > 99\%$), REML was prioritized for inference due to its favorable statistical properties and reduced bias in estimating between-study variance.

Additionally, in the revised manuscript, we have stated the between-study variance (tau, τ^2) and the 95% Prediction Interval (PI) alongside the pooled estimate. However, important differences were observed in the estimation of between-study heterogeneity (τ^2) among the models. The Der Simonian–Laird method results a substantially lower heterogeneity ($\tau^2 = 53.92$) compared with REML ($\tau^2 = 146.21$) and Sidik–Jonkman ($\tau^2 = 145.81$). These findings could be due to the well-documented methodological limitations of the DL estimator, which tends to underestimate τ^2 in the presence of extreme heterogeneity. For this reason, we are fully convinced that the REML approach provides a more accurate and less biased estimate of virologic suppression, and we have accordingly applied it consistently across the revised manuscript, from the abstract to the conclusion section of the manuscript.

Reviewer #4: 3) Explaining heterogeneity rather than averaging it away

Your region/country subgrouping is helpful. If feasible, consider a compact set of clinically/programmatically meaningful moderators, ART era (EFV vs DTG rollout) and maternal age (15-24 vs ≥25 years), via simple stratified analyses or a brief meta-regression.

If further analyses are not practical, a short justification for the current scope plus a more cautious interpretation (with the PI) is acceptable.

Authors' response: Thank you for your suggestion. We agree with the reviewer that explaining the sources of heterogeneity is more informative than simply reporting the overall estimate of VS. Even though we encountered to extract consistently categorized data from the primary studies, we try our utmost effort to improve the quality of this manuscript. As per the comment, we have conducted stratified analyses to explore clinically important moderators. This includes regions of Africa, Country, and ART era (comparing the DTG-based and non-DTG based). These results are stated in the result and discussion section (Table 2: Meta-regression).

Reviewer #4: 4) Small-study effects / publication bias

Under heavy heterogeneity, Egger is suboptimal for single-arm proportions. Please consider Peters/Harbord tests where the number of studies permits and temper funnel-plot interpretations accordingly; if underpowered, state this explicitly.

Authors' response: We thank the reviewer for this valuable methodological suggestion. We considered using Peters and Harbord tests for assessing publication bias, however we opted to retain Egger's test. Peters and Harbord tests are generally preferred for binary outcome variables; our study focuses on prevalence estimate. For this reason, we retained Egger's test, which remains a robust approach for assessing publication bias when the number of studies is sufficient (>30 studies).

Reviewer #4: 5) Unit of analysis & potential double counting

Where a single dataset contributes multiple effects (e.g., pregnant vs intrapartum vs postpartum; or multiple VL thresholds), please explain how you avoided double counting, for example, by pre-specifying one time-point, aggregating within-study effects, or using a multilevel model.

Authors' response: Thank you very much for this crucial point. We considered virologic suppression (VS) as independent across pregnancy, intrapartum, and postpartum (breastfeeding),

allowing each effect to be examined independently, in order to mitigate the possibility of double counting. To ensure that each study contributed fairly without inflating the outcome, we combined these time-specific effects to determine the overall pooled estimate of VS for these groups of population.

Reviewer's comment: 6) Risk of bias & transparency (search, RoB, data/code**)**

Include a per-study JBI risk-of-bias matrix in the supplement, justify any scoring thresholds, and add a sensitivity analysis excluding higher-risk studies. For search reproducibility, provide full search strings, last search date, and the deduplication procedure; also clarify whether Embase/Scopus and Africa-focused sources (e.g., AIM/AJOL) were searched or provide a documented rationale for their exclusion.

For reproducibility, make the extraction sheet (study-level events/total and moderators) fully available per PLOS policy (as SI or in a DOI-minted repository). Sharing analysis code (Stata/R) is strongly encouraged; at minimum, include sufficient model/command detail for re-analysis.

Authors' response: Authors' response: Thank you once again. We have stated the use of PubMed as a main database for literature search. PubMed is a widely recognized biomedical database for accessing indexed published articles. To ensure comprehensive searching and bridge potential gap for using PubMed, we augmented our search by systematically exploring Google Scholar to identify both published and unpublished studies, and we also used ScienceDirect to retrieve relevant articles from Elsevier-indexed journals. We have uploaded relevant data as supplementary file, including search strategy, data extraction sheet, and Stata file and code for possible re-analysis as well as journal's data availability guideline.

Authors' response: Thank you very much for this insightful suggestion.

Reviewer's comment: 7) Determinants / associated factors (AORs)

Please specify the eligibility criteria for studies entering the determinants analysis and ensure alignment of reference categories and reasonable consistency of adjustment sets before pooling on the log-OR scale. If adjustment sets differ substantially or heterogeneity is high with wide CIs, consider a narrative synthesis or meta-regression rather than a pooled AOR. Clarify why

only a subset of studies contributed and whether others were excluded for design/measure reasons.

Authors' response: Thank you dear reviewer. As a result, we have made the eligibility requirements for research that are part of the determinants analysis clear and making sure that reference categories are consistently aligned across studies. We examined adjustment sets closely and fixed any discrepancies. We used systematic synthesis for VS variables in cases with significant variations in adjustments or high variability with wide confidence intervals. We have made substantial revision in the result and discussion sections.

Reviewer's comment: Minor comments

Reviewer #4: • Move very long descriptive tables to the supplement and keep concise summary tables in the main text.

Authors' response: We sincerely appreciate your constructive feedback and valuable suggestions aimed at improving the quality of our manuscript. Following your suggestion, we have made the following changes: Table 1 (formerly describing Table 1: Descriptive summary of studies included in systematic review of virologic suppression among HIV-positive pregnant and lactating women receiving ART in Africa) has been moved to the Supplementary Material as Table S1 file.

Reviewer #4: • Include the 95% prediction interval in the Abstract/Results so readers immediately see expected variability across settings.

Authors' response: We agree that the 95% prediction interval (PI) provides essential context for interpreting the pooled prevalence in the presence of extreme heterogeneity. As requested, we have added the 95% PI to both the Abstract and the Results section of the manuscript.

Reviewer #4: • Define acronyms at first use (VS/VLS, PMTCT, DTG/EFV) and streamline a few long sentences with a light language edit.

Authors' response: We thank the reviewer for this helpful suggestion to improve the manuscript's readability. We have conducted a thorough editorial review and implemented to define acronyms at first use and concise long sentences.

Reviewer #4: • Proofread tables for numerical slips (e.g., any CI exceeding 100%) and ensure consistent terminology.

Authors' response: Response: We thank the reviewer for inviting us to re-check for typos errors. We have conducted a recheck to all tables and figures to ensure accuracy and consistency. All proportions and intervals have been corrected and rounded consistently as well as consistent use terminologies in the main text.

Reviewer #4: • Generalizability: where certain sub-analyses rely heavily on national program data, please comment on applicability to smaller or non-program settings.

Authors' response: Response: We thank the reviewer for this critical point regarding the generalizability of our findings. We acknowledge that our meta-analysis includes several large-scale studies derived from national program data, which reflect real-world implementation but may differ from smaller, controlled clinical settings.

***Reviewer #4*:** • Briefly cite the PROSPERO registration (ID) and ensure the PRISMA flow diagram is referenced in the main text for reproducibility.

Authors' response: We have ensured that the manuscript adheres to the highest standards of transparency and reproducibility. We have cited PROSPERO registration (ID) in the method section and we have reference the PRISMA flow diagram in the result section.

Reviewer #4: Recommendation: Major Revision.

The manuscript is valuable; the requested clarifications, outcome harmonization, robust proportion modeling, focused heterogeneity exploration (including ART era and age), and transparent materials (search/RoB/extraction file and, ideally, code), will make the conclusions more reliable and reproducible.

Authors' response: Thank you in advanced for your scientific and insightful comment. We appreciate the acknowledgement of the manuscript's worth. In response to these recommendations, we have made important modifications to enhance clarity, establish uniform outcomes definitions, utilize more rigorous proportion modeling approaches, and conduct targeted investigations of variability, particularly by ART period and age. We have also increased transparency by giving thorough search strategy, risk-of-bias assessments, data extraction

materials, and Stata analysis code. We believe these changes significantly improve the validity, reproducibility, and clarity of our findings.

Reviewer #5: This manuscript presents a systematic review and meta-analysis assessing the pooled prevalence of virologic suppression (VS) among HIV-positive pregnant and lactating women receiving antiretroviral therapy (ART) in Africa. The authors included 55 studies encompassing 304,883 participants across various African regions. The study followed PRISMA guidelines and used robust statistical methods, including a random-effects model, subgroup analyses, and heterogeneity assessment.

The topic is highly relevant, particularly in the context of achieving the global UNAIDS 95–95–95 targets and improving maternal and child health outcomes in sub-Saharan Africa. The manuscript contributes meaningful evidence regarding ART adherence and determinants of viral suppression in this key population group. This manuscript has major strengths such as relevance, scope and data size, systematic approach, robust analysis, policy value.

Authors' response: We truly appreciate Reviewer #5's careful and thorough assessment of our submission. We value the acknowledgement of the study's importance, scope, and rigorous methodology, as well as its contribution to our knowledge of virologic suppression in African pregnant and lactating women receiving antiretroviral therapy. We particularly grateful for the recognition of the manuscript's relevance to policy and linkage with initiatives to improve mother and child health outcomes and achieve the UNAIDS 95–95–95 targets. We really appreciate your positive remarks, which have inspired us to improve the updated manuscript's impact and clarity.

Reviewer #5: Minor Editorial Comments:

- In Abstract section, replace "remains low" with quantitative context ("approximately 81%, below global target of 95%").

Authors' response: As suggested, we have revised the Abstract to replace the phrase "remains low" with specific data.

Reviewer #5*:* Conduct meta-regression and sensitivity analysis by study design, ART regimen, and publication year.

Authors' response: In accordance with the reviewer's suggestion, we have conducted both meta-regression and sensitivity analyses to systematically explore the sources of heterogeneity.

Reviewer #5: ⎫ Provide more discussion on possible small-study or regional publication bias.

Authors' response: We thank the reviewer for your suggestion; we have expanded the discussion and acknowledge the regional concentration of studies as a limitation.

Reviewer #5: ⎫ Include qualitative evidence or secondary data review to contextualize findings.

Authors' response: Thank you. In our revised manuscript, we have carried out a systematic synthesis and integrated evidence from qualitative studies in Africa. We incorporated structural and sociocultural barriers for VS.

Reviewer #5: ⎫ Ensure all forest plots (Figures 2–8) have consistent formatting and readable axis labels.

Authors' response: We thank the reviewer for this attention to detail. We revised the formatting of forest plots (figures) to ensure a high-quality.

Reviewer #5: ⎫ For References, several citations lack full journal details or DOI (e.g., refs. 70, 77). Please verify all references conform to PLOS ONE style. Conduct sensitivity analysis by publication period (pre/post-2020).

Authors' response: We appreciate Reviewer #5's insightful and useful recommendations. To ensure that references completely adhere to PLOS ONE formatting criteria, including the completion of any missing publication details and DOIs (such as references 70 and 77), we have carefully examined and revised all references. A sensitivity analysis stratified by publication period (pre-2020 vs. post-2020) was also carried out, and the results are included in the updated manuscript.

Reviewer #5: ⎫ Minor edits needed for flow in Introduction and Discussion to grammar.

Authors' response: We thank the reviewer for this suggestion. We have conducted a substantial revision of the Introduction and Discussion sections to improve the grammatical accuracy and overall narrative flow.

Reviewer #5: Closing Statement:

This manuscript is a well-executed, timely, and policy-relevant meta-analysis that adds significant value to the understanding of virologic suppression among HIV-positive women in Africa.
With minor clarifications on methodology, expanded heterogeneity analysis, and slight editorial adjustments, the paper will meet PLOS ONE’s publication standards and provide actionable insights for maternal HIV prevention and control programs across the continent.

Authors’ response: Overall, we have expanded the heterogeneity analyses, clarified the methodology details, and made the required editorial changes. In our opinion, these revisions ensure PLOS ONE's publishing requirements and enhance its applicability for improving maternal HIV prevention and control initiatives in Africa.

Reviewer #6: Authors section: I suggest you only use Names, instead of Initials Delete, let’s only have correspondent Authors. The email addresses for each will be put in the PLOS ONE form as you submit the manuscript.

Add **Problem Statement**, and **Objectives**. Research questions without Problem statement are hanging. I suggest you add

1. Problem statement
2. Significance of this Systematic Review
   Refer to the attached manuscript with track changes

Authors’ response: Thank you very much for these constructive and helpful comments. We have revised the Authors’ section as suggested. Full author names are now used instead of initials. In addition, only the corresponding author is indicated in the manuscript, and individual author email addresses have been removed from the main text.

According to the comment, we have added the significance of the study to clearly show the importance, relevance, and potential contribution of this review to policy, practice, and future research. In the “Introduction section”, we have clearly described the background, triggers, existing gaps in the literature, and the rationale for conducting this systematic review.

Reviewer #7: Page 139-143: The problem needs to be further refined, especially for research issues concerning viral load viruses. Ethical clearance issues also sometimes become obstacles

and the lack of study in HIV research. page 168 -175: Please clarify the source of the data taken, because in the background of the problem, you describe something that highlights the lack of research in the field regarding this topic.

Authors' response: We have refined the problem statement to more clearly articulate the key research gaps related to viral load suppression among HIV-positive pregnant and lactating women. Of course, there are ethical issues and require to adhere ethical considerations for HIV patients to undergo primary studies. This systematic review was conducted in accordance with the PRISMA guidelines, ensuring transparency, methodological rigor, and ethical reporting standards.

Reviewer #7: page 194-200: Could you provide more details on the research method conducted with a pooled prevalence, including which data was used and the operational definitions applied?

Authors' response: We have revised the Methods section to provide a clearer description of the data sources used to estimate the pooled prevalence, the inclusion and extraction procedures, and the statistical analysis. We have also added explicit operational definitions for outcome variable (Virologic suppression) to enhance clarity and reader friendly.

Reviewer #7: page 211- 220: is it only using this data to determine the pooled prevalence?

Authors' response: Thank you very much. We confirm that the pooled prevalence was estimated using data extracted from the included studies. Standard errors of prevalence were calculated and, where appropriate, logit-transformed prevalence estimates were applied to stabilize variance before pooling. Then, data exported to Stata 17 for further statistical analysis. A random-effects model with Restricted maximum–likelihood (REML) was used to determine the pooled prevalence of virologic suppression. We performed some important changes in the Methods section.

Reviewer #7: page 231-233: Why you leave-one-out sensitivity analysis?

Authors' response: Thank you for the query. A leave-one-out sensitivity analysis was conducted to assess the influence of each individual study on the pooled estimate and to evaluate the robustness of the findings in the presence of heterogeneity. This analysis helped determine whether any single study disproportionately affected the overall results. Methods and Results

sections.

Reviewer #7: page 255 - 256: can you more explain with this result?

Authors' response: We thank the reviewer for requesting clarification. The high level of heterogeneity (I^2 = 99.57%) indicates substantial variability in effect estimates across the included studies beyond probability. For this reason, a random-effects model was applied to account for between-study variability and to provide a more conservative pooled estimate. Accordingly, we have revised in the main text.

Reviewer #7: page 351 -356: This explanation must be further clarified and supported by literature that underpins this statement.

Authors' response: We appreciate the reviewer's idea. Appropriate references are now provided to support the scientific justification.

Reviewer #7: page 417: Although this research is a systematic review and meta-analysis using secondary data, researchers must still consider intellectual property rights and proper attribution for the data used.

Authors' response: Absolutely. We have ensured that all data sources are appropriately acknowledged and attributed while respecting intellectual property rights. References and acknowledgments have been thoroughly examined and updated in the manuscript to provide proper credit to the original works.

Reviewer #8: Given the high prevalence of HIV-positive women in African countries, it is important to understand virological suppression in pregnant and breastfeeding women. Conducting this systematic review provides insight into the current status of this population.

Authors' response: Thank you for providing general and specific comments and suggestions.

Reviewer #8: The authors have considered the necessary methodological criteria and clearly described the process for developing this systematic review. However, it is not entirely clear how they analyzed and interpreted the determinants of virological suppression and associated variables, nor how they addressed potential confounders. It would be helpful to detail these aspects in the review manuscript.

Authors' response: We thank Reviewer #8 for this constructive comment. In response, we have modified the Results and Discussion sections to further explain how factors of virologic suppression and associated variables were analyzed using meta-analysis and systematic synthesis.

Reviewer #8: When evaluating individual studies, why was the Critical Appraisal Checklist considered instead of a validated tool for assessing the methodological quality of studies? Potential biases arising from the study assessment are not addressed in the limitations section of the review. Please comment.

Authors' response: We thank the reviewer for this important comment. We employed the JBI Critical Appraisal Checklist, a widely recognized and comprehensive tool designed to evaluate the methodological quality of diverse study designs typically included in systematic reviews. Given its rigor and standardized approach, we believe that potential biases arising from study assessment have been sufficiently addressed, and additional assessment of such biases is not necessary.

Reviewer #9: Thank you for doing research on his interesting topic. It is essential to emphasize the importance of implementing targeted interventions for young HIV-positive women, including the disclosure of HIV status, initiation of first-line antiretroviral therapy (ART) regimens, and promotion of ART adherence.

Authors' response: We sincerely thank the reviewer for highlighting this important point. Accordingly, we have emphasized the significance of targeted interventions for young HIV-positive women in the revised Discussion and Conclusion sections, including the roles of HIV status disclosure, timely initiation of first-line ART, and strategies to promote adherence. This addition strengthens the practical relevance and public health implications of our findings.

Reviewer #10: The manuscript (PONE-D-25-49195) addresses a critical public health issue concerning HIV viral suppression among pregnant and lactating women on antiretroviral therapy (ART) in Africa. The topic aligns well with global health and HIV epidemiology. The study is comprehensive but requires significant improvements in methodological transparency, reporting, and interpretation. I have the following concerns which if addressed will improve the study.

Authors' response: We sincerely thank the reviewer for recognizing the public health importance of our study and its relevance to HIV epidemiology in Africa. We appreciate your constructive feedback regarding methodological transparency, reporting, and interpretation. In response, we have carefully revised the manuscript to clarify the methodology, improve reporting of results, and strengthen the interpretation in line with the study objectives and current literature. Your insights have greatly helped enhance the clarity, rigor, and overall quality of our work.

Reviewer #10: 1. Prospero has CRD420251077063, registered as "Knowledge of mother-to-child transmission of HIV and its prevention among reproductive-age women in Africa: a systematic and meta-analysis 2025".

- Register your review again in Prospero and state the ID correctly.

Authors' response: We sincerely thank the reviewer for pointing this out. We apologize for the inadvertent error in reporting the PROSPERO ID. The correct study protocol for this systematic review and meta-analysis has been registered on PROSPERO (**ID**: **CRD420251186121**, **https://www.crd.york.ac.uk/PROSPERO/view/CRD420251186121**). The manuscript has been updated to reflect the correct registration information.

Reviewer #10: 2. Under introduction (line 144 – 153), the research question is a repetition from the goal of the study. Merge the goal and research question to avoid repetition.

Authors' response: Thank you in advance. The Introduction has been revised to merge the study goal and research question, eliminating redundancy while maintaining coherence. This change improves the flow and readability of the manuscript.

Reviewer #10: 3. Remove (line 168- 175) and present as part of your supplementary file S2 file.

Authors' response: We sincerely appreciate the reviewer's comment. We have clarified that the keywords listed were used to build search strategies across multiple databases and other search engines. To ensure transparency, the full search strategy is provided in **Supplementary File S2.** Therefore, we wish to retain the current explanation in the manuscript while attached the detailed search strategy as supplementary file.

Reviewer #10: 4. Include a complete PRISMA 2020 checklist and flow diagram under the results as part of the manuscript not part of list of figures (Figure 1). Draw it nicely or get it the flow diagram rom online, present it well in the manuscript, with well labelled figure legend.

Authors' response: Accordingly, we have incorporated a PRISMA 2020 checklist and a well-presented flow diagram directly within the Results section of the manuscript. The list figures are attached separately as a tiff figure in line with the journal guideline. The flow diagram has been clearly formatted with a detailed, well-labeled legend to clearly illustrate the study selection process and referenced in the result section.

Reviewer #10: 5. For the risk of bias assessment, present the JBI quality assessment results in a summary table or figure.

Authors' response: We thank the reviewer for this valuable suggestion. For this reason, we have added a summary table presenting the JBI quality assessment results for all included studies. This addition improves transparency and allows readers to easily interpret the methodological quality of the studies included in our review (S1 file Table 1).

Reviewer #10: 6. Table 1 should be added to the results section with a well described table legend under the table, stating what criteria or reference was used for the risk of bias.

7. Table 2 should be added to the results section, not as part of list of tables, then with a legend describing or stating what was used to achieve the sub-group analysis.

Authors' response: Thank you. Table 1 has been added to the Results section, and a clear legend has been included below the table specifying the criteria and reference used for the risk of bias assessment (JBI score) in the method section. Additionally, Table 2 also added to the Results section, with a detailed legend explaining the criteria and data used to perform the subgroup analyses.

Reviewer #10: 8. Figure 1-8 should be presented as part of the results with a well labelled figure legend, stating how it was achieved.

Authors' response: Thank you dear reviewer for this suggestion. We have tried to give clear and detailed legend describing the methods and data used to generate the figures.

Reviewer #10: 9. Consider sources of heterogeneity and use meta-regression by study year, region/country, ART regimen and viral load threshold to improve the robustness of the findings.

Authors' response: We thank you the reviewer for this insightful suggestion. We have carefully considered potential sources of heterogeneity, including publication year, region/country, ART regimen, and study design. Meta-regression analyses were conducted based on the above variables. The results have been included in the revised manuscript to provide a stronger understanding of factors contributing to heterogeneity.

Reviewer #10: 10. Include in the discussion, a more clearly link between the UNAIDS 95-95-95 targets, national PMTCT programs and strategies to address low suppression in younger women. Overall, this review is timely and relevant. Addressing the above comments will substantially enhance its scientific rigor, methodological transparency, and readability.

Authors' response: We truly thank the reviewer for this constructive feedback. As per the reviewer comment, we have revised the Discussion to explicitly link our findings to the UNAIDS 95-95-95 targets and national PMTCT programs, highlighting strategies to improve viral suppression among younger women.

Reviewer #11: Overall, this is good research, this review takes a deep dive into how well HIV-positive pregnant and breastfeeding women in Africa are responding to antiretroviral therapy, specifically looking at how many are achieving virologic suppression. The study is methodologically solid, using validated tools and statistical models to analyze the data. It also identifies key factors that influence outcomes, like age, relationship status, ART regimen, HIV status disclosure, and adherence. Younger women and those with poor adherence were less likely to achieve suppression, pointing to the need for youth-friendly care and stronger support systems. While the results are promising, the high variability across studies and regions suggests more targeted interventions and further research are needed to close the gap and reduce mother-to-child transmission.

Authors' response: To sum up, the authors are grateful for Reviewer #11 detailed and insightful assessment. We are grateful that the study's methodological rigor, thorough analysis, and identification of important factors influencing virologic suppression in HIV-positive

pregnant and breastfeeding women in Africa were acknowledged. We particularly appreciate the reviewer's observations on the challenges younger women and those with poor adherence encounter, as well as the necessity of youth-friendly care and targeted interventions. We agreed that these findings emphasize the significance of specific actions and further study to close gaps and lower mother-to-child HIV transmission, even though we acknowledge the high heterogeneity among studies in Africa.

Reviewer #12: 1. General Assessment

This manuscript presents a systematic review and meta-analysis evaluating the prevalence of virologic suppression and its associated factors among HIV-positive pregnant and lactating women receiving ART in Africa. The study is timely, methodologically sound, and addresses a critical gap in maternal HIV care across the continent. The authors adhered to PRISMA guidelines, registered their protocol on PROSPERO, and employed appropriate statistical techniques including random-effects modeling, subgroup analysis, and sensitivity testing. The manuscript is generally well-written and organized, with clear objectives and relevant findings. However, several areas require clarification or refinement to meet PLOS ONE’s standards for transparency, reproducibility, and scientific rigor.

Authors’ response: We truly valued Reviewer #12's extensive and informative review. We are grateful that the study's relevance, rigor of methodology, adherence to PRISMA guidelines, PROSPERO registration, and application of reliable statistical techniques were acknowledged. To improve transparency, consistency, and scientific rigor, we have addressed the given suggestions and improvements. As a result, the revised manuscript meets PLOS ONE's standards.

Reviewer #12 2. Section-by-Section Evaluation

Methods and Materials

Areas for Improvement:

1. Search Strategy: The Boolean search string is extensive but lacks structure. Present full search strategies (e.g., for PubMed) in a supplementary file.

Authors' response: Thank you very much for this important comment. The main text outlines the keywords used to construct search strategies across various databases and search engines. Additionally, the complete, structured search strategy for each database, including PubMed, has been provided as a **supplementary file (S2 file)** to ensure full transparency and reproducibility.

Reviewer #12 2. Database Justification: Justify inclusion of Google Scholar due to its non-standard indexing.

Authors' response: We thank the reviewer for this important comment. Google Scholar was included as a supplementary search engine to capture all relevant articles, particularly unpublished (grey) literature, thereby helping to minimize publication bias. Its use complements standard databases and ensures a more comprehensive and inclusive evidence base for the review.

Reviewer #12 3. Quality Threshold: Rephrase "excellent quality" for studies scoring ≥5 on JBI to "acceptable quality."

Authors' response: We thank the reviewer for this suggestion. We have revised the manuscript to replace "excellent quality" with "acceptable quality" for studies scoring ≥5 on the JBI checklist.

Reviewer #12: 4. Outcome Definitions: Clarify whether all studies used the same VL threshold (≤1000 copies/mL).

Authors' response: We thank the reviewer for this comment. To ensure consistency, we included only studies that used the same viral load threshold (≤1000 copies/mL). The Methods section has been revised and updated accordingly to reflect this.

Reviewer #12: 5. Statistical Rationale: Justify use of Der Simonian and Laird's method over alternatives like Hartung-Knapp.

Authors' response: We thank the reviewer for this insightful comment. We compared and contrast the pooled estimates of virologic suppression using three random-effects meta-analysis estimators: Der Simonian–Laird (DL), Sidik–Jonkman (SJ), and restricted maximum likelihood (REML). Finally, **we convinced to use a random-effects model with REML because it is a**

widely recognized and commonly applied method for pooling prevalence estimates in meta-analyses with high heterogeneity. The REML approach provides a more accurate and less biased estimate of virologic suppression. However, Der Simonian–Laird (DL) underestimates the between-study heterogeneity (τ^2). We have accordingly applied REML model consistently across the revised manuscript, from the beginning to the conclusion section of the manuscript.

Reviewer #12: 6. Publication Bias Tests: Clarify whether Egger's and Begg's tests were applied to both prevalence and AORs.

Authors' response: We thank the reviewer for this comment. The Egger's and Begg's tests were applied to assess publication bias for the pooled prevalence estimates. For the pooled AORs, the number of included studies was limited, making these tests less reliable.

Reviewer #12: Results

Areas for Improvement:

1. PRISMA Flow: Include a complete PRISMA diagram and a table of excluded studies with reasons.

Authors' response: Thank you for the comment. We have included a complete PRISMA 2020 flow diagram for study selection in the Results section.

Reviewer #12: 2. Heterogeneity: $I^2 = 99.57\%$ is extremely high. Consider meta-regression or deeper exploration of sources.

Authors' response: Dear reviewer thanks you for this insightful comment. Given that our review included diverse studies across Africa, high heterogeneity was anticipated. Accordingly, we explored potential sources of heterogeneity using subgroup and sensitivity analyses. Following the reviewer's recommendation, we also conducted meta-regression analyses to further investigate and account for possible sources of heterogeneity, and the results have been included in the revised manuscript.

Reviewer #12: 3. Subgroup Analysis: Report statistical significance of subgroup differences and I^2 values per subgroup.

Authors' response: We have reported in the main text.

Reviewer #12: 4. Associated Factors: Provide AORs and CIs for all discussed predictors. Clarify model type used for pooling.

Authors' response: Thank you. The statistical significance of subgroup differences and I^2 values for each subgroup have now been reported and are presented in Table 2 of the revised manuscript.

Reviewer #12: 5. Figures: Ensure all figures (e.g., funnel plot, forest plots) are clearly labeled and referenced.

Authors' response: We thank the reviewer for this comment. We have ensured that all figures, including funnel and forest plots, are clearly labeled and properly referenced in the manuscript.

Reviewer #12: Discussion, Strengths & Limitations, Conclusion

Areas for Improvement:

1. Interpretation: Discuss clinical significance of the 14% gap from the 95% target.

Authors' response: Dear reviewer, thank you very much for this valuable comment. We have made a notable revision in the discussion part that show the implication of the 14% gap behind the USAIDS the 95% target in PMTCT.

Reviewer #12: 2. Causal Language: Soften causal claims to reflect observational nature (e.g., "associated with").

Authors' response: We thank the reviewer for this important comment. We have revised the manuscript to soften causal language throughout, using terms such as "associated with" to accurately reflect the observational nature of the included studies.

Reviewer #12: 3. Policy Framing: Reference WHO/UNAIDS frameworks when recommending interventions.

Authors' response: We have acknowledged the WHO/UNAIDS frameworks for recommended interventions to achieve the 95% VS target.

Reviewer #12: 4. Limitations: Clarify geographic skew and discuss potential impact of publication bias despite non-significant tests.

Authors' response: The manuscript has been revised to present actionable interventions aligned with WHO and UNAIDS frameworks, ensuring that our policy recommendations are evidence-based international guidelines.

Reviewer #12: 5. Conclusion: Separate findings from recommendations for clarity. Specify which health system components need strengthening.

Reviewer #12:
3. Minor Editorial Suggestions
• Correct grammar and phrasing (e.g., "All searching databases" → "All searched databases").
• Ensure consistent terminology: "virologic suppression" vs. "viral load suppression."
• Fix typos (e.g., "studies is required" → "studies are required").

Authors' response: We thank the reviewer for these helpful editorial suggestions. We have carefully corrected grammar and phrasing, ensured consistent terminology throughout the manuscript, and addressed identified typos to enhance clarity and readability.
