# Supplementary material for: Virologic suppression among HIV-positive pregnant and lactating women receiving antiretroviral therapy in Africa: A systematic review and meta-analysis
Source: PLoS One. 2026 Apr 13;21(4):e0346045. doi: 10.1371/journal.pone.0346045 (PMC13075719; doi:10.1371/journal.pone.0346045)
Supplement: Attachment — Submitted filename: PONE-D-25-49195_Reviewer JB 26012026.pdf [file pone.0346045.s015.pdf]

PLOS One

Virologic suppression among HIV-positive pregnant and lactating women receiving antiretroviral therapy in Africa: A systematic review and meta-analysis

--Manuscript Draft--

**Manuscript Number:**	PONE-D-25-49195R1
**Article Type:**	Research Article
**Full Title:**	Virologic suppression among HIV-positive pregnant and lactating women receiving antiretroviral therapy in Africa: A systematic review and meta-analysis
**Short Title:**	Virologic suppression among HIV-positive women in Africa: systematic review and meta-analysis
**Corresponding Author:**	Berihun Agegn Mengistie<br>University of Gondar College of Medicine and Health Sciences<br>Gondar, ETHIOPIA
**Keywords:**	Africa; antiretroviral therapy; HIV; pregnant women; lactating women; virologic suppression
**Abstract:**	Background: The elimination of mother-to-child transmission of human immunodeficiency virus (HIV) is a key global public health priority. To stop the vertical transmission of HIV and lower the risk of maternal morbidity and mortality, it is important to achieve viral load suppression. While many African countries have adopted the global 95-95-95 targets, comprehensive data on virologic suppression among pregnant and lactating mothers across the continent remains limited. The objective of this systematic review and meta-analysis was to determine the pooled estimate of virologic suppression and to examine the factors associated with it among HIV-positive pregnant and lactating women on antiretroviral therapy in Africa.<br>Methods: This study followed the Preferred Reporting Items for Systematic Reviews and Meta-Analyses (PRISMA) guidelines. The study protocol was registered in the International Prospective Register of Systematic Reviews (PROSPERO; CRD420251186121). We carried out a thorough systematic review by examining PubMed, ScienceDirect, Hinari, and Google Scholar for relevant studies. Data from the studies were retrieved using an Excel sheet and analyzed with STATA version 17. The Joanna Briggs Institute appraisal tool was used to evaluate the methodological quality of studies. A random-effects model with restricted maximum likelihood (REML) was applied to determine the pooled prevalence of virologic suppression (viral load threshold ≤1000 copies/ml) among pregnant and lactating mothers in Africa. A funnel plot and the Egger's test were used to investigate publication bias. Statistical heterogeneity was assessed using the I^2 statistic and Cochrane's Q test.<br>Results: A total of 55 eligible studies, comprising 304,883 participants, were included in the quantitative meta-analysis. Accordingly, the overall prevalence of virologic suppression among HIV-positive pregnant and breastfeeding women in Africa was 80.86% (95% CI: 77.63%, 84.09%, I2 = 99.84%). In contrast, the pooled estimate for achieving an undetectable viral load was substantially lower, at 60.92% (95% CI: 52.46%, 69.39%; I^2 = 99.91%). Virologic suppression was significantly associated with women's age (15-24 years) (AOR = 0.49; 95% CI: 0.32-0.77), disclosure of HIV status to a partner (AOR = 1.66; 95% CI: 1.31-2.11), first-line antiretroviral therapy regimen (AOR = 6.53; 95% CI: 1.93-22.06), and good antiretroviral drug adherence (AOR = 3.61; 95% CI: 1.18-11.02). In addition, other socio-demographic variables, higher educational level, being married/cohabitant, urban residency, healthcare utilization (time of ANC booking, time of ART initiation, duration of ART), fear of stigma, distance to health facility, shortage of health professionals, ART drug stock-out, and lack of HIV care commodities were significantly associated with virologic suppression among HIV-positive pregnant and lactating women in Africa.<br>Conclusion: The pooled estimate of virologic suppression among HIV-positive pregnant and breastfeeding women in Africa was approximately 81%, below the global target of 95% virological suppression. This emphasizes the necessity of targeted strategies for younger HIV-positive women, disclosing HIV status, initiating first-line antiretroviral regimens, and promoting antiretroviral treatment adherence. Upgrading health care systems to enable regular viral load monitoring, as well as addressing socio-demographic and antiretroviral therapy-related variables, are vital steps towards

Powered by Editorial Manager® and ProduXion Manager® from Aries Systems Corporation

	attaining and sustaining VS in these groups of population, ultimately assisting in achieving elimination of MTCT of HIV.
Order of Authors:	Berihun Agegn Mengistie
	Getie Mihret Aragaw
	Gebrye Gizaw Mulatu
	Kindu Yinges Wondie
	Alemneh Tadesse Kassie
	Alemken Eyayu Abuhay
	Wondimnew Mersha Biset
	Moges Tesfa Tsega
	Abay Eyayu Asrie
	Tazeb Alemu Anteneh
	Habtu Kifle Negash
	Eshet Gebrie
	Nuhamin Tesfa Tsega
Response to Reviewers:	Dear Editorial Team,<br><br>Thank you for timely feedback and carefully reviewing our manuscript, "Virologic suppression among HIV-positive pregnant and lactating women receiving antiretroviral therapy in Africa: A systematic review and meta-analysis" (PONE-D-25-49195), and bringing to our attention the concerns about text overlap with previous articles.<br><br>We sincerely apologize for being late to revise our submitted manuscript. We are grateful to the academic editor for facilitating our submission and to the reviewers for their valuable scientific comments, expertise, and guidance, which were instrumental in improving the overall quality of our manuscript. The authors diligently and meticulously address all the comments and recommendations provided by all reviewers and the academic editor. As a result, we believe that the manuscript substantially improved in terms of grammar, punctuation, coherence, clarity, methodological rigor, transparency, and reproducibility. We have submitted the revised manuscript alongside the manuscript with track changes, "Response to Reviewers," and other relevant supplementary files.<br>We look forward to your timely feedback for our revised manuscript.<br><br>Thank you for your time and ongoing support.
Additional Information:	
Question	Response
**Financial Disclosure**<br><br>Enter a financial disclosure statement that describes the sources of funding for the work included in this submission. Review the submission guidelines for detailed requirements. View published research articles from *PLOS ONE* for specific examples.<br><br>This statement is required for submission and **will appear in the published article** if	The author(s) received no specific funding for this work.

Powered by Editorial Manager® and ProduXion Manager® from Aries Systems Corporation

the submission is accepted. Please make sure it is accurate.<br><br>**Funded studies**<br>Enter a statement with the following details:<br>• Initials of the authors who received each award<br>• Grant numbers awarded to each author<br>• The full name of each funder<br>• URL of each funder website<br>• Did the sponsors or funders play any role in the study design, data collection and analysis, decision to publish, or preparation of the manuscript?<br><br>Did you receive funding for this work?	
**Competing Interests**<br><br>Use the instructions below to enter a competing interest statement for this submission. On behalf of all authors, disclose any competing interests that could be perceived to bias this work—acknowledging all financial support and any other relevant financial or non-financial competing interests.<br><br>This statement is required for submission and **will appear in the published article** if the submission is accepted. Please make sure it is accurate and that any funding sources listed in your Funding Information later in the submission form are also declared in your Financial Disclosure statement.<br><br>View published research articles from *PLOS ONE* for specific examples.	The authors have declared that no competing interests exist.

**NO authors have competing interests**<br><br>Enter: *The authors have declared that no competing interests exist.*<br><br>**Authors with competing interests**<br><br>Enter competing interest details beginning with this statement:<br><br>*I have read the journal's policy and the authors of this manuscript have the following competing interests: [insert competing interests here]*<br><br>* typeset	
**Ethics Statement**<br><br>Enter an ethics statement for this submission. This statement is required if the study involved:<br><br>• Human participants<br>• Human specimens or tissue<br>• Vertebrate animals or cephalopods<br>• Vertebrate embryos or tissues<br>• Field research<br><br>Write "N/A" if the submission does not require an ethics statement.<br><br>General guidance is provided below. Consult the submission guidelines for detailed instructions. **Make sure that all information entered here is included in the Methods section of the manuscript.**	N/A

**Format for specific study types**<br><br>**Human Subject Research (involving human participants and/or tissue)**<br>• Give the name of the institutional review board or ethics committee that approved the study<br>• Include the approval number and/or a statement indicating approval of this research<br>• Indicate the form of consent obtained (written/oral) or the reason that consent was not obtained (e.g. the data were analyzed anonymously)<br><br>**Animal Research (involving vertebrate animals, embryos or tissues)**<br>• Provide the name of the Institutional Animal Care and Use Committee (IACUC) or other relevant ethics board that reviewed the study protocol, and indicate whether they approved this research or granted a formal waiver of ethical approval<br>• Include an approval number if one was obtained<br>• If the study involved *non-human primates*, add *additional details* about animal welfare and steps taken to ameliorate suffering<br>• If anesthesia, euthanasia, or any kind of animal sacrifice is part of the study, include briefly which substances and/or methods were applied<br><br>**Field Research**<br><br>Include the following details if this study involves the collection of plant, animal, or other materials from a natural setting:<br>• Field permit number<br>• Name of the institution or relevant body that granted permission	
**Data Availability**<br><br>Authors are required to make all data underlying the findings described fully available, without restriction, and from the time of publication. PLOS allows rare exceptions to address legal and ethical concerns. See the PLOS Data Policy and FAQ for detailed information.	Yes - all data are fully available without restriction

Powered by Editorial Manager® and ProduXion Manager® from Aries Systems Corporation

A Data Availability Statement describing where the data can be found is required at submission. Your answers to this question constitute the Data Availability Statement and **will be published in the article**, if accepted.<br><br>**Important:** Stating 'data available on request from the author' is not sufficient. If your data are only available upon request, select 'No' for the first question and explain your exceptional situation in the text box.<br><br>Do the authors confirm that all data underlying the findings described in their manuscript are fully available without restriction?	
**Describe where the data may be found in full sentences. If you are copying our sample text, replace any instances of XXX with the appropriate details.**<br><br>• If the data are **held or will be held in a public repository**, include URLs, accession numbers or DOIs. If this information will only be available after acceptance, indicate this by ticking the box below. For example: *All XXX files are available from the XXX database (accession number(s) XXX, XXX.).*<br>• If the data are all contained **within the manuscript and/or Supporting Information files**, enter the following: *All relevant data are within the manuscript and its Supporting Information files.*<br>• If neither of these applies but you are able to provide **details of access elsewhere**, with or without limitations, please do so. For example:<br><br>*Data cannot be shared publicly because of [XXX]. Data are available from the XXX Institutional Data Access / Ethics Committee (contact via XXX) for researchers who meet the criteria for access to confidential data.*<br><br>*The data underlying the results presented in the study are available from (include the name of the third party*	All relevant data are within the manuscript and its Supporting Information files.

and contact information or URL).<br>• This text is appropriate if the data are owned by a third party and authors do not have permission to share the data.<br><br>* typeset	
Additional data availability information:	

Powered by Editorial Manager® and ProduXion Manager® from Aries Systems Corporation

Manuscript Click here to access/download;Manuscript;Main Manuscript.docx

Virologic suppression among HIV-positive pregnant and lactating women receiving antiretroviral therapy in Africa: A systematic review and meta-analysis

Berihun Agegn Mengistie[1*], Getie Mihret Aragaw [1], Gebrye Gizaw Mulatu[2], Kindu Yinges Wondie[3], Alemneh Tadesse Kassie[3], Alemken Eyayu Abuhay[4], Wondimnew Mersha Biset[5], Moges Tesfa Tsega[6], Abay Eyayu Asrie[7], Tazeb Alemu Anteneh[3], Habtu Kifle Negash[8], Eshet Gebrie[9], Nuhamin Tesfa Tsega

[1] Department of General Midwifery, School of Midwifery, College of Medicine and Health Sciences, University of Gondar, Gondar, Ethiopia, [2] Department of Health Informatics, College of Medicine and Health Sciences, University of Gondar, Gondar, Ethiopia, [3] Department of Clinical Midwifery, School of Midwifery, College on Medicine and Health Sciences, University of Gondar, Gondar, Ethiopia, [4] University of Gondar Comprehensive Specialized Hospital, Gondar, Ethiopia, [5] Department of Anesthesiology, School of Medicine, Saint Paul's Hospital Millennium Medical College, Addis Ababa, Ethiopia, [6] Department of Internal Medicine, School of Medicine, College of Medicine and Health Sciences, University of Gondar, Gondar, Ethiopia, [7] Department of Obstetrics and Gynecology, College of Medicine and Health Sciences, University of Gondar, Gondar, Ethiopia, [8] Department of Human Anatomy, School of Medicine, College of Medicine and Health Sciences, University of Gondar, Gondar, Ethiopia, [9] Department of Clinical Chemistry, School of Biomedical and Laboratory Sciences, College of Medicine and Health Sciences, University of Gondar, P.O. Box 196, Gondar, Ethiopia, [10] Department of Women's and Family Health, School of Midwifery, College on Medicine and Health Sciences, University of Gondar, Gondar, Ethiopia

Corresponding author*: berihunagegn21@gmail.com

Abstract

Background: The elimination of mother-to-child transmission of human immunodeficiency virus (HIV) is a key global public health priority. To stop the vertical transmission of HIV and lower the risk of maternal morbidity and mortality, it is important to achieve viral load suppression. While many African countries have adopted the global 95-95-95 targets, comprehensive data on virologic suppression among pregnant and lactating mothers across the continent remains limited. The objective of this systematic review and meta-analysis was to determine the pooled estimate of virologic suppression and to examine the factors associated with it among HIV-positive pregnant and lactating women on antiretroviral therapy in Africa.

Methods: This study followed the Preferred Reporting Items for Systematic Reviews and Meta-Analyses (PRISMA) guidelines. The study protocol was registered in the International Prospective Register of Systematic Reviews (PROSPERO; CRD420251186121). We carried out a thorough systematic review by examining PubMed, ScienceDirect, Hinari, and Google Scholar for relevant studies. Data from the studies were retrieved using an Excel sheet and analyzed with STATA version 17. The Joanna Briggs Institute appraisal tool was used to evaluate the methodological quality of studies. A random-effects model with restricted maximum likelihood (REML) was applied to determine the pooled prevalence of virologic suppression (viral load threshold ≤1000 copies/ml) among pregnant and lactating mothers in Africa. A funnel plot and the Egger's test were used to investigate publication bias. Statistical heterogeneity was assessed using the I^2 statistic and Cochrane's Q test.

Results: A total of 55 eligible studies, comprising 304,883 participants, were included in the quantitative meta-analysis. Accordingly, the overall prevalence of virologic suppression among

HIV-positive pregnant and breastfeeding women in Africa was 80.86% (95% CI: 77.63%, 84.09%, I^2 = 99.84%). In contrast, the pooled estimate for achieving an undetectable viral load was substantially lower, at 60.92% (95% CI: 52.46%, 69.39%; I^2 = 99.91%). Virologic suppression was significantly associated with women's age (15-24 years) (AOR = 0.49; 95% CI: 0.32-0.77), disclosure of HIV status to a partner (AOR = 1.66; 95% CI: 1.31-2.11), first-line antiretroviral therapy regimen (AOR = 6.53; 95% CI: 1.93-22.06), and good antiretroviral drug adherence (AOR = 3.61; 95% CI: 1.18-11.02). In addition, other socio-demographic variables, higher educational level, being married/cohabitant, urban residency, healthcare utilization (time of ANC booking, time of ART initiation, duration of ART), fear of stigma, distance to health facility, shortage of health professionals, ART drug stock-out, and lack of HIV care commodities were significantly associated with virologic suppression among HIV-positive pregnant and lactating women in Africa.

Conclusion: The pooled estimate of virologic suppression among HIV-positive pregnant and breastfeeding women in Africa was approximately 81%, below the global target of 95% virological suppression. This emphasizes the necessity of targeted strategies for younger HIV-positive women, disclosing HIV status, initiating first-line antiretroviral regimens, and promoting antiretroviral treatment adherence. Upgrading health care systems to enable regular viral load monitoring, as well as addressing socio-demographic and antiretroviral therapy-related variables, are vital steps towards attaining and sustaining VS in these groups of population, ultimately assisting in achieving elimination of MTCT of HIV.

Keywords: Africa; Antiretroviral therapy; HIV; pregnant women; lactating women; virologic suppression

Introduction

Human immunodeficiency virus (HIV) is one of the world's most critical public health challenges, affecting millions of people [1, 2]. According to the Joint United Nations Programme on HIV/AIDS (UNAIDS) and World Health Organization (WHO) report, approximately 40.8 million people worldwide were infected with HIV, of which 21.0 million were women (15+ years); 1.3 million new HIV cases were reported, and 630,000 people died from AIDS-related illnesses at the end of 2024. In this report, nearly 1.4 million children aged below 15 years were living with HIV [1, 2]. Sub-Saharan African countries were responsible for 65% (an estimated 26.3 million) of the worldwide HIV burden [1, 2]. Women and girls of all ages contributed to 44% of all new HIV infections globally in 2023 [3].

The primary route of HIV infection in children is mother-to-child transmission (MTCT) of HIV during pregnancy, childbirth, and postpartum periods through breastfeeding [4]. It accounts for more than 90% of all newly acquired pediatric HIV infections worldwide [5]. Without intervention, the overall MTCT of HIV incidence ranges from 15% to 45% [6]. However, appropriate interventions during pregnancy, labor, and breastfeeding can lower the risk of perinatal HIV transmission to less than 2% in non-breastfeeding and 5% in breastfeeding populations, respectively [7, 8].

The World Health Organization (WHO) is committed to a global public health mission that focuses on expanding access to HIV testing, antiretroviral therapy (ART), and treatment monitoring, aiming to enhance clinical management of HIV, achieve sustained viral suppression, and lower HIV-related morbidity and mortality [9]. Maternal plasma HIV viral load (VL) status is the strongest determinant of HIV transmission from mother to child (MTCT) [10-12]. Nowadays, viral load assessment has become the main approach for monitoring HIV patients' clinical and virologic

outcome to ART [13]. HIV viral load is categorized into three main stages: unsuppressed (>1000 copies/mL), suppressed (detected but ≤1000 copies/mL), and undetectable (<50 copies/mL or not detected by the assay used) [9, 10, 14]. In 2021, the World Health Organization revised its HIV treatment monitoring algorithm to enhance support for people living with HIV in attaining viral suppression, with the ultimate aim of maintaining an undetectable viral load [9]. Achieving HIV viral suppression is crucial for enhancing individual health, preventing sexual transmission, and lowering the risk of perinatal transmission [9]. On the contrary, pregnant and lactating women who do not achieve virologic suppression (VS) are at a higher risk of HIV-related complications, death, and vertical transmission to their infants [10, 15].

Studies show that achieving and maintaining VS among pregnant and postpartum WLWH remains to be challenging, despite significant progress in the scaling up of ART via prevention of mother-to-child transmission (PMTCT) initiatives [16-18]. Several countries have been implementing the prevention of mother-to-child transmission of HIV (PMTCT) program packages [19-21]. It includes lifetime ART for pregnant and breastfeeding women, preventive antiretroviral (ARV) medications for HIV-exposed infants (HEIs), serial testing for HEIs, and prompt ART initiation for children who acquire HIV [4, 22]. Maintaining virologic suppression (≤1000 copies/ml) in HIV-positive pregnant and postpartum women is a global public health goal for improving maternal health and producing HIV-free children [12, 23].

Antiretroviral therapy (ART) as part of PMTCT is an important strategy to combat the global AIDS crisis and lower vertical transmission rates [24]. The WHO guidelines indicated that ART should be initiated in all pregnant and breastfeeding HIV-positive women, regardless of their VL, clinical stage, or CD4 cell count [15]. The fixed-dose combination (FDC) of tenofovir disoproxil fumarate (TDF), lamivudine (3TC), and dolutegravir (DTG) is the ideal first-line regimen for the treatment

of HIV infection in adults and adolescents, including pregnant and breastfeeding women [25]. Timely initiation and consistent adherence to ART are key predictors for achieving virologic suppression, specifically undetectable HIV viral load and preventing mother-to-child transmission of HIV, mitigating a range of clinical, virological, and immunological risks [15, 26]. Additionally, viral suppression enhances health outcomes for HIV-positive women, notably decreased illness, longer life expectancy, and better birth outcomes [27-29].

To tackle the continuing HIV crisis, the UNAIDS program is striding toward a 95-95-95 global target, ensuring that 95% of individuals living with HIV are aware of their HIV status, 95% of those diagnosed initiate lifelong ART, and at least 95% of patients beginning ART achieve virologic suppression [30-32]. However, in many of the SSA countries, virologic suppression is still behind the UNAIDS 95-95-95 targets [32-34].

In Africa, ART treatment failure or virologic failure among HIV-positive individuals continues to be a significant public health concern [35-37]. Similarly, the rate of virologic suppression among pregnant and lactating women is still unsatisfactory throughout the continent [16, 38]. In Africa, previous study findings on the proportion of virologic suppression among pregnant and breastfeeding women living with HIV revealed inconsistent and equivocal results that ranged from 29.1% to 97.8% [39, 40]. A body of evidence has shown that sociodemographic variables, adherence to ART, ART regimens, enhanced counseling, baseline viral load status, duration of ART, disclosing HIV status, medication tolerance, drug–drug interactions, CD4 cell counts, prior treatment history, and antiretroviral drug resistance were found to be significantly associated with virologic suppression [36, 41-44].

Achieving the third target of the UNAIDS 95-95-95 goals, ensuring that 95% of people living with HIV on ART achieve sustained VS, is essential to curbing HIV transmission and improving health

outcomes [45]. Individual-level variables, maternal healthcare utilization, structural barriers, health system factors, and socioeconomic factors are the main challenges in achieving this goal [44-49].

In addition, there is a lack of comprehensive evidence on virologic suppression among pregnant and breastfeeding women at the national and regional levels in Africa. Thus, the goal of this systematic review and meta-analysis is to assess the pooled prevalence of virologic suppression and identify associated factors among HIV-positive pregnant and breastfeeding women receiving ART in Africa. This comprehensive data will assist health professionals, policymakers, and guideline developers in implementing evidence-based strategies to improve viral load suppression in these populations, hence reducing HIV perinatal transmission.

Methods and materials

Study protocol and search strategy

This systematic review adhered to the Preferred Reporting Items for Systematic Reviews and Meta-Analysis (PRISMA) standards [50] **(S1 file)**. The study protocol for the review has been registered on PROSPERO with reference ID CRD420251186121 and available on: https://www.crd.york.ac.uk/PROSPERO/view/CRD420251186121.

A rigorous and comprehensive review of Google Scholar, PubMed, Hinari, and ScienceDirect for original research on virologic suppression and associated factors conducted in Africa. The inclusion and exclusion criteria were established based on the condition, context, and study population (Co Co Pop) approach. Previous studies that met the eligibility criteria were retrieved. A search strategy was developed for the databases by combining keywords with Boolean operators. Ultimately, we employed the following search term combination: "viral load suppression", "virologic suppression", "viral suppression", "HIV viral load non-suppression",

"viral load", "detectable viral load", "undetectable viral load", "virological suppression", "HIV viral load", "plasma viremia", "HIV viral suppression", "HIV viremia", "HIV-positive women", pregnant, "lactating mothers", "postpartum women", "breastfeeding mothers", "antiretroviral therapy", ART and Africa. To obtain additional articles from the citation list of publications found in the databases that were accessible, snowballing techniques were also employed **(S2 file)**.

Eligibility criteria

Inclusion criteria

Condition: The condition of interest was virologic suppression among HIV-positive pregnant and lactating women receiving antiretroviral therapy in Africa.

Context: All primary studies that reported the prevalence and/or associated factors of virologic suppression in the Africa context.

Population: The study population for this review is pregnant and lactating mothers living with HIV on ART.

Study design: All primary observational studies, including cross-sectional, case-control, and cohort studies that reported the prevalence and/or associated factors virologic suppression among HIV-positive pregnant and lactating women receiving ART.

Publication year: Both published and unpublished articles between January 1, 2016 (benchmark for the beginning of the Sustainable Development Goals (SDG) era, and April 30, 2025, were included.

Exclusion criteria

Articles that failed to report the outcome of interest, narrative reviews, qualitative reviews, expert comments, case reports, editorials, letters, and methodological studies were excluded from the review.

Measurement of outcome variables

This study aimed to determine the pooled prevalence of virologic suppression among pregnant and lactating women receiving antiretroviral therapy in Africa. This study's second objective was to determine the variables that are associated with virologic suppression in these key populations. The World Health Organization (WHO) defined virological suppression as when viral load copies become ≤1,000 copies/mL of blood after six months of ART initiation [9, 51]. However, virologic failure (VF) is defined as a VL persistently over 1000 copies/mL in two consecutive measurements after 6 months (3 months apart) on enhanced ART adherence support. In this systematic review and meta-analysis, we employed a viral load threshold of ≤1,000 copies/mL (virologic suppression), which is consistent with the WHO guideline criteria utilized in standard clinical decision-making [16, 51, 52].

Quality assessment for included studies

The quality of the included primary studies was evaluated independently by two authors (BAM and NTT). Each study was assessed using the Critical Appraisal Checklist developed by the Joanna Briggs Institute (JBI). Twenty-eight of the included papers were evaluated using the 9-item cross-sectional checklist, while 27 used the 11-item cohort checklist [53, 54]. Studies with a JBI checklist score of five or above were regarded as being of acceptable quality, indicating a low likelihood of bias. Any disputes between the two authors were settled by open conversation and consultation with GMA, the third author.

Data extraction and management

Data extraction from the included publications was performed independently by two authors (BAM and NTT) using a standardized abstraction form developed in Excel. In accordance with

the inclusion and exclusion criteria, all identified studies were imported into the reference manager EndNote 20. After screening titles and abstracts, full texts were reviewed for eligibility. Disagreements were resolved through discussion with a third reviewer (GMA). To avoid duplication, studies with overlapping or identical data were excluded. For eligible studies, the following information was extracted: identification details (first author's last name and year of publication), proportion of virologic suppression, factors associated with virologic suppression, adjusted odds ratios with 95% confidence intervals, study area, study design, study population, sample size, and risk of bias assessment method **(S3 file)**.

Data synthesis and statistical analysis

All statistical data analyses were carried out after the data were collected using a Microsoft Excel spreadsheet and exported to the statistical program STATA version 17. Texts, tables, and forest plots were used to display the extracted data. A binomial distribution was used to determine each study's standard error of prevalence of the outcome variable. The Higgins I-squared (I^2) test was used to check for heterogeneity in the pooled prevalence of the studies. The heterogeneity among the included studies was classified as low, moderate, or high based on I^2 values of <25%, 50–75%, and >75%, respectively [55].

A random-effects model with restricted maximum likelihood (REML) was used to determine the pooled prevalence of virologic suppression in Africa. REML is preferred over the Der Simonian and Laird method because it employs a likelihood-based approach, which typically offers a more stable and unbiased estimate of between-study variance (τ^2) and more accurate confidence interval coverage, especially in the presence of high heterogeneity. Even though Der Simonian and Laird's method offers a narrower confidence interval (CI), it underestimates the between-study variance

(τ^2) and inflates type I error (false positive) under extreme heterogeneity, which affects the robustness of the pooled estimate [56, 57].

A subgroup analysis and meta-regression analysis were conducted across the publication year, country, regions of Africa, ART regimen, and study population to identify potential sources of study heterogeneity. In addition, we performed a leave-one-out sensitivity analysis to look at how particular studies influence the pooled estimate. The pooled estimates from across the continent were then displayed in forest plots and tables, along with their corresponding 95% confidence intervals. Potential small-study effects were assessed using funnel plots and Egger's regression test. Visually, publication bias has been assessed using a funnel plot (23). Statistically, potential publication bias was evaluated using Egger's test, with a p-value < 0.05 suggesting evidence of publication bias [58]. Finally, we conducted a meta-analysis and systematic synthesis to examine the determinants of virologic suppression among pregnant and lactating women receiving ART in Africa.

Results

Study selection and characteristics of the included studies

All searched databases, including Google Scholar, yielded a total of 1,639 research articles; 406 duplicate records were deleted, and the remaining 1,233 papers were further screened. However, the majority of papers (n = 1,108) were removed after reading their titles and abstracts. The remaining 125 full-text articles were then reviewed for eligibility criteria, and 70 studies were excluded for a variety of reasons, including insufficient data, a difference in the viral load suppression cutoff point, variation in the study context, being unrelated to the outcome of interest, systematic reviews, and qualitative reviews. Then, the remaining 55 eligible studies with a total of 304,883 HIV-positive pregnant and breastfeeding women were included in the final quantitative

meta-analysis [16, 28, 44, 52, 59-97]. Among the included studies, 26 articles had reported an undetectable VL threshold (VL <50 copies/ml). In terms of study distribution across Africa, 31 and 13 studies were undertaken in Southern Africa and Eastern Africa, respectively **(Fig. 1).**

Figure 1: PRISMA flow diagram showing the selection process of studies for the virologic suppression among HIV-positive pregnant and lactating women receiving ART in Africa.

Prevalence of virological suppression among pregnant and lactating mothers receiving ART in Africa

In this meta-analysis, the pooled prevalence of virologic suppression among pregnant and lactating women receiving ART in Africa was 80.86% (95% CI: 77.63%, 84.09%, $I^2 = 99.84\%$, $\tau^2 = 146.21$, p-value = 0.000). Additionally, the pooled estimate showed that only 60.92% (95% CI: 52.46%, 69.39%, $I^2 = 99.91\%$) of participants achieved an undetectable viral load. Subgroup analyses were conducted to explore potential sources of heterogeneity, such as differences in study setting, population characteristics, and study design. Besides, sensitivity analyses were performed to assess the robustness of the pooled prevalence and to determine whether any single study had a disproportionate influence on the overall results **(Fig. 2)**.

Figure 1: A forest plot that shows the pooled prevalence of virologic suppression among HIV-positive pregnant and lactating women receiving antiretroviral therapy in Africa.

The 95% prediction interval (PI) was 57.4% to 95.5%, indicating that the effect size of a new study conducted in a comparable setting could plausibly fall within this CI. Even though the pooled prevalence offers a useful overall summary, the wide prediction interval indicates substantial between-study heterogeneity. Accordingly, the pooled estimate should be interpreted as a descriptive summary of existing studies rather than a precise estimate for all settings. Due to the substantial heterogeneity among studies, a median-based summary was employed as an alternative

statistical method. Consequently, the median prevalence across studies was 84.1% (IQR: 75.5%, 89.7%), providing a robust, non-parametric summary that is not influenced by extreme values or high variability among studies.

Heterogeneity and subgroup analysis

Subgroup analysis was performed based on the country, the regions of Africa, and the study population. Accordingly, Ethiopia had the highest prevalence of VS, 94.11% (95% CI: 91.16, 97.07), while the Democratic Republic of Congo had the lowest prevalence, 62.66% (95% CI: 58.56, 66.75). Moreover, the lowest overall prevalence of VS in pregnant and lactating mothers living with HIV was found in the Central Africa region (62.66%; 95% CI: 58.56, 66.75), whereas the highest was in Western and East Africa, which found nearly 84% of viral suppression. In terms of the study population, 81% of pregnant and postpartum mothers had VS, which is nearly the same pooled prevalence.

Overall, the subgroup analysis demonstrated substantial geographic variation in virologic suppression across Africa, highlighting differences in ART program effectiveness and context-specific variables. This significant between-studies heterogeneity suggests that multiple factors, such as variations in ART regimens, level of ART adherence, health system capacity, availability of viral load monitoring, and sociocultural barriers, contribute to the observed differences in virologic suppression **(Table 1)**.

Publication bias

The presence of publication bias was graphically checked using a funnel plot, and it was confirmed using Egger's test. The symmetrical appearance of the funnel plot in this analysis indicates that there is no publication bias among the studies **(Fig. 3)**. Egger's (p-value = 0.2402) test were

statistically non-significant, suggesting that there was no substantial publication bias in the included studies.

Figure 2: A funnel plot test that indicates the virologic suppression among HIV-positive pregnant and lactating women receiving antiretroviral therapy in Africa.

Sensitivity analysis

To examine the impact of a single study on the estimated effect size, a leave-one-out sensitivity analysis was conducted using the random-effects model. The results, however, demonstrate that the pooled estimate was not substantially impacted by a single study, and the point estimate of the excluded study is within the confidence interval of the overall estimate of virologic suppression. This demonstrated that the average estimate of virologic suppression among HIV-positive pregnant and breastfeeding mothers in Africa was robust **(Fig. 4)**.

Figure 4: A leave-one-out sensitivity analysis of virologic suppression among HIV-positive pregnant and lactating women on antiretroviral therapy in Africa.

Meta-regression analysis

A meta-regression analysis, together with subgroup and sensitivity analyses, was conducted to identify potential sources of heterogeneity. Study-level characteristics, including publication year (before vs. after 2020), ART regimen, country, African region, and sample size, were entered as covariates in the model. The analysis showed that use of a DTG-based ART regimen and the African region were significant moderators, accounting for part of the observed heterogeneity across studies. Thus, DTG-based ART regimens demonstrated significantly higher virologic suppression compared to non-DTG-based regimens ($\beta = 16.43$, $p = 0.012$). This suggests that variations in ART regimens had a significant role in the observed heterogeneity between studies, with DTG-based therapy significantly associated with virologic suppression. Furthermore, the

region of Africa was substantially linked with viral load suppression (β = -2.92, p = 0.037), indicating considerable regional variations in virologic suppression rate. This study suggests that contextual factors related to geographic location, such as disparities in health system capability, implementation of ART programs, and VL monitoring, influence the observed between-study heterogeneity **(Table 2)**.

Factors associated with virologic suppression among pregnant and lactating mothers receiving ART in Africa

This systematic review and meta-analysis investigated 16 studies that identified factors associated with virologic suppression among pregnant and lactating mothers receiving ART in Africa [16, 17, 44, 60, 61, 64, 65, 74, 76, 79-81, 87, 89, 98]. In general, virologic suppression among HIV-positive pregnant and lactating women was substantially correlated with sociodemographic variables (women's age, higher educational level, being married/cohabitating, urban residency) [16, 17, 44, 52, 60, 65, 74, 98-100], healthcare utilization (time of ANC booking and ART initiation, regimen and duration of ART, baseline VL threshold, adherence status of ART) [17, 39, 44, 60, 65, 98, 99, 101], and accessibility and availability of healthcare services (long distance to health facility, shortage of health professionals, inadequate counseling, ART drugs running out, sub-optimal sample transportation) [12, 102, 103].

The odds of having virologic suppression were higher among women living in urban compared with rural resident women [65, 98, 100]. Furthermore, women's educational status was significantly associated with VS. Evidence from different studies consistently showed that women with secondary school or above had higher VS compared to women with no formal education [65, 74].

Virologic suppression was not achieved among women who started ANC booking or initiated ART in the second or third trimester [44, 65, 98, 101]. Similarly, long distance to reach a health facility, shortage of health providers, inadequate counseling, ART drugs running out, and sub-optimal sample transportation are linked with failure of virologic suppression [12, 102, 103]. In addition, advanced WHO stage of HIV (clinical stage II, III or IV) [39, 101]; fear of stigma and discrimination [12, 102, 103]; and disclosure of HIV status [16, 39, 98, 104-106] were found to be significantly associated with virologic suppression among HIV-positive pregnant and lactating women receiving ART **(S4 file).**

In this meta-analysis, younger women (15–24 years) (AOR = 0.49; 95% CI: 0.32–0.77) were 51% less likely to attain virologic suppression compared to those aged 25 years and older [16, 65, 99]. Furthermore, a pooled analysis of three studies found that women who disclosed their HIV status (AOR = 1.66; 95% CI: 1.31, 2.11) were 1.66 times more likely to achieve virologic suppression compared to their counterparts.

In addition, the likelihood of achieving viral load suppression was 6.5 times higher for pregnant and lactating women on a first-line ART regimen (AOR = 6.53; 95% CI: 1.93, 22.06) than for those on second- or third-line regimens. Finally, the odds of virologic suppression among pregnant and lactating women who had good ART adherence (AOR = 3.61, 95% CI: 1.18, 11.02) were 3.61 times more likely as compared to those who had poor adherence to ART **(Table 3)**

Discussion

The main objectives of this systematic review and meta-analysis were to determine the proportion of virologic suppression among pregnant and lactating women receiving ART and identify factors associated with it. Thus, the pooled prevalence of virologic suppression among pregnant and lactating women taking ART in Africa was 80.86% (95% CI: 77.63%, 84.09%). Additionally, the

pooled estimate showed that only 60.92% of participants achieved an undetectable viral load (95% CI: 52.46%, 69.39%; $I^2 = 99.91\%$). This conclusion was comparable with a study conducted in East Africa, which found 80.6% virologic suppression among patients living with HIV [107]. However, the finding of this review was lower than studies reported: 84% in Ethiopia [108], 85% in Sub-Saharan Africa [109], and far lower than the global target of achieving 95% viral suppression in patients receiving ART [31]. In contrast, the findings of the current study were higher than a study conducted in Ethiopia (71%) [110].

This could be due to the difference in the study population, study setting, and availability and adherence to ART. Thus, the study population for the previous studies was adults living with HIV, whereas the current study involves pregnant and lactating women receiving ART in Africa. The possible explanation could relate to physiological and immunological changes during perinatal periods [111]. Pregnancy alters the immune system and pharmacokinetics of ART drugs, which reduce the effectiveness of ART and increase maternal viral load [111, 112].

Furthermore, delayed initiation or inadequate adherence to ART, perceived shame, and socioeconomic or health system constraints, such as limited regular VL surveillance, ART stock-outs, and a shortage of maternity professionals, could be the possible reason for non-virological suppression [16, 60, 113]. Suboptimal viral suppression or virologic failure jeopardizes not only the health of women but also raises the risk of HIV MTCT during pregnancy, childbirth, and breastfeeding, posing a challenge to the global effort towards eliminating pediatric HIV infection [60, 114, 115].

The UNAIDS's 95-95-95 goals for all groups of populations, including pregnant and lactating women, demonstrate the globe's ongoing dedication to combating HIV/AIDS and reaching the 2030 sustainable development goal of "ending AIDS" as a global health concern [32]. Despite

considerable global progress towards reaching the 95-95-95 goals in the past decade, many low-middle-income countries, including many African countries, are still far from this goal [116]. Reaching UNAIDS's third 95% target, ensuring that 95% of people living with HIV on ART maintain sustained VS, is vital for preventing HIV transmission, improving health outcomes, and optimizing the quality of life [47]. Nonetheless, ART availability and adherence, ongoing structural and socioeconomic inequities, health-care system fragilities, and financing constraints Despite the hurdles, targeted interventions, including better ART adherence support, integrated maternal-HIV care, robust VL monitoring, and addressing structural, financial, and sociocultural barriers to close the gap and achieve the global target, ultimately lower vertical transmission and improve maternal and child health outcomes [47, 117].

According to the findings of this study, Africa has not yet achieved the global target of attaining virological suppression in 95% of patients who received ART. To increase the possibility of successful virological outcomes, a variety of factors and problems must be addressed. In this systematic review, individual-level variables, healthcare utilization (time of ANC booking, time of ART initiation, being on a first-line ART regimen, duration of ART, baseline VL threshold, adherence status to ART), and barriers to healthcare services, including distance to health facility, shortage of health professionals, disclosure of HIV status, inadequate counseling, ART drug stock-out, and sub-optimal sample transportation, were significantly associated with virologic suppression among HIV-positive pregnant and lactating women in Africa.

In this study, the odds of virologic suppression among young women, particularly those 15-24 years old, were decreased by 51% when compared with those aged 25 years and above. The finding of this study was consistent with other studies [113, 118-120]. Younger people receiving treatment for HIV might have difficulty with compliance due to various factors, including employment

status, societal expectations, worries regarding stigma and disclosure, mental health challenges, and lacking health literacy [113, 121]. In Africa, the stigma that exists at the individual, interpersonal, social, and organizational levels remain to pose significant hurdles for receiving and adhering to ART for adolescents living with HIV [122, 123].

Antiretroviral therapy (ART) has changed the treatment of HIV from a deadly illness to a chronic condition that can be managed owing to its increased accessibility and continuing adherence [124]. Nevertheless, there are several obstacles in the adolescents HIV care cohort, which frequently put this age group at risk for less favorable outcomes than adults in the HIV care pathway. These issues include poorer adherence, lower retention in therapy, a lower rate of virological suppression, and greater rates of mortality [121, 125-127].

Collaborated global, regional, and national efforts are needed to reach the UNAIDS 95% sustained viral suppression rate, especially for adolescents living with HIV, who are frequently linked with elevated rates of virological failure (VF) [32, 117]. Understanding and addressing the unique challenges faced by young women helps healthcare workers and decision-makers create specific treatments that boost viral suppression rates and enhance health for this vulnerable age group. Therefore, promoting ART adherence support, regular VL monitoring, and accessible and flexible adolescent-youth-friendly HIV care, while guaranteeing accessibility, privacy, and a favorable environment, plays a pivotal role in achieving VS in these groups of populations [122, 123].

The odds of achieving virologic suppression were higher among women living in urban areas than women residing in rural areas. This finding was consistent with previous study findings [65, 98, 100]. This could be due to women living in urban areas being more likely to have better awareness and access to HIV care, including periodic VL monitoring and continuous ART supply, as well as

enhanced counseling on ART adherence. Whereas rural women frequently encounter geographical, infrastructural, and service-related barriers that impede optimal ART adherence and VS [98]. Furthermore, women's educational status was significantly associated with virologic suppression, as studies consistently show that women with secondary or higher education are more likely to achieve VS than those with no formal education [65, 74]. This could be explained by the fact that education increases awareness of navigating medical care, the importance of ART adherence, and access to HIV care; in contrast, less educated women could find it challenging to grasp treatment, receive regular medical care, and fear stigma. Moreover, pregnant and breastfeeding women who disclosed their HIV status were significantly associated with achieving virological suppression compared to those who did not. This finding is in agreement with other studies that revealed disclosure of HIV status increases the likelihood of virologic suppression [16, 36, 39, 98, 104-106, 108, 113, 128]. Disclosing one's HIV status to family, friends, and intimate partners can have positive health impacts. Several studies show that individuals who disclosed their serostatus had better social assistance, stronger family and relationship solidarity, lower symptoms of depression and anxiety, improved physical health, psychological assistance, financial support, and better adherence to ART [129-131]. However, fear of stigma and discrimination was found to be significantly associated with virologic suppression among HIV-positive pregnant and lactating women receiving ART [12, 102, 103, 113]. Stigma can take many forms, including societal discrimination, internalized prejudice, family stigma, and stigma in healthcare environments. This may result in fear of disclosing status or withholding drugs, lack of self-worth, and failure to adhere to ART [45, 132, 133].

Women who began ANC contacts or started ART in the second or third trimester were less likely to achieve virologic suppression [44, 65, 98, 101]. A late beginning of ANC follow-up and ART

decreases the time period of effective ART for decreasing VL before childbirth and limits chances for regular monitoring of ART treatment outcomes, enhanced counseling for adherence, and prompt response to treatment challenges, which are essential for attaining and sustaining optimum virologic suppression to prevent MTCT of HIV.

Advanced WHO HIV stages (II, III, or IV), marked by opportunistic infections, were significantly associated with failure to achieve virologic suppression among pregnant and lactating women on ART [39, 101, 113]. Advanced HIV stages elevate the likelihood of virologic suppression failure because growing immune suppression and opportunistic infections may hamper ART efficacy, alter adherence, and increase the risk of treatment failure, thereby rendering VL control more challenging for pregnant and breastfeeding women [39, 113].

This study also found that patients on first-line ART regimens had higher rates of virologic suppression compared to those on second- or third-line regimens, consistent with findings from other studies [36, 37, 134, 135]. This could be explained as first-line ART drugs (TDF-3TC-DTG) having higher efficacy (sustained VL suppression), fewer adverse effects, and lower resistance when compared to second- and third-line ART regimens [25, 136, 137]. Therefore, all HIV-positive pregnant and lactating women should initiate triple ART as early as possible, regardless of their CD4 count or WHO clinical stage. This leads to optimal suppression of viral load to undetectable levels, thereby preventing the vertical transmission of HIV [138]. Nevertheless, the virus can never be totally eliminated from the human body, so an individual should continue to take the treatments even after the signs and symptoms have subsided and adhere to safer sexual practices [25, 136, 139].

Furthermore, good adherence to antiretroviral therapy was significantly associated with virologic suppression among pregnant and breastfeeding women, aligning with findings from previous

studies [36, 37, 39, 134]. The plausible explanation is due to the fact that good adherence to ART sustains maximal viral load suppression to undetectable levels, lowers drug resistance, and boosts the immune system (CD4 cell counts), which leads to substantially lowering the risk of mother-to-child transmission of HIV and improving the overall health of individuals [136, 140]. However, adherence to ART remains a major health issue worldwide, particularly in low- and middle-income nations. Virologic and clinical outcomes are highly dependent on strong compliance to ART, while poor adherence to antiretroviral drugs has been found to be a significant predictor of virologic failure, drug resistance, progression of disease, admissions to hospitals, high death rate, and medical care expenses [113, 141, 142].

On the other hand, long distance to reach a health facility, shortage of health providers, inadequate counseling, ART drugs running out, and shortage of HIV commodities linked are with failure of virologic suppression [12, 45, 98, 102, 103]. [45, 143]. Health facility availability is a crucial infrastructural variable determining adherence to ART, especially in low-income countries, in which medical infrastructure is frequently poor. Individuals with work or domestic responsibilities have significant challenges due to the facility's limited operation hours or inconvenient hours, which are often only available during weekday daytime [45].

Similarly, ART stockouts in healthcare facilities result in viral rebounding, missed ART doses, drug resistance, and switching to the second-line therapy [45, 144-146]. ART drugs can occasionally be inaccessible due to supply chain issues or financing shortages in several developing countries. Patients may receive an insufficient supply or nothing at all as they come for refills following a stockout, which would cause them to skip medications [45, 146, 147].

In general, the findings of this review have various implications for clinical practice, policy, and future research works in these population groups. Since lifelong first-line ART regimens and

enhanced adherence to ART have been strongly linked to greater virologic suppression, healthcare practitioners should increase adherence counseling, particularly during prenatal and postpartum contacts. The lower suppression rates in younger women demonstrate the necessity for youth-specific strategies like youth-friendly clinics, psychosocial assistance, and sexual and reproductive health education incorporated into HIV treatment programs. Optimizing virologic suppression in pregnant and breastfeeding women on ART in Africa requires a comprehensive strategy that includes upgrading clinical practices, ensuring responsive policy, and ongoing VL monitoring and evaluation to address ongoing barriers, gaps, and disparities to achieve VS. Further qualitative and quantitative studies are required to investigate different sociocultural, behavioral, psychological, and structural factors that lead to virologic non-suppression among HIV-positive pregnant and breastfeeding women in Africa.

Strengths and limitations of the study

To our knowledge, this would be the first meta-analysis to assess the overall estimate of virologic suppression and associated factors among HIV-positive pregnant and nursing women in Africa. We searched multiple databases to include both published and unpublished studies and followed a meticulous screening approach to compile all eligible studies. Thus, the findings of this review provide important baseline evidence for health providers, policymakers, academicians, and global partners to design and implement evidence-based interventions to achieve the WHO 95-95-95 global target to eliminate vertical transmission of HIV.

Despite the fact that enough primary studies were used to estimate the overall viral load suppression, many countries and some African regions were not evenly represented. The presence of significant heterogeneity among the included studies might affect the generalizability of the finding. This might be due to variations in the study area and the methodological quality of the

studies, such as study design, sampling technique, response rate, sample size, and study setting, that could alter the pooled estimate's generalizability. Additionally, due to the concentration of studies in some countries, such as South Africa, it could restrict the conclusion of the finding to the entire continent. We employed a random-effects model for analysis in order to overcome this significant variability. Additionally, we performed subgroup analyses, sensitivity analyses, and meta-regression to identify potential causes of substantial between-study heterogeneity.

Conclusions

According to this systematic review and meta-analysis, the overall prevalence of virologic suppression among pregnant and lactating women taking ART in Africa (80.86%) is lower than USAID's 95% global target of VL suppression. In this study, socio-demographic characteristics (women's age, higher educational level, being married/cohabitant, and urban residency) and healthcare utilization (time of ANC booking, time of ART initiation, being on a first-line ART regimen, duration of ART, and adherence status to ART) were significantly associated with virologic suppression among HIV-positive pregnant and lactating women in Africa.

Additionally, virologic suppression is also substantially linked with various facilitators and barriers, including disclosing of HIV status, fear of stigma, distance to health facility, shortage of health professionals, ART drug stock-out, and lack of HIV care commodities. This highlights the importance of targeted interventions for young HIV-positive women, encouraging disclosing HIV status, initiating first-line antiretroviral therapy regimens, and promoting antiretroviral therapy adherence; strengthening healthcare systems that offer regular viral load monitoring; and addressing socio-demographic and ART-related factors as vital steps toward improving treatment outcomes and reducing perinatal transmission of HIV.

List of abbreviations

ART: Antiretroviral therapy, AOR: Adjusted odds ratio, CI: Confidence Interval, CD4: Cluster of differentiation 4, HIV: Human Immunodeficiency Virus, JBI: Joanna Briggs Institute, MTCT: Mother-to-Child Transmission of HIV, SRM: Systematic review and meta-analysis, VL: Viral load, VS: Virologic Suppression, UNAIDS: United Nations Programme on HIV/AIDS, WHO: World health organization,

Acknowledgements

The authors of this study would like to express our heartfelt gratitude to all of the authors of the studies included in this systematic review and meta-analysis.

References

1. World Health Organization (WHO). HIV statistics, globally and by WHO region, 2025. Accessed on: https://cdn.who.int/media/docs/default-source/hq-hiv-hepatitis-and-stis-library/who-ias-hiv-statistics_2025-new.pdf. 2025 [
2. United Nations Programme on HIV/AIDS (UNAIDS). UNPoHA. Global HIV & AIDS statistics — Fact sheet. Access: https://www.unaids.org/en/resources/fact-sheet. 2025 [
3. United Nations Programme on HIV/AIDS (UNAIDS). Fact sheet 2024 - Latest global and regional HIV statistics on the status of the AIDS epidemic. 2024.
4. Tilaye BA, Hailemeskel HS, Baye FD, Hailu MK, Mekonnen GB, Arage G, et al. Incidence of mother-to-child transmission of HIV and predictors of positivity among HIV exposed infants in South Gondar public hospitals, Northwest Ethiopia: competing risk regression model. *BMC Pediatrics*. 2024;24(1):597.
5. Chi BH, Mbori-Ngacha D, Essajee S, Mofenson LM, Tsiouris F, Mahy M, et al. Accelerating progress towards the elimination of mother-to-child transmission of HIV: a narrative review. *J Int AIDS Soc*. 2020;23(8):e25571.

6. World Health Organization (WHO). Mother-to-child transmission of HIV. https://www.who.int/teams/global-hiv-hepatitis-and-stis-programmes/hiv/prevention/mother-to-child-transmission-of hiv#:~:text=Globally%2C%20an%20estimated%201.3%20million,been%20shown%20to%20be%20feasible. 2024 [

7. Omonaiye O, Kusljic S, Nicholson P, Manias E. Medication adherence in pregnant women with human immunodeficiency virus receiving antiretroviral therapy in sub-Saharan Africa: a systematic review. *BMC public health*. 2018;18:1-20.

8. World Health Organization (WHO). Progress report 2016: prevent HIV, test and treat all: WHO support for country impact. Progress report 2016: prevent HIV, test and treat all: WHO support for country impact 2016.

9. Seale A, Baggaley R, Vojnov L, Doherty M. Role of viral suppression in HIV treatment and prevention and its potential in addressing harmful laws and discrimination. *Sexual Health*. 2023;20(3):181-5.

10. World Health Organization (WHO). The role of HIV viral suppression in improving individual health and reducing transmission: policy brief: *World Health Organization*; 2023.

11. Chilaka VN, Konje JC. HIV in pregnancy–An update. E*uropean Journal of Obstetrics & Gynecology and Reproductive Biology*. 2021;256:484-91.

12. Musanhu CCC, Takarinda KC, Shea J, Chitsike I, Eley B. Viral load testing among pregnant women living with HIV in Mutare district of Manicaland province, Zimbabwe. *AIDS Research and Therapy*. 2022;19(1):52.

13. World Health Organization (WHO). Updated recommendations on HIV prevention, infant diagnosis, antiretroviral initiation and monitoring. *World Health Organization*; 2021.

14. World Health Organization (WHO). Updated Recommendations on HIV Prevention, Infant Diagnosis, Antiretroviral Initiation and Monitoring: W*orld Health Organization*; 2022.

15. World Health Organization (WHO) Guidelines Approved by the Guidelines Review Committee. Consolidated Guidelines on the Use of Antiretroviral Drugs for Treating and Preventing HIV Infection: Recommendations for a Public Health Approach. Geneva: *World Health Organization*.; 2016.

16. Schrubbe LA, Stöckl H, Hatcher AM, Marston M, Kuchukhidze S, Calvert C. Prevalence and risk factors of unsuppressed viral load among pregnant and breastfeeding women in sub-Saharan Africa: analysis from population-based surveys. *AIDS*. 2023;37(4):659-69.

17. Hailu G, Keraleme A, Zealiyas K, Tesema A, Nuramed N, Girmachew F, et al. Human Immunodeficiency Virus (HIV) viral load suppression status and associated factors among pregnant women receiving Highly Active Antiretroviral Therapy (HAART) in Ethiopia. *Virology Journal*. 2025;22(1):49.

18. Munyaneza A, Kim H-Y, Shi Q, Brazier E, Ross J, Muhoza B, et al. Factors associated with viral load suppression in pregnant and postpartum women living with HIV in Rwanda: an open-observational cohort study. *Frontiers in Public Health*. 2025;13:1544165.

19. Elijah O, Umukoro E, Igben V, Moke E, Ojezele M, Onitiri-Elijah C. Prevention of Mother-To-Child Transmission of HIV (PMTCT) in Sub-Saharan Africa: Past and Present Perspectives. *International Journal of Forensic Medical Investigation*. 2025;11(1).

20. Mukose AD. Implementation of lifelong ART (Option B+) for Prevention of Mother-to-Child Transmission (PMTCT) of HIV in Uganda: *University of Antwerp*; 2024.

21. World Health Organization (WHO). Introducing a framework for implementing triple elimination of mother-to-child transmission of HIV, syphilis and hepatitis B virus: policy brief: *World Health Organization*; 2024.

22. Adelekan B, Harry-Erin B, Okposo M, Aliyu A, Ndembi N, Dakum P, et al. Final HIV status outcome for HIV-exposed infants at 18 months of age in nine states and the Federal Capital Territory, Nigeria. *PLoS One*. 2022;17(2):e0263921.

23. Gilleece DY, Tariq DS, Bamford DA, Bhagani DS, Byrne DL, Clarke DE, et al. British HIV Association guidelines for the management of HIV in pregnancy and postpartum 2018. *HIV Medicine*. 2019;20 Suppl 3:s2-s85.

24. Rasmussen DN, Vieira N, Hønge BL, da Silva Té D, Jespersen S, Bjerregaard-Andersen M, et al. HIV-1 and HIV-2 prevalence, risk factors and birth outcomes among pregnant women in Bissau, Guinea-Bissau: a retrospective cross-sectional hospital study. *Scintific Reports*. 2020;10(1):12174.

25. World Health Organization (WHO). Updated recommendations on first-line and second-line antiretroviral regimens and post-exposure prophylaxis and recommendations on early infant diagnosis of HIV; 2018. p. [1-82].

26. Hoffman RM, Black V, Technau K, van der Merwe KJ, Currier J, Coovadia A, et al. Effects of highly active antiretroviral therapy duration and regimen on risk for mother-to-child transmission of HIV in Johannesburg, South Africa. *J Acquir Immune Defic Syndr*. 2010;54(1):35-41.

27. Fox MP, Pascoe S, Huber AN, Murphy J, Phokojoe M, Gorgens M, et al. Adherence clubs and decentralized medication delivery to support patient retention and sustained viral suppression in care: Results from a cluster-randomized evaluation of differentiated ART delivery models in South Africa. *PLoS Med*. 2019;16(7):e1002874.

28. Chagomerana MB, Miller WC, Tang JH, Hoffman IF, Mthiko BC, Phulusa J, et al. Optimizing prevention of HIV mother to child transmission: Duration of antiretroviral therapy and viral suppression at delivery among pregnant Malawian women. *PLoS One*. 2018;13(4):e0195033.

29. Lazenby GB, Powell AM, Sullivan SA, Soper DE. The Impact of Delivery in a Rural County on a Cohort of Women Living with HIV Infection and Their Infants. *J Rural Health*. 2019;35(3):319-29.

30. Awaidy SA, Ghazy RM, Mahomed O. Progress of the gulf cooperation council (gcc) countries towards achieving the 95-95-95 UNAIDS targets: a review. *Journal of epidemiology and global health*. 2023;13(3):397-406.

31. Mwende Wairimu G. The Global Burden of HIV and Progress toward the 2030 UNAIDS Targets: A Comprehensive Review.

32. Frescura L, Godfrey-Faussett P, Feizzadeh A, El-Sadr W, Syarif O, Ghys PD, et al. Achieving the 95 95 95 targets for all: A pathway to ending AIDS. *Plos one*. 2022;17(8):e0272405.

33. Mosha IH, Nyondo GG, Munishi CG, Njiro BJ, Bwire GM. Prevalence and factors associated with viral non-suppression in people living with HIV receiving antiretroviral therapy in sub-Saharan Africa: A systematic review and meta-analysis. *Rev Med Virol*. 2024;34(3):e2540.

34. Agegnehu CD, Techane MA, Mersha AT, Atalell KA. Burden and Associated Factors of Virological Failure Among People Living with HIV in Sub-Saharan Africa: A Systematic Review and Meta-Analysis. *AIDS Behav*. 2022;26(10):3327-36.

35. Namaganda MM, Mukasa Kafeero H, Nakatumba Nabende J, Kateete DP, Batte C, Wanyengera M, et al. Prevalence and predictors of virological failure among the people living with HIV on antiretroviral treatment in East Africa: evidence from a systematic review with meta-

analysis and meta-regression of published studies from 2016 to 2023. *HIV Research & Clinical Practice*. 2025;26(1):2490774.

36. Melak D, Bayou FD, Yasin H, Zerga AA, Wagaye B, Ayele FY, et al., editors. Virological suppression and its predictors among HIV/AIDS patients on antiretroviral therapy in Ethiopia: Systematic review and meta-analysis. *Open forum infectious diseases*; 2024: Oxford University Press US.

37. Temere BC, Mewahegn AA, Zewudie BT, GebreEyesus FA, Abebe H, Nuru A, et al. Burden of virologic failure, and its determinants among HIV positive children in East Africa: a systematic review and meta-analysis. *African Health Sciences*. 2024;24(3):1-9.

38. Abuogi LL, Humphrey JM, Mpody C, Yotebieng M, Murnane PM, Clouse K, et al. Achieving UNAIDS 90-90-90 targets for pregnant and postpartum women in sub-Saharan Africa: progress, gaps and research needs. *Journal of Virus Eradication*. 2018;4:33-9.

39. Woldesemayat B, Yizengaw A, Worku A, Yilma A, Adane S, Bulti J, et al. HIV Viral Non-Suppression and Its Associated Factors Among PMTCT Mothers Receiving ARV Treatment in Ethiopia. *medRxiv*. 2024:2024.06. 13.24308915.

40. Gabagaya G, Rukundo G, Amone A, Wavamunno P, Namale-Matovu J, Lubega I, et al. Prevalence of undetectable and suppressed viral load in HIV-infected pregnant women initiating Option B+ in Uganda: an observational study nested within a randomized controlled trial. *BMC infectious diseases*. 2021;21:1-7.

41. Bulage L, Ssewanyana I, Nankabirwa V, Nsubuga F, Kihembo C, Pande G, et al. Factors associated with virological non-suppression among HIV-positive patients on antiretroviral therapy in Uganda, August 2014–July 2015. *BMC infectious diseases*. 2017;17:1-11.

42. Agegnehu CD, Techane MA, Mersha AT, Atalell KA. Burden and associated factors of virological failure among people living with HIV in sub-Saharan Africa: a systematic review and meta-analysis. *AIDS and Behavior*. 2022;26(10):3327-36.

43. Bakari HM, Alo O, Mbwana MS, Salim SM, Ally ZM, Musoke R, et al. Enhanced adherence counselling completion rates, viral load suppression in people with HIV in Africa: systematic review and meta-analysis. *Discover Public Health*. 2025;22(1):12.

44. Woldesenbet SA, Kufa T, Barron P, Chirombo BC, Cheyip M, Ayalew K, et al. Viral suppression and factors associated with failure to achieve viral suppression among pregnant women in South Africa. *Aids*. 2020;34(4):589-97.

45. Magura J, Nhari SR, Nzimakwe TI. Barriers to ART adherence in sub-Saharan Africa: a scoping review toward achieving UNAIDS 95-95-95 targets. *Frontiers in Public Health*. 2025;13:1609743.

46. Kulohoma B, Wesonga CA. HIV response financing challenges in Sub-Saharan Africa: barriers to achieving the 95-95-95 UNAIDS targets. Frontiers in Public Health. 2025;13:1658229.

47. Mukuku O, Govender K. At a crossroads: confronting setbacks and advancing the 95-95-95 HIV targets in sub-Saharan Africa. *AIDS Research and Therapy*. 2025;22(1):92.

48. Omonaiye O, Kusljic S, Nicholson P, Manias E. Medication adherence in pregnant women with human immunodeficiency virus receiving antiretroviral therapy in sub-Saharan Africa: a systematic review. *BMC public health.* 2018;18(1):805.

49. Kadia BM, Dimala CA, Fongwen NT, Smith AD. Barriers to and enablers of uptake of antiretroviral therapy in integrated HIV and tuberculosis treatment programmes in sub-Saharan Africa: a systematic review and meta-analysis. *AIDS research and therapy*. 2021;18(1):85.

50. Page MJ, McKenzie JE, Bossuyt PM, Boutron I, Hoffmann TC, Mulrow CD, et al. The PRISMA 2020 statement: an updated guideline for reporting systematic reviews. *BMJ (Clinical research ed)*. 2021;372:n71.

51. World Health Organization (WHO). Consolidated guidelines on HIV prevention, testing, treatment, service delivery and monitoring: recommendations for a public health approach: W*orld Health Organization*; 2021.

52. Moyo F, Mazanderani AH, Murray T, Sherman GG, Kufa T. Achieving maternal viral load suppression for elimination of mother-to-child transmission of HIV in South Africa. *Aids*. 2021;35(2):307-16.

53. Munn Z, Moola S, Lisy K, Riitano D, Tufanaru C. Methodological guidance for systematic reviews of observational epidemiological studies reporting prevalence and cumulative incidence data. *JBI Evidence Implementation*. 2015;13(3):147-53.

54. Barker TH HS, Aromataris E, Stone JC, Leonardi-Bee J, Sears K, Habibi N, Klugar M, Tufanaru C, Moola S, Liu XL. The revised JBI critical appraisal tool for the assessment of risk of bias for cohort studies. JBI Evidence Synthesis 2025.

55. Huedo-Medina TB, Sánchez-Meca J, Marín-Martínez F, Botella J. Assessing heterogeneity in meta-analysis: Q statistic or I^2 index? *Psychological methods*. 2006;11(2):193.

56. Langan D, Higgins JPT, Jackson D, Bowden J, Veroniki AA, Kontopantelis E, et al. A comparison of heterogeneity variance estimators in simulated random-effects meta-analyses. *Res Synth Methods*. 2019;10(1):83-98.

57. Boedeker P, Henson RK. Evaluation of heterogeneity and heterogeneity interval estimators in random-effects meta-analysis of the standardized mean difference in education and psychology. *Psychol Methods*. 2020;25(3):346-64.

58. Begg CB, Mazumdar M. Operating characteristics of a rank correlation test for publication bias. *Biometrics*. 1994:1088-101.

59. Ntombela NP, Kharsany ABM, Soogun A, Yende-Zuma N, Baxter C, Kohler HP, et al. Viral suppression among pregnant adolescents and women living with HIV in rural KwaZulu-Natal, South Africa: a cross sectional study to assess progress towards UNAIDS indicators and Implications for HIV Epidemic Control. *Reprod Health*. 2022;19(1):116.

60. Ngandu NK, Lombard CJ, Mbira TE, Puren A, Waitt C, Prendergast AJ, et al. HIV viral load non-suppression and associated factors among pregnant and postpartum women in rural northeastern South Africa: a cross-sectional survey. *BMJ Open*. 2022;12(3):e058347.

61. Adeniyi OV, Obi CL, Goon DT, Iweriebor B, Selanto-Chairman N, Carty C, et al. Factors Associated With Peripartum Virologic Suppression in Eastern Cape Province, South Africa: A Retrospective Cross-Sectional Analysis. *Clin Infect Dis*. 2021;73(10):1750-8.

62. Chetty T, Newell ML, Thorne C, Coutsoudis A. Viraemia before, during and after pregnancy in HIV-infected women on antiretroviral therapy in rural KwaZulu-Natal, South Africa, 2010-2015. *Trop Med Int Health*. 2018;23(1):79-91.

63. Phillips TK, Clouse K, Zerbe A, Orrell C, Abrams EJ, Myer L. Linkage to care, mobility and retention of HIV-positive postpartum women in antiretroviral therapy services in South Africa. *J Int AIDS Soc*. 2018;21 Suppl 4(Suppl Suppl 4):e25114.

64. Woldesenbet S, Cheyip M, Lombard C, Manda S, Ayalew K, Kufa T, et al. Progress towards the UNAIDS 95-95-95 targets among pregnant women in South Africa: Results from the 2017 and 2019 national Antenatal HIV Sentinel Surveys. *PLoS One*. 2022;17(7):e0271564.

65. Woldesenbet S, Kufa T, Manda S, Ayalew K, Lombard C, Cheyip M, et al. Association between viral suppression during the third trimester of pregnancy and unintended pregnancy among women on antiretroviral therapy: Results from the 2019 antenatal HIV Sentinel Survey, South Africa. *Plos one*. 2022;17(3):e0265124.

66. Myer L, Dunning L, Lesosky M, Hsiao NY, Phillips T, Petro G, et al. Frequency of Viremic Episodes in HIV-Infected Women Initiating Antiretroviral Therapy During Pregnancy: A Cohort Study. *Clin Infect Dis*. 2017;64(4):422-7.

67. Myer L, Phillips TK, Hsiao NY, Zerbe A, Petro G, Bekker LG, et al. Plasma viraemia in HIV-positive pregnant women entering antenatal care in South Africa. *J Int AIDS Soc*. 2015;18(1):20045.

68. Brittain K, Remien RH, Mellins CA, Phillips TK, Zerbe A, Abrams EJ, et al. Determinants of suboptimal adherence and elevated HIV viral load in pregnant women already on antiretroviral therapy when entering antenatal care in Cape Town, South Africa. *AIDS Care*. 2018;30(12):1517-23.

69. Moyo F, Haeri Mazanderani A, Murray T, Technau KG, Carmona S, Kufa T, et al. Characterizing Viral Load Burden Among HIV-Infected Women Around the Time of Delivery: Findings From Four Tertiary Obstetric Units in Gauteng, South Africa. *J Acquir Immune Defic Syndr*. 2020;83(4):390-6.

70. Myer L, Phillips TK, McIntyre JA, Hsiao NY, Petro G, Zerbe A, et al. HIV viraemia and mother-to-child transmission risk after antiretroviral therapy initiation in pregnancy in Cape Town, South Africa. *HIV Med*. 2017;18(2):80-8.

71. Langwenya N, Phillips TK, Brittain K, Zerbe A, Abrams EJ, Myer L. Same-day antiretroviral therapy (ART) initiation in pregnancy is not associated with viral suppression or engagement in care: A cohort study. *J Int AIDS Soc*. 2018;21(6):e25133.

72. Anderson K, Kalk E, Heekes A, Phelanyane F, Jacob N, Boulle A, et al. Factors associated with vertical transmission of HIV in the Western Cape, South Africa: a retrospective cohort analysis. *J Int AIDS Soc*. 2024;27(3):e26235.

73. Tsondai PR. HIV viral load monitoring in HIV-infected pregnant women established on antiretroviral therapy in Cape Town, South Africa: University of Cape Town; 2016.

74. Landes M, van Lettow M, van Oosterhout JJ, Schouten E, Auld A, Kalua T, et al. Early post-partum viremia predicts long-term non-suppression of viral load in HIV-positive women on ART in Malawi: Implications for the elimination of infant transmission. *PLoS One*. 2021;16(3):e0248559.

75. Chagomerana MB, Miller WC, Tang JH, Hoffman IF, Harrington BJ, DiPrete B, et al. Prevalence of antiretroviral therapy treatment failure among HIV-infected pregnant women at first antenatal care: PMTCT Option B+ in Malawi. *PLoS One*. 2018;13(12):e0209052.

76. Landes M, van Lettow M, Nkhoma E, Tippett Barr B, Truwah Z, Shouten E, et al. Low detectable postpartum viral load is associated with HIV transmission in Malawi's prevention of mother-to-child transmission programme. *J Int AIDS Soc*. 2019;22(6):e25290.

77. Napyo A, Tumwine JK, Mukunya D, Tumuhamye J, Arach AAO, Ndeezi G, et al. Detectable HIV-RNA Viral Load Among HIV-Infected Pregnant Women on Treatment in Northern Uganda. *Int J MCH AIDS*. 2020;9(2):232-41.

78. Munyaneza A, Kim HY, Shi Q, Brazier E, Ross J, Muhoza B, et al. Factors associated with viral load suppression in pregnant and postpartum women living with HIV in Rwanda: an open-observational cohort study. *Front Public Health*. 2025;13:1544165.

79. Gill MM, Hoffman HJ, Bobrow EA, Mugwaneza P, Ndatimana D, Ndayisaba GF, et al. Detectable Viral Load in Late Pregnancy among Women in the Rwanda Option B+ PMTCT Program: Enrollment Results from the Kabeho Study. *PLoS One*. 2016;11(12):e0168671.

80. Koss CA, Natureeba P, Kwarisiima D, Ogena M, Clark TD, Olwoch P, et al. Viral Suppression and Retention in Care up to 5 Years After Initiation of Lifelong ART During Pregnancy (Option B+) in Rural Uganda. *J Acquir Immune Defic Syndr.* 2017;74(3):279-84.

81. Gabagaya G, Rukundo G, Amone A, Wavamunno P, Namale-Matovu J, Lubega I, et al. Prevalence of undetectable and suppressed viral load in HIV-infected pregnant women initiating Option B+ in Uganda: an observational study nested within a randomized controlled trial. *BMC Infect Dis*. 2021;21(1):907.

82. Humphrey JM, Songok J, Ofner S, Musick B, Alera M, Kipchumba B, et al. Retention in care and viral suppression in the PMTCT continuum at a large referral facility in western Kenya. *AIDS Behav*. 2022;26(11):3494-505.

83. Sandbulte M, Brown M, Wexler C, Maloba M, Gautney B, Goggin K, et al. Maternal viral load monitoring: Coverage and clinical action at 4 Kenyan hospitals. *PLoS One*. 2020;15(5):e0232358.

84. Boisson-Walsh A, Ravelomanana NLR, Tabala M, Malongo F, Kawende B, Babakazo P, et al. Association of comprehensiveness of antiretroviral care and detectable HIV viral load

suppression among pregnant and postpartum women in the Democratic Republic of the Congo: a cross-sectional study. *Front Glob Womens Health*. 2024;5:1308019.

85. Yotebieng M, Mpody C, Ravelomanana NL, Tabala M, Malongo F, Kawende B, et al. HIV viral suppression among pregnant and breastfeeding women in routine care in the Kinshasa province: a baseline evaluation of participants in CQI-PMTCT study. *J Int AIDS Soc*. 2019;22(9):e25376.

86. Woldesemayat B, Yizengaw A, Worku A, Yilma A, Adane S, Bulti J, et al. HIV Viral Non-Suppression and Its Associated Factors Among PMTCT Mothers Receiving ARV Treatment in Ethiopia. *medRxiv*. 2024:2024.06.13.24308915.

87. Hailu G, Keraleme A, Zealiyas K, Tesema A, Nuramed N, Girmachew F, et al. Human Immunodeficiency Virus (HIV) viral load suppression status and associated factors among pregnant women receiving Highly Active Antiretroviral Therapy (HAART) in Ethiopia. *Virol J*. 2025;22(1):49.

88. Demissie DB, Bulto GA, Mekuria WT, Dufera FN, Gamshe EN. Evaluation of Antiretroviral Therapy Initiated Among Pregnant Women Under Option B+ by Viral Load and CD4 Count Outcomes in Selected Hospitals of West Shewa Zone, Oromia Region, Ethiopia. HIV *AIDS (Auckl)*. 2020;12:127-34.

89. Endalamaw Alamneh D, Shiferaw MB, Getachew Demissie M, Emiru MA, Zemene Kassie T, Endaylalu Lakew K, et al. Virological Outcomes Among Pregnant Women Receiving Antiretroviral Treatment in the Amhara Region, North West Ethiopia. *HIV AIDS (Auckl)*. 2023;15:209-16.

90. Aduloju O, Aduloju T, Ade-Ojo I, Akintayo A. Medication adherence in HIV-positive pregnant women on antiretroviral therapy attending antenatal clinics in Ado metropolis, south-west Nigeria: A multicentre study. South African *Journal of Obstetrics and Gynaecology*. 2020;26:65.

91. Musanhu CCC, Takarinda KC, Shea J, Chitsike I, Eley B. Viral load testing among pregnant women living with HIV in Mutare district of Manicaland province, Zimbabwe. A*IDS Res Ther*. 2022;19(1):52.

92. Duri K, Munjoma P, Mazhandu A, Marere T, Gomo E, Banhwa S, et al. Predictors and Timing to Viral Suppression in HIV-Infected Pregnant Women in the University of Zimbabwe

Birth Cohort Study During the Era of Lifelong Antiretroviral Therapy (Option B+ Treatment Strategy). *Frontiers in Virology.* 2022;2.

93. Atanga PN, Ndetan HT, Fon PN, Meriki HD, Muffih TP, Achidi EA, et al. Using a composite adherence tool to assess ART response and risk factors of poor adherence in pregnant and breastfeeding HIV-positive Cameroonian women at 6 and 12 months after initiating option B. *BMC Pregnancy Childbirth*. 2018;18(1):418.

94. Kafack EVF, Fokam J, Nana TN, Saniotis A, Halle-Ekane GE. Evaluation of plasma viral-load monitoring and the prevention of mother-to-child transmission of HIV-1 in three health facilities of the Littoral region of Cameroon. *PLoS One*. 2022;17(11):e0277271.

95. Tegueni K, Gbeasor-Komlanvi FA, Adama OIW, Sadio AJ, Amenyah-Ehlan AP, Dagnra CA, et al. Epidemiological and virological surveillance of the prevention of mother-to-child transmission of HIV among pregnant women in Togo. *BMC Pregnancy Childbirth*. 2024;24(1):278.

96. Flynn PM, Taha TE, Cababasay M, Butler K, Fowler MG, Mofenson LM, et al. Association of Maternal Viral Load and CD4 Count With Perinatal HIV-1 Transmission Risk During Breastfeeding in the PROMISE Postpartum Component. *J Acquir Immune Defic Syndr*. 2021;88(2):206-13.

97. Mazuguni N, Mazuguni F, Muro E. Detectable viral load among HIV -1 positive pregnant women on ART (Option B +) in northern Tanzania: Baseline results from the HIVDR study. *East Africa Science*. 2021;3:44-50.

98. Yotebieng M, Mpody C, Ravelomanana NL, Tabala M, Malongo F, Kawende B, et al. HIV viral suppression among pregnant and breastfeeding women in routine care in the Kinshasa province: a baseline evaluation of participants in CQI-PMTCT study. *Journal of the International AIDS Society*. 2019;22(9):e25376.

99. Adeniyi OV, Obi CL, Goon DT, Iweriebor B, Selanto-Chairman N, Carty C, et al. Factors associated with peripartum virologic suppression in Eastern Cape Province, South Africa: a retrospective cross-sectional analysis. *Clinical Infectious Diseases*. 2021;73(10):1750-8.

100. Boisson-Walsh A, Ravelomanana NL, Tabala M, Malongo F, Kawende B, Babakazo P, et al. Association of comprehensiveness of antiretroviral care and detectable HIV viral load suppression among pregnant and postpartum women in the Democratic Republic of the Congo: a cross-sectional study. *Frontiers in Global Women's Health*. 2024;5:1308019.

101. Endalamaw Alamneh D, Shiferaw MB, Getachew Demissie M, Emiru MA, Zemene Kassie T, Endaylalu Lakew K, et al. Virological outcomes among pregnant women receiving antiretroviral treatment in the Amhara Region, North West Ethiopia. *HIV/AIDS-Research and Palliative Care*. 2023:209-16.

102. Kabami J, Balzer LB, Kagoya F, Okiring J, Nangendo J, Ruhamyankaka E, et al. A multicomponent, peer-led intervention for pregnant and postpartum women with HIV improves viral suppression over time in rural Uganda. AJE Advances: *Research in Epidemiology*. 2025;1(3):uuaf017.

103. Kabami J, Akatukwasa C, Kabageni S, Nangendo J, Byamukama A, Atwiine F, et al. "I desire to have an HIV-free baby": pregnant and breastfeeding mothers' perceptions of Viral load testing and suppression in HIV care in southwestern Uganda. *Discover Social Science and Health*. 2024;4(1):60.

104. Koss CA, Natureeba P, Kwarisiima D, Ogena M, Clark TD, Olwoch P, et al. Viral suppression and retention in care up to 5 years after initiation of lifelong ART during pregnancy (Option B+) in rural Uganda. *JAIDS Journal of Acquired Immune Deficiency Syndromes*. 2017;74(3):279-84.

105. Gabagaya G, Rukundo G, Amone A, Wavamunno P, Namale-Matovu J, Lubega I, et al. Prevalence of undetectable and suppressed viral load in HIV-infected pregnant women initiating Option B+ in Uganda: an observational study nested within a randomized controlled trial. *BMC infectious diseases*. 2021;21(1):907.

106. Chohan BH, Ronen K, Khasimwa B, Matemo D, Osborn L, Unger JA, et al. Food insecurity, drug resistance and non-disclosure are associated with virologic non-suppression among HIV pregnant women on antiretroviral treatment. *PloS one*. 2021;16(8):e0256249.

107. Namaganda MM, Mukasa Kafeero H, Nakatumba Nabende J, Kateete DP, Batte C, Wanyengera M, et al. Prevalence and predictors of virological failure among the people living with HIV on antiretroviral treatment in East Africa: evidence from a systematic review with meta-analysis and meta-regression of published studies from 2016 to 2023. *HIV Research & Clinical Practice*. 2025;26(1):2490774.

108. Aytenew TM, Asferie WN, Ejigu N, Birhane BM, Tiruneh YM, Kassaw A, et al. Virological failure and associated factors among patients receiving anti-retroviral therapy in Ethiopia: A systematic review and meta-analysis. *BMJ open*. 2024;14(11):e087569.

109. Taieb F, Madec Y, Cournil A, Delaporte E. Virological success after 12 and 24 months of antiretroviral therapy in sub-Saharan Africa: Comparing results of trials, cohorts and cross-sectional studies using a systematic review and meta-analysis. *PloS one*. 2017;12(4):e0174767.

110. Melak D, Bayou FD, Yasin H, Zerga AA, Wagaye B, Ayele FY, et al. Virological Suppression and its Predictors Among HIV/AIDS Patients on Antiretroviral Therapy in Ethiopia: Systematic Review and Meta-analysis. *Open Forum Infectious Diseases*. 2024;11(4).

111. Mor G, Cardenas I. The immune system in pregnancy: a unique complexity. *American journal of reproductive immunology*. 2010;63(6):425-33.

112. Hodel EM, Marzolini C, Waitt C, Rakhmanina N. Pharmacokinetics, placental and breast milk transfer of antiretroviral drugs in pregnant and lactating women living with HIV. *Current pharmaceutical design*. 2019;25(5):556-76.

113. Mosha IH, Nyondo GG, Munishi CG, Njiro BJ, Bwire GM. Prevalence and factors associated with viral non-suppression in people living with HIV receiving antiretroviral therapy in sub-Saharan Africa: A systematic review and meta-analysis. *Reviews in medical virology*. 2024;34(3):e2540.

114. Jiang W. Adverse pregnancy outcomes, non-retention, eligibility of differentiated care and virologic failure among women living with HIV in prevention of vertical transmission programs: University of Washington; 2023.

115. Ankunda C. Longitudinal Analysis of Viral Suppression Before, During, and After Pregnancy Among Women on Antiretroviral Therapy in Uganda: Six-year Real-World Evidence. 2025.

116. Ngoufack Jagni Semengue E, NKA AD, Tueguem PP, Ka'e CA, Molimbou E, Forgwei LY, et al. Gaps, challenges and opportunities towards achieving the 95-95-95 targets in Cameroon: A systematic review and meta-analysis protocol. *PloS one*. 2025;20(2):e0317850.

117. Mukuku O, Govender K, Kulohoma B, Wembonyama SO. Challenges in reaching the UNAIDS 95-95-95 targets in sub-Saharan Africa: status, innovations and pathways forward. *Frontiers in Public Health*; 2025. p. 1701425.

118. Nyongesa MK, Mwatasa MH, Kagonya VA, Mwambingu G, Ngetsa C, Newton C, et al. HIV virological non-suppression is highly prevalent among 18- to 24-year-old youths on antiretroviral therapy at the Kenyan coast. *BMC Infect Dis*. 2022;22(1):449.

119. United Nations Programme on HIV/AIDS (UNAIDS). Young people and HIV. accessed on: https://www.unaids.org/sites/default/files/media_asset/young-people-and-hiv_en.pdf 2021 [

120. Hlophe LD, Tamuzi JL, Shumba CS, Nyasulu PS. Barriers and facilitators to anti-retroviral therapy adherence among adolescents aged 10 to 19 years living with HIV in sub-Saharan Africa: A mixed-methods systematic review and meta-analysis. *PLoS One*. 2023;18(5):e0276411.

121. Han WM, Law MG, Egger M, Wools-Kaloustian K, Moore R, McGowan C, et al. Global estimates of viral suppression in children and adolescents and adults on antiretroviral therapy adjusted for missing viral load measurements: a multiregional, retrospective cohort study in 31 countries. *Lancet HIV*. 2021;8(12):e766-e75.

122. Ruel T, Mwangwa F, Balzer LB, Ayieko J, Nyabuti M, Mugoma WE, et al. A multilevel health system intervention for virological suppression in adolescents and young adults living with HIV in rural Kenya and Uganda (SEARCH-Youth): a cluster randomised trial. *The Lancet HIV*. 2023;10(8):e518-e27.

123. Johnson-Peretz J, Lebu S, Akatukwasa C, Getahun M, Ruel T, Lee J, et al. "I was still very young": agency, stigma and HIV care strategies at school, baseline results of a qualitative study among youth in rural Kenya and Uganda. *Journal of the International AIDS Society*. 2022;25:e25919.

124. Deeks SG, Lewin SR, Havlir DV. The end of AIDS: HIV infection as a chronic disease. *The lancet*. 2013;382(9903):1525-33.

125. Mengesha MM, Teshome A, Ajema D, Tura AK, Hallström IK, Jerene D. The association between HIV diagnosis disclosure and adherence to anti-retroviral therapy among adolescents living with HIV in Sub-Saharan Africa: A systematic review and meta-analysis. *Plos one*. 2023;18(5):e0285571.

126. Lamb MR, Fayorsey R, Nuwagaba-Biribonwoha H, Viola V, Mutabazi V, Alwar T, et al. High attrition before and after ART initiation among youth (15–24 years of age) enrolled in HIV care. *Aids*. 2014;28(4):559-68.

127. Salou M, Dagnra AY, Butel C, Vidal N, Serrano L, Takassi E, et al. High rates of virological failure and drug resistance in perinatally HIV-1-infected children and adolescents receiving lifelong antiretroviral therapy in routine clinics in Togo. *Journal of the International AIDS Society*. 2016;19(1):20683.

128. Duri K, Munjoma PT, Mazhandu AJ, Marere T, Gomo E, Banhwa S, et al. Predictors and timing to viral suppression in HIV-infected pregnant women in the university of Zimbabwe birth cohort study during the era of lifelong antiretroviral therapy (Option B+ Treatment Strategy). *Frontiers in Virology*. 2022;2:838234.

129. Dessie G, Wagnew F, Mulugeta H, Amare D, Jara D, Leshargie CT, et al. The effect of disclosure on adherence to antiretroviral therapy among adults living with HIV in Ethiopia: a systematic review and meta-analysis. *BMC Infect Dis*. 2019;19(1):528.

130. Endalamaw A, Assefa Y, Geremew D, Belete H, Dachew BA, Belachew A, et al. Disclosure of HIV seropositivity to sexual partner in Ethiopia: A systematic review. *Womens Health (Lond)*. 2021;17:17455065211063021.

131. Mekonnen FA, Lakew AM, Muchie KF, Teshome DF. Sero-positive HIV result disclosure to sexual partner in Ethiopia: a systematic review and meta-analysis. *BMC Public Health*. 2019;19(1):1743.

132. Jones HS, Floyd S, Stangl A, Bond V, Hoddinott G, Pliakas T, et al. Association between HIV stigma and antiretroviral therapy adherence among adults living with HIV: baseline findings from the HPTN 071 (PopART) trial in Zambia and South Africa. *Tropical Medicine & International Health*. 2020;25(10):1246-60.

133. Perger T, Davtyan M, Foster C, Evangeli M, Berman C, Kacanek D, et al. Impact of HIV-related stigma on antiretroviral therapy adherence, engagement and retention in HIV care, and transition to adult HIV Care in Pediatric and Young Adult Populations Living with HIV: a literature review. *AIDS and Behavior*. 2025;29(2):497-516.

134. Zhao S, Wang W, Li S, He J, Duan W, Fang Z, et al. The prevalence of low-level viraemia and its association with virological failure in people living with HIV: a systematic review and meta-analysis. *Emerging Microbes & Infections*. 2025;14(1):2447613.

135. Kibui NK. Factors Associated With Virological Non-suppression in Treatment-experienced HIV+ Patients in Kenya: a Populationwide Study: University of Nairobi; 2024.

136. Ministry of Health E. National Guideline for Prevention of Mother-to-child Transmission of HIV, Syphilis and Hepatitis B Virus. Access: https://www.prepwatch.org/wp-content/uploads/2022/07/National-Guideline-for-Prevention-of-Mother-to-child-Transmission-of-HIV-Syphilis-and-Hepatitis-B-Virus-2021.pdf 2021 [

137. Yang J, Zhao X, Li F. INSTIs-centered antiviral regimens for first-line treatment of HIV/AIDS: a network meta-analysis and cost-effectiveness analysis. *BMC Infect Dis*. 2025;25(1):604.

138. Fleșeriu T, Meliț LE, Mărginean CO, Pop AV, Văsieșiu AM. Maternal HIV Infection and Antiretroviral Therapy in Pregnancy: Implications for Vertical Transmission, Fetal Safety, and Long-Term Infant Outcomes. *Pathogens*. 2025;14(8).

139. Fassinou LC, Songwa Nkeunang D, Delvaux T, Nagot N, Kirakoya-Samadoulougou F. Adherence to option B + antiretroviral therapy and associated factors in pregnant and breastfeeding women in Sub-Saharan Africa: a systematic review and meta-analysis. *BMC Public Health*. 2024;24(1):94.

140. Sibiude J, Le Chenadec J, Mandelbrot L, Hoctin A, Dollfus C, Faye A, et al. Update of perinatal human immunodeficiency virus type 1 transmission in France: zero transmission for 5482 mothers on continuous antiretroviral therapy from conception and with undetectable viral load at delivery. *Clinical infectious diseases*. 2023;76(3):e590-e8.

141. Nachega JB, Marconi VC, van Zyl GU, Gardner EM, Preiser W, Hong SY, et al. HIV treatment adherence, drug resistance, virologic failure: evolving concepts. *Infect Disord Drug Targets*. 2011;11(2):167-74.

142. Daltro ACB, Almeida CS, Unfried AGC, de Aquino TR, Travassos AGÁ. Virological failure and adherence to antiretroviral therapy in adolescents and young adults living with human immunodeficiency virus. *Tropical Medicine & International Health*. 2023;28(3):162-74.

143. Gils T, Bossard C, Verdonck K, Owiti P, Casteels I, Mashako M, et al. Stockouts of HIV commodities in public health facilities in Kinshasa: Barriers to end HIV. *PLoS One*. 2018;13(1):e0191294.

144. Thomford NE, Mhandire D, Dandara C, Kyei GB. Promoting U ndetectable Equals U ntransmittable in Sub-Saharan Africa: Implication for Clinical Practice and ART Adherence. *International journal of environmental research and public health*. 2020;17(17):6163.

145. Jennings Mayo-Wilson L, Devoto B, Coleman J, Mukasa B, Shelton A, MacCarthy S, et al. Habit formation in support of antiretroviral medication adherence in clinic-enrolled HIV-infected adults: a qualitative assessment using free-listing and unstructured interviewing in Kampala, Uganda. *AIDS research and therapy*. 2020;17(1):30.

146. Colvin CJ, Konopka S, Chalker JC, Jonas E, Albertini J, Amzel A, et al. A systematic review of health system barriers and enablers for antiretroviral therapy (ART) for HIV-infected pregnant and postpartum women. *PloS one*. 2014;9(10):e108150.

147. Moriarty K, Genberg B, Norman B, Reece R. The effect of antiretroviral stock-outs on medication adherence among patients living with HIV in Ghana: a qualitative study. J*ournal of the Association of Nurses in AIDS Care*. 2018;29(2):231-40.

Table 1: Subgroup analysis for virologic suppression among HIV-positive pregnant and lactating women receiving antiretroviral therapy in Africa.

Parameters	Studies	VS (%) at 95% CI	I^2 (%)	P-value
**Study design**				
Cohort studies	27	82.74% (80.80, 84.69)	99.35%	0.000
Cross-sectional studies	28	79.34% (74.06, 84.63)	99.69%	0.000
**Country**				
Cameroon	2	91.89% (89.23, 94.55)	0.00	0.327
DR Congo	4	62.66% (58.56, 66.75)	92.49	0.000
Ethiopia	4	94.11% (91.16, 97.07)	94.97	0.000
Kenya	3	84.82% (74.86, 94.78)	92.80	0.000
Malawi	6	88.06% (85.62, 90.51)	80.81	0.000
SSA	3	66.28% (60.04, 72.51)	95.09	0.000
South Africa	21	82.8% (80.42, 85.21)	99.64	0.000
Uganda	3	66.99% (23.93, 110.05)	99.72	0.000
Rwanda	2	88.30% (81.15, 95.46)	93.31	0.000
Zimbabwe	2	82.58% (73.20, 91.97)	87.27	0.005
Others*	3	76.52% (69.68, 83.35)	71.31	0.031
**Regions of Africa**				
Central Africa	4	62.66% (58.56, 66.75)	92.49	0.000
East Africa	13	83.49% (77.69, 89.3)	99.09	0.000
Sub-Saharan Africa	4	66.28% (60.04, 72.51)	95.09	0.000

Southern Africa	31	83.74% (81.72, 85.76)	99.47	0.000
Western Africa	3	84.49% (72.77, 96.20)	94.24	0.000
**Study population**				
Pregnant women	38	81.15% (78.66, 83.63)	99.61	0.000
Postpartum women	9	80.61% (73.28, 87.94)	99.08	0.000
Both	8	80.21% (73.25, 87.18)	98.87	0.000

*Nigeria, Estiwani, Tanzania

Table 2: Meta-regression for factors correlated with virologic suppression among HIV-positive pregnant and lactating women receiving antiretroviral therapy in Africa.

Variables	β-coefficient	Std. error	z	P-value	95% CI
Publication year<br>After 2020 (Ref.)<br>Before 2020	-2.73	3.55	-0.77	0.443	-9.69, 4.24
ART regimen<br>Non-DTG based (Ref.)<br>DTG-based	16.43	6.55	2.51	0.012	3.58, 29.26
Country	-0.24	0.183	-1.33	0.183	-0.60, 0.12
Region of Africa	-2.92	1.40	-2.08	0.037	-5.66, -0.17
Sample size	-0.001	0.0002	-1.30	0.195	-0.0005, 0.0001
Constant	91.48	5.77	15.85	0.000	80.17, 102.80

Table 3: Meta-analysis for factors associated virologic suppression among HIV-positive pregnant and lactating women on antiretroviral therapy in Africa.

Variables	No_ studies	AOR (95% CI)	I^2 (%)	P-value
Maternal age<br>≥25 years (Ref.)<br>15-24 years	4	0.49 (0.32, 0.77)	79.7%	0.002
Disclosing HIV status to partner	4	1.66 (1.31, 2.11)	0.00%	0.04
ART type<br>First line ART<br>Second and/ third line ART (Ref.)	3	6.53 (1.93, 22.03)	86.07%	0.001
Adherence status to ART<br>Good<br>Poor (Ref.)	5	3.61 (1.18, 11.02)	94.6%	0.001

Figure

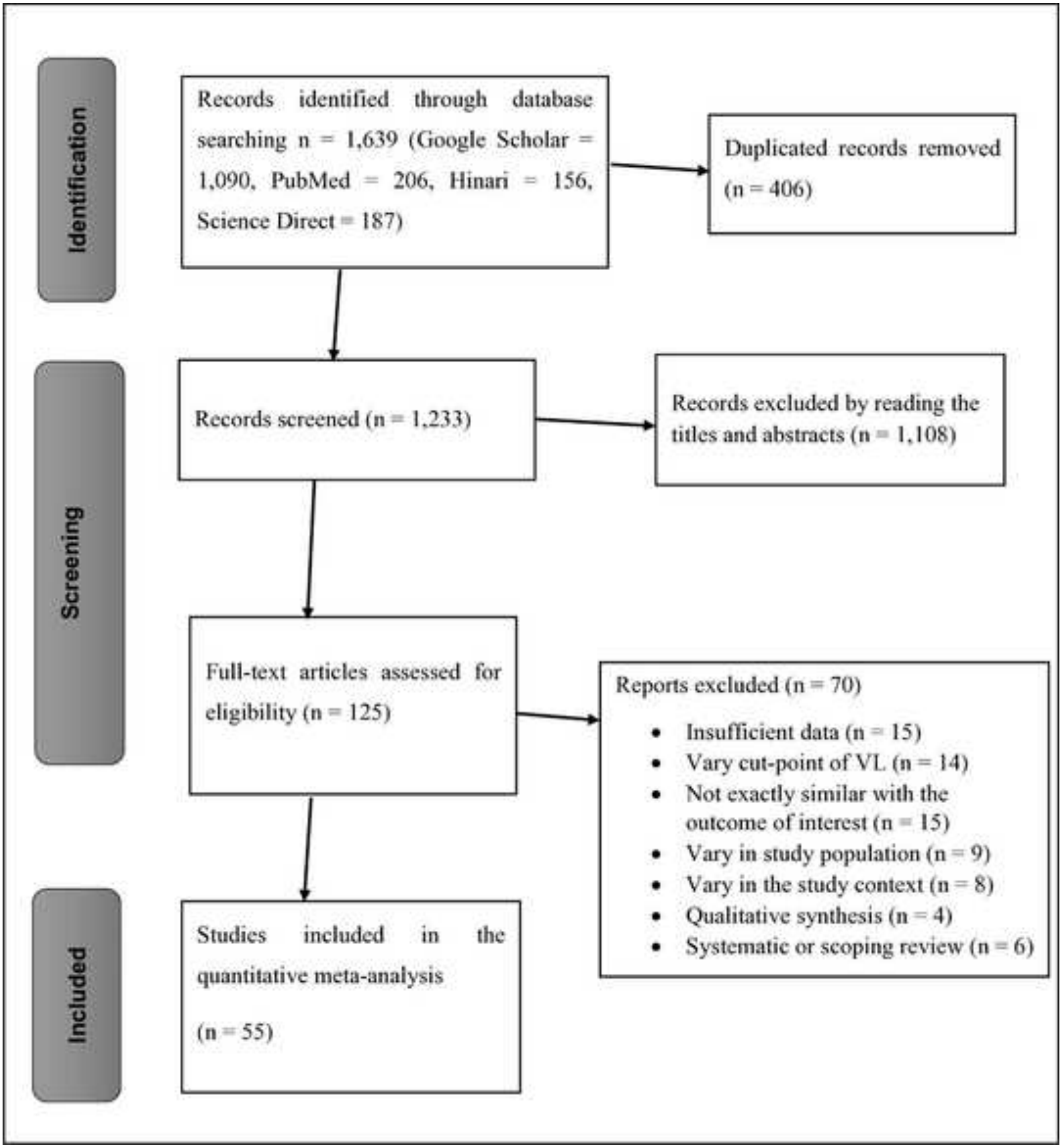
Identification
Screening
Included
Records identified through database searching n = 1,639 (Google Scholar = 1,090, PubMed = 206, Hinari = 156, Science Direct = 187)
Duplicated records removed (n = 406)
Records screened (n = 1,233)
Records excluded by reading the titles and abstracts (n = 1,108)
Full-text articles assessed for eligibility (n = 125)
Reports excluded (n = 70)
• Insufficient data (n = 15)
• Vary cut-point of VL (n = 14)
• Not exactly similar with the outcome of interest (n = 15)
• Vary in study population (n = 9)
• Vary in the study context (n = 8)
• Qualitative synthesis (n = 4)
• Systematic or scoping review (n = 6)
Studies included in the quantitative meta-analysis (n = 55)

Figure

Click here to access/download;Figure;Fig 2 Forest plot.tif

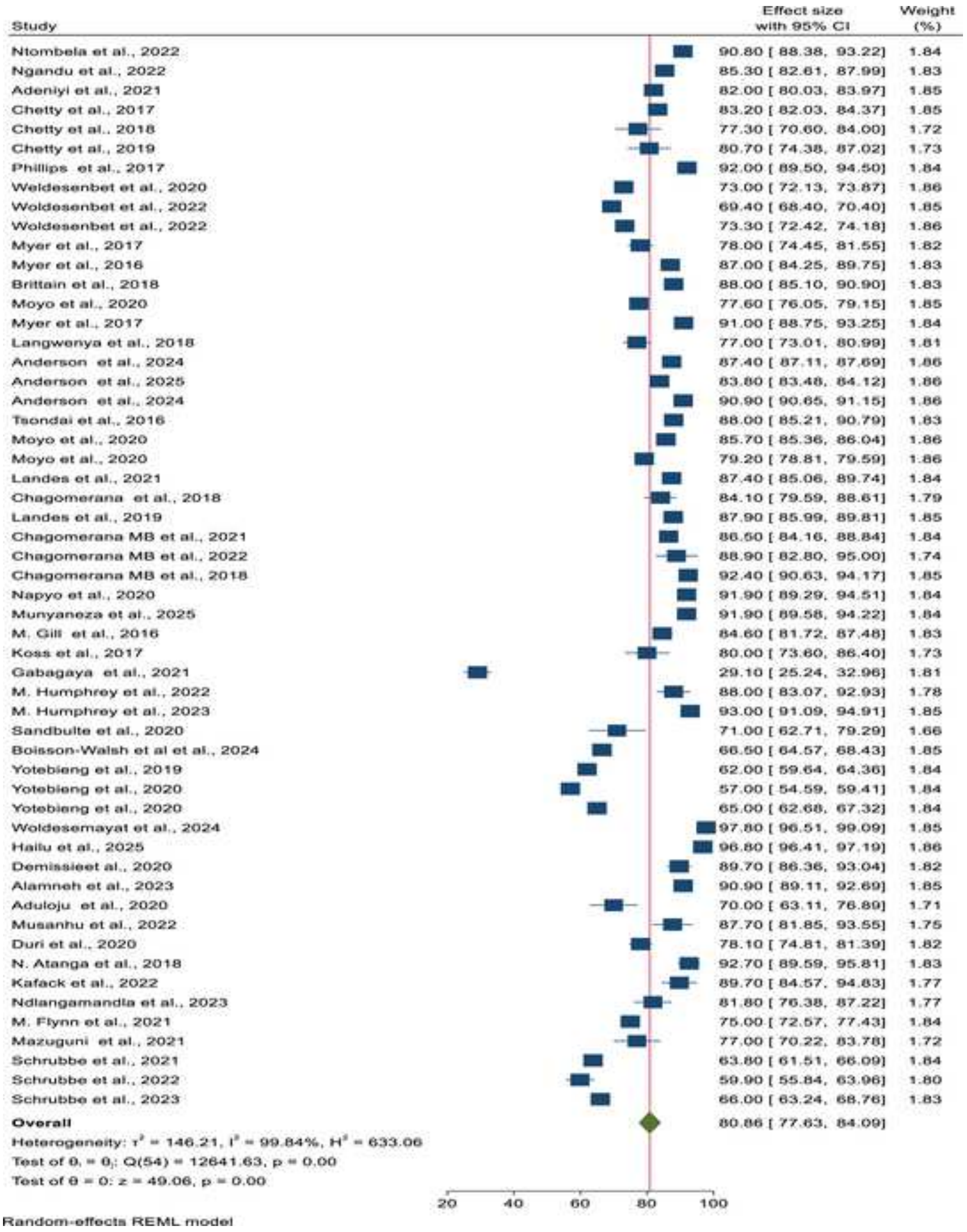

Figure

Click here to access/download;Figure;Fig 3 Funnel plot.tif

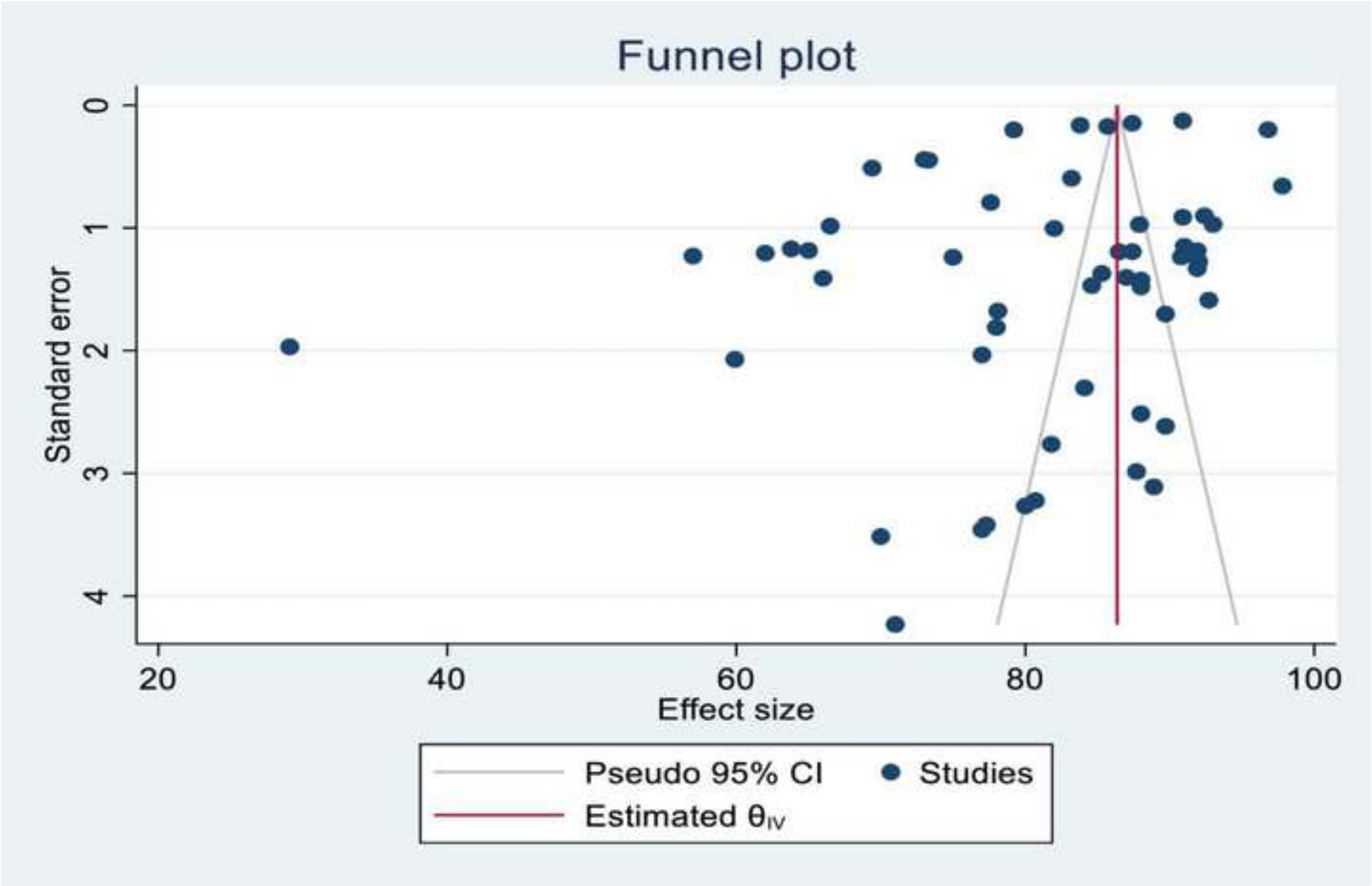

Figure

Click here to access/download;Figure;Fig 4 Sensitivity analysis.tif

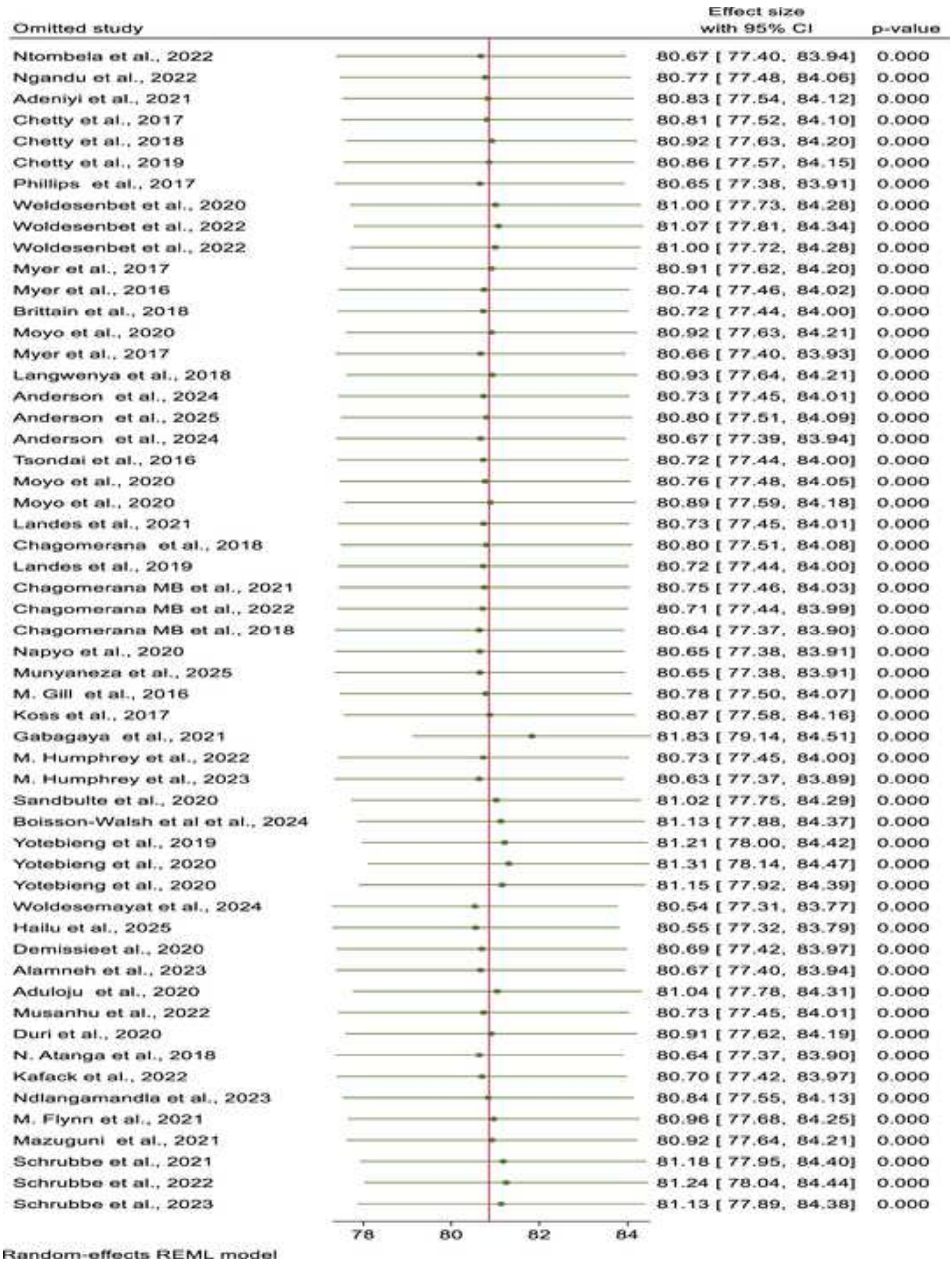

Click here to access/download
Supporting Information
S1 file PRISMA Checklist 2020.docx

Click here to access/download
Supporting Information
S2 file Search Strategies.docx

Click here to access/download
Supporting Information
S3 file Summary for studies.docx

Click here to access/download
Supporting Information
S4 file Associated factors for virologic suppression.docx

Virologic suppression among HIV-positive pregnant and lactating women receiving antiretroviral therapy in Africa: A systematic review and meta-analysis

Berihun Agegn Mengistie (BAM)[1*], Getie Mihret Aragaw (GMA)[1], Gebrye Gizaw Mulatu (GGM)[2], Kindu Yinges Wondie (KYW)[3], Alemneh Tadesse Kassie (ATK)[3], Alemken Eyayu Abuhay (AEA)[4], Wondimnew Mersha Biset (WMB)[5], Moges Tesfa Tsega (MTT)[6], Abay Eyayu Asrie (AEA)[7], Tazeb Alemu Anteneh (TAA)[3], Habtu Kifle Negash (HKN)[8], Eshet Gebrie (EG)[9], Nuhamin Tesfa Tsega (NTT)[10]

[1]Department of General Midwifery, School of Midwifery, College of Medicine and Health Sciences, University of Gondar, Gondar, Ethiopia, [2]Department of Health Informatics, College of Medicine and Health Sciences, University of Gondar, Gondar, Ethiopia, [3]Department of Clinical Midwifery, School of Midwifery, College on Medicine and Health Sciences, University of Gondar, Gondar, Ethiopia, [4]University of Gondar Comprehensive Specialized Hospital, Gondar, Ethiopia, [5]Department of Anesthesiology, School of Medicine, Saint Paul's Hospital Millennium Medical College, Addis Ababa, Ethiopia, [6]Department of Internal Medicine, School of Medicine, College of Medicine and Health Sciences, University of Gondar, Gondar, Ethiopia, [7]Department of Obstetrics and Gynecology, College of Medicine and Health Sciences, University of Gondar, Gondar, Ethiopia, [8]Department of Human Anatomy, School of Medicine, College of Medicine and Health Sciences, University of Gondar, Gondar, Ethiopia, [9]Department of Clinical Chemistry, School of Biomedical and Laboratory Sciences, College of Medicine and Health Sciences, University of Gondar, P.O. Box 196, Gondar, Ethiopia, [10]Department of Women's and Family Health, School of Midwifery, College on Medicine and Health Sciences, University of Gondar, Gondar, Ethiopia

Authors' addresses

BAM: berihunagegn21@gmail.com

GMA: getie895@gmail.com

GGM: gebrye.gizaw@gmail.com

KYW: Kinduyinges2010@gmail.com

ATK: alexotadesse.79@gmail.com

AEA: alexeyayu6@gmail.com

WMB: Wondemer2096@gmail.com

MTT: mogestesfa46@gmail.com

AEA: eyayuabay3@gmail.com

TAA: tazebalemu05@gmail.com

HKN: habtuk8@gmail.com

EG: eshetgebrie@gmail.com

NTT: nuha26tesfa@gmail.com

BAM Corresponding author*: berihunagegn21@gmail.com (Corresponding author)

Abstract

Background: The elimination of mother-to-child transmission of human immunodeficiency virus (HIV) is a key global public health priority. To stop the vertical transmission of HIV and lower the risk of maternal morbidity and mortality, it is important to achieve viral load suppression. Achieving viral load suppression is essential for preventing vertical transmission of HIV and reducing associated maternal morbidity and mortality. While many African countries have adopted the global 95-95-95 targets, comprehensive data on virologic suppression among pregnant and lactating mothers across the continent remains limited. The objective of this systematic review and meta-analysis was to determine the pooled estimate of virologic suppression and to examine the factors associated with it among HIV-positive pregnant and lactating women on antiretroviral therapy in Africa.

Methods: This study followed the Preferred Reporting Items for Systematic Reviews and Meta-Analyses (PRISMA) guidelines. The study protocol was registered in the International Prospective Register of Systematic Reviews (PROSPERO; CRD420251186121 CRD420251077063). We carried out a thorough systematic review by examining PubMed, ScienceDirect, Hinari, and Google Scholar for relevant studies. Data from the studies were retrieved using an Excel sheet and analyzed with STATA version 17. The Joanna Briggs Institute appraisal tool was used to evaluate the methodological quality of studies. A random-effects model with Restricted maximum–likelihood (REML) (Der Simonian- Laird method) was applied to calculate determine the pooled prevalence of virologic suppression (viral load threshold <1000 copies/ml) among pregnant and lactating mothers in Africa. A funnel plot and the Egger's test were used to investigate publication bias. Statistical heterogeneity was assessed using the I^2 statistic and Cochrane's Q test.

Results: A total of 55 eligible studies conducted in Africa, comprising 304,883 participants, were included in the quantitative meta-analysis. Accordingly, the overall prevalence of virologic suppression among HIV-positive pregnant and breastfeeding women in Africa was 80.86% (95% CI: 77.63%, 84.09% ~~80.92% (95% CI: 78.92% 82.91%~~, I^2 = 99.84%). In contrast, the pooled estimate for achieving an undetectable viral load was substantially lower, at 60.92% (95% CI: 52.46%, 69.39%; I^2 = 99.91%). Virological suppression was significantly associated with women's age (15-24 years) (AOR = 0.49; 95% CI: 0.32-0.77), disclosure of HIV status to a partner (AOR = 1.66; 95% CI: 1.31-2.11), first-line antiretroviral therapy regimen (AOR = 6.53; 95% CI: 1.93-22.06), and good antiretroviral drug adherence (AOR = 3.61; 95% CI: 1.18-11.02). In addition, other socio-demographic variables, higher educational level, being married/cohabitant, urban residency, healthcare utilization (time of ANC booking, time of ART initiation, duration of ART), fear of stigma, distance to health facility, shortage of health professionals, ART drug stock-out, and lack of HIV care commodities were significantly associated with virologic suppression among HIV-positive pregnant and lactating women in Africa.

Conclusion: The overall pooled estimate of virologic suppression among HIV-positive pregnant and breastfeeding women in Africa ~~remains low~~was approximately 81%, ~~compared with the~~below the global target ~~for~~ of 95% vir~~o~~a~~l~~logical suppression. This emphasizes the necessity of targeted strategies for younger HIV-positive women, disclosing HIV status, initiating first-line antiretroviral regimens, and promoting antiretroviral treatment adherence. Upgrading health care systems to enable regular viral load monitoring, as well as addressing socio-demographic and antiretroviral therapy-related variables, are vital steps towards attaining and sustaining virologic suppression in these groups of population, ultimately assisting in achieve elimination of MTCT of HIV. ~~reduced viral load and improved treatment outcomes.~~

Keywords: Africa; Antiretroviral therapy; HIV; pregnant women; lactating women; virologic suppression

Introduction

Human immunodeficiency virus (HIV) is one of the world's most critical public health challenges, affecting millions of people [1, 2]. According to the Joint United Nations Programme on HIV/AIDS (UNAIDS) and World Health Organization (WHO) report, approximately 40.8 million people worldwide were infected with HIV, of which 21.0 million were women (15+ years); 1.3 million new HIV cases were reported, and 630,000 people died from AIDS-related illnesses at the end of 2024. In this report, nearly 1.4 million children aged below 15 years were living with HIV [1, 2]. Sub-Saharan African countries were responsible for 65% (an estimated 26.3 million) of the worldwide HIV burden [1, 2]. Women and girls of all ages contributed to 44% of all new HIV infections globally in 2023 [3].

~~Human immunodeficiency virus (HIV) is one of the world's most critical public health challenges, affecting millions of people. In 2024, 39.9 million people worldwide were infected with HIV, 1.3 million new HIV cases were reported, and 630,000 people died from AIDS-related illnesses~~ [3, 4]~~.~~ ~~Sub-Saharan African countries were responsible for 65% of the worldwide HIV burden. Women and girls of all ages contributed to 44% of all new HIV infections globally in 2023~~ [3]~~. According to the Joint United Nations Programme on HIV/AIDS (UNAIDS) 2024 report, nearly 120,000 children aged 0-14 were infected with HIV for the first time~~ [3]~~.~~

The primary route of HIV infection in children is mother-to-child transmission (MTCT) of HIV during pregnancy, childbirth, and postpartum periods through breastfeeding ~~also referred to as vertical transmission~~ [5]. It accounts for more than 90% of all newly acquired pediatric HIV infections worldwide [6]. Without intervention, the overall MTCT of HIV incidence ranges from

15% to 45% [7]. However, appropriate interventions during pregnancy, labor, and breastfeeding can lower the risk of perinatal HIV transmission to less than 2% in non-breastfeeding and 5% in breastfeeding populations, respectively [8, 9].

The World Health Organization (WHO) is committed to a global public health mission that focuses on expanding access to HIV testing, antiretroviral therapy (ART), and treatment monitoring, aiming to enhance clinical management of HIV, achieve sustained viral suppression, and lowering HIV-related morbidity, and mortality [10]. Maternal plasma HIV viral load (VL) status is the strongest determinant of HIV transmission from mother to child (MTCT) [11-13]. Nowadays, viral load assessment has become the main approach for monitoring HIV patients' clinical and virologic outcome to ART [14]. HIV viral load is categorized into three main stages: unsuppressed (>1000 copies/mL), suppressed (detected but ≤1000 copies/mL), and undetectable (≤50 copies/ml or not detected by the assay used) [10, 11, 15]. In 2021, the World Health Organization revised its HIV treatment monitoring algorithm to enhance support for people living with HIV in attaining viral suppression, with the ultimate aim of maintaining an undetectable viral load [10]. Achieving HIV viral suppression is crucial for enhancing individual health, preventing sexual transmission, and lowering the risk of perinatal transmission [10]. In the contrary, ~~P~~pregnant and lactating women who do not achieve virologic suppression (VS) are at a higher risk of HIV-related complications, death, and vertical transmission to their infants [11, 16].

~~Maintaining viral load suppression (≤1000 copies/ml) in HIV positive pregnant and postpartum women is a global public health goal for improving maternal health and producing HIV free children~~ [13, 17]~~. Viral load suppression (VLS) is an important indicator of antiretroviral therapy's (ART) effectiveness.~~

Studies show that achieving and maintaining VS among pregnant and postpartum WLWH remains to be challenging, despite significant progress in the scaling up of ART via prevention of mother-to-child transmission (PMTCT) initiatives [18-20]. Several countries have been implementing the prevention of mother-to-child transmission of HIV (PMTCT) program~~s~~ packages [21-23]. ~~are implemented by a number of interventions, such as~~ It includes lifetime ART for pregnant and breastfeeding women, preventive antiretroviral (ARV) medications for HIV-exposed infants (HEIs), serial testing for HEIs, and prompt ART initiation for children who acquire HIV [5, 24]. Maintaining virologic suppression (<1000 copies/ml) in HIV-positive pregnant and postpartum women is a global public health goal for improving maternal health and producing HIV-free children [13, 17].

Antiretroviral therapy (ART) as part of PMTCT is an important strategy to combat the global AIDS crisis and ~~to~~ lower vertical transmission rates [25]. The WHO guidelines indicated that ART should be initiated in all pregnant and breastfeeding HIV-positive women, regardless of their VL, clinical stage, or CD4 cell count [16]. The fixed-dose combination (FDC) of tenofovir disoproxil fumarate (TDF), lamivudine (3TC), and dolutegravir (DTG) is the ideal first-line regimen for the treatment of HIV infection in adults and adolescents, including pregnant and breastfeeding women [26]. Timely initiation and consistent adherence to ART are key predictors for achieving virologic suppression, specifically undetectable HIV viral load and preventing mother-to-child transmission of HIV, mitigating a range of clinical, virological, and immunological risks [16, 27]. Additionally, viral suppression enhances health outcomes for HIV-positive women, notably decreased illness, longer life expectancy, and better birth outcomes [28-30]. ~~Factors associated with virologic non-suppression include suboptimal adherence, medication intolerance, drug–diet interactions, low CD4 cell counts, prior treatment history, and antiretroviral drug resistance~~ [31]~~.~~

~~To tackle the continuing HIV crisis, WHO, the Global Fund, and UNAIDS have established global HIV plans that support the achievement of SDG target 3.3, aiming to end the HIV epidemic by 2030~~ [4]~~.~~ To tackle the continuing HIV crisis, the UNAIDS program is striding ~~working~~ toward a 95-95-95 global target, ensuring that 95% of individuals living with HIV are aware of their HIV status, 95% of those diagnosed initiate life-long ART, and at least 95% of patients beginning ART achieve virologic suppression ~~aiming at eliminating the global epidemic by 2030~~ [32-34]. ~~These universal aims include ensuring that 95% of individuals living with HIV are aware of their HIV status, 95% of those diagnosed obtain continuous ART medication, and at least 95% of patients beginning ART achieve viral suppression~~ [33]~~.~~ However, in many of the SSA countries, ~~viral load~~virologic suppression is still behind the UNAIDS 95-95-95 targets [34-36].

In Africa, ART treatment failure or virologic failure among HIV-positive individuals continues to be a significant public health concern [37-39]. Similarly, the rate of virologic suppression among pregnant and lactating women is still unsatisfactory throughout the continent [18, 40].~~Broadly~~In Africa, previous study findings on the proportion of ~~viral load~~virologic suppression among pregnant and breastfeeding women living with HIV ~~in Africa~~ revealed inconsistent and equivocal results that ranged from 29.1% to 97.8% [41, 42]. A mount of evidence have shown that sociodemographic variables, adherence to ART, ART regimens, enhanced counseling, baseline viral load status, duration of ART, disclosing HIV status, medication tolerance, drug–diet interactions, CD4 cell counts, prior treatment history, and antiretroviral drug resistance were found significantly associated with virologic suppression [31, 38, 43-45].

Achieving the third target of the UNAIDS 95-95-95 goals, ensuring that 95% of PLHIV on ART achieve sustained VLS, is essential to curbing HIV transmission and improving health outcomes

[46]. Individual-level variables, maternal healthcare utilization, structural barriers, health system factors, and socioeconomic factors are the main challenges in achieving this goal [45-50]. ~~Women in most African countries frequently experience major challenges to accessing healthcare due to socioeconomic barriers, caregiver responsibilities, gender based discrimination, and low economic freedom~~ [45, 49-51]~~. This could have been associated with a greater viral load in pregnant and nursing women.~~

~~However~~In addition, there is a lack of comprehensive ~~study~~ evidence on virologic suppression among pregnant and breastfeeding women at the national and regional levels in Africa. Thus, the goal of this systematic review and meta-analysis is to assess the pooled prevalence of virologic suppression and identify associated factors among HIV-positive pregnant and breastfeeding women receiving ART in Africa. This comprehensive data will assist health professionals, policymakers, and guideline developers in implementing evidence-based strategies to improve viral load suppression in these populations, hence reducing HIV perinatal transmission.

~~Research questions~~

1. ~~What is the pooled estimate of virologic suppression among HIV-positive pregnant and lactating women receiving antiretroviral therapy in Africa?~~
2. ~~What are the determinant factors of virologic suppression among HIV-positive pregnant and lactating women receiving antiretroviral therapy in Africa?~~

Methods and materials

Study protocol and search strategy

This systematic review adhered to the Preferred Reporting Items for Systematic Reviews and Meta-Analysis (PRISMA) standards [52] **(S1 file)**. The study protocol for the review has been registered on PROSPERO with reference ID CRD420251186121 and available on: https://www.crd.york.ac.uk/PROSPERO/view/CRD420251186121. A rigorous and comprehensive review of Google Scholar, PubMed, Hinari, and ScienceDirect for original research on virologic suppression and associated factors conducted in Africa. The inclusion and exclusion criteria were established based on the condition, context, and study population (Co Co Pop) approach. Previous studies that met the eligibility criteria were retrieved.

A search strategy was developed for the databases by combining keywords with Boolean operators. Ultimately, we employed the following search term combination: “viral load suppression”, “virologic suppression”, “viral suppression”, “HIV viral load non-suppression”, “viral load”, “detectable viral load”, “undetectable viral load”, “virological suppression”, “HIV viral load”, “plasma viremia”, “HIV viral suppression”, “HIV viremia”, “HIV-positive women”, pregnant, “lactating mothers”, “postpartum women”, “breastfeeding mothers”, "antiretroviral therapy", ART and Africa. To obtain additional articles from the citation list of publications found in the databases that were accessible, snowballing techniques were also employed **(S2 file)**.

Eligibility criteria

Inclusion criteria

Condition: The condition of interest was virologic suppression among HIV-positive pregnant and lactating women receiving antiretroviral therapy in Africa.

Context: All primary studies that reported the prevalence and/or associated factors of virologic suppression in the Africa context.

Population: The study population for this review is pregnant and lactating mothers living with HIV on ART.

Study design: All primary observational studies, including cross-sectional, case-control, and cohort studies that reported the prevalence and/or associated factors virologic suppression among HIV-positive pregnant and lactating women receiving ART.

Publication year: Both published and unpublished articles between January 1, 2016, and April 30, 2025, were included.

Exclusion criteria

Articles that failed to report the outcome of interest, narrative reviews, qualitative reviews, expert comments, case reports, editorials, letters, and methodological studies were excluded from the review.

Measurement of outcome variables

This study aimed to determine the pooled prevalence of virologic suppression among African pregnant and lactating women receiving antiretroviral therapy in Africa. This study's second objective was to determine the variables that are associated with virologic suppression in these key population ~~Africa~~. The World Health Organization (WHO) defined virological suppression as when viral load copies become ≤1,000 copies/mL of blood after six months of ART initiation [10, 53]. However, virologic failure (VF) is defined as a VL persistently over 1000 copies/ml~~L~~ in two consecutive measurements, ~~3 months apart,~~ after 6 months (3 months apart) on ~~ART and~~ enhanced ART adherence support. In this systematic review and meta-analysis, we employed a viral load threshold of ≤1,000 copies/ml (virologic suppression), which is consistent with the WHO guideline criteria utilized in standard clinical decision-making [18, 53, 54].

Quality assessment for included studies

The quality of the included primary studies was evaluated independently by two authors (BAM and NTT). Each study was assessed using the Critical Appraisal Checklist developed by the Joanna Briggs Institute (JBI). Twenty-eight of the included papers were evaluated using the 9-item cross-sectional checklist, while 27 used the 11-item cohort checklist [55, 56]. Studies with a JBI checklist score of five or above were regarded as being of acceptable quality, indicating a low likelihood of bias. ~~Studies with a JBI checklist score of five or higher were deemed to be of excellent quality or to have minimal likelihood of bias.~~ Any disputes between the two authors were settled by open conversation and consultation with GMA, the third author**.**

Data extraction and management

Data extraction from the included publications was performed independently by two authors (BAM and NTT) using a standardized abstraction form developed in Excel. In accordance with the inclusion and exclusion criteria, all identified studies were imported into the reference manager EndNote 20. After screening titles and abstracts, full texts were reviewed for eligibility. Disagreements were resolved through discussion with a third reviewer (GMA). To avoid duplication, studies with overlapping or identical data were excluded. For eligible studies, the following information was extracted: identification details (first author's last name and year of publication), proportion of virologic suppression, factors associated with virologic suppression, adjusted odds ratios with 95% confidence intervals, study area, study design, study population, sample size, and risk of bias assessment method~~-~~ (S3 file).

Data synthesis and statistical analysis

All statistical data analyses were carried out after the data were collected using a Microsoft Excel spreadsheet and exported to the statistical program STATA version 17. Texts, tables, and forest plots were used to display the extracted data. A binomial distribution was used to determine each

study's standard error of prevalence of the outcome variable. The Higgins I-squared (I^2) test was used to check for heterogeneity in the pooled prevalence of the studies. The heterogeneity among the included studies was classified as low, moderate, or high based on I^2 values of <25%, 50–75%, and >75%, respectively [57].

A random-effects model with Restricted maximum–likelihood (REML) ~~(Der Simonian and Laird's method)~~ was used to determine the pooled prevalence of virologic suppression in Africa. REML is preferred over the Der Simonian and Laird method because it employs a likelihood-based approach, which typically offers a more stable and unbiased estimate of between-study variance (τ^2) and more accurate confidence interval coverage, especially in the presence of high heterogeneity. Even though, Der Simonian and Laird's method offer a narrower CI, it underestimates the between-study variance (τ^2) and inflates type I error (false positive) under extreme heterogeneity, which affect the robustness of the pooled estimate [58, 59].

A subgroup analysis and meta-regression were~~as~~ conducted across the publication year, country, regions of Africa, ART regimen, and study population ~~the country and study population~~ to identify potential sources of study heterogeneity. In addition, we performed a leave-one-out sensitivity analysis to look at how particular studies influence the pooled estimate. The pooled estimates from across the continent were then displayed in forest plots and tables, along with their corresponding 95% confidence intervals. Potential small-study effects were assessed using funnel plots and Egger's regression test. Visually, publication bias has been assessed using a funnel plot (23). Statistically, potential publication bias was evaluated using Egger's and Begg's tests, with a p-value < 0.05 suggesting evidence of publication bias [60]. Finally, we conducted a meta-analysis and systematic synthesis to examine the determinants of virologic suppression among pregnant

and lactating women receiving ART in Africa. Finally, the pooled adjusted odds ratios (AORs) with 95% confidence intervals (CI) were presented in forest plots.

Formatted: Font: (Default) +Body (Aptos), Font color: Text 1

Results

Study selection and characteristics of the included studies

All searcheding databases, including Google Scholar, yielded a total of 1,639 research articles; 406 duplicate records were deleted, and the remaining 1,233 papers were further screened. However, the majority of papers (n = 1,108) were removed after reading their titles and abstracts. The remaining 125 full-text articles were then reviewed for eligibility criteria, and 70 studies were excluded for a variety of reasons, including insufficient data, a difference in the VLS cut-point, variation in the study context, being unrelated to the outcome of interest, systematic reviews, and qualitative reviews. Then, the remaining 55 eligible studies with a total of 304,883 HIV-positive pregnant and breastfeeding women were included in the final quantitative meta-analysis [18, 29, 45, 54, 61-99]. Among the included studies, 26 articles had reported undetectable VL threshold (VL <50 copies/ml). In terms of study distribution across Africa, 31 and 13 studies were undertaken in Southern Africa and Eastern Africa, respectively **(Fig. 1).**

Figure 1: PRISMA flow diagram showing the selection process of studies for the virologic suppression among HIV-positive pregnant and lactating women receiving ART in Africa.

Prevalence of virological suppression among pregnant and lactating mothers receiving ART in Africa

In this meta-analysis, the pooled prevalence of virological suppression among pregnant and lactating women receiving ART in Africa was 80.86% (95% CI: 77.63%, 84.09%, $I^2 = 99.84\%$, $\tau^2 = 146.21$, p-value = 0.000)80.92% (95% CI: 78.92%, 82.91%). Additionally, the pooled estimate

showed that only 60.92% (95% CI: 52.46%, 69.39%, I^2 = 99.91%) of participants achieved an undetectable viral load. statistical test indicated the presence of significant heterogeneity among the included studies (I^2 = 99.57%, p-value = 0.000). As a result, a random-effects model, subgroup analyses, and sensitivity analyses were performedSubgroup analyses were conducted to explore potential sources of heterogeneity, such as differences in study setting, population characteristics, and study design. Additionally, sensitivity analyses were performed to assess the robustness of the pooled prevalence and to determine whether any single study had a disproportionate influence on the overall results **(Fig. 2)**.

Figure 1: A forest plot that shows the pooled prevalence of virologic suppression among HIV-positive pregnant and lactating women receiving antiretroviral therapy in Africa.

The 95% prediction interval (PI) was 57.4% to 95.5%, indicating that the effect size of a new study conducted in comparable setting could plausibly fall within this CI. Even though the pooled prevalence offers a useful overall summary, the wide prediction interval indicates substantial between-study heterogeneity. Accordingly, the pooled estimate should be interpreted as a descriptive summary of existing studies rather than a precise estimate for all settings. Due to the substantial heterogeneity among studies, a median-based summary was employed as an alternative statistical method. Consequently, the median prevalence across studies was 84.1% (IQR: 75.5%, 89.7%), providing a robust, non-parametric summary that is not influenced by extreme values or high variability among studies.

Heterogeneity and subgroup analysis

Subgroup analysis was performed based on the country, the regions of Africa, and the study population. Accordingly, Ethiopia had the highest prevalence of VS, 94.11% (95% CI: 91.16, 97.07), while the Democratic Republic of Congo had the lowest prevalence, 62.66% (95% CI:

58.56, 66.75). Moreover, the lowest overall prevalence of VS in pregnant and lactating mothers living with HIV was found in the Central Africa region (62.66%; 95% CI: 58.56, 66.75), whereas the highest was in Western and East Africa, which found nearly 84% of viral suppression. In terms of the study population, 81% of pregnant and postpartum mothers had VS, which is nearly the same pooled prevalence. Even though subgroup analysis was performed based on the previously listed parameters, no noticeable reduction in heterogeneity was observed in the pooled estimate of virological suppression. Overall, the subgroup analysis demonstrated substantial geographic variation in virologic suppression across Africa, highlighting differences in ART program effectiveness and context-specific variables. This significant between studies heterogeneity suggests that multiple factors, such as variations in ART regimens, adherence levels, health system capacity, availability of viral load monitoring, and sociocultural barriers, contribute to the observed differences in virologic suppression **(Table 1~~2~~)**.

Publication bias

The presence of publication bias was graphically checked using a funnel plot, and it was confirmed using Egger's test. The symmetrical appearance of the funnel plot in this analysis indicates that there is no publication bias among the studies **(Fig. 3)**. Egger's (p-value = 0.2402~~0.0512~~) test were statistically non-significant, suggesting that there was no substantial publication bias in the included studies.

Figure 2: A funnel plot test that indicates the virologic suppression among HIV-positive pregnant and lactating women receiving antiretroviral therapy in Africa.

Sensitivity analysis

To examine the impact of a single study on the estimated effect size, a leave-one-out sensitivity analysis was conducted using the random-effects model. The results, however, demonstrate that

the pooled estimate was not substantially impacted by a single study, and the point estimate of the excluded study is within the confidence interval of the overall estimate of virologic suppression. This demonstrated that the average estimate of virologic suppression among HIV-positive pregnant and breastfeeding mothers in Africa was robust **(Fig. 4)**.

Figure 4: A leave-one-out sensitivity analysis of virologic suppression among HIV-positive pregnant and lactating women on antiretroviral therapy in Africa.

Meta-regression analysis

A meta-regression analysis, together with subgroup and sensitivity analyses, was conducted to identify potential sources of heterogeneity. Study-level characteristics, including publication year (before vs. after 2020), ART regimen, country, African region, and sample size, were entered as covariates in the model. The analysis showed that use of a DTG-based ART regimen and the African region were significant moderators, accounting for part of the observed heterogeneity across studies. Thus, DTG-based ART regimens demonstrated significantly higher virologic suppression compared to non-DTG-based regimens ($\beta = 16.43$, $p = 0.012$). This suggests that variations in ART regimens had a significant role in the observed heterogeneity between studies, with DTG-based therapy linked with significantly associated with virologic suppression. Furthermore, the region of Africa was substantially linked with viral load suppression ($\beta = -2.92$, $p = 0.037$), indicating considerable regional variations in virologic suppression rate. This study suggests that contextual factors related to geographic location, such as disparities in health system capability, implementation of ART programs, and VL monitoring, influence to the observed between-study heterogeneity **(Table 2)**.

Factors associated with virological suppression among pregnant and lactating mothers receiving ART in Africa

This systematic review and meta-analysis investigated 16~~1~~ studies that identified factors associated with virologic suppression among pregnant and lactating mothers receiving ART in Africa [18, 19, 45, 62, 63, 66, 67, 76, 78, 81-83, 89, 91, 100]. In general, virologic suppression among HIV-positive pregnant and lactating women was substantially correlated with sociodemographic variables (women's age, higher educational level, being married/ cohabitants, urban residency) [18, 19, 45, 54, 62, 67, 76, 100-102], healthcare utilization (time of ANC booking and ART initiation, regimen and duration on ART, baseline VL threshold, adherence status of ART) [19, 41, 45, 62, 67, 100, 101, 103], accessibility and availability of healthcare services (long distance to health facility, shortage of health professionals, inadequate counseling, ART drugs running out, sub-optimal sample transportation) [13, 104, 105].

The odds of having virologic suppression were higher among women living in urban compared with rural resident women [67, 100, 102]. Furthermore, women's educational status was significantly associated with VS. Evidence from different studies consistently showed that women with secondary school or above had higher VS compared to women with no formal education [67, 76].

Virologic suppression was not achieved among women who started ANC booking or initiated ART in second or third trimester [45, 67, 100, 103]. Similarly, long distance to reach health facility, shortage of health provider, inadequate counseling, ART drugs running out, and sub-optimal sample transportation linked with failure of virologic suppression [13, 104, 105]. In addition, advanced WHO stage of HIV (clinical stage II, III, IV) [41, 103] and fear of stigma and discrimination [13, 104, 105], and disclosure of HIV status [18, 41, 100, 106-108] were found significantly associated with virologic suppression among HIV-positive pregnant and lactating women receiving ART **(S4 file).**

~~Thereby, virologic suppression among HIV-positive pregnant and lactating women was substantially correlated with younger age (15–24 years), being unmarried, first-line ART use, HIV status disclosure to a partner, and good adherence to ART.~~

In this review, younger age women (15–24 years) (AOR = 0.49; 95% CI: 0.32–0.77) were 51% less likely to attain virologic suppression compared to those aged 25 years and older [18, 67, 101]~~(Fig. 5)~~. Furthermore, a pooled analysis of three studies found that women who disclosed their HIV status (AOR = 1.66; 95% CI: 1.31, 2.11) were 1.66 times more likely to achieve virologic suppression compared to their counterparts ~~(Fig. 6)~~. In addition, the likelihood of achieving viral load suppression was 6.5 times higher for pregnant and lactating women on a first-line ART regimen (AOR = 6.53; 95% CI: 1.93, 22.06) than for those on second- or third-line regimens ~~(Fig. 7)~~. Finally, the odds of virologic suppression among pregnant and lactating women who had good ART adherence (AOR = 3.61, 95% CI: 1.18, 11.02) were 3.61 times more likely as compared to those who had poor adherence to ART (**Table 3**~~**Fig. 8**~~).

Discussion

The main objectives of this systematic review and meta-analysis were to determine the proportion of virologic suppression among pregnant and lactating women receiving ART and identifying factors associated with it. Thus, the pooled prevalence of virologic suppression among pregnant and lactating women taking ART in Africa was 80.86% (95% CI: 77.63%, 84.09%)~~80.92% (95% CI: 78.92%, 82.91%)~~. Additionally, the pooled estimate showed that only 60.92% of participants achieved an undetectable viral load (95% CI: 52.46%, 69.39%; $I^2 = 99.91\%$). This conclusion was comparable with a study conducted in East Africa, which found 80.6% ~~viral load~~virologic suppression among patients living with HIV [109]. However, the finding of this review was lower than studies reported 84% in Ethiopia [110], 85% in Sub-Saharan Africa [111], and far lower than

~~a~~ the global target of achieving 95% viral suppression in ~~at least 95% of~~ patients ~~initiating~~ receiving ART [33]. In contrast, the findings of the current study were higher than a study conducted in Ethiopia (71%) [112].

This could be due to the difference in the study population, study setting, and availability and adherence to ART. Thus, the study population for the previous studies was adults living with HIV, whereas the current study involves pregnant and lactating women receiving ART in Africa. The possible explanation could relate to physiological and immunological changes during perinatal periods [113]. Pregnancy alters the immune system and pharmacokinetics of ART drugs, which reduce the effectiveness of ART and increase maternal viral load [113, 114].

Furthermore, delayed initiation or inadequate adherence to ART, perceived shame, and socioeconomic or health system constraints, such as limited regular VL surveillance, ART stock-outs, and a shortage of maternity professionals could be the possible reason for non-virological suppression [18, 62, 115]. Suboptimal viral suppression or virologic failure jeopardizes not only the health of women but also raises the risk of HIV MTCT during pregnancy, childbirth, and breastfeeding, posing a challenge to the global effort towards eliminating pediatric HIV infection [62, 116, 117].

The UNAIDS's 95-95-95 goals for all group of populations, including pregnant and lactating women demonstrate the globe's ongoing dedication to combating the HIV/AIDS and reaching the 2030 sustainable development goal of "ending AIDS" as a global health concern [34].

Despite considerable global progress towards reaching the 95-95-95 goals in the past decade, many low-middle-income countries, including many African countries are still far from this goal [118].

Reaching UNAIDS's third 95% target; ensuring that 95% of people living with HIV on ART maintain sustained VLS; is vital for preventing HIV transmission, improving health outcomes, and

optimizing the quality of life [48]. Nonetheless, ART availability and adherence, ongoing structural and socioeconomic inequities, health-care system fragilities, and financing constraints, considerably impede towards achieving VS in these key population groups [47, 48, 119]. Despite the hurdles targeted interventions, including better ART adherence support, integrated maternal-HIV care, robust VL monitoring, addressing structural, financial and sociocultural barriers to close the gap and achieve the global target, ultimately lowering vertical transmission and improving maternal and child health outcomes [48, 119].

According to the findings of this study, Africa has not yet achieved the global target of attaining virological suppression in 95% of patients who received ART. To increase the possibility of successful virological outcomes, a variety of factors and problems must be addressed. In this systematic review, individual-level variables, healthcare utilization (time of ANC booking, time of ART initiation, being on a first-line ART regimen, duration of ART, baseline VL threshold, adherence status to ART), and barrier of healthcare services including, distance to health facility, shortage of health professionals, disclosure of HIV status, inadequate counseling, ART drug stock-out, sub-optimal sample transportation, were significantly associated with virologic suppression among HIV-positive pregnant and lactating women in Africa.

~~women's age category of 15-24 years, being unmarried, being on a first line ART regimen, disclosure of HIV status to a partner, and good adherence to ART were significantly associated with virological suppression in Africa.~~

In this study, the odds of virologic suppression among young women, particularly those 15-24 years old, were decreased by 51% when compared with those aged 25 years and above. The finding of this study was consistent with other studies [115, 120-122]. Younger people receiving treatment for HIV might have difficulty with compliance due to various factors, including ~~lifestyle demands~~

~~such as education or a job~~employment status, societal expectations, worries regarding stigma and disclosure, mental health challenges, and lacking health literacy [115, 123]. In Africa, the stigma that exists at the individual, interpersonal, social, and organizational levels remain to pose significant hurdles for receiving and adhering ART for adolescents living with HIV [124, 125]. Antiretroviral therapy (ART) has changed the treatment of HIV from a deadly illness to a chronic condition that can be managed owing to its increased accessibility, and continuing adherence [126]. Nevertheless, there are several obstacles in adolescents HIV care cohort, which frequently put this age group at risk for less favorable outcomes than adults in the HIV care pathway. These issues include poorer adherence, lower retention in therapy, a lower rate of virological suppression, and greater rates of mortality [123, 127-129].

Collaborated global, regional and national efforts are needed to reach the UNAIDS 95% sustained viral suppression rate, especially for adolescents living with HIV, who are frequently linked with elevated rates of virological failure (VF) [34, 119]. Understanding and addressing the unique challenges faced by young women helps healthcare workers and decision-makers create specific treatments that boost viral suppression rates and enhance health for this ~~at risk~~vulnerable age group. Therefore, promoting ART adherence support, regular VL monitoring, accessible and flexible adolescent-youth-friendly HIV care, while guaranteeing accessibility, privacy, and a favorable environment plays pivotal role in achieving VS in these groups of population [124, 125]. ~~Enhance compliance support, introduce youth-friendly programs, and cater to the special requirements of youths and young adults, while guaranteeing accessibility, privacy, and a favorable environment.~~

The odds of achieving virologic suppression were higher among women living in urban areas than women residing in rural areas. This finding was consistent with previous study findings [67, 100,

102]. This could due to women live in urban areas are more likely to have better awareness and access to HIV care, including periodic VL monitoring, continuous ART supply, as well as enhanced counseling on ART adherence. Whereas rural women frequently encounter geographical, infrastructural, and service-related barriers that impede optimal ART adherence and VS [100]. Furthermore, women's educational status was significantly associated with virologic suppression, as studies consistently show that women with secondary or higher education are more likely to achieve VS than those with no formal education [67, 76]. This could be explained as education increases awareness of navigating medical care, importance of ART adherence, and access to HIV care; in contrast, lower educated women could find it challenging to grasp treatment, receive regular medical care, and fear of stigma.

Moreover, pregnant and breastfeeding women who disclosed their HIV status were significantly associated with achieving virological suppression compared to those who did not. This finding is in agreement with other studies that revealed disclosure of HIV status increases the likelihood of ~~viral load~~ virologic suppression [38, 110, 130]~~.~~[18, 38, 41, 100, 106-108, 110, 115, 131]. ~~The~~ D~~d~~isclosing ~~ure of~~ one's HIV status to family, friends, and intimate partner ~~relationships~~ can have ~~major~~ positive health -impacts ~~consequences~~. Several studies show that individuals who disclosed their serostatus had better social assistance, stronger family and relationship solidarity, lower symptoms of depression and anxiety, improved physical health, psychological assistance, ~~and~~ financial support, and ~~were~~ better ~~able to take and~~ adherence to ART [132-134]. However, fear of stigma and discrimination was found significantly associated with virologic suppression among HIV-positive pregnant and lactating women receiving ART [13, 104, 105, 115]. Stigma can take many forms, including societal discrimination, internalized prejudice, family stigma, and stigma

in healthcare environments. This may result to fear of disclosing status or withholding drugs, lack of self-worth, and failure to ART adhere [46, 135, 136].

Women who began ANC contacts or started ART in the second or third trimester were less likely to achieve virologic suppression [45, 67, 100, 103]. Late beginning of ANC follow-up and ART decreases the time period of effective ART for decreasing VL before childbirth and limiting chances for regular monitoring of ART treatment outcomes, enhanced counseling for adherence, and prompt response to treatment challenges, which are essential for attaining and sustaining optimum virologic suppression to prevent MTCT of HIV.

Advanced WHO HIV stages (II–IV), marked by opportunistic infections, were significantly associated with failure to achieve virologic suppression among pregnant and lactating women on ART [41, 103, 115]. Advanced HIV stages elevate the likelihood of virologic suppression failure because growing immune suppression and opportunistic infections may hamper ART efficacy, alter adherence, and increase the risk of treatment failure, thereby rendering VL control more challenging for pregnant and breastfeeding women [41, 115].

This study also found that patients on first-line ART regimens had higher rates of virologic suppression compared to those on second- or third-line regimens, consistent with findings from other studies [38, 39, 137, 138]. This could be explained as first-line ART drugs (TDF-3TC-DTG) having higher efficacy (sustained VL suppression), fewer adverse effects, and lower resistance when compared to second- and third-line ART regimens [26, 139, 140]. Therefore, all HIV-positive pregnant and lactating women should initiate triple ART as early as possible, regardless of their CD4 count or WHO clinical stage. This leads to optimal suppression of viral load to undetectable levels, thereby preventing the vertical transmission of HIV [141]. Nevertheless, the virus can never be totally eliminated from the human body, so an individual should continue to

take the treatments even after the signs and symptoms have subsided and adhere to safer sexual practices [26, 139, 142].

~~Finally~~Furthermore, good adherence to antiretroviral therapy was significantly associated with virologic suppression among pregnant and breastfeeding women, aligning with findings from previous studies [38, 39, 41, 137]. The plausible explanation is due to the fact that good adherence to ART sustains maximal viral load suppression to undetectable levels, lowers drug resistance, and boosts the immune system (CD4 cell counts), which leads to substantially lowering the risk of mother-to-child transmission of HIV and improving the overall health of individuals [139, 143]. However, ~~compliance~~ adherence ~~with~~ to ART remains a major health issue worldwide, particularly in low- and middle-income nations. Virologic and clinical outcomes are highly dependent on strong compliance to ART, while poor adherence to antiretroviral drugs has been found to be a significant predictor of virologic failure, drug resistance, progression of disease, admissions to hospitals, high death rate, and medical care expenses [115, 144, 145].

On the other hand, long distance to reach health facility, shortage of health provider, inadequate counseling, ART drugs running out, and shortage of HIV commodities linked with failure of virologic suppression [13, 46, 100, 104, 105]. [46, 146]. Health facility availability is a crucial infrastructural variable determining adherence to ART, especially in low-income countries, in which medical infrastructure is frequently poor. Individuals with work or domestic responsibilities have significant challenges due to the facility's limited operation hours or inconvenient hours, which are often only available during weekdays daytime [46].

Similarly, ART stockouts in healthcare facilities result in viral rebounding, missed ART doses, drug resistance, and switching to the second-line therapy [46, 147-149]. ART drugs can occasionally be inaccessible due to supply-chain issues or financing shortages in several

developing countries. Patients may receive an insufficient supply or nothing at all as they come for refilling following a stockout, which would cause them to skip medications [46, 149, 150]. In general, the findings of this review have various implications for clinical practice, policy, and future research works in these population groups. Since lifelong first-line ART regimens and enhanced adherence to ART have been strongly linked to greater virologic suppression, healthcare practitioners should increase adherence counseling, particularly during prenatal and postpartum contacts. The lower suppression rates in younger women demonstrate the necessity for youth-specific strategies like youth-friendly clinics, psychosocial assistance, and sexual and reproductive health education incorporated into HIV treatment programs. Optimizing virologic suppression in pregnant and breastfeeding women on ART in Africa requires a comprehensive strategy that includes upgrading clinical practices, ensuring responsive policy, and ongoing VL monitoring and evaluation to address ongoing barriers, gaps and disparities to achieve VS. Further qualitative and quantitative studies is required to investigate different sociocultural, behavioral, psychological, and structural factors that lead to virologic non-suppression among HIV-positive pregnant and breastfeeding women in Africa~~the continent~~.

Strengths and limitations of the study

To our knowledge, this would be the first meta-analysis to assess the overall estimate of virologic suppression and associated factors among HIV-positive pregnant and nursing women in Africa. We searched multiple databases to include both published and unpublished studies and followed a meticulous screening approach to compile all eligible studies. Thus, the findings of this review provide important baseline evidence for health providers, policymakers, academicians, and global partners to design and implement evidence-based interventions to achieve the WHO 95-95-95 global target to eliminate vertical transmission of HIV.

Despite the fact that enough primary studies were used to estimate the overall viral load suppression, many countries and some African regions were not evenly represented. The presence of significant heterogeneity among the included studies might affect the generalizability of the finding. This might be due to variations in the study area and the methodological quality of the studies, such as study design, sampling technique, response rate, sample size, and study setting, that could alter the pooled estimate's generalizability. Additionally, due to the concentration of studies in some countries, such as South Africa, it could restrict the conclusion of the finding to the whole continent. We employed a random-effects model for analysis in order to overcome this significant variability. Additionally, we performed subgroup analyses and sensitivity analyses to identify potential causes of substantial heterogeneity.

Conclusions

According to this systematic review and meta-analysis, the overall prevalence of virologic suppression among pregnant and lactating women taking ART in Africa (80.92%) is lower than USAID's global target of viral load suppression (95%).

In this study, socio-demographic characteristics (women’s age, higher educational level, being married/cohabitant, urban residency) and healthcare utilization (time of ANC booking, time of ART initiation, being on a first-line ART regimen, duration of ART, and adherence status to ART) were significantly associated with virologic suppression among HIV-positive pregnant and lactating women in Africa. Additionally, virologic suppression also substantially linked with various facilitators and barriers, including disclosing of HIV status, fear of stigma, distance to health facility, shortage of health professionals, ART drug stock-out, and lack of HIV care commodities. Being younger (15-24 years), being on a first-line ART regimen, disclosing HIV status to a partner, and having good adherence to ART were significantly associated with virologic

suppression. This highlights the importance of targeted interventions for young HIV-positive women, encouraging disclosing HIV status, initiating first-line antiretroviral therapy regimens, and promoting antiretroviral therapy adherence; strengthening healthcare systems that offer regular viral load monitoring; and addressing socio-demographic and ART-related factors as vital steps toward improving treatment outcomes and reducing perinatal transmission of HIV.

List of abbreviations

ART: Antiretroviral therapy, AOR: Adjusted odds ratio, CI: Confidence Interval, CD4: Cluster of differentiation 4, HIV: Human Immunodeficiency Virus, MTCT: Mother-to-Child Transmission of HIV, SRM: Systematic review and meta-analysis, VL: Viral load, VS: Virologic Suppression, UNAIDS: Joint United Nations Programme on HIV/AIDS, WHO: World health organization,

Declarations

Ethical approval

Not applicable

Consent for publication

Not applicable

Availability of data and materials

All relevant data are available within the manuscript or supplementary materials.

Competing interests

The authors have declared that no competing interests exist.

Funding

~~None funding~~

~~Author contributions~~

~~Conceptualization: BAM, NTT, GGM~~

~~Data curation: NTT, BAM, TAA, KYW, AEA, HKN~~

~~Formal analysis: NTT, BAM, KYW, HKN, ATK, WMB, GMA~~

~~Methodology: BAM, NTT, GMA, AEA, GGM, EG, MTT~~

~~Software: BAM, ATK, WMB, GMA, NTT~~

~~Validation: NTT, BAM, KYW, GMA, AEA, EG~~

~~Visualization: GMA, NTT, AEA, GGM~~

~~Writing original draft: TAA, KYW, ATK, MTT, WMB, HKN, GMA, AEA, NTT~~

~~Writing – review & editing: NTT, BAM, TAA, EG, KYW, ATK, GGM, WMB, GMA~~

Acknowledgements

The authors of this study would like to express our heartfelt gratitude to all of the authors of the studies included in this systematic review and meta-analysis.

References

1. (WHO). WHO. HIV statistics, globally and by WHO region, 2025. Accessed on: https://cdn.who.int/media/docs/default-source/hq-hiv-hepatitis-and-stis-library/who-ias-hiv-statistics_2025-new.pdf. 2025 [
2. (UNAIDS). UNPoHA. Global HIV & AIDS statistics — Fact sheet. Access: https://www.unaids.org/en/resources/fact-sheet. 2025 [
3. UNAIDS. Fact sheet 2024 - Latest global and regional HIV statistics on the status of the AIDS epidemic. 2024.
4. (WHO) WHO. HIV and AIDS 2024 [
5. Tilaye BA, Hailemeskel HS, Baye FD, Hailu MK, Mekonnen GB, Arage G, et al. Incidence of mother-to-child transmission of HIV and predictors of positivity among HIV exposed infants in South Gondar public hospitals, Northwest Ethiopia: competing risk regression model. BMC Pediatr. 2024;24(1):597.

6. Chi BH, Mbori-Ngacha D, Essajee S, Mofenson LM, Tsiouris F, Mahy M, et al. Accelerating progress towards the elimination of mother-to-child transmission of HIV: a narrative review. J Int AIDS Soc. 2020;23(8):e25571.
7. WHO. Mother-to-child transmission of HIV. https://www.who.int/teams/global-hiv-hepatitis-and-stis-programmes/hiv/prevention/mother-to-child-transmission-of-hiv#:~:text=Globally%2C%20an%20estimated%201.3%20million,been%20shown%20to%20be%20feasible. 2024 [
8. Omonaiye O, Kusljic S, Nicholson P, Manias E. Medication adherence in pregnant women with human immunodeficiency virus receiving antiretroviral therapy in sub-Saharan Africa: a systematic review. BMC public health. 2018;18:1-20.
9. Organization WH. Progress report 2016: prevent HIV, test and treat all: WHO support for country impact. Progress report 2016: prevent HIV, test and treat all: WHO support for country impact2016.
10. Seale A, Baggaley R, Vojnov L, Doherty M. Role of viral suppression in HIV treatment and prevention and its potential in addressing harmful laws and discrimination. Sex Health. 2023;20(3):181-5.
11. Organization WH. The role of HIV viral suppression in improving individual health and reducing transmission: policy brief: World Health Organization; 2023.
12. Chilaka VN, Konje JC. HIV in pregnancy–An update. European Journal of Obstetrics & Gynecology and Reproductive Biology. 2021;256:484-91.
13. Musanhu CCC, Takarinda KC, Shea J, Chitsike I, Eley B. Viral load testing among pregnant women living with HIV in Mutare district of Manicaland province, Zimbabwe. AIDS Research and Therapy. 2022;19(1):52.
14. Organization WH. Guidelines: updated recommendations on HIV prevention, infant diagnosis, antiretroviral initiation and monitoring. Guidelines: updated recommendations on HIV prevention, infant diagnosis, antiretroviral initiation and monitoring2021.
15. Organization WH. Updated Recommendations on HIV Prevention, Infant Diagnosis, Antiretroviral Initiation and Monitoring: World Health Organization; 2022.
16. WHO Guidelines Approved by the Guidelines Review Committee. Consolidated Guidelines on the Use of Antiretroviral Drugs for Treating and Preventing HIV Infection: Recommendations for a Public Health Approach. Geneva: World Health Organization

Copyright © World Health Organization 2016.; 2016.
17. Gilleece DY, Tariq DS, Bamford DA, Bhagani DS, Byrne DL, Clarke DE, et al. British HIV Association guidelines for the management of HIV in pregnancy and postpartum 2018. HIV Med. 2019;20 Suppl 3:s2-s85.
18. Schrubbe LA, Stöckl H, Hatcher AM, Marston M, Kuchukhidze S, Calvert C. Prevalence and risk factors of unsuppressed viral load among pregnant and breastfeeding women in sub-Saharan Africa: analysis from population-based surveys. Aids. 2023;37(4):659-69.
19. Hailu G, Keraleme A, Zealiyas K, Tesema A, Nuramed N, Girmachew F, et al. Human Immunodeficiency Virus (HIV) viral load suppression status and associated factors among pregnant women receiving Highly Active Antiretroviral Therapy (HAART) in Ethiopia. Virology Journal. 2025;22(1):49.
20. Munyaneza A, Kim H-Y, Shi Q, Brazier E, Ross J, Muhoza B, et al. Factors associated with viral load suppression in pregnant and postpartum women living with HIV

in Rwanda: an open-observational cohort study. Frontiers in Public Health. 2025;13:1544165.
21. Elijah O, Umukoro E, Igben V, Moke E, Ojezele M, Onitiri-Elijah C. Prevention of Mother-To-Child Transmission of HIV (PMTCT) in Sub-Saharan Africa: Past and Present Perspectives. International Journal of Forensic Medical Investigation. 2025;11(1).
22. Mukose AD. Implementation of lifelong ART (Option B+) for Prevention of Mother-to-Child Transmission (PMTCT) of HIV in Uganda: University of Antwerp; 2024.
23. Organization WH. Introducing a framework for implementing triple elimination of mother-to-child transmission of HIV, syphilis and hepatitis B virus: policy brief: World Health Organization; 2024.
24. Adelekan B, Harry-Erin B, Okposo M, Aliyu A, Ndembi N, Dakum P, et al. Final HIV status outcome for HIV-exposed infants at 18 months of age in nine states and the Federal Capital Territory, Nigeria. PLoS One. 2022;17(2):e0263921.
25. Rasmussen DN, Vieira N, Hønge BL, da Silva Té D, Jespersen S, Bjerregaard-Andersen M, et al. HIV-1 and HIV-2 prevalence, risk factors and birth outcomes among pregnant women in Bissau, Guinea-Bissau: a retrospective cross-sectional hospital study. Sci Rep. 2020;10(1):12174.
26. Organization WH. Updated recommendations on first-line and second-line antiretroviral regimens and post-exposure prophylaxis and recommendations on early infant diagnosis of HIV. Updated recommendations on first-line and second-line antiretroviral regimens and post-exposure prophylaxis and recommendations on early infant diagnosis of HIV2018. p. [1-82].
27. Hoffman RM, Black V, Technau K, van der Merwe KJ, Currier J, Coovadia A, et al. Effects of highly active antiretroviral therapy duration and regimen on risk for mother-to-child transmission of HIV in Johannesburg, South Africa. J Acquir Immune Defic Syndr. 2010;54(1):35-41.
28. Fox MP, Pascoe S, Huber AN, Murphy J, Phokojoe M, Gorgens M, et al. Adherence clubs and decentralized medication delivery to support patient retention and sustained viral suppression in care: Results from a cluster-randomized evaluation of differentiated ART delivery models in South Africa. PLoS Med. 2019;16(7):e1002874.
29. Chagomerana MB, Miller WC, Tang JH, Hoffman IF, Mthiko BC, Phulusa J, et al. Optimizing prevention of HIV mother to child transmission: Duration of antiretroviral therapy and viral suppression at delivery among pregnant Malawian women. PLoS One. 2018;13(4):e0195033.
30. Lazenby GB, Powell AM, Sullivan SA, Soper DE. The Impact of Delivery in a Rural County on a Cohort of Women Living with HIV Infection and Their Infants. J Rural Health. 2019;35(3):319-29.
31. Bulage L, Ssewanyana I, Nankabirwa V, Nsubuga F, Kihembo C, Pande G, et al. Factors associated with virological non-suppression among HIV-positive patients on antiretroviral therapy in Uganda, August 2014–July 2015. BMC infectious diseases. 2017;17:1-11.
32. Awaidy SA, Ghazy RM, Mahomed O. Progress of the gulf cooperation council (gcc) countries towards achieving the 95-95-95 UNAIDS targets: a review. Journal of epidemiology and global health. 2023;13(3):397-406.
33. Mwende Wairimu G. The Global Burden of HIV and Progress toward the 2030 UNAIDS Targets: A Comprehensive Review.

34. Frescura L, Godfrey-Faussett P, Feizzadeh A, El-Sadr W, Syarif O, Ghys PD, et al. Achieving the 95 95 95 targets for all: A pathway to ending AIDS. Plos one. 2022;17(8):e0272405.

35. Mosha IH, Nyondo GG, Munishi CG, Njiro BJ, Bwire GM. Prevalence and factors associated with viral non-suppression in people living with HIV receiving antiretroviral therapy in sub-Saharan Africa: A systematic review and meta-analysis. Rev Med Virol. 2024;34(3):e2540.

36. Agegnehu CD, Techane MA, Mersha AT, Atalell KA. Burden and Associated Factors of Virological Failure Among People Living with HIV in Sub-Saharan Africa: A Systematic Review and Meta-Analysis. AIDS Behav. 2022;26(10):3327-36.

37. Namaganda MM, Mukasa Kafeero H, Nakatumba Nabende J, Kateete DP, Batte C, Wanyengera M, et al. Prevalence and predictors of virological failure among the people living with HIV on antiretroviral treatment in East Africa: evidence from a systematic review with meta-analysis and meta-regression of published studies from 2016 to 2023. HIV Research & Clinical Practice. 2025;26(1):2490774.

38. Melak D, Bayou FD, Yasin H, Zerga AA, Wagaye B, Ayele FY, et al., editors. Virological suppression and its predictors among HIV/AIDS patients on antiretroviral therapy in Ethiopia: Systematic review and meta-analysis. Open forum infectious diseases; 2024: Oxford University Press US.

39. Temere BC, Mewahegn AA, Zewudie BT, GebreEyesus FA, Abebe H, Nuru A, et al. Burden of virologic failure, and its determinants among HIV positive children in East Africa: a systematic review and meta-analysis. African Health Sciences. 2024;24(3):1-9.

40. Abuogi LL, Humphrey JM, Mpody C, Yotebieng M, Murnane PM, Clouse K, et al. Achieving UNAIDS 90-90-90 targets for pregnant and postpartum women in sub-Saharan Africa: progress, gaps and research needs. Journal of Virus Eradication. 2018;4:33-9.

41. Woldesemayat B, Yizengaw A, Worku A, Yilma A, Adane S, Bulti J, et al. HIV Viral Non-Suppression and Its Associated Factors Among PMTCT Mothers Receiving ARV Treatment in Ethiopia. medRxiv. 2024:2024.06. 13.24308915.

42. Gabagaya G, Rukundo G, Amone A, Wavamunno P, Namale-Matovu J, Lubega I, et al. Prevalence of undetectable and suppressed viral load in HIV-infected pregnant women initiating Option B+ in Uganda: an observational study nested within a randomized controlled trial. BMC infectious diseases. 2021;21:1-7.

43. Agegnehu CD, Techane MA, Mersha AT, Atalell KA. Burden and associated factors of virological failure among people living with HIV in sub-Saharan Africa: a systematic review and meta-analysis. AIDS and Behavior. 2022;26(10):3327-36.

44. Bakari HM, Alo O, Mbwana MS, Salim SM, Ally ZM, Musoke R, et al. Enhanced adherence counselling completion rates, viral load suppression in people with HIV in Africa: systematic review and meta-analysis. Discover Public Health. 2025;22(1):12.

45. Woldesenbet SA, Kufa T, Barron P, Chirombo BC, Cheyip M, Ayalew K, et al. Viral suppression and factors associated with failure to achieve viral suppression among pregnant women in South Africa. AIDS. 2020;34(4):589-97.

46. Magura J, Nhari SR, Nzimakwe TI. Barriers to ART adherence in sub-Saharan Africa: a scoping review toward achieving UNAIDS 95-95-95 targets. Frontiers in Public Health. 2025;13:1609743.

47. Kulohoma B, Wesonga CA. HIV response financing challenges in Sub-Saharan Africa: barriers to achieving the 95-95-95 UNAIDS targets. Frontiers in Public Health. 2025;13:1658229.
48. Mukuku O, Govender K. At a crossroads: confronting setbacks and advancing the 95-95-95 HIV targets in sub-Saharan Africa. AIDS Research and Therapy. 2025;22(1):92.
49. Omonaiye O, Kusljic S, Nicholson P, Manias E. Medication adherence in pregnant women with human immunodeficiency virus receiving antiretroviral therapy in sub-Saharan Africa: a systematic review. BMC public health. 2018;18(1):805.
50. Kadia BM, Dimala CA, Fongwen NT, Smith AD. Barriers to and enablers of uptake of antiretroviral therapy in integrated HIV and tuberculosis treatment programmes in sub-Saharan Africa: a systematic review and meta-analysis. AIDS research and therapy. 2021;18(1):85.
51. Munkhondya TEM, Smyth RM, Lavender T. Facilitators and barriers to retention in care under universal antiretroviral therapy (Option B+) for the Prevention of Mother to Child Transmission of HIV (PMTCT): A narrative review. International Journal of Africa Nursing Sciences. 2021;15:100372.
52. Page MJ, McKenzie JE, Bossuyt PM, Boutron I, Hoffmann TC, Mulrow CD, et al. The PRISMA 2020 statement: an updated guideline for reporting systematic reviews. BMJ (Clinical research ed). 2021;372:n71.
53. Organization WH. Consolidated guidelines on HIV prevention, testing, treatment, service delivery and monitoring: recommendations for a public health approach: World Health Organization; 2021.
54. Moyo F, Mazanderani AH, Murray T, Sherman GG, Kufa T. Achieving maternal viral load suppression for elimination of mother-to-child transmission of HIV in South Africa. Aids. 2021;35(2):307-16.
55. Munn Z, Moola S, Lisy K, Riitano D, Tufanaru C. Methodological guidance for systematic reviews of observational epidemiological studies reporting prevalence and cumulative incidence data. JBI Evidence Implementation. 2015;13(3):147-53.
56. Barker TH HS, Aromataris E, Stone JC, Leonardi-Bee J, Sears K, Habibi N, Klugar M, Tufanaru C, Moola S, Liu XL. The revised JBI critical appraisal tool for the assessment of risk of bias for cohort studies. . JBI Evidence Synthesis 2025.
57. Huedo-Medina TB, Sánchez-Meca J, Marín-Martínez F, Botella J. Assessing heterogeneity in meta-analysis: Q statistic or I² index? Psychological methods. 2006;11(2):193.
58. Langan D, Higgins JPT, Jackson D, Bowden J, Veroniki AA, Kontopantelis E, et al. A comparison of heterogeneity variance estimators in simulated random-effects meta-analyses. Res Synth Methods. 2019;10(1):83-98.
59. Boedeker P, Henson RK. Evaluation of heterogeneity and heterogeneity interval estimators in random-effects meta-analysis of the standardized mean difference in education and psychology. Psychol Methods. 2020;25(3):346-64.
60. Begg CB, Mazumdar M. Operating characteristics of a rank correlation test for publication bias. Biometrics. 1994:1088-101.
61. Ntombela NP, Kharsany ABM, Soogun A, Yende-Zuma N, Baxter C, Kohler HP, et al. Viral suppression among pregnant adolescents and women living with HIV in rural KwaZulu-Natal, South Africa: a cross sectional study to assess progress towards UNAIDS indicators and Implications for HIV Epidemic Control. Reprod Health. 2022;19(1):116.

62. Ngandu NK, Lombard CJ, Mbira TE, Puren A, Waitt C, Prendergast AJ, et al. HIV viral load non-suppression and associated factors among pregnant and postpartum women in rural northeastern South Africa: a cross-sectional survey. BMJ open. 2022;12(3):e058347.
63. Adeniyi OV, Obi CL, Goon DT, Iweriebor B, Selanto-Chairman N, Carty C, et al. Factors Associated With Peripartum Virologic Suppression in Eastern Cape Province, South Africa: A Retrospective Cross-Sectional Analysis. Clin Infect Dis. 2021;73(10):1750-8.
64. Chetty T, Newell ML, Thorne C, Coutsoudis A. Viraemia before, during and after pregnancy in HIV-infected women on antiretroviral therapy in rural KwaZulu-Natal, South Africa, 2010-2015. Trop Med Int Health. 2018;23(1):79-91.
65. Phillips TK, Clouse K, Zerbe A, Orrell C, Abrams EJ, Myer L. Linkage to care, mobility and retention of HIV-positive postpartum women in antiretroviral therapy services in South Africa. J Int AIDS Soc. 2018;21 Suppl 4(Suppl Suppl 4):e25114.
66. Woldesenbet S, Cheyip M, Lombard C, Manda S, Ayalew K, Kufa T, et al. Progress towards the UNAIDS 95-95-95 targets among pregnant women in South Africa: Results from the 2017 and 2019 national Antenatal HIV Sentinel Surveys. PLoS One. 2022;17(7):e0271564.
67. Woldesenbet S, Kufa T, Manda S, Ayalew K, Lombard C, Cheyip M, et al. Association between viral suppression during the third trimester of pregnancy and unintended pregnancy among women on antiretroviral therapy: Results from the 2019 antenatal HIV Sentinel Survey, South Africa. Plos one. 2022;17(3):e0265124.
68. Myer L, Dunning L, Lesosky M, Hsiao NY, Phillips T, Petro G, et al. Frequency of Viremic Episodes in HIV-Infected Women Initiating Antiretroviral Therapy During Pregnancy: A Cohort Study. Clin Infect Dis. 2017;64(4):422-7.
69. Myer L, Phillips TK, Hsiao NY, Zerbe A, Petro G, Bekker LG, et al. Plasma viraemia in HIV-positive pregnant women entering antenatal care in South Africa. J Int AIDS Soc. 2015;18(1):20045.
70. Brittain K, Remien RH, Mellins CA, Phillips TK, Zerbe A, Abrams EJ, et al. Determinants of suboptimal adherence and elevated HIV viral load in pregnant women already on antiretroviral therapy when entering antenatal care in Cape Town, South Africa. AIDS Care. 2018;30(12):1517-23.
71. Moyo F, Haeri Mazanderani A, Murray T, Technau KG, Carmona S, Kufa T, et al. Characterizing Viral Load Burden Among HIV-Infected Women Around the Time of Delivery: Findings From Four Tertiary Obstetric Units in Gauteng, South Africa. J Acquir Immune Defic Syndr. 2020;83(4):390-6.
72. Myer L, Phillips TK, McIntyre JA, Hsiao NY, Petro G, Zerbe A, et al. HIV viraemia and mother-to-child transmission risk after antiretroviral therapy initiation in pregnancy in Cape Town, South Africa. HIV Med. 2017;18(2):80-8.
73. Langwenya N, Phillips TK, Brittain K, Zerbe A, Abrams EJ, Myer L. Same-day antiretroviral therapy (ART) initiation in pregnancy is not associated with viral suppression or engagement in care: A cohort study. J Int AIDS Soc. 2018;21(6):e25133.
74. Anderson K, Kalk E, Heekes A, Phelanyane F, Jacob N, Boulle A, et al. Factors associated with vertical transmission of HIV in the Western Cape, South Africa: a retrospective cohort analysis. J Int AIDS Soc. 2024;27(3):e26235.

75. Tsondai PR. HIV viral load monitoring in HIV-infected pregnant women established on antiretroviral therapy in Cape Town, South Africa: University of Cape Town; 2016.
76. Landes M, van Lettow M, van Oosterhout JJ, Schouten E, Auld A, Kalua T, et al. Early post-partum viremia predicts long-term non-suppression of viral load in HIV-positive women on ART in Malawi: Implications for the elimination of infant transmission. PLoS One. 2021;16(3):e0248559.
77. Chagomerana MB, Miller WC, Tang JH, Hoffman IF, Harrington BJ, DiPrete B, et al. Prevalence of antiretroviral therapy treatment failure among HIV-infected pregnant women at first antenatal care: PMTCT Option B+ in Malawi. PLoS One. 2018;13(12):e0209052.
78. Landes M, van Lettow M, Nkhoma E, Tippett Barr B, Truwah Z, Shouten E, et al. Low detectable postpartum viral load is associated with HIV transmission in Malawi's prevention of mother-to-child transmission programme. J Int AIDS Soc. 2019;22(6):e25290.
79. Napyo A, Tumwine JK, Mukunya D, Tumuhamye J, Arach AAO, Ndeezi G, et al. Detectable HIV-RNA Viral Load Among HIV-Infected Pregnant Women on Treatment in Northern Uganda. Int J MCH AIDS. 2020;9(2):232-41.
80. Munyaneza A, Kim HY, Shi Q, Brazier E, Ross J, Muhoza B, et al. Factors associated with viral load suppression in pregnant and postpartum women living with HIV in Rwanda: an open-observational cohort study. Front Public Health. 2025;13:1544165.
81. Gill MM, Hoffman HJ, Bobrow EA, Mugwaneza P, Ndatimana D, Ndayisaba GF, et al. Detectable Viral Load in Late Pregnancy among Women in the Rwanda Option B+ PMTCT Program: Enrollment Results from the Kabeho Study. PLoS One. 2016;11(12):e0168671.
82. Koss CA, Natureeba P, Kwarisiima D, Ogena M, Clark TD, Olwoch P, et al. Viral Suppression and Retention in Care up to 5 Years After Initiation of Lifelong ART During Pregnancy (Option B+) in Rural Uganda. J Acquir Immune Defic Syndr. 2017;74(3):279-84.
83. Gabagaya G, Rukundo G, Amone A, Wavamunno P, Namale-Matovu J, Lubega I, et al. Prevalence of undetectable and suppressed viral load in HIV-infected pregnant women initiating Option B+ in Uganda: an observational study nested within a randomized controlled trial. BMC Infect Dis. 2021;21(1):907.
84. Humphrey JM, Songok J, Ofner S, Musick B, Alera M, Kipchumba B, et al. Retention in care and viral suppression in the PMTCT continuum at a large referral facility in western Kenya. AIDS Behav. 2022;26(11):3494-505.
85. Sandbulte M, Brown M, Wexler C, Maloba M, Gautney B, Goggin K, et al. Maternal viral load monitoring: Coverage and clinical action at 4 Kenyan hospitals. PLoS One. 2020;15(5):e0232358.
86. Boisson-Walsh A, Ravelomanana NLR, Tabala M, Malongo F, Kawende B, Babakazo P, et al. Association of comprehensiveness of antiretroviral care and detectable HIV viral load suppression among pregnant and postpartum women in the Democratic Republic of the Congo: a cross-sectional study. Front Glob Womens Health. 2024;5:1308019.
87. Yotebieng M, Mpody C, Ravelomanana NL, Tabala M, Malongo F, Kawende B, et al. HIV viral suppression among pregnant and breastfeeding women in routine care in the

Kinshasa province: a baseline evaluation of participants in CQI-PMTCT study. J Int AIDS Soc. 2019;22(9):e25376.
88. Woldesemayat B, Yizengaw A, Worku A, Yilma A, Adane S, Bulti J, et al. HIV Viral Non-Suppression and Its Associated Factors Among PMTCT Mothers Receiving ARV Treatment in Ethiopia. medRxiv. 2024:2024.06.13.24308915.
89. Hailu G, Keraleme A, Zealiyas K, Tesema A, Nuramed N, Girmachew F, et al. Human Immunodeficiency Virus (HIV) viral load suppression status and associated factors among pregnant women receiving Highly Active Antiretroviral Therapy (HAART) in Ethiopia. Virol J. 2025;22(1):49.
90. Demissie DB, Bulto GA, Mekuria WT, Dufera FN, Gamshe EN. Evaluation of Antiretroviral Therapy Initiated Among Pregnant Women Under Option B+ by Viral Load and CD4 Count Outcomes in Selected Hospitals of West Shewa Zone, Oromia Region, Ethiopia. HIV AIDS (Auckl). 2020;12:127-34.
91. Endalamaw Alamneh D, Shiferaw MB, Getachew Demissie M, Emiru MA, Zemene Kassie T, Endaylalu Lakew K, et al. Virological Outcomes Among Pregnant Women Receiving Antiretroviral Treatment in the Amhara Region, North West Ethiopia. HIV AIDS (Auckl). 2023;15:209-16.
92. Aduloju O, Aduloju T, Ade-Ojo I, Akintayo A. Medication adherence in HIV-positive pregnant women on antiretroviral therapy attending antenatal clinics in Ado metropolis, south-west Nigeria: A multicentre study. South African Journal of Obstetrics and Gynaecology. 2020;26:65.
93. Musanhu CCC, Takarinda KC, Shea J, Chitsike I, Eley B. Viral load testing among pregnant women living with HIV in Mutare district of Manicaland province, Zimbabwe. AIDS Res Ther. 2022;19(1):52.
94. Duri K, Munjoma P, Mazhandu A, Marere T, Gomo E, Banhwa S, et al. Predictors and Timing to Viral Suppression in HIV-Infected Pregnant Women in the University of Zimbabwe Birth Cohort Study During the Era of Lifelong Antiretroviral Therapy (Option B+ Treatment Strategy). Frontiers in Virology. 2022;2.
95. Atanga PN, Ndetan HT, Fon PN, Meriki HD, Muffih TP, Achidi EA, et al. Using a composite adherence tool to assess ART response and risk factors of poor adherence in pregnant and breastfeeding HIV-positive Cameroonian women at 6 and 12 months after initiating option B. BMC Pregnancy Childbirth. 2018;18(1):418.
96. Kafack EVF, Fokam J, Nana TN, Saniotis A, Halle-Ekane GE. Evaluation of plasma viral-load monitoring and the prevention of mother-to-child transmission of HIV-1 in three health facilities of the Littoral region of Cameroon. PLoS One. 2022;17(11):e0277271.
97. Tegueni K, Gbeasor-Komlanvi FA, Adama OIW, Sadio AJ, Amenyah-Ehlan AP, Dagnra CA, et al. Epidemiological and virological surveillance of the prevention of mother-to-child transmission of HIV among pregnant women in Togo. BMC Pregnancy Childbirth. 2024;24(1):278.
98. Flynn PM, Taha TE, Cababasay M, Butler K, Fowler MG, Mofenson LM, et al. Association of Maternal Viral Load and CD4 Count With Perinatal HIV-1 Transmission Risk During Breastfeeding in the PROMISE Postpartum Component. J Acquir Immune Defic Syndr. 2021;88(2):206-13.
99. Mazuguni N, Mazuguni F, Muro E. Detectable viral load among HIV -1 positive pregnant women on ART (Option B +) in northern Tanzania: Baseline results from the HIVDR study. East Africa Science. 2021;3:44-50.

100. Yotebieng M, Mpody C, Ravelomanana NL, Tabala M, Malongo F, Kawende B, et al. HIV viral suppression among pregnant and breastfeeding women in routine care in the Kinshasa province: a baseline evaluation of participants in CQI-PMTCT study. Journal of the International AIDS Society. 2019;22(9):e25376.
101. Adeniyi OV, Obi CL, Goon DT, Iweriebor B, Selanto-Chairman N, Carty C, et al. Factors associated with peripartum virologic suppression in Eastern Cape Province, South Africa: a retrospective cross-sectional analysis. Clinical Infectious Diseases. 2021;73(10):1750-8.
102. Boisson-Walsh A, Ravelomanana NL, Tabala M, Malongo F, Kawende B, Babakazo P, et al. Association of comprehensiveness of antiretroviral care and detectable HIV viral load suppression among pregnant and postpartum women in the Democratic Republic of the Congo: a cross-sectional study. Frontiers in Global Women's Health. 2024;5:1308019.
103. Endalamaw Alamneh D, Shiferaw MB, Getachew Demissie M, Emiru MA, Zemene Kassie T, Endaylalu Lakew K, et al. Virological outcomes among pregnant women receiving antiretroviral treatment in the Amhara Region, North West Ethiopia. HIV/AIDS-Research and Palliative Care. 2023:209-16.
104. Kabami J, Balzer LB, Kagoya F, Okiring J, Nangendo J, Ruhamyankaka E, et al. A multicomponent, peer-led intervention for pregnant and postpartum women with HIV improves viral suppression over time in rural Uganda. AJE Advances: Research in Epidemiology. 2025;1(3):uuaf017.
105. Kabami J, Akatukwasa C, Kabageni S, Nangendo J, Byamukama A, Atwiine F, et al. “I desire to have an HIV-free baby”: pregnant and breastfeeding mothers’ perceptions of Viral load testing and suppression in HIV care in southwestern Uganda. Discover Social Science and Health. 2024;4(1):60.
106. Koss CA, Natureeba P, Kwarisiima D, Ogena M, Clark TD, Olwoch P, et al. Viral suppression and retention in care up to 5 years after initiation of lifelong ART during pregnancy (Option B+) in rural Uganda. JAIDS Journal of Acquired Immune Deficiency Syndromes. 2017;74(3):279-84.
107. Gabagaya G, Rukundo G, Amone A, Wavamunno P, Namale-Matovu J, Lubega I, et al. Prevalence of undetectable and suppressed viral load in HIV-infected pregnant women initiating Option B+ in Uganda: an observational study nested within a randomized controlled trial. BMC infectious diseases. 2021;21(1):907.
108. Chohan BH, Ronen K, Khasimwa B, Matemo D, Osborn L, Unger JA, et al. Food insecurity, drug resistance and non-disclosure are associated with virologic non-suppression among HIV pregnant women on antiretroviral treatment. PloS one. 2021;16(8):e0256249.
109. Namaganda MM, Mukasa Kafeero H, Nakatumba Nabende J, Kateete DP, Batte C, Wanyengera M, et al. Prevalence and predictors of virological failure among the people living with HIV on antiretroviral treatment in East Africa: evidence from a systematic review with meta-analysis and meta-regression of published studies from 2016 to 2023. HIV Research & Clinical Practice. 2025;26(1):2490774.
110. Aytenew TM, Asferie WN, Ejigu N, Birhane BM, Tiruneh YM, Kassaw A, et al. Virological failure and associated factors among patients receiving anti-retroviral therapy in Ethiopia: A systematic review and meta-analysis. BMJ open. 2024;14(11):e087569.

111. Taieb F, Madec Y, Cournil A, Delaporte E. Virological success after 12 and 24 months of antiretroviral therapy in sub-Saharan Africa: Comparing results of trials, cohorts and cross-sectional studies using a systematic review and meta-analysis. PloS one. 2017;12(4):e0174767.
112. Melak D, Bayou FD, Yasin H, Zerga AA, Wagaye B, Ayele FY, et al. Virological Suppression and its Predictors Among HIV/AIDS Patients on Antiretroviral Therapy in Ethiopia: Systematic Review and Meta-analysis. Open Forum Infectious Diseases. 2024;11(4).
113. Mor G, Cardenas I. The immune system in pregnancy: a unique complexity. American journal of reproductive immunology. 2010;63(6):425-33.
114. Hodel EM, Marzolini C, Waitt C, Rakhmanina N. Pharmacokinetics, placental and breast milk transfer of antiretroviral drugs in pregnant and lactating women living with HIV. Current pharmaceutical design. 2019;25(5):556-76.
115. Mosha IH, Nyondo GG, Munishi CG, Njiro BJ, Bwire GM. Prevalence and factors associated with viral non-suppression in people living with HIV receiving antiretroviral therapy in sub-Saharan Africa: A systematic review and meta-analysis. Reviews in medical virology. 2024;34(3):e2540.
116. Jiang W. Adverse pregnancy outcomes, non-retention, eligibility of differentiated care and virologic failure among women living with HIV in prevention of vertical transmission programs: University of Washington; 2023.
117. Ankunda C. Longitudinal Analysis of Viral Suppression Before, During, and After Pregnancy Among Women on Antiretroviral Therapy in Uganda: Six-year Real-World Evidence. 2025.
118. Ngoufack Jagni Semengue E, NKA AD, Tueguem PP, Ka'e CA, Molimbou E, Forgwei LY, et al. Gaps, challenges and opportunities towards achieving the 95-95-95 targets in Cameroon: A systematic review and meta-analysis protocol. PloS one. 2025;20(2):e0317850.
119. Mukuku O, Govender K, Kulohoma B, Wembonyama SO. Challenges in reaching the UNAIDS 95-95-95 targets in sub-Saharan Africa: status, innovations and pathways forward. Frontiers; 2025. p. 1701425.
120. Nyongesa MK, Mwatasa MH, Kagonya VA, Mwambingu G, Ngetsa C, Newton C, et al. HIV virological non-suppression is highly prevalent among 18- to 24-year-old youths on antiretroviral therapy at the Kenyan coast. BMC Infect Dis. 2022;22(1):449.
121. USAIDS. Young people and HIV. accessed on: https://www.unaids.org/sites/default/files/media_asset/young-people-and-hiv_en.pdf 2021 [
122. Hlophe LD, Tamuzi JL, Shumba CS, Nyasulu PS. Barriers and facilitators to anti-retroviral therapy adherence among adolescents aged 10 to 19 years living with HIV in sub-Saharan Africa: A mixed-methods systematic review and meta-analysis. PLoS One. 2023;18(5):e0276411.
123. Han WM, Law MG, Egger M, Wools-Kaloustian K, Moore R, McGowan C, et al. Global estimates of viral suppression in children and adolescents and adults on antiretroviral therapy adjusted for missing viral load measurements: a multiregional, retrospective cohort study in 31 countries. Lancet HIV. 2021;8(12):e766-e75.
124. Ruel T, Mwangwa F, Balzer LB, Ayieko J, Nyabuti M, Mugoma WE, et al. A multilevel health system intervention for virological suppression in adolescents and young

adults living with HIV in rural Kenya and Uganda (SEARCH-Youth): a cluster randomised trial. The Lancet HIV. 2023;10(8):e518-e27.
125. Johnson-Peretz J, Lebu S, Akatukwasa C, Getahun M, Ruel T, Lee J, et al. “I was still very young”: agency, stigma and HIV care strategies at school, baseline results of a qualitative study among youth in rural Kenya and Uganda. Journal of the International AIDS Society. 2022;25:e25919.
126. Deeks SG, Lewin SR, Havlir DV. The end of AIDS: HIV infection as a chronic disease. The lancet. 2013;382(9903):1525-33.
127. Mengesha MM, Teshome A, Ajema D, Tura AK, Hallström IK, Jerene D. The association between HIV diagnosis disclosure and adherence to anti-retroviral therapy among adolescents living with HIV in Sub-Saharan Africa: A systematic review and meta-analysis. Plos one. 2023;18(5):e0285571.
128. Lamb MR, Fayorsey R, Nuwagaba-Biribonwoha H, Viola V, Mutabazi V, Alwar T, et al. High attrition before and after ART initiation among youth (15–24 years of age) enrolled in HIV care. Aids. 2014;28(4):559-68.
129. Salou M, Dagnra AY, Butel C, Vidal N, Serrano L, Takassi E, et al. High rates of virological failure and drug resistance in perinatally HIV-1-infected children and adolescents receiving lifelong antiretroviral therapy in routine clinics in Togo. Journal of the International AIDS Society. 2016;19(1):20683.
130. Duri K, Munjoma PT, Mazhandu AJ, Marere T, Gomo E, Banhwa S, et al., editors. Predictors and Timing to Viral Suppression in HIV-Infected Pregnant Women in the University of Zimbabwe Birth Cohort Study During the Era of Lifelong Antiretroviral Therapy (Option B+ Treatment Strategy). Frontiers in Virology; 2022.
131. Duri K, Munjoma PT, Mazhandu AJ, Marere T, Gomo E, Banhwa S, et al. Predictors and timing to viral suppression in HIV-infected pregnant women in the university of Zimbabwe birth cohort study during the era of lifelong antiretroviral therapy (Option B+ Treatment Strategy). Frontiers in Virology. 2022;2:838234.
132. Dessie G, Wagnew F, Mulugeta H, Amare D, Jara D, Leshargie CT, et al. The effect of disclosure on adherence to antiretroviral therapy among adults living with HIV in Ethiopia: a systematic review and meta-analysis. BMC Infect Dis. 2019;19(1):528.
133. Endalamaw A, Assefa Y, Geremew D, Belete H, Dachew BA, Belachew A, et al. Disclosure of HIV seropositivity to sexual partner in Ethiopia: A systematic review. Womens Health (Lond). 2021;17:17455065211063021.
134. Mekonnen FA, Lakew AM, Muchie KF, Teshome DF. Sero-positive HIV result disclosure to sexual partner in Ethiopia: a systematic review and meta-analysis. BMC Public Health. 2019;19(1):1743.
135. Jones HS, Floyd S, Stangl A, Bond V, Hoddinott G, Pliakas T, et al. Association between HIV stigma and antiretroviral therapy adherence among adults living with HIV: baseline findings from the HPTN 071 (PopART) trial in Zambia and South Africa. Tropical Medicine & International Health. 2020;25(10):1246-60.
136. Perger T, Davtyan M, Foster C, Evangeli M, Berman C, Kacanek D, et al. Impact of HIV-related stigma on antiretroviral therapy adherence, engagement and retention in HIV care, and transition to adult HIV Care in Pediatric and Young Adult Populations Living with HIV: a literature review. AIDS and Behavior. 2025;29(2):497-516.

137. Zhao S, Wang W, Li S, He J, Duan W, Fang Z, et al. The prevalence of low-level viraemia and its association with virological failure in people living with HIV: a systematic review and meta-analysis. Emerging Microbes & Infections. 2025;14(1):2447613.
138. Kibui NK. Factors Associated With Virological Non-suppression in Treatment-experienced HIV+ Patients in Kenya: a Populationwide Study: University of Nairobi; 2024.
139. Ministry of Health E. National Guideline for Prevention of Mother-to-child Transmission of HIV, Syphilis and Hepatitis B Virus. Access: https://www.prepwatch.org/wp-content/uploads/2022/07/National-Guideline-for-Prevention-of-Mother-to-child-Transmission-of-HIV-Syphilis-and-Hepatitis-B-Virus-2021.pdf 2021 [
140. Yang J, Zhao X, Li F. INSTIs-centered antiviral regimens for first-line treatment of HIV/AIDS: a network meta-analysis and cost-effectiveness analysis. BMC Infect Dis. 2025;25(1):604.
141. Fleșeriu T, Meliț LE, Mărginean CO, Pop AV, Văsieșiu AM. Maternal HIV Infection and Antiretroviral Therapy in Pregnancy: Implications for Vertical Transmission, Fetal Safety, and Long-Term Infant Outcomes. Pathogens. 2025;14(8).
142. Fassinou LC, Songwa Nkeunang D, Delvaux T, Nagot N, Kirakoya-Samadoulougou F. Adherence to option B + antiretroviral therapy and associated factors in pregnant and breastfeeding women in Sub-Saharan Africa: a systematic review and meta-analysis. BMC Public Health. 2024;24(1):94.
143. Sibiude J, Le Chenadec J, Mandelbrot L, Hoctin A, Dollfus C, Faye A, et al. Update of perinatal human immunodeficiency virus type 1 transmission in France: zero transmission for 5482 mothers on continuous antiretroviral therapy from conception and with undetectable viral load at delivery. Clinical infectious diseases. 2023;76(3):e590-e8.
144. Nachega JB, Marconi VC, van Zyl GU, Gardner EM, Preiser W, Hong SY, et al. HIV treatment adherence, drug resistance, virologic failure: evolving concepts. Infect Disord Drug Targets. 2011;11(2):167-74.
145. Daltro ACB, Almeida CS, Unfried AGC, de Aquino TR, Travassos AGÁ. Virological failure and adherence to antiretroviral therapy in adolescents and young adults living with human immunodeficiency virus. Tropical Medicine & International Health. 2023;28(3):162-74.
146. Gils T, Bossard C, Verdonck K, Owiti P, Casteels I, Mashako M, et al. Stockouts of HIV commodities in public health facilities in Kinshasa: Barriers to end HIV. PLoS One. 2018;13(1):e0191294.
147. Thomford NE, Mhandire D, Dandara C, Kyei GB. Promoting U ndetectable Equals U ntransmittable in Sub-Saharan Africa: Implication for Clinical Practice and ART Adherence. International journal of environmental research and public health. 2020;17(17):6163.
148. Jennings Mayo-Wilson L, Devoto B, Coleman J, Mukasa B, Shelton A, MacCarthy S, et al. Habit formation in support of antiretroviral medication adherence in clinic-enrolled HIV-infected adults: a qualitative assessment using free-listing and unstructured interviewing in Kampala, Uganda. AIDS research and therapy. 2020;17(1):30.
149. Colvin CJ, Konopka S, Chalker JC, Jonas E, Albertini J, Amzel A, et al. A systematic review of health system barriers and enablers for antiretroviral therapy (ART) for HIV-infected pregnant and postpartum women. PloS one. 2014;9(10):e108150.

150. Moriarty K, Genberg B, Norman B, Reece R. The effect of antiretroviral stock-outs on medication adherence among patients living with HIV in Ghana: a qualitative study. Journal of the Association of Nurses in AIDS Care. 2018;29(2):231-40.

S5 Table 1: Descriptive summary of studies included in systematic review of virologic suppression among HIV-positive pregnant and lactating women receiving ART in Africa.

Authors	Study design	Country	Study population	Sample size	VS (%)	JBI score
Adeniyi et al., 2021	CS	South Africa	Pregnant and postpartum	1463	82	9
Aduloju et al., 2020	CS	Nigeria	Pregnant women	170	70	7
Alamneh et al., 2023	CS	Ethiopia	Pregnant women	992	90.9	8
Anderson et al., 2024	CH	South Africa	Pregnant women	50764	87.4	8
Anderson et al., 2024	CH	South Africa	Postpartum women	50764	90.9	9
Anderson et al., 2025	CH	South Africa	Intrapartum women	50764	83.8	9
Boisson-Walsh et al et al., 2024	CS	DR Congo	Pregnant and postpartum	2295	66.5	8
Brittain et al., 2018	CS	South Africa	Pregnant women	482	88	9
Chagomerana et al., 2018	CH	Malawi	Postpartum women	252	84.1	9
Chagomerana et al., 2018	CH	Malawi	Pregnant women	864	92.4	9
Chagomerana et al., 2021	CH	Malawi	Pregnant women	819	86.5	9
Chagomerana et al., 2022	CH	Malawi	Postpartum women	102	88.9	9
Chetty et al., 2017	CH	South Africa	Pregnant and postpartum	3941	83.2	8
Chetty et al., 2018	CH	South Africa	Pregnant women	150	77.3	8
Chetty et al., 2019	CH	South Africa	Postpartum women	150	80.7	8
Demissie et al., 2020	CS	Ethiopia	Pregnant women	319	89.7	9
Duri et al., 2020	CH	Zimbabwe	Pregnant women	608	78.1	8
Gabagaya et al., 2021	CH	Uganda	Pregnant women	532	29.1	9
Hailu et al., 2025	CS	Ethiopia	Pregnant women	7764	96.8	9
Kafack et al., 2022	CH	Cameroon	Pregnant women	135	89.7	8
Koss et al., 2017	CH	Uganda	Pregnant women	150	80	9
Landes et al., 2019	CS	Malawi	Postpartum women	1124	87.9	8
Landes et al., 2021	CH	Malawi	Postpartum women	773	87.4	8
Langwenya et al., 2018	CH	South Africa	Pregnant and postpartum	428	77	7

M. Flynn et al., 2021	CH	SSA	Postpartum women	1220	75	8
M. Gill et al., 2016	CH	Rwanda	Pregnant women	603	84.6	8
M. Humphrey et al., 2022	CH	Kenya	Pregnant women	167	88	8
M. Humphrey et al., 2023	CH	Kenya	Pregnant women	689	93	8
Mazuguni et al., 2021	CS	Tanzania	Pregnant women	148	77	9
Moyo et al., 2020	CS	South Africa	Pregnant women	2769	77.6	8
Moyo et al., 2020	CH	South Africa	Pregnant women	40660	79.2	8
Moyo et al., 2020	CH	South Africa	Intrapartum women	40660	85.7	8
Munyaneza et al., 2025		Rwanda	Both women	530	91.9	8
Musanhu et al., 2022		Zimbabwe	Pregnant women	121	87.7	8
Myer et al., 2015	CS	South Africa	Pregnant women	574	87	9
Myer et al., 2017	CH	South Africa	Pregnant women	523	78	9
Myer et al., 2017	CS	South Africa	Pregnant women	620	91	9
N. Atanga et al., 2018	CH	Cameroon	Pregnant women	268	92.7	9
Napyo et al., 2020	CS	Uganda	Pregnant women	420	91.9	9
Ndlangamandla et al., 2023	CS	Estiwani	Pregnant women	195	81.8	7
Ngandu et al., 2022	CS	South Africa	Pregnant and postpartum	667	85.3	9
Ntombela et al., 2022	CS	South Africa	Pregnant women	546	90.8	7
Phillips et al., 2017	CS	South Africa	Pregnant and postpartum	452	92	9
Sandbulte et al., 2020	CH	Kenya	Pregnant women	115	71	8
Schrubbe et al., 2021	CS	SSA	Pregnant and postpartum	1685	63.8	8
Schrubbe et al., 2022	CS	SSA	Pregnant women	560	59.9	8
Schrubbe et al., 2023	CS	SSA	Postpartum women	1129	66	8
Tsondai et al., 2016	CH	South Africa	Pregnant women	520	88	7
Weldesenbet et al., 2020	CS	South Africa	Pregnant women	10052	73	8
Woldesemayat et al., 2024	CS	Ethiopia	Pregnant women	496	97.8	8
Woldesenbet et al., 2022	CS	South Africa	Pregnant women	8087	69.4	9
Woldesenbet et al., 2022	CS	South Africa	Pregnant women	9733	73.3	9
Yotebieng et al., 2019	CS	DR Congo	Pregnant women	1623	62	9
Yotebieng et al., 2020	CS	DR Congo	Intrapartum women	1623	57	9
Yotebieng et al., 2020	CS	DR Congo	Postpartum women	1623	65	9

Key: CS: Cross-sectional studies, CH: cohort studies, DR: Democratic Republic Congo, SSA: Sub-Saharan AfricA

Table 21: Sub-group analysis for virologic suppression among HIV-positive pregnant and lactating women receiving ART in Africa.

Parameters	Studies	Samples	VS (%) at 95% CI	I^2 (%)	P-value
Study design					
Cohort studies	27		82.74% (80.80, 84.69)	99.35%	0.000
Cross-sectional studies	28		79.34% (74.06, 84.63)	99.69%	0.000
**Country**					
Cameroon	2		91.89% (89.23, 94.55)	0.00	0.327
DR Congo	4		62.66% (58.56, 66.75)	92.49	0.000
Ethiopia	4		94.11% (91.16, 97.07)	94.97	0.000
Kenya	3		84.82% (74.86, 94.78)	92.80	0.000
Malawi	6		88.06% (85.62, 90.51)	80.81	0.000
SSA	3		66.28% (60.04, 72.51)	95.09	0.000
South Africa	21		82.8% (80.42, 85.21)	99.64	0.000
Uganda	3		66.99% (23.93, 110.05)	99.72	0.000
Rwanda	2		88.30% (81.15, 95.46)	93.31	0.000
Zimbabwe	2		82.58% (73.20, 91.97)	87.27	0.005
Others*	3		76.52% (69.68, 83.35)	71.31	0.031
**Regions of Africa**					
Central Africa	4		62.66% (58.56, 66.75)	92.49	0.000
East Africa	13		83.49% (77.69, 89.3)	99.09	0.000
Sub-Saharan Africa	4		66.28% (60.04, 72.51)	95.09	0.000
Southern Africa	31		83.74% (81.72, 85.76)	99.47	0.000

Western Africa	3		84.49% (72.77, 96.20)	94.24	0.000
**Study population**					
Pregnant women	38		81.15% (78.66, 83.63)	99.61	0.000
Postpartum women	9		80.61% (73.28, 87.94)	99.08	0.000
Both	8		80.21% (73.25, 87.18)	98.87	0.000

*Nigeria, Estiwani, Tanzania

Table 1: Meta-regression for factors correlated with virologic suppression among HIV-positive pregnant and lactating women receiving antiretroviral therapy in Africa.

Variables	β-coefficient	Std. error	z	P-value	95% confidence interval
Publication year<br>Before 2020	-2.73	3.55	-0.77	0.443	-9.69, 4.24
ART regimen<br>DTG-based	16.43	6.55	2.51	0.012	3.58, 29.26
Country	-0.24	0.183	-1.33	0.183	-0.60, 0.12
Region of Africa	-2.92	1.40	-2.08	0.037	-5.66, -0.17
Sample size	-0.001	0.0002	-1.30	0.195	-0.0005, 0.0001
Constant	91.48	5.77	15.85	0.000	80.17, 102.80

Table 3: Meta-analysis for factors associated virologic suppression among HIV-positive pregnant and lactating women on antiretroviral therapy in Africa.

Variables	No_ studies	AOR (95% CI)	I^2 (%)	P-value
Maternal age<br>≥25 years (Ref.)<br>15-24 years	4	0.49 (0.32, 0.77)	79.7%	0.002
Disclosing HIV status to partner	4	1.66 (1.31, 2.11)	0.00%	0.04
ART type<br>First line ART<br>Second and/ third line ART (Ref.)	3	6.53 (1.93, 22.03)	86.07%	0.001
Adherence status to ART<br>Good<br>Poor (Ref.)	5	3.61 (1.18, 11.02)	94.6%	0.001

December 24, 2025

Virologic suppression among HIV-positive pregnant and lactating women receiving antiretroviral therapy in Africa: A systematic review and meta-analysis

Manuscript number: PONE-D-25-49195

Dear **Richard Makurumidze (MBChB, MPH, PhD, FCPHP, Academic Editor),** we are delighted to receive your comments for our submission to PLOS One. We are also grateful for the honored reviewers; their scientific comments, expertise, and guidance have been fundamental for enhancing the overall quality of our paper. We have diligently addressed the raised comments and suggestions given by the reviewers. All the amendments highlighted using tracked changes. The changes have been fully included in the revised manuscript. Finally, we kindly invite you to take a look at a point-by-point response below:

Point-by-point response to Reviewers' comments

Reviewer #1: The manuscript addresses a critical public health concern by evaluating virologic suppression among HIV-positive pregnant and lactating women in Africa. Its significance is evident given the implications for mother-to-child transmission and the broader context of achieving global HIV targets. The inclusion of 55 studies and over 300,000 participants strengthen the analysis through a large sample size, and the use of PRISMA guidelines, PROSPERO registration, and appropriate statistical tools such as random-effects models and Egger's tests demonstrate methodological rigor. The study presents relevant findings with public health utility, highlighting key factors such as age, adherence, disclosure of HIV status, and ART regimen as significantly associated with viral suppression.

Authors' response: Dear Reviewer, we would like to express our sincere gratitude for your valuable time and constructive feedback to our manuscript. We greatly appreciate your insightful comments and for sharing your expertise to improve our paper. In response to the reviewer's thoughtful comments and suggestions, we have carefully and thoroughly addressed each of the

points raised. As a result, we carried out a substantial revision throughout the manuscript and we have addressed all concerns raised by the reviewer as much as possible.

Reviewer's comment: Despite these strengths, several issues warrant attention. A major concern lies in the extremely high heterogeneity ($I^2 > 99\%$), which remains unexplained even after subgroup analyses. The use of meta-regression would have been appropriate to explore potential sources of heterogeneity more systematically. The overrepresentation of studies from South Africa introduces geographical bias, making it difficult to generalize findings across the diverse healthcare contexts of the African continent. Furthermore, although the authors state there was no publication bias, the Egger's test result ($p = 0.0512$) suggests borderline significance, which should not be dismissed as negligible without further investigation

Authors' response: Dear reviewer, thank you for these thoughtful comments. We acknowledge the high heterogeneity and agree that meta-regression would have been valuable. As a result, we have performed meta-regression analysis to examine potential moderators for the high heterogeneity. We also recognize the overrepresentation of studies in South African and have clearly noted its potential effect on generalizability of the finding as a limitation. Regarding assessing publication bias, we have re-analyzed using a random-effects model with Restricted maximum–likelihood (REML). Then, the statistical test (Egger's test) shows non-significant.

Reviewer's comment: The study also overlooks key confounding factors such as socioeconomic status, healthcare accessibility, or rural–urban disparities, which are known to influence ART outcomes. In addition, the reporting lacks clarity in some areas.

Authors' response: Thank you for the comment. We were constrained by the variables mentioned in the original papers because our review is based on secondary data from preexisting studies, making it tough to extract and identify all possible factor affecting virologic suppression in pregnant and lactating women. However, in response to the reviewer's comment, we strengthened our systematic synthesis by including additional variables when the available data permitted. The results and discussion sections have undergone significant revisions.

Reviewer's comment: The year range for included studies curiously extends to 2025, which undermines credibility and suggests a typographical or planning oversight. The quality

assessment using the Joanna Briggs Institute tool is not detailed, and no summary of individual study scores is provided, which limits transparency.

Authors' response: Thank you. Thank you for this helpful comment. The study period of 2016–2025 is correct, as our review includes all eligible publications up to 2025. We have clarified this as all articles between January 1, 2016 (benchmark for the beginning of the Sustainable Development Goals (SDG) era). We also appreciate the feedback on the quality assessment and have added the Joanna Briggs Institute criteria with a summary of each study scores in S4 file.

Reviewer's comment: Wide confidence intervals for certain effect estimates (e.g., ART adherence) further point to imprecision or potential small-study effects.

Authors' response: We appreciate your insightful comment. Exactly, the possible reason for slightly wide CI could be related with number of studies included. Along with the meta-analysis, we try to carry out a systematic synthesis to elaborate factors associated with VS.

Reviewer's comment: Narratively, the discussion is repetitive, often restating results without providing deeper contextual interpretation or drawing clear distinctions between policy implications and existing literature. The paper also misses the opportunity to provide targeted, practical recommendations for policymakers, especially considering its relevance to achieving UNAIDS 95-95-95 targets. Finally, factors such as ART duration, viral load assay variability, and breastfeeding practices, which could significantly influence virologic suppression, are insufficiently addressed. Overall, while the study presents valuable findings, it would benefit from a more nuanced analytical approach, clearer methodological transparency, and a stronger focus on contextual and actionable insights.

Authors' response: We appreciate the reviewer's insightful and helpful comments. As a result, we changed the Discussion to focus more on understanding and contextualizing within the body of current literature and less on repetition. We improve the link between our findings and advancement toward the UNAIDS 95–95–95 targets and more clearly differentiate implications for policy from previous studies. Additionally, we included more detailed and practical suggestions for decision-makers. We also went into more detail on important variables that affect virologic suppression, including as study socioeconomic status, viral load assay variability, and

the duration of ART. Overall, these revisions could enhance the analytical rigor, transparency, and practical relevance of the manuscript.

Reviewer #2: thank you for your interested topic

The study is scientifically relevant, methodologically solid, and has potential impact for HIV programmatic policy in Africa. I advise to Revise for minor grammar mistakes, consistent abbreviation use, and concise phrasing.

Authors' response: Dear Reviewer #2, thank you so much for your comments on grammatical errors, consistency and precision. We have considered the reviewer's comments throughout the manuscript to enhance its rigorous and clarity.

Reviewer #3: 1. Does the title accurately reflect the study's objectives and population?

Authors' response: We appreciate the reviewer's comment. We agree that the title adequately describes the study's emphasis and objectives. It precisely identifies the target population (HIV-positive pregnant and lactating women on ART), the study area (Africa), and the outcome of interest (virologic suppression), all of which are in line with the objectives of our systematic review and meta-analysis.

Reviewer #3: 2. The abstract mentions an 80.92% pooled prevalence should confidence intervals or heterogeneity measures (I2) also appear for completeness?

Authors' response: Thank you! We have stated the confidence intervals and heterogeneity measures (I^2) in the abstract section.

Reviewer #3: 3. Were the inclusion and exclusion criteria too broad (2016–2025) given the variability in ART regimens over time?

Authors' response: Thank you for your insightful comment. While our inclusion period spans years in which ART regimens evolved, most included studies were based on the Option B+ regimen. Only a few studies were in the current first-line regimen (TDF-3TC-DTG). To address this variability, we conducted a meta-analysis analysis by ART regimen, allowing us to account for differences in treatment protocols over time. As a result, ART regimen was found one of the moderators or covariates for virologic suppression. We have included all the changes in the revised manuscript.

Reviewer #3: 4. How were overlapping datasets or duplicate reports from large cohort programs handled?

Authors' response: Thank you for raising this important point. We thoroughly examined each study for overlapping datasets to prevent duplication. We only included the dataset that offered the most pertinent and non-overlapping data for our results when reports were from the same cohort program. We only considered publications that examined different time periods (pregnancy, intrapartum, or postpartum) to be independent if the populations did not overlap. Furthermore, other studies have reported the VS for certain study populations, such as breastfeeding mothers and pregnant women, at a particular point in time.

Reviewer #3: 5. Were data extraction and bias assessments blinded and independently verified by more than two reviewers?

Authors' response: Absolutely! Data extraction from the included publications was performed independently by two authors (BAM and NTT).

Reviewer #3: 6. How did the authors deal with variations in the definition of "virologic suppression" across studies (e.g., ≤50 vs ≤1000 copies/mL)?

Authors' response: I appreciate you making this crucial insight. By using the WHO definition, which defines virologic suppression as VL ≤1000 copies/mL, we were able to address differences in criteria. Furthermore, we extracted data independently and computed a pooled estimate of undetectable viral load for studies that reported VL ≤50 copies/ml. This approach helps us to harmonize the cutoff points for viral load thresholds.

Reviewer #3: 7. Given the high heterogeneity (I2 = 99.57%), was the use of a pooled random-effects model justified without meta-regression?

Authors' response: Thank you for highlighting this concern. We used a random-effects model because substantial methodological and contextual differences across studies were anticipated. To further explore the observed high heterogeneity, we conducted subgroup analyses based on key study characteristics (ART regimen, study design, and geographic region). Thank you for raising this important point. In addition, we conducted a meta-regression using sample size, study period, country, ART regimen, and African region as covariates to explore sources of

heterogeneity. ART regimen, and regions of Africa were found significant covariate for virologic suppression. All changes have been included in the revised manuscript.

Reviewer #3: 8. Could differences in study design (cross-sectional vs. cohort) have contributed to heterogeneity, and were these tested?

Authors' response: Thank you for this insightful comment. We conducted a subgroup analysis to see if the study design was a factor in the reported heterogeneity. For both cohort and cross-sectional studies, the pooled prevalence was 82.74% and 79.34%, respectively. The I-square remains significantly high in both groups (99.35% and 99.69%, respectively), despite the fact that the prevalence estimates are comparatively equal across the two study designs. This suggests that the statistical heterogeneity in this meta-analysis cannot be explained by the differences between cross-sectional and cohort designs.

Reviewer's comment: 9. how were missing data handled in the included studies?

Authors' response: Thank you for your insightful comment. Our analysis and findings relied on the reported data of the original studies. The majority of included studies state how missing data were handled or excluded the study subject with missing viral load values from their analyses. Due to the lack of individual participant-level data, we did not do extra imputation for missing outcome data.

Reviewer's comment: Could differences in viral-load testing availability or assay methods influence reported suppression rates?

Authors' response: Thank you very much for this scientific comment. We appreciate the reviewer's insightful comment regarding the influence of viral load (VL) testing availability and assay methods. We agree that these are critical determinants for regular assessment of VL. For instance, limited access to routine testing often results in 'targeted' testing for virologic failure.

Reviewer #4: Thank you for the opportunity to review this manuscript.

The topic, "virologic suppression among HIV-positive pregnant and lactating women in Africa" is important and policy-relevant. With a few targeted methodological clarifications and stronger transparency, this can become a solid, reproducible synthesis.

Authors' response: Thank you very much for your time and effort to review our manuscript. We express our gratitude to Reviewer #4 for giving extensive evaluation of our work and for acknowledging the significance and policy relevance of virologic suppression among pregnant and breastfeeding women in Africa. We value the constructive feedback that was provided. In response to your recommendations, we have improved the transparency of our reporting and included the necessary methodological explanations that ensure that the manuscript fulfills the highest requirements for a reproducible synthesis. We have diligently revised the main manuscript and our detailed responses to your specific points are provided below.

Reviewer #4: Major comments

1) Outcome definition & measurement window

Please harmonize the outcome definition and clarify, at study level, the VL threshold used (e.g., ≤1000 vs other), specimen type (plasma vs DBS), and timing (pregnancy trimester / intrapartum / postpartum window). Provide either:

• A succinct subgroup/meta-regression using these moderators; or

• A sensitivity analysis restricted to ≤1000 copies/ml.

This avoids definitional mixing and improves interpretability.

Authors' response: Thank you for your valuable comments.

We thank the reviewer for this important suggestion to improve the precision and transparency of our findings. We have addressed these points as follows:

To ensure consistency, our primary meta-analysis was restricted to studies using the standard threshold of ≤1000 copies/ml. We have performed a sensitivity analysis exclusively including studies that strictly utilized this threshold, which confirmed the robustness of our pooled prevalence estimates.

We recognize that virologic suppression (VS) could fluctuate across the continuum maternity care. Accordingly, we have conducted a subgroup analysis categorized by the timing of measurement: pregnancy (by trimester where available), intrapartum, and postpartum periods.

However, the subgroup analysis did not show significant variation in the VS. Additionally, based on the included studies, viral load threshold was determined commonly utilized blood plasma.

2) Modeling proportions under extreme heterogeneity

Given the very high between-study heterogeneity ($I^2 \approx 99.6\%$), please complement Der Simonian–Laird with HKSJ or REML, and add a logit/GLMM approach suitable for proportions (with appropriate continuity correction where needed).

In the main text, report τ^2 and a 95% prediction interval (PI) alongside the pooled mean.

Authors' response: Thank you for this valuable comment. We compared pooled estimates of viral load suppression using three random-effects meta-analysis estimators: Der Simonian–Laird (DL), Sidik–Jonkman (SJ), and restricted maximum likelihood (REML). All analyses included 55 studies and demonstrated extremely high between-study heterogeneity. Across all three methods, the pooled estimates were remarkably consistent, with point estimates centered around 81% VS. The Der Simonian–Laird method yielded a pooled estimate of 80.92% (95% CI: 78.92–82.91), while both REML and Sidik–Jonkman produced nearly identical pooled estimates of 80.86%, with slightly wider CI (REML: 77.63, 84.09; Sidik–Jonkman: 77.63, 84.08). Given the extreme heterogeneity observed ($I^2 > 99\%$), REML was prioritized for inference due to its favorable statistical properties and reduced bias in estimating between-study variance.

Additionally, in the revised manuscript, we have stated the between-study variance (tau, τ^2) and the 95% Prediction Interval (PI) alongside the pooled estimate. However, important differences were observed in the estimation of between-study heterogeneity (τ^2) among the models. The Der Simonian–Laird method results a substantially lower heterogeneity ($\tau^2 = 53.92$) compared with REML ($\tau^2 = 146.21$) and Sidik–Jonkman ($\tau^2 = 145.81$). These findings could be due to the well-documented methodological limitations of the DL estimator, which tends to underestimate τ^2 in the presence of extreme heterogeneity. For this reason, we are fully convinced that the REML approach provides a more accurate and less biased estimate of virologic suppression, and we have accordingly applied it consistently across the revised manuscript, from the abstract to the conclusion section of the manuscript.

Reviewer #4: 3) Explaining heterogeneity rather than averaging it away

Your region/country subgrouping is helpful. If feasible, consider a compact set of clinically/programmatically meaningful moderators, ART era (EFV vs DTG rollout) and maternal age (15-24 vs ≥25 years), via simple stratified analyses or a brief meta-regression.

If further analyses are not practical, a short justification for the current scope plus a more cautious interpretation (with the PI) is acceptable.

Authors' response: Thank you for your suggestion. We agree with the reviewer that explaining the sources of heterogeneity is more informative than simply reporting the overall estimate of VS. Even though we encountered to extract consistently categorized data from the primary studies, we try our utmost effort to improve the quality of this manuscript. As per the comment, we have conducted stratified analyses to explore clinically important moderators. This includes regions of Africa, Country, and ART era (comparing the DTG-based and non-DTG based). These results are stated in the result and discussion section (Table 2: Meta-regression).

Reviewer #4: 4) Small-study effects / publication bias

Under heavy heterogeneity, Egger is suboptimal for single-arm proportions. Please consider Peters/Harbord tests where the number of studies permits and temper funnel-plot interpretations accordingly; if underpowered, state this explicitly.

Authors' response: We thank the reviewer for this valuable methodological suggestion. We considered using Peters and Harbord tests for assessing publication bias, however we opted to retain Egger's test. Peters and Harbord tests are generally preferred for binary outcome variables; our study focuses on prevalence estimate. For this reason, we retained Egger's test, which remains a robust approach for assessing publication bias when the number of studies is sufficient (>30 studies).

Reviewer #4: 5) Unit of analysis & potential double counting

Where a single dataset contributes multiple effects (e.g., pregnant vs intrapartum vs postpartum; or multiple VL thresholds), please explain how you avoided double counting, for example, by pre-specifying one time-point, aggregating within-study effects, or using a multilevel model.

Authors' response: Thank you very much for this crucial point. We considered virologic suppression (VS) as independent across pregnancy, intrapartum, and postpartum (breastfeeding),

allowing each effect to be examined independently, in order to mitigate the possibility of double counting. To ensure that each study contributed fairly without inflating the outcome, we combined these time-specific effects to determine the overall pooled estimate of VS for these groups of population.

Reviewer's comment: 6) Risk of bias & transparency (search, RoB, data/code**)**

Include a per-study JBI risk-of-bias matrix in the supplement, justify any scoring thresholds, and add a sensitivity analysis excluding higher-risk studies. For search reproducibility, provide full search strings, last search date, and the deduplication procedure; also clarify whether Embase/Scopus and Africa-focused sources (e.g., AIM/AJOL) were searched or provide a documented rationale for their exclusion.

For reproducibility, make the extraction sheet (study-level events/total and moderators) fully available per PLOS policy (as SI or in a DOI-minted repository). Sharing analysis code (Stata/R) is strongly encouraged; at minimum, include sufficient model/command detail for re-analysis.

Authors' response: Authors' response: Thank you once again. We have stated the use of PubMed as a main database for literature search. PubMed is a widely recognized biomedical database for accessing indexed published articles. To ensure comprehensive searching and bridge potential gap for using PubMed, we augmented our search by systematically exploring Google Scholar to identify both published and unpublished studies, and we also used ScienceDirect to retrieve relevant articles from Elsevier-indexed journals. We have uploaded relevant data as supplementary file, including search strategy, data extraction sheet, and Stata file and code for possible re-analysis as well as journal's data availability guideline.

Authors' response: Thank you very much for this insightful suggestion.

Reviewer's comment: 7) Determinants / associated factors (AORs)

Please specify the eligibility criteria for studies entering the determinants analysis and ensure alignment of reference categories and reasonable consistency of adjustment sets before pooling on the log-OR scale. If adjustment sets differ substantially or heterogeneity is high with wide CIs, consider a narrative synthesis or meta-regression rather than a pooled AOR. Clarify why

only a subset of studies contributed and whether others were excluded for design/measure reasons.

Authors' response: Thank you dear reviewer. As a result, we have made the eligibility requirements for research that are part of the determinants analysis clear and making sure that reference categories are consistently aligned across studies. We examined adjustment sets closely and fixed any discrepancies. We used systematic synthesis for VS variables in cases with significant variations in adjustments or high variability with wide confidence intervals. We have made substantial revision in the result and discussion sections.

Reviewer's comment: Minor comments

Reviewer #4: • Move very long descriptive tables to the supplement and keep concise summary tables in the main text.

Authors' response: We sincerely appreciate your constructive feedback and valuable suggestions aimed at improving the quality of our manuscript. Following your suggestion, we have made the following changes: Table 1 (formerly describing Table 1: Descriptive summary of studies included in systematic review of virologic suppression among HIV-positive pregnant and lactating women receiving ART in Africa) has been moved to the Supplementary Material as Table S1 file.

Reviewer #4: • Include the 95% prediction interval in the Abstract/Results so readers immediately see expected variability across settings.

Authors' response: We agree that the 95% prediction interval (PI) provides essential context for interpreting the pooled prevalence in the presence of extreme heterogeneity. As requested, we have added the 95% PI to both the Abstract and the Results section of the manuscript.

Reviewer #4: • Define acronyms at first use (VS/VLS, PMTCT, DTG/EFV) and streamline a few long sentences with a light language edit.

Authors' response: We thank the reviewer for this helpful suggestion to improve the manuscript's readability. We have conducted a thorough editorial review and implemented to define acronyms at first use and concise long sentences.

Reviewer #4: • Proofread tables for numerical slips (e.g., any CI exceeding 100%) and ensure consistent terminology.

Authors' response: Response: We thank the reviewer for inviting us to re-check for typos errors. We have conducted a recheck to all tables and figures to ensure accuracy and consistency. All proportions and intervals have been corrected and rounded consistently as well as consistent use terminologies in the main text.

Reviewer #4: • Generalizability: where certain sub-analyses rely heavily on national program data, please comment on applicability to smaller or non-program settings.

Authors' response: Response: We thank the reviewer for this critical point regarding the generalizability of our findings. We acknowledge that our meta-analysis includes several large-scale studies derived from national program data, which reflect real-world implementation but may differ from smaller, controlled clinical settings.

***Reviewer #4*:** • Briefly cite the PROSPERO registration (ID) and ensure the PRISMA flow diagram is referenced in the main text for reproducibility.

Authors' response: We have ensured that the manuscript adheres to the highest standards of transparency and reproducibility. We have cited PROSPERO registration (ID) in the method section and we have reference the PRISMA flow diagram in the result section.

Reviewer #4: Recommendation: Major Revision.

The manuscript is valuable; the requested clarifications, outcome harmonization, robust proportion modeling, focused heterogeneity exploration (including ART era and age), and transparent materials (search/RoB/extraction file and, ideally, code), will make the conclusions more reliable and reproducible.

Authors' response: Thank you in advanced for your scientific and insightful comment. We appreciate the acknowledgement of the manuscript's worth. In response to these recommendations, we have made important modifications to enhance clarity, establish uniform outcomes definitions, utilize more rigorous proportion modeling approaches, and conduct targeted investigations of variability, particularly by ART period and age. We have also increased transparency by giving thorough search strategy, risk-of-bias assessments, data extraction

materials, and Stata analysis code. We believe these changes significantly improve the validity, reproducibility, and clarity of our findings.

Reviewer #5: This manuscript presents a systematic review and meta-analysis assessing the pooled prevalence of virologic suppression (VS) among HIV-positive pregnant and lactating women receiving antiretroviral therapy (ART) in Africa. The authors included 55 studies encompassing 304,883 participants across various African regions. The study followed PRISMA guidelines and used robust statistical methods, including a random-effects model, subgroup analyses, and heterogeneity assessment.

The topic is highly relevant, particularly in the context of achieving the global UNAIDS 95–95–95 targets and improving maternal and child health outcomes in sub-Saharan Africa. The manuscript contributes meaningful evidence regarding ART adherence and determinants of viral suppression in this key population group. This manuscript has major strengths such as relevance, scope and data size, systematic approach, robust analysis, policy value.

Authors' response: We truly appreciate Reviewer #5's careful and thorough assessment of our submission. We value the acknowledgement of the study's importance, scope, and rigorous methodology, as well as its contribution to our knowledge of virologic suppression in African pregnant and lactating women receiving antiretroviral therapy. We particularly grateful for the recognition of the manuscript's relevance to policy and linkage with initiatives to improve mother and child health outcomes and achieve the UNAIDS 95–95–95 targets. We really appreciate your positive remarks, which have inspired us to improve the updated manuscript's impact and clarity.

Reviewer #5: Minor Editorial Comments:

- In Abstract section, replace "remains low" with quantitative context ("approximately 81%, below global target of 95%").

Authors' response: As suggested, we have revised the Abstract to replace the phrase "remains low" with specific data.

Reviewer #5*:* Conduct meta-regression and sensitivity analysis by study design, ART regimen, and publication year.

Authors' response: In accordance with the reviewer's suggestion, we have conducted both meta-regression and sensitivity analyses to systematically explore the sources of heterogeneity.

Reviewer #5: ⎫ Provide more discussion on possible small-study or regional publication bias.

Authors' response: We thank the reviewer for your suggestion; we have expanded the discussion and acknowledge the regional concentration of studies as a limitation.

Reviewer #5: ⎫ Include qualitative evidence or secondary data review to contextualize findings.

Authors' response: Thank you. In our revised manuscript, we have carried out a systematic synthesis and integrated evidence from qualitative studies in Africa. We incorporated structural and sociocultural barriers for VS.

Reviewer #5: ⎫ Ensure all forest plots (Figures 2–8) have consistent formatting and readable axis labels.

Authors' response: We thank the reviewer for this attention to detail. We revised the formatting of forest plots (figures) to ensure a high-quality.

Reviewer #5: ⎫ For References, several citations lack full journal details or DOI (e.g., refs. 70, 77). Please verify all references conform to PLOS ONE style. Conduct sensitivity analysis by publication period (pre/post-2020).

Authors' response: We appreciate Reviewer #5's insightful and useful recommendations. To ensure that references completely adhere to PLOS ONE formatting criteria, including the completion of any missing publication details and DOIs (such as references 70 and 77), we have carefully examined and revised all references. A sensitivity analysis stratified by publication period (pre-2020 vs. post-2020) was also carried out, and the results are included in the updated manuscript.

Reviewer #5: ⎫ Minor edits needed for flow in Introduction and Discussion to grammar.

Authors' response: We thank the reviewer for this suggestion. We have conducted a substantial revision of the Introduction and Discussion sections to improve the grammatical accuracy and overall narrative flow.

Reviewer #5: Closing Statement:

This manuscript is a well-executed, timely, and policy-relevant meta-analysis that adds significant value to the understanding of virologic suppression among HIV-positive women in Africa.

With minor clarifications on methodology, expanded heterogeneity analysis, and slight editorial adjustments, the paper will meet PLOS ONE's publication standards and provide actionable insights for maternal HIV prevention and control programs across the continent.

Authors' response: Overall, we have expanded the heterogeneity analyses, clarified the methodology details, and made the required editorial changes. In our opinion, these revisions ensure PLOS ONE's publishing requirements and enhance its applicability for improving maternal HIV prevention and control initiatives in Africa.

Reviewer #6: Authors section: I suggest you only use Names, instead of Initials Delete, let's only have correspondent Authors. The email addresses for each will be put in the PLOS ONE form as you submit the manuscript.

Add **Problem Statement**, and **Objectives**. Research questions without Problem statement are hanging. I suggest you add

1. Problem statement
2. Significance of this Systematic Review

   Refer to the attached manuscript with track changes

Authors' response: Thank you very much for these constructive and helpful comments. We have revised the Authors' section as suggested. Full author names are now used instead of initials. In addition, only the corresponding author is indicated in the manuscript, and individual author email addresses have been removed from the main text.

According to the comment, we have added the significance of the study to clearly show the importance, relevance, and potential contribution of this review to policy, practice, and future research. In the "Introduction section", we have clearly described the background, triggers, existing gaps in the literature, and the rationale for conducting this systematic review.

Reviewer #7: Page 139-143: The problem needs to be further refined, especially for research issues concerning viral load viruses. Ethical clearance issues also sometimes become obstacles

and the lack of study in HIV research. page 168 -175: Please clarify the source of the data taken, because in the background of the problem, you describe something that highlights the lack of research in the field regarding this topic.

Authors' response: We have refined the problem statement to more clearly articulate the key research gaps related to viral load suppression among HIV-positive pregnant and lactating women. Of course, there are ethical issues and require to adhere ethical considerations for HIV patients to undergo primary studies. This systematic review was conducted in accordance with the PRISMA guidelines, ensuring transparency, methodological rigor, and ethical reporting standards.

Reviewer #7: page 194-200: Could you provide more details on the research method conducted with a pooled prevalence, including which data was used and the operational definitions applied?

Authors' response: We have revised the Methods section to provide a clearer description of the data sources used to estimate the pooled prevalence, the inclusion and extraction procedures, and the statistical analysis. We have also added explicit operational definitions for outcome variable (Virologic suppression) to enhance clarity and reader friendly.

Reviewer #7: page 211- 220: is it only using this data to determine the pooled prevalence?

Authors' response: Thank you very much. We confirm that the pooled prevalence was estimated using data extracted from the included studies. Standard errors of prevalence were calculated and, where appropriate, logit-transformed prevalence estimates were applied to stabilize variance before pooling. Then, data exported to Stata 17 for further statistical analysis. A random-effects model with Restricted maximum–likelihood (REML) was used to determine the pooled prevalence of virologic suppression. We performed some important changes in the Methods section.

Reviewer #7: page 231-233: Why you leave-one-out sensitivity analysis?

Authors' response: Thank you for the query. A leave-one-out sensitivity analysis was conducted to assess the influence of each individual study on the pooled estimate and to evaluate the robustness of the findings in the presence of heterogeneity. This analysis helped determine whether any single study disproportionately affected the overall results. Methods and Results

sections.

Reviewer #7: page 255 - 256: can you more explain with this result?

Authors' response: We thank the reviewer for requesting clarification. The high level of heterogeneity (I^2 = 99.57%) indicates substantial variability in effect estimates across the included studies beyond probability. For this reason, a random-effects model was applied to account for between-study variability and to provide a more conservative pooled estimate. Accordingly, we have revised in the main text.

Reviewer #7: page 351 -356: This explanation must be further clarified and supported by literature that underpins this statement.

Authors' response: We appreciate the reviewer's idea. Appropriate references are now provided to support the scientific justification.

Reviewer #7: page 417: Although this research is a systematic review and meta-analysis using secondary data, researchers must still consider intellectual property rights and proper attribution for the data used.

Authors' response: Absolutely. We have ensured that all data sources are appropriately acknowledged and attributed while respecting intellectual property rights. References and acknowledgments have been thoroughly examined and updated in the manuscript to provide proper credit to the original works.

Reviewer #8: Given the high prevalence of HIV-positive women in African countries, it is important to understand virological suppression in pregnant and breastfeeding women. Conducting this systematic review provides insight into the current status of this population.

Authors' response: Thank you for providing general and specific comments and suggestions.

Reviewer #8: The authors have considered the necessary methodological criteria and clearly described the process for developing this systematic review. However, it is not entirely clear how they analyzed and interpreted the determinants of virological suppression and associated variables, nor how they addressed potential confounders. It would be helpful to detail these aspects in the review manuscript.

Authors' response: We thank Reviewer #8 for this constructive comment. In response, we have modified the Results and Discussion sections to further explain how factors of virologic suppression and associated variables were analyzed using meta-analysis and systematic synthesis.

Reviewer #8: When evaluating individual studies, why was the Critical Appraisal Checklist considered instead of a validated tool for assessing the methodological quality of studies? Potential biases arising from the study assessment are not addressed in the limitations section of the review. Please comment.

Authors' response: We thank the reviewer for this important comment. We employed the JBI Critical Appraisal Checklist, a widely recognized and comprehensive tool designed to evaluate the methodological quality of diverse study designs typically included in systematic reviews. Given its rigor and standardized approach, we believe that potential biases arising from study assessment have been sufficiently addressed, and additional assessment of such biases is not necessary.

Reviewer #9: Thank you for doing research on his interesting topic. It is essential to emphasize the importance of implementing targeted interventions for young HIV-positive women, including the disclosure of HIV status, initiation of first-line antiretroviral therapy (ART) regimens, and promotion of ART adherence.

Authors' response: We sincerely thank the reviewer for highlighting this important point. Accordingly, we have emphasized the significance of targeted interventions for young HIV-positive women in the revised Discussion and Conclusion sections, including the roles of HIV status disclosure, timely initiation of first-line ART, and strategies to promote adherence. This addition strengthens the practical relevance and public health implications of our findings.

Reviewer #10: The manuscript (PONE-D-25-49195) addresses a critical public health issue concerning HIV viral suppression among pregnant and lactating women on antiretroviral therapy (ART) in Africa. The topic aligns well with global health and HIV epidemiology. The study is comprehensive but requires significant improvements in methodological transparency, reporting, and interpretation. I have the following concerns which if addressed will improve the study.

Authors' response: We sincerely thank the reviewer for recognizing the public health importance of our study and its relevance to HIV epidemiology in Africa. We appreciate your constructive feedback regarding methodological transparency, reporting, and interpretation. In response, we have carefully revised the manuscript to clarify the methodology, improve reporting of results, and strengthen the interpretation in line with the study objectives and current literature. Your insights have greatly helped enhance the clarity, rigor, and overall quality of our work.

Reviewer #10: 1. Prospero has CRD420251077063, registered as "Knowledge of mother-to-child transmission of HIV and its prevention among reproductive-age women in Africa: a systematic and meta-analysis 2025".

- Register your review again in Prospero and state the ID correctly.

Authors' response: We sincerely thank the reviewer for pointing this out. We apologize for the inadvertent error in reporting the PROSPERO ID. The correct study protocol for this systematic review and meta-analysis has been registered on PROSPERO (**ID**: **CRD420251186121**, **https://www.crd.york.ac.uk/PROSPERO/view/CRD420251186121**). The manuscript has been updated to reflect the correct registration information.

Reviewer #10: 2. Under introduction (line 144 – 153), the research question is a repetition from the goal of the study. Merge the goal and research question to avoid repetition.

Authors' response: Thank you in advance. The Introduction has been revised to merge the study goal and research question, eliminating redundancy while maintaining coherence. This change improves the flow and readability of the manuscript.

Reviewer #10: 3. Remove (line 168- 175) and present as part of your supplementary file S2 file.

Authors' response: We sincerely appreciate the reviewer's comment. We have clarified that the keywords listed were used to build search strategies across multiple databases and other search engines. To ensure transparency, the full search strategy is provided in **Supplementary File S2.** Therefore, we wish to retain the current explanation in the manuscript while attached the detailed search strategy as supplementary file.

Reviewer #10: 4. Include a complete PRISMA 2020 checklist and flow diagram under the results as part of the manuscript not part of list of figures (Figure 1). Draw it nicely or get it the flow diagram rom online, present it well in the manuscript, with well labelled figure legend.

Authors' response: Accordingly, we have incorporated a PRISMA 2020 checklist and a well-presented flow diagram directly within the Results section of the manuscript. The list figures are attached separately as a tiff figure in line with the journal guideline. The flow diagram has been clearly formatted with a detailed, well-labeled legend to clearly illustrate the study selection process and referenced in the result section.

Reviewer #10: 5. For the risk of bias assessment, present the JBI quality assessment results in a summary table or figure.

Authors' response: We thank the reviewer for this valuable suggestion. For this reason, we have added a summary table presenting the JBI quality assessment results for all included studies. This addition improves transparency and allows readers to easily interpret the methodological quality of the studies included in our review (S1 file Table 1).

Reviewer #10: 6. Table 1 should be added to the results section with a well described table legend under the table, stating what criteria or reference was used for the risk of bias.

7. Table 2 should be added to the results section, not as part of list of tables, then with a legend describing or stating what was used to achieve the sub-group analysis.

Authors' response: Thank you. Table 1 has been added to the Results section, and a clear legend has been included below the table specifying the criteria and reference used for the risk of bias assessment (JBI score) in the method section. Additionally, Table 2 also added to the Results section, with a detailed legend explaining the criteria and data used to perform the subgroup analyses.

Reviewer #10: 8. Figure 1-8 should be presented as part of the results with a well labelled figure legend, stating how it was achieved.

Authors' response: Thank you dear reviewer for this suggestion. We have tried to give clear and detailed legend describing the methods and data used to generate the figures.

Reviewer #10: 9. Consider sources of heterogeneity and use meta-regression by study year, region/country, ART regimen and viral load threshold to improve the robustness of the findings.

Authors' response: We thank you the reviewer for this insightful suggestion. We have carefully considered potential sources of heterogeneity, including publication year, region/country, ART regimen, and study design. Meta-regression analyses were conducted based on the above variables. The results have been included in the revised manuscript to provide a stronger understanding of factors contributing to heterogeneity.

Reviewer #10: 10. Include in the discussion, a more clearly link between the UNAIDS 95-95-95 targets, national PMTCT programs and strategies to address low suppression in younger women. Overall, this review is timely and relevant. Addressing the above comments will substantially enhance its scientific rigor, methodological transparency, and readability.

Authors' response: We truly thank the reviewer for this constructive feedback. As per the reviewer comment, we have revised the Discussion to explicitly link our findings to the UNAIDS 95-95-95 targets and national PMTCT programs, highlighting strategies to improve viral suppression among younger women.

Reviewer #11: Overall, this is good research, this review takes a deep dive into how well HIV-positive pregnant and breastfeeding women in Africa are responding to antiretroviral therapy, specifically looking at how many are achieving virologic suppression. The study is methodologically solid, using validated tools and statistical models to analyze the data. It also identifies key factors that influence outcomes, like age, relationship status, ART regimen, HIV status disclosure, and adherence. Younger women and those with poor adherence were less likely to achieve suppression, pointing to the need for youth-friendly care and stronger support systems. While the results are promising, the high variability across studies and regions suggests more targeted interventions and further research are needed to close the gap and reduce mother-to-child transmission.

Authors' response: To sum up, the authors are grateful for Reviewer #11 detailed and insightful assessment. We are grateful that the study's methodological rigor, thorough analysis, and identification of important factors influencing virologic suppression in HIV-positive

pregnant and breastfeeding women in Africa were acknowledged. We particularly appreciate the reviewer's observations on the challenges younger women and those with poor adherence encounter, as well as the necessity of youth-friendly care and targeted interventions. We agreed that these findings emphasize the significance of specific actions and further study to close gaps and lower mother-to-child HIV transmission, even though we acknowledge the high heterogeneity among studies in Africa.

Reviewer #12: 1. General Assessment

This manuscript presents a systematic review and meta-analysis evaluating the prevalence of virologic suppression and its associated factors among HIV-positive pregnant and lactating women receiving ART in Africa. The study is timely, methodologically sound, and addresses a critical gap in maternal HIV care across the continent. The authors adhered to PRISMA guidelines, registered their protocol on PROSPERO, and employed appropriate statistical techniques including random-effects modeling, subgroup analysis, and sensitivity testing. The manuscript is generally well-written and organized, with clear objectives and relevant findings. However, several areas require clarification or refinement to meet PLOS ONE's standards for transparency, reproducibility, and scientific rigor.

Authors' response: We truly valued Reviewer #12's extensive and informative review. We are grateful that the study's relevance, rigor of methodology, adherence to PRISMA guidelines, PROSPERO registration, and application of reliable statistical techniques were acknowledged. To improve transparency, consistency, and scientific rigor, we have addressed the given suggestions and improvements. As a result, the revised manuscript meets PLOS ONE's standards.

Reviewer #12 2. Section-by-Section Evaluation

Methods and Materials

Areas for Improvement:

1. Search Strategy: The Boolean search string is extensive but lacks structure. Present full search strategies (e.g., for PubMed) in a supplementary file.

Authors' response: Thank you very much for this important comment. The main text outlines the keywords used to construct search strategies across various databases and search engines. Additionally, the complete, structured search strategy for each database, including PubMed, has been provided as a **supplementary file (S2 file)** to ensure full transparency and reproducibility.

Reviewer #12 2. Database Justification: Justify inclusion of Google Scholar due to its non-standard indexing.

Authors' response: We thank the reviewer for this important comment. Google Scholar was included as a supplementary search engine to capture all relevant articles, particularly unpublished (grey) literature, thereby helping to minimize publication bias. Its use complements standard databases and ensures a more comprehensive and inclusive evidence base for the review.

Reviewer #12 3. Quality Threshold: Rephrase "excellent quality" for studies scoring ≥5 on JBI to "acceptable quality."

Authors' response: We thank the reviewer for this suggestion. We have revised the manuscript to replace "excellent quality" with "acceptable quality" for studies scoring ≥5 on the JBI checklist.

Reviewer #12: 4. Outcome Definitions: Clarify whether all studies used the same VL threshold (≤1000 copies/mL).

Authors' response: We thank the reviewer for this comment. To ensure consistency, we included only studies that used the same viral load threshold (≤1000 copies/mL). The Methods section has been revised and updated accordingly to reflect this.

Reviewer #12: 5. Statistical Rationale: Justify use of Der Simonian and Laird's method over alternatives like Hartung-Knapp.

Authors' response: We thank the reviewer for this insightful comment. We compared and contrast the pooled estimates of virologic suppression using three random-effects meta-analysis estimators: Der Simonian–Laird (DL), Sidik–Jonkman (SJ), and restricted maximum likelihood (REML). Finally, **we convinced to use a random-effects model with REML because it is a**

widely recognized and commonly applied method for pooling prevalence estimates in meta-analyses with high heterogeneity. The REML approach provides a more accurate and less biased estimate of virologic suppression. However, Der Simonian–Laird (DL) underestimates the between-study heterogeneity (τ^2). We have accordingly applied REML model consistently across the revised manuscript, from the beginning to the conclusion section of the manuscript.

Reviewer #12: 6. Publication Bias Tests: Clarify whether Egger's and Begg's tests were applied to both prevalence and AORs.

Authors' response: We thank the reviewer for this comment. The Egger's and Begg's tests were applied to assess publication bias for the pooled prevalence estimates. For the pooled AORs, the number of included studies was limited, making these tests less reliable.

Reviewer #12: Results

Areas for Improvement:

1. PRISMA Flow: Include a complete PRISMA diagram and a table of excluded studies with reasons.

Authors' response: Thank you for the comment. We have included a complete PRISMA 2020 flow diagram for study selection in the Results section.

Reviewer #12: 2. Heterogeneity: $I^2 = 99.57\%$ is extremely high. Consider meta-regression or deeper exploration of sources.

Authors' response: Dear reviewer thanks you for this insightful comment. Given that our review included diverse studies across Africa, high heterogeneity was anticipated. Accordingly, we explored potential sources of heterogeneity using subgroup and sensitivity analyses. Following the reviewer's recommendation, we also conducted meta-regression analyses to further investigate and account for possible sources of heterogeneity, and the results have been included in the revised manuscript.

Reviewer #12: 3. Subgroup Analysis: Report statistical significance of subgroup differences and I^2 values per subgroup.

Authors' response: We have reported in the main text.

Reviewer #12: 4. Associated Factors: Provide AORs and CIs for all discussed predictors. Clarify model type used for pooling.

Authors' response: Thank you. The statistical significance of subgroup differences and I^2 values for each subgroup have now been reported and are presented in Table 2 of the revised manuscript.

Reviewer #12: 5. Figures: Ensure all figures (e.g., funnel plot, forest plots) are clearly labeled and referenced.

Authors' response: We thank the reviewer for this comment. We have ensured that all figures, including funnel and forest plots, are clearly labeled and properly referenced in the manuscript.

Reviewer #12: Discussion, Strengths & Limitations, Conclusion

Areas for Improvement:

1. Interpretation: Discuss clinical significance of the 14% gap from the 95% target.

Authors' response: Dear reviewer, thank you very much for this valuable comment. We have made a notable revision in the discussion part that show the implication of the 14% gap behind the USAIDS the 95% target in PMTCT.

Reviewer #12: 2. Causal Language: Soften causal claims to reflect observational nature (e.g., "associated with").

Authors' response: We thank the reviewer for this important comment. We have revised the manuscript to soften causal language throughout, using terms such as "associated with" to accurately reflect the observational nature of the included studies.

Reviewer #12: 3. Policy Framing: Reference WHO/UNAIDS frameworks when recommending interventions.

Authors' response: We have acknowledged the WHO/UNAIDS frameworks for recommended interventions to achieve the 95% VS target.

Reviewer #12: 4. Limitations: Clarify geographic skew and discuss potential impact of publication bias despite non-significant tests.

Authors' response: The manuscript has been revised to present actionable interventions aligned with WHO and UNAIDS frameworks, ensuring that our policy recommendations are evidence-based international guidelines.

Reviewer #12: 5. Conclusion: Separate findings from recommendations for clarity. Specify which health system components need strengthening.

Reviewer #12:

3. Minor Editorial Suggestions

• Correct grammar and phrasing (e.g., "All searching databases" → "All searched databases").

• Ensure consistent terminology: "virologic suppression" vs. "viral load suppression."

• Fix typos (e.g., "studies is required" → "studies are required").

Authors' response: We thank the reviewer for these helpful editorial suggestions. We have carefully corrected grammar and phrasing, ensured consistent terminology throughout the manuscript, and addressed identified typos to enhance clarity and readability.
