# Supplementary material for: Virologic suppression among HIV-positive pregnant and lactating women receiving antiretroviral therapy in Africa: A systematic review and meta-analysis
Source: PLoS One. 2026 Apr 13;21(4):e0346045. doi: 10.1371/journal.pone.0346045 (PMC13075719; doi:10.1371/journal.pone.0346045)
Supplement: Attachment — Submitted filename: Response_to_Reviewers_auresp_2.pdf [file pone.0346045.s018.pdf]

February 12, 2026

Virologic suppression among HIV-positive pregnant and lactating women receiving antiretroviral therapy in Africa: A systematic review and meta-analysis

Manuscript number: PONE-D-25-49195R1

Dear Richard Makurumidze (MBChB, MPH, PhD, FCPHP, Academic Editor),

We are delighted to receive your comments on our submission to PLOS ONE. We are also genuinely grateful to the esteemed reviewers; their scientific insights, expertise, and guidance have been invaluable in enhancing the overall quality, clarity, and rigor of our manuscript.

We have carefully and diligently addressed all comments and suggestions provided by the reviewers. All amendments have been clearly highlighted using tracked changes and are fully incorporated into the revised manuscript. Finally, we kindly invite you to review our point-by-point responses below.

Point-by-point response to Reviewers' comments

Reviewer #1: I did not find a point-by-point reply to my recommendations. Next time I would like to find them. I read the revised paper. The revised manuscript effectively addresses most of the reviewer's concerns. It now includes a meta-regression analysis that identifies significant moderators of heterogeneity, acknowledges and discusses the geographic concentration of studies in Southern Africa, and presents expanded discussion on key contextual factors such as socioeconomic status, healthcare access, and rural–urban disparities. The authors have also improved the narrative quality of the discussion, providing clearer interpretations and actionable policy recommendations aligned with the UNAIDS 95-95-95 goals. Additionally, they incorporated relevant factors such as ART duration, viral load assay thresholds, and breastfeeding practices, which were previously under-addressed.

However, a few issues remain unresolved. The manuscript still refers to an implausible study inclusion period extending to April 2025, which undermines credibility. Moreover, while the

Joanna Briggs Institute tool is used for quality assessment, the authors do not provide a detailed summary of individual study scores, limiting transparency. These remaining concerns should be addressed to fully satisfy the reviewer's expectations.

Authors' response: Dear Reviewer,

We would like to express our sincere gratitude for your valuable time and constructive feedback on our manuscript. We greatly appreciate your insightful comments and the opportunity to benefit from your expertise in improving the quality and clarity of our work. In response to your thoughtful comments and suggestions, we have carefully revised the manuscript and thoroughly rechecked all relevant sections to address the points raised.

Regarding your first concern, this study was conceptualized in March 2025 and required more than four months to complete the review process and prepare the final manuscript for submission. We conducted the final literature search across all selected databases through the end of July 2025, and the manuscript was submitted to PLOS ONE in early September 2025.

The review was designed to include studies conducted from January 2016 onward, marking the commencement of the Sustainable Development Goals (SDGs), or from the end of 2015, which signified the conclusion of the Millennium Development Goals (MDGs). This timeframe intentionally selected to serve as a benchmark for assessing progress in the SDG era. Accordingly, our analysis covers nearly a decade of evidence, spanning from the end of 2015 to 2025.

With respect to the Joanna Briggs Institute (JBI) critical appraisal scores and transparency, we have now clearly included the JBI score for each included study in the supplementary material, as presented in "S3 File: Summary of Studies." This addition enhances the transparency and methodological rigor of the review. We hope that these revisions adequately address your concerns, and we sincerely appreciate your guidance in strengthening our manuscript.

Reviewer #7: I noticed in this journal writing that you used PROSPERO, but after I checked, it was only at the initial screening stage up to completion. The same applies to the subsequent stages, which are the most important in PROSPERO, namely data extraction, bias/quality assessment, and data synthesis. Could you explain this further? Some of the arguments, results, and conclusions do not sufficiently elaborate on the analysis that demonstrates the novelty and urgency of this research.

Authors' response: We sincerely appreciate the reviewer's careful observation and constructive feedback. We confirm that the review was registered in PROSPERO during the initial stages of screening and study selection. All subsequent procedures, including data extraction, quality and risk-of-bias assessment, data management, and evidence synthesis, were conducted strictly in accordance with the registered protocol. We have now included the PROSPERO registration ID and the corresponding online link in the revised manuscript to enhance transparency and methodological rigor. If any additional clarification or updates required, we will promptly verify and amend the PROSPERO record as needed.

Furthermore, we have carefully addressed the reviewer's comments regarding the need to strengthen the background, clarify the urgency and novelty of the topic, and more clearly articulate the study's contribution. All revisions have been clearly marked using tracked changes and are reflected in the revised manuscript. We are grateful for the reviewer's insightful comments, which have significantly strengthened the quality and clarity of our work.

Reviewer's comments: In this explanation, it is necessary to provide a more detailed explanation and analysis of the background as to why the limitations of this study are important as future research that should be conducted.

Authors' response: We thank the reviewer for this insightful comment. We sincerely thank the reviewer for this insightful and constructive comment. We acknowledge that the limitations of our study, particularly the uneven geographic representation and the substantial variability across included studies, reflect important gaps in the existing evidence base. These limitations may affect the generalizability and comparability of the findings across different settings. Addressing these gaps through future research is essential to strengthen the applicability of evidence and to better inform context-specific interventions and policy decisions.

In response to the reviewer's suggestion, we have expanded the limitations section to provide a more detailed and transparent discussion of these issues and their potential implications for interpretation of the results. We have also emphasized the need for geographically representative, methodologically rigorous, and standardized studies to improve the quality and consistency of evidence in this field. Additionally, we have outlined specific recommendations for future research aimed at overcoming the limitations identified in the current study.

All revisions have been incorporated into the revised manuscript and are clearly indicated.

Authors' Concluding Response:

In conclusion, we have undertaken substantial and thorough revisions to the manuscript, particularly during the first round of review, to comprehensively address the reviewers' comments and suggestions. We have also carefully responded to the additional points raised during the second round of revision.

We sincerely appreciate the Academic Editor for handling and facilitating the review process and for providing thoughtful and ongoing guidance. We are equally grateful to all reviewers for their timely, constructive, and insightful feedback. Their comments have been instrumental in strengthening the manuscript across multiple dimensions, including clarity of content, scope and significance, coherence, methodological transparency and rigor, statistical analysis and data synthesis, interpretation of findings, and the balanced discussion of the study's strengths and limitations.

We would like to extend our appreciation to Reviewer #1, Reviewer #2, Reviewer #3, Reviewer #6, Reviewer #7, Reviewer #9, Reviewer #10 **(Second round review)**, and all other reviewers for their careful evaluation and for recognizing the relevance and scientific contribution of this systematic review and meta-analysis to the broader scientific community. Finally, we really appreciate your continued time, thoughtful consideration, and invaluable support throughout the review process.
