# Supplementary material for: Virologic suppression among HIV-positive pregnant and lactating women receiving antiretroviral therapy in Africa: A systematic review and meta-analysis
Source: PLoS One. 2026 Apr 13;21(4):e0346045. doi: 10.1371/journal.pone.0346045 (PMC13075719; doi:10.1371/journal.pone.0346045)
Supplement: Attachment — Submitted filename: Review comments on PONE-D-25-49195.docx [file pone.0346045.s009.docx]

Review comments on **“Virologic suppression among HIV-positive pregnant and lactating women receiving antiretroviral therapy in Africa: A systematic review and meta-analysis (PONE-D-25-49195)”**

The manuscript (PONE-D-25-49195) addresses a critical public health issue concerning HIV viral suppression among pregnant and lactating women on antiretroviral therapy (ART) in Africa. The topic aligns well with global health and HIV epidemiology. The study is comprehensive but requires significant improvements in methodological transparency, reporting, and interpretation. I have the following concerns which if addressed will improve the study.

1.Prospero has CRD420251077063, registered as “Knowledge of mother-to-child transmission of HIV and its prevention among reproductive-age women in Africa: a systematic and meta-analysis 2025”.

- Register your review again in Prospero and state the ID correctly.

2. Under introduction (line 144 – 153), the research question is a repetition from the goal of the study. Merge the goal and research question to avoid repetition.

3. Remove (line 168- 175) and present as part of your supplementary file S2 file.

4. Include a complete PRISMA 2020 checklist and flow diagram under the results as part of the manuscript not part of list of figures (Figure 1). Draw it nicely or get it the flow diagram rom online, present it well in the manuscript, with well labelled figure legend.

5. For the risk of bias assessment, present the JBI quality assessment results in a summary table or figure.

6. Table 1 should be added to the results section with a well described table legend under the table, stating what criteria or reference was used for the risk of bias.

7. Table 2 should be added to the results section, not as part of list of tables, then with a legend describing or stating what was used to achieve the sub-group analysis.

8. Figure 1-8 should be presented as part of the results with a well labelled figure legend, stating how it was achieved.

9. Consider sources of heterogeneity and use meta-regression by study year, region/country, ART regimen and viral load threshold to improve the robustness of the findings.

10.Include in the discussion, a more clearly link between the UNAIDS 95-95-95 targets, national PMTCT programs and strategies to address low suppression in younger women.

Overall, this review is timely and relevant. Addressing the above comments will substantially enhance its scientific rigor, methodological transparency, and readability.

END.
