# Supplementary material for: Virologic suppression among HIV-positive pregnant and lactating women receiving antiretroviral therapy in Africa: A systematic review and meta-analysis
Source: PLoS One. 2026 Apr 13;21(4):e0346045. doi: 10.1371/journal.pone.0346045 (PMC13075719; doi:10.1371/journal.pone.0346045)
Supplement: Attachment — Submitted filename: 23012026_Review comments.docx [file pone.0346045.s014.docx]

**Reviewer #10**

Review comments on “**Virologic suppression among HIV-positive pregnant and lactating women receiving antiretroviral therapy in Africa: A systematic review and meta-analysis (PONE-D-25-49195)”**

I have carefully reviewed the revised manuscript and the authors’ point-by-point responses. The authors have adequately and thoughtfully addressed all the comments raised. The PROSPERO registration has been done again and correctly clarified, redundancies in the introduction have been resolved, and methodological transparency has been substantially improved through the inclusion of a complete PRISMA 2020 checklist and a clearly presented flow diagram.

Importantly, the addition of meta-regression analyses strengthens the robustness of the findings, and the revised discussion now clearly aligns the results with the UNAIDS 95-95-95 targets and national PMTCT programs, highlighting strategies to improve viral suppression among younger women. Overall, the manuscript is scientifically sound, methodologically rigorous, and timely.

I have no further major concerns and recommend the manuscript for acceptance.

END
